# Stereoselective two-carbon ring expansion of allylic amines *via* electronic control of palladium-promoted equilibria†

**DOI:** 10.1039/d3sc02303f

**Published:** 2023-06-06

**Authors:** Charles P. Mikan, Aidan Matthews, Daniel Harris, Charlotte E. McIvor, Paul G. Waddell, Mark T. Sims, Jonathan P. Knowles

**Affiliations:** a Department of Applied Sciences, Northumbria University Newcastle upon Tyne NE1 8ST UK jonathan.p.knowles@northumbria.ac.uk; b School of Natural and Environmental Sciences, Newcastle University Newcastle upon Tyne NE1 7RU UK

## Abstract

General methodologies enabling the two-carbon homologation of pyrrolidine and piperidine systems have yet to be developed. Herein we report that palladium-catalysed allylic amine rearrangements enable efficient two-carbon ring expansion of 2-alkenyl pyrrolidine and piperidines to their azepane and azocane counterparts. Conditions are mild, tolerant of a range of functional groups and the process can occur with high enantioretention. The products formed undergo a range of orthogonal transformations, making them ideal scaffolds for the creation of compound libraries.

## Abstract 2

Palladium-catalysed allylic amine rearrangements enable the ring expansion of 5- and 6-membered cyclic amines to their azepane and azocane homologues. Reaction conditions are mild and tolerate a range of functionality, including aryl halides.

## Graphical abstract

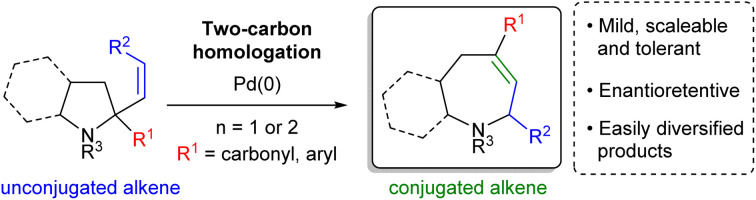

## Introduction

**Fig. 1:**
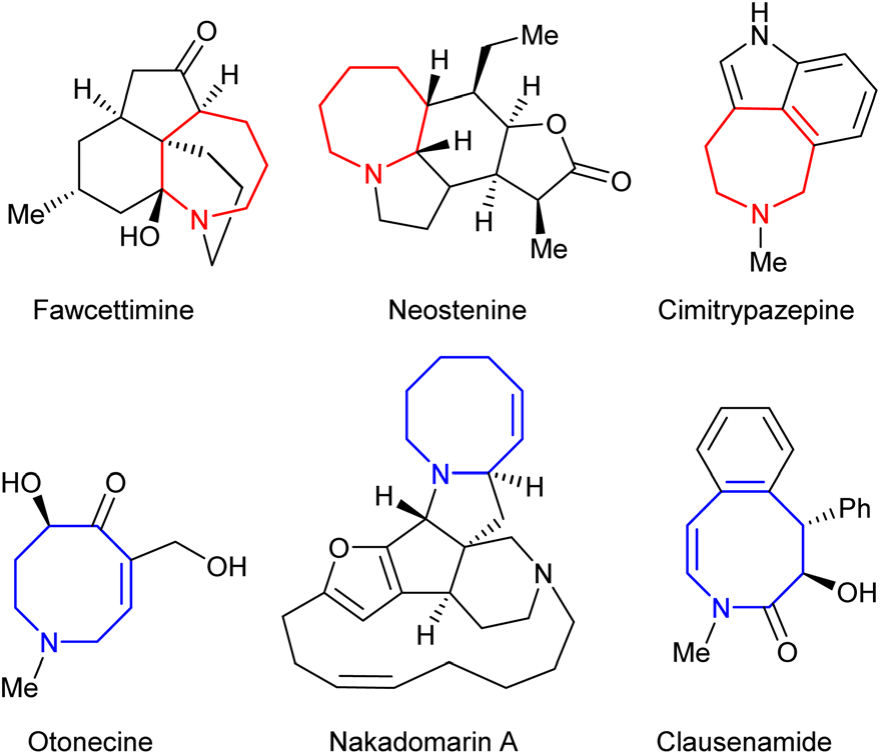
Selected examples of natural products containing azepane and azocane motifs.

Saturated nitrogen-containing heterocycles represent a key class of bioactive molecules, both as natural products and more broadly within medicinal chemistry.^1^ Compounds containing azepane and azocane motifs are of particular interest, as they are widely featured within species possessing potent biological activity as shown within Fig. 1.^2,3^ However, despite considerable effort being directed towards their synthesis, general routes to their construction have yet to be fully developed, thus limiting synthetic access to key chemical space.^4–6^ Ring expansion processes offer an ideal approach to achieving this if they are capable of employing common-ring (*i.e.* 5- to 7-membered) starting materials;^7^ however, the formation of medium rings (*i.e.* 8- to 11-membered) in particular is a well-documented synthetic challenge.^8^

**Scheme 1:**
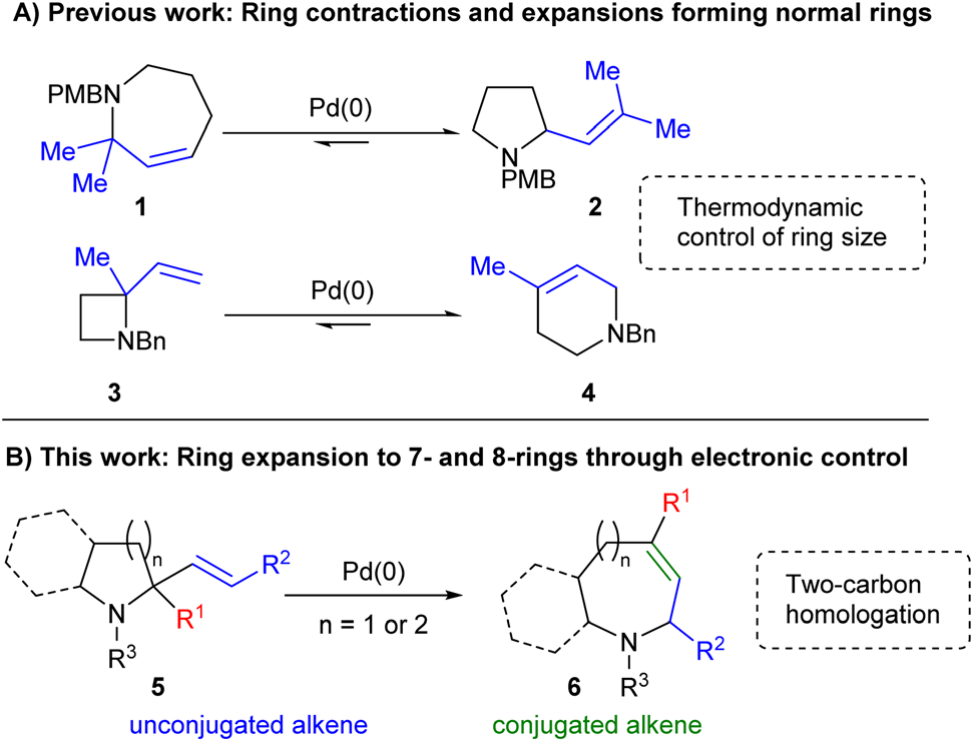
Applications of allylic amine rearrangements.

Previous studies have shown that Pd-catalysed allylic amine rearrangements offer the ability to perform two-carbon ring expansion or contraction of cyclic amines.^9^ However, as equilibria^10^ these reactions were driven purely by reduction of ring strain and were thus restricted to the conversion of strained 4- or 7-ring starting materials into 6- or 5-ring products respectively (Scheme 1A). We hypothesised that the inclusion of appropriate functionality might enable a complete reversal of the selectivity of such processes. As shown in Scheme 1B, the inclusion of an sp^2^-hybridised R^1^ moiety would offer one way of achieving this, with the desired ring expansion reaction bringing the alkene into conjugation with this group in the final product 6. Thus, increased alkene conjugation within the product would provide sufficient drive for the process, overcoming the associated increase in ring strain, thereby switching the expected direction of the reaction towards less easily accessible cyclic systems.

The use of Pd catalysis to achieve this transformation is also attractive as such processes have revolutionized synthetic chemistry since their discovery.^11^ Indeed, their reliability has led to their widespread adoption within medicinal chemistry despite the knowledge that the sp^2^-rich structures formed tend to reduce success within clinical trials.^12^ An approach combining the reliability of Pd catalysis with the ability to form complex, sp^3^-rich structures would therefore be of considerable value within this field. This paper details our successful use of electronic drive to enable the two-carbon ring expansion of 2-alkenyl pyrrolidines and piperidines into their 7- and 8-membered homologues.

## Results and discussion

We initially focused on allylic amine 7b, where ring expansion would form azepane 8b with concurrent migration of the alkene into conjugation with the ester moiety. As an isomerisation process, density functional theory (DFT) studies offer an ideal way to probe the viability of the reaction through direct comparison of the relative energies of the starting material and product.^13^ Pleasingly, we found our hypothesis to be supported by such calculations, which predicted a 5.9 kJ mol^−1^ drive toward azepane 8b (see ESI† for details). We therefore prepared pyrrolidine 7b and studied its ring expansion.

Subjecting pyrrolidine 7b to a range of conditions (Table 1) quickly demonstrated this approach to be feasible, providing modest yields of ring-expanded product 8b (entry 1). However, mass recovery and the *E*/*Z* ratio of the recovered starting material were poor. We therefore investigated the Pd source and ligand, with Pd_2_(dba)_3_ initially appearing most effective (entries 4, 6 and 8). However, *E*/*Z* isomerisation of the alkene moiety (see ESI† for full details) remained problematic. Use of [Pd(allyl)Cl]_2_ initially proved largely ineffective (entries 5 and 7); however, exploration of amine/acid additives and stoichiometry showed greatly improved results in the presence of morpholine (entry 9), while also reducing isomerization. Interestingly, morpholine proved appreciably more effective than apparently similar amines, including piperidine, ethanolamine and *N*-methyl morpholine (see ESI†), suggesting careful control of amine p*K*_a_ and steric demand to be essential. The reaction also proved sensitive to the nature of the acid, with TFA or methanesulfonic acid (entry 10) proving most effective. Indeed, these conditions are remarkably similar to those previously reported.^9^ These conditions also proved successful in other solvents, most notably MeCN (entry 11), allowing reactions to be performed at higher temperature. Use of phosphine ligands under these conditions proved less effective (entry 12), while exploration of bidentate ligands gave little or no conversion.

**Table 1:** Optimisation studies

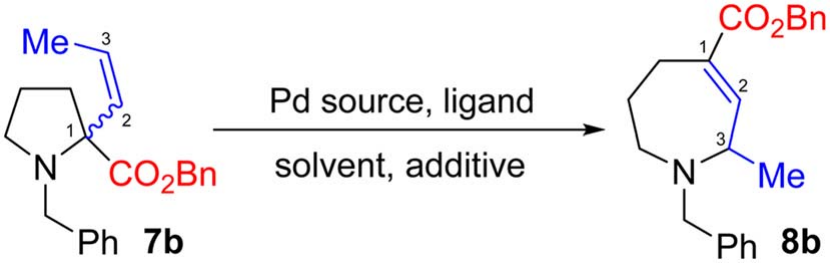				
Entry	Pd source^a^	Ligand	Additive(s) (equiv.)	Yield/%^b^
1^c^	Pd(OAc)_2_	PPh_3_	TFA·^i^Pr_2_NH	30
2^d^	Pd(OAc)_2_	PPh_3_	TFA·^i^Pr_2_NH	<1
3^e^	Pd(OAc)_2_	PPh_3_	TFA·^i^Pr_2_NH	53
4^d^	Pd_2_(dba)_3_	PPh_3_	TFA·^i^Pr_2_NH	55
5^d^	[Pd(allyl)Cl]_2_	PPh_3_	TFA·^i^Pr_2_NH	3
6^d^	Pd_2_(dba)_3_	P(OPh)_3_	TFA·^i^Pr_2_NH	40
7^d^	[Pd(allyl)Cl]_2_	P(OEt)_3_	TFA·^i^Pr_2_NH	20
8^d^	Pd_2_(dba)_3_	P(OEt)_3_	TFA, morpholine (1 : 0.4)	72
9^d^	[Pd(allyl)Cl]_2_	P(OEt)_3_	TFA, morpholine (1 : 0.4)	78 (64)
10^d^	[Pd(allyl)Cl]_2_	P(OEt)_3_	MsOH, morpholine (1 : 0.4)	59
11^e^	[Pd(allyl)Cl]_2_	P(OEt)_3_	TFA, morpholine (1 : 0.4)	75
12^d^	[Pd(allyl)Cl]_2_	PPh_3_	TFA, morpholine (1 : 0.4)	57

a Reactions employing Pd(OAc)_2_ employed 10 mol% catalyst whereas 5 mol% catalyst was used for both [Pd(allyl)Cl]_2_ and Pd_2_(dba)_3_.

b Determined by ^1^H NMR against internal standard, with isolated yields in parentheses.

c Performed in 1,4-dioxane at 100 °C.

d Performed in CH_2_Cl_2_ at 40 °C.

e Performed in MeCN at 80 °C. TFA = trifluoroacetic acid, dba = dibenzylideneacetone, Ms = methanesulfonyl.

**Scheme 2:**
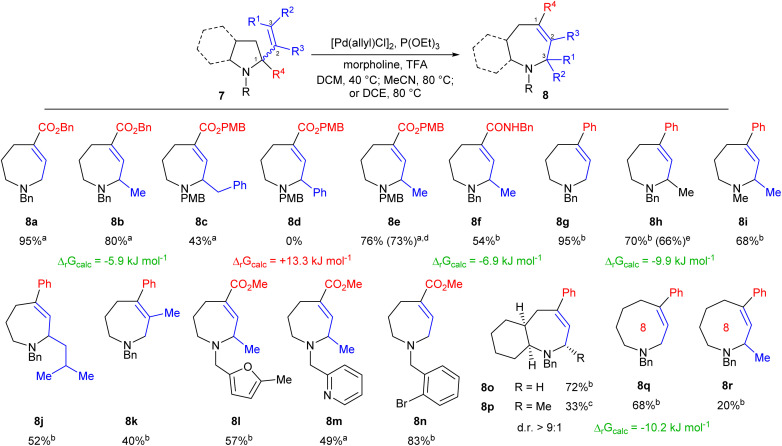
Scope of the ring expansion process and selected calculated Δ_r_*G* values. All reactions employed the *Z*-alkene and 5 mol% [Pd(allyl)Cl]_2_ unless otherwise stated. ^*a*^Performed at 40 °C in DCM. ^*b*^Performed at 80 °C in MeCN. ^*c*^Performed at 80 °C in DCE. ^*d*^Performed on 2 mmol scale with 2.5 mol% [Pd(allyl)Cl]_2_. ^*e*^Starting from *E*-alkene 7h. DCM = dichloromethane; DCE = 1,2-dichloroethane.

With effective conditions identified we next explored the scope of the transformation. Gratifyingly, the reaction was found to tolerate variation of alkene substituents (Scheme 2), with increasing steric bulk leading to a gradual reduction in yield (8a to 8c). Variation of the N-protecting group also proved possible, and PMB-substituted system 8e allowed efficient scale up to 2 mmol with concurrent reduction in catalyst loading to 2.5 mol%. Changing the activating group was also possible, with amides providing sufficient drive for the formation of azepane 8f. While aromatic rings were tolerated (*e.g.*8c) phenyl-substituted alkenes proved essentially unreactive (8d), reflecting the conjugation of the alkene within the starting material removing the required thermodynamic drive. Both such results were again supported by DFT calculations.

We next considered whether aromatic rings might instead allow control of the direction of reaction, and thus prepared a series of 2-phenylpyrrolidine derivatives 7g–j. This proved successful, forming the corresponding azepanes in comparable yield to that seen for their ester analogues, and with somewhat greater tolerance of steric bulk within the alkene substituents as shown by the formation of 8j. Further, simple *N*-methyl system 8i also formed effectively, demonstrating that the key drive of the reaction is electronic rather than steric. *E*-Alkenes proved similarly reactive as shown for 8h and geminally disubstituted alkenes were also found to undergo ring expansion, forming 8k in moderate yield. The methodology also showed appreciable functional group tolerance, with heteroaromatic moieties having limited impact on yield as shown by the synthesis of compounds 8l and 8m. The formation of pyridine 8m is particularly welcome given the reaction's reliance on acid as an activator, and both examples also demonstrate the ability of simple methyl esters to function as activating groups. Importantly, sp^2^-hybridised C–Br bonds were also well tolerated as shown by the efficient formation of 8n, allowing the methodology to efficiently mesh with established cross-coupling based diversification strategies.

We then explored the expansion of more complex ring systems. Bicyclic systems 7o and 7p were prepared using reported approaches^14^ and reacted under standard reaction conditions. Bicycle 8o was formed in high yield, demonstrating the power of the methodology to efficiently form complex fused ring systems. Methyl-substituted system 8p was also formed successfully, albeit in lower yield after an extended reaction time; however, we were pleased to see that the resulting product was formed with a strong preference for one diastereomer (d.r. > 9 : 1), with the methyl substituent adopting a pseudo-equatorial conformation. We then explored whether the methodology could be applied to the appreciably more challenging formation of medium rings. Phenylpiperidine analogues were prepared^15^ and subjected to our standard conditions. Gratifyingly, this proved successful, furnishing 8-ring system 8q in good yield, thus providing direct conversion of a simple piperidine to its azocane homologue. Substitution of the alkene was also possible, albeit more challenging, with Me-substituted 8-ring 8r being formed in 20% yield due to a competing β-hydride elimination process, consistent with the well-known challenge of accessing medium rings.^8^ Again, reaction feasibility was supported by DFT calculation, underlining the predictive power of this approach.

**Scheme 3:**
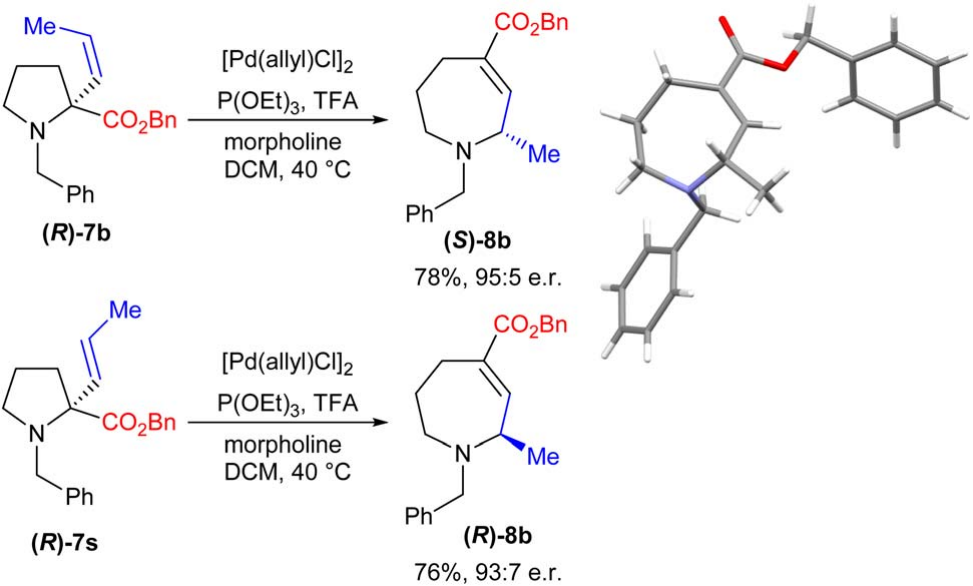
Studies of the enantioretention of the process.

The diastereoselectivity observed in the reaction to form bicyclic amine 8p led us to consider whether the process might also allow enantioretention when using simpler enantiopure starting materials. We therefore prepared enantiopure^16^ (*R*)-7b and subjected it to our reaction conditions. Gratifyingly, this proved to be successful, yielding product (*S*)-8b with an e.r. of 95 : 5, with the absolute stereochemistry proven by XRD (Scheme 3). Further, the use of enantiopure *E*-isomer (*R*)-7s led to the formation of the opposite enantiomer with comparable levels of enantiopurity.

This level of stereoretention is intriguing given that such processes are demonstrably equilibria (*vide infra*), and epimerization of the product might be expected. Despite this, running the reaction for up to 48 h led to only minimal erosion of the e.r., demonstrating that any epimerization is slow. Overall, this process thus allows the conversion of simple l-proline-derived pyrrolidines into either azepane enantiomer with high selectivity.

**Scheme 4:**
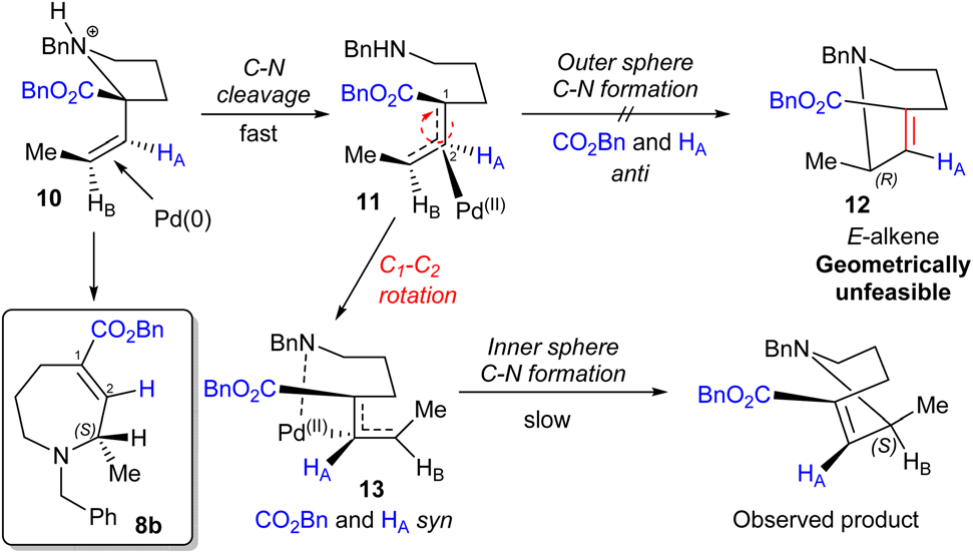
Proposed mechanism.

Trapping reactions were also attempted; however, ring expansion of amine 7e in the presence of electrophiles formed no alternative products. Indeed, in the case of acetic anhydride the reaction gave clean conversion to the standard ring expansion product 8e, thereby apparently ruling out a nucleophilic free secondary amine as an intermediate. Taken together, these observations are consistent with the mechanism depicted in Scheme 4, in which rapid acid-assisted C–N cleavage occurs in the presence of Pd(0). This is presumed to occur in an *anti* fashion based on previous reports,^17^ leading to an intermediate which is then unable to undergo immediate outer sphere C–N bond formation as this would result in the geometrically unfeasible *E*-alkene 12. The complex therefore appears to undergo C_1_–C_2_ rotation, potentially aided by the adjacent ester,^18^ to form N–Pd intermediate 13 which is presumed to be the non-nucleophilic catalyst resting state. Following this, C–N bond formation *via* an inner sphere process gives the (*S*)-stereocentre observed. An analogous mechanism starting from (*R*)-7s leads to the formation of (*R*)-8b as observed.

**Scheme 5:**
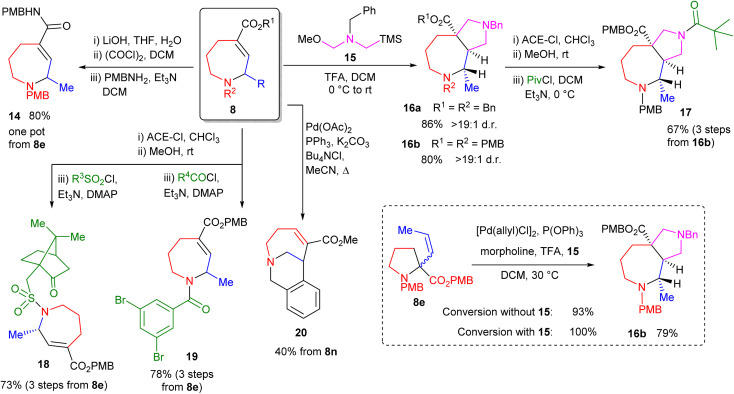
Product derivatisation and *in situ* trapping reactions. DCM = dichloromethane; ACE-Cl = 1-chloroethyl chloroformate.

Finally, we focused on derivatization of the products. As shown in Scheme 5, these compounds represent highly modifiable scaffolds in which orthogonal reactivity is readily achieved. Efficient one-pot amidation of the ester moiety is easily accomplished as shown by the formation of amide 14. Further amide library formation was also easily achieved from PMB-substituted product 8e, as shown by the formation of sulfonamide 18 and amide 19. In the case of sulfonamide 18, a major diastereomer (d.r. > 9 : 1) is formed when using enantioenriched 8e and (*R*)-camphorsulfonyl chloride. The alkene moiety also appeared an attractive handle for diversification and [3 + 2] dipolar cycloaddition processes^19^ proved highly effective on ester-functionalised alkenes, forming bicyclic products 16. Both reactions showed high diasteroselectivity, with only one diastereomer being detectable. These products also undergo efficient and selective amidation as exemplified by the reaction to form amide 17, in which the *N*-benzyl moiety within 16b is selectively deprotected by reaction with 1-chloroethyl chloroformate. This selectivity appears counterintuitive given such reactions operate by initial nucleophilic attack of the reacting amine, with more electron-rich amines generally reacting in preference.^20^ The process thus appears to operate under steric rather than electronic control. Pd-catalysed reactions of system 8n are also possible as shown by the intramolecular Heck reaction to form bicyclic amine 20 in moderate yield. Combined, these reactions demonstrate the capacity for such systems to generate broad 3D compound libraries, giving the methodology considerable potential for use within medicinal chemistry. Finally, we explored telescoping the allylic amine rearrangement with a [3 + 2] cycloaddition to form 16b. This proved successful, forming the desired compound in a single operation. Further, the reaction was found to proceed to 100% conversion under these conditions (previously 93%), demonstrating that *in situ* cycloaddition reactions can operate concurrently with these Pd-catalysed processes, driving reactions that are necessarily equilibria to completion.

## Conclusions

In conclusion, we have shown that efficient two-carbon ring expansion of pyrrolidines and piperidines can be effected by electronic control of the Pd-catalysed rearrangement of allylic amines. This approach offers direct and stereoselective conversion of easily accessed common-ring substrates into sp^3^-rich azepane and azocane systems. The reactions can proceed with high levels of enantioretention, thereby allowing direct access to enantioenriched non-natural amino acids. The methodology thus also offers access to complex and modifiable scaffolds, which appear of potential value within medicinal chemistry. Further studies are underway to develop a more detailed understanding of the mechanism of this process, and to expand the use of *in situ* secondary reactions to increase conversion in more challenging systems.

## Data availability

Data for all compounds reported in this manuscript are available in the ESI,† which includes experimental details, characterisation, copies of ^1^H and ^13^C NMR spectra and HPLC traces. Crystallographic data for compounds has been deposited at the CCDC with deposition numbers 2257860 (for 8b) and 2257861 (see ESI, page S101†).

## Author contributions

C. P. M., A. M., C. E. M., D. H. and J. P. K. synthesised the compounds. P. G. W. performed X-ray crystallographic studies. M. T. S. performed DFT calculations. J. P. K. conceived and supervised the project. All authors contributed to data analysis and writing of the manuscript.

## Conflicts of interest

There are no conflicts to declare.

## Supplementary Material

SC-014-D3SC02303F-s001

SC-014-D3SC02303F-s002
