# Supplementary material for: Stereoselective two-carbon ring expansion of allylic amines via electronic control of palladium-promoted equilibria
Source: Chem Sci. 2023 Jun 6;14(25):6992–6. doi: 10.1039/d3sc02303f (PMC10306103; doi:10.1039/d3sc02303f)
Supplement: SC-014-D3SC02303F-s001 [file SC-014-D3SC02303F-s001.pdf]

Electronic Supplementary Material (ESI) for Chemical Science.
This journal is © The Royal Society of Chemistry 2023

Stereoselective Two-Carbon Ring Expansion of Allylic Amines via Electronic Control of Palladium-Promoted Equilibria

Charles P. Mikan, [a] Aidan Matthews, [a] Daniel Harris, [a] Charlotte E. McIvor, [a] Paul G. Waddell, [b] Mark T. Sims [a] and and Jonathan P. Knowles* [a]

[a] Department of Applied Sciences, Northumbria University, Newcastle upon Tyne, NE1 8ST, UK.

[b] School of Natural and Environmental Sciences, Newcastle University, Newcastle upon Tyne, NE1 7RU, UK.

Supporting Information

Supporting Information

CONTENTS

1.0 Screening of ring expansion conditions 2

2.0 Preliminary mechanistic studies and in situ cycloaddition reactions 3

3.1 General Materials, Methods and Instrumentation 6

3.2 Preparation of phosphonium salts and 4-methoxybenzyl bromide 6

3.3 Preparation of oxazolidone precursors 7

3.4 Preparation of vinyl oxazolidinone precursors 8

3.5 Preparation of proline-derived allylic amine substrates 10

3.6 Preparation of 2-phenylpyrrolidine allylic amine substrates 15

3.7 Preparation of 2-phenyl-octahydroindole allylic amine substrates 20

3.8 Preparation of 2-phenylpiperidine allylic amine substrates 23

3.9 Preparation of *trans*-alkenyl isomer **7r** 25

3.10 Palladium-catalysed ring expansion reactions 26

3.11 Derivatisation reactions. 32

4.0 NMR Spectra 37

5.0 X-ray Crystallography Data 99

6.0 Density Functional Theory Data 102

7.0 References 102

1.0 Screening of ring expansion conditions

Table S1: Further details of screening studies.

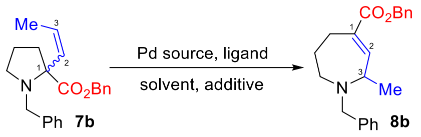

Entry	Pd source[a]	Ligand	Additive(s) (equiv.)	Yield/ %[b]	5 *E*/*Z* ratio	
1[c]	$Pd(OAc)_2$	PPh_3	TFA.$^iPr_2NH$	30	2.25:1	Results from main manuscript
2[d]	$Pd(OAc)_2$	PPh_3	TFA.$^iPr_2NH$	<1	<2:98	
3[e]	$Pd(OAc)_2$	PPh_3	TFA.$^iPr_2NH$	53	7:3	
4[d]	$Pd_2(dba)_3$	PPh_3	TFA.$^iPr_2NH$	55	<2:98	
5[d]	$[Pd(allyl)Cl]_2$	PPh_3	TFA.$^iPr_2NH$	3	<2:98	
6[d]	$Pd_2(dba)_3$	$P(OPh)_3$	TFA.$^iPr_2NH$	40	15:85	
7[d]	$[Pd(allyl)Cl]_2$	$P(OEt)_3$	TFA.$^iPr_2NH$	20	<2:98	
8[d]	$Pd_2(dba)_3$	$P(OEt)_3$	TFA, morpholine (1:0.4)	72	9:1	
9[d]	$[Pd(allyl)Cl]_2$	$P(OEt)_3$	TFA, morpholine (1:0.4)	78 (64)	1:1	
10[e]	$[Pd(allyl)Cl]_2$	$P(OEt)_3$	TFA, morpholine (1:0.4)	75	65:35	
11[d]	$[Pd(allyl)Cl]_2$	PPh_3	TFA, morpholine (1:0.4)	57	13:87	
12[d]	$[Pd(allyl)Cl]_2$	$P(OEt)_3$	TFA, piperidine (1:0.4)	15	<2:98	Variation of amine
13[d]	$[Pd(allyl)Cl]_2$	$P(OEt)_3$	TFA, *N*-Me morpholine (1:0.4)	<1	<2:98	
14[d]	$[Pd(allyl)Cl]_2$	$P(OEt)_3$	TFA, ethanolamine (1:0.4)	20	<2:98	
15[d]	$[Pd(allyl)Cl]_2$	$P(OEt)_3$	MsOH, morpholine (1:0.4)	59	7:93	Variation of acid
16[d]	$[Pd(allyl)Cl]_2$	$P(OEt)_3$	2,4-DNBA, morpholine (1:0.4)	23	<2:98	
17[d]	$[Pd(allyl)Cl]_2$	$P(OEt)_3$	TCA, morpholine (1:0.4)	<1	<2:98	
18[d]	$[Pd(allyl)Cl]_2$	$P(OEt)_3$	$PhCO_2H$, morpholine	<1	<2:98	
19[d]	$[Pd(allyl)Cl]_2$	$P(OEt)_3$	TFA, morpholine (1:0.2)	32	<2:98	Variation of stoichiometry
20[d]	$[Pd(allyl)Cl]_2$	$P(OEt)_3$	TFA, morpholine (1:0.6)	59	1:4	
21[d]	$[Pd(allyl)Cl]_2$	$P(OEt)_3$	TFA only (1)	<1	<2:98	
22[d]	$[Pd(allyl)Cl]_2$	$P(OEt)_3$	Morpholine only (0.4)	<1	<2:98	
23[d]	$[Pd(allyl)Cl]_2$	$P(OEt)_3$	MsOH, morpholine (1:1)	70	85:15	
24[d]	$[Pd(allyl)Cl]_2$	$P(O^iPr)_3$	TFA, morpholine (1:0.4)	17	<2:98	Variation of ligand
25[d]	$[Pd(allyl)Cl]_2$	PCy_3	TFA, morpholine (1:0.4)	<1	<2:98	
26[d]	$[Pd(allyl)Cl]_2$	BINAP	TFA, morpholine (1:0.4)	<1	<2:98	
27[d]	$[Pd(allyl)Cl]_2$	DPEPhos	TFA, morpholine (1:0.4)	<1	<2:98	
28[d]	$[Pd(allyl)Cl]_2$	dppbz	TFA, morpholine (1:0.4)	<1	<2:98	
29[d]	$[Pd(allyl)Cl]_2$	dppp	TFA, morpholine (1:0.4)	<1	<2:98	
30[d]	$[Pd(allyl)Cl]_2$	XantPhos	TFA, morpholine (1:0.4)	4	<2:98	

[a] Reactions employing $Pd(OAc)_2$ employed 10 mol% catalyst whereas 5 mol% was used for both $[Pd(allyl)Cl]_2$ and $Pd_2(dba)_3$. [b] Determined by ^1H NMR against internal standard, with isolated yields in parentheses. [c] Performed in 1,4-dioxane at 100 °C. [d] Performed in CH_2Cl_2 at 40 °C. [e] Performed in MeCN at 80 °C. TFA = trifluoroacetic acid, 2,4- DNBA = 2,4-dinitrobenzoic acid, TCA = trichloroacetic acid.

2.0 Preliminary mechanistic studies and in situ cycloaddition reactions

Reaction of product **8b** under standard reaction conditions.

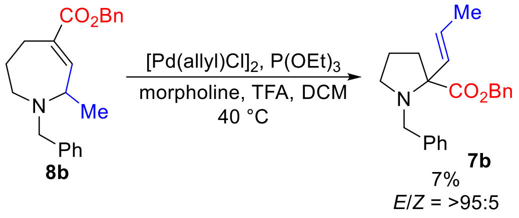

Unsuccessful trapping of intermediates using acetic anhydride.

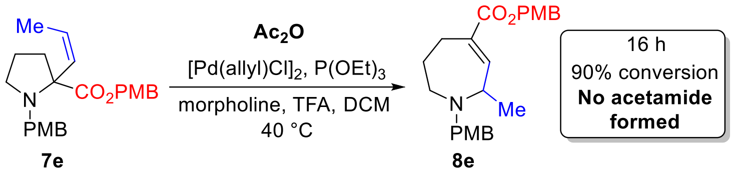

Effect of electron density on reaction conversion at fixed time points.

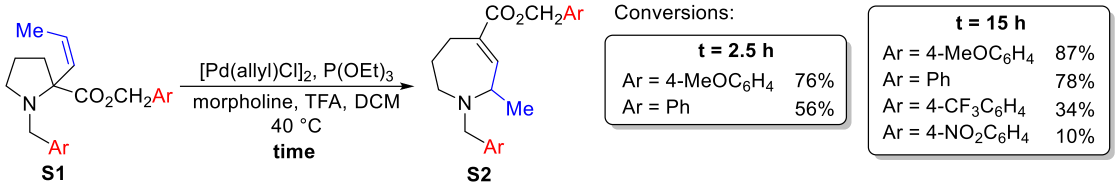

Reaction of *E*/*Z* mixtures of **7b** (from recovered starting materials).

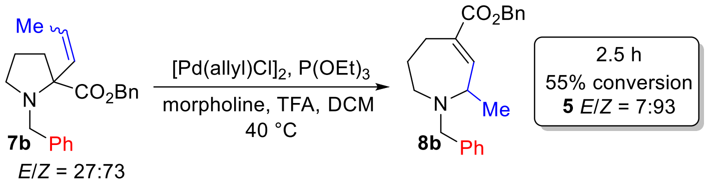

In situ trapping via [3+2]-cycloaddition reaction.

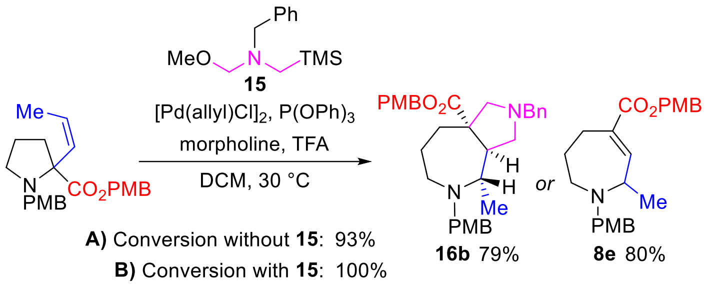

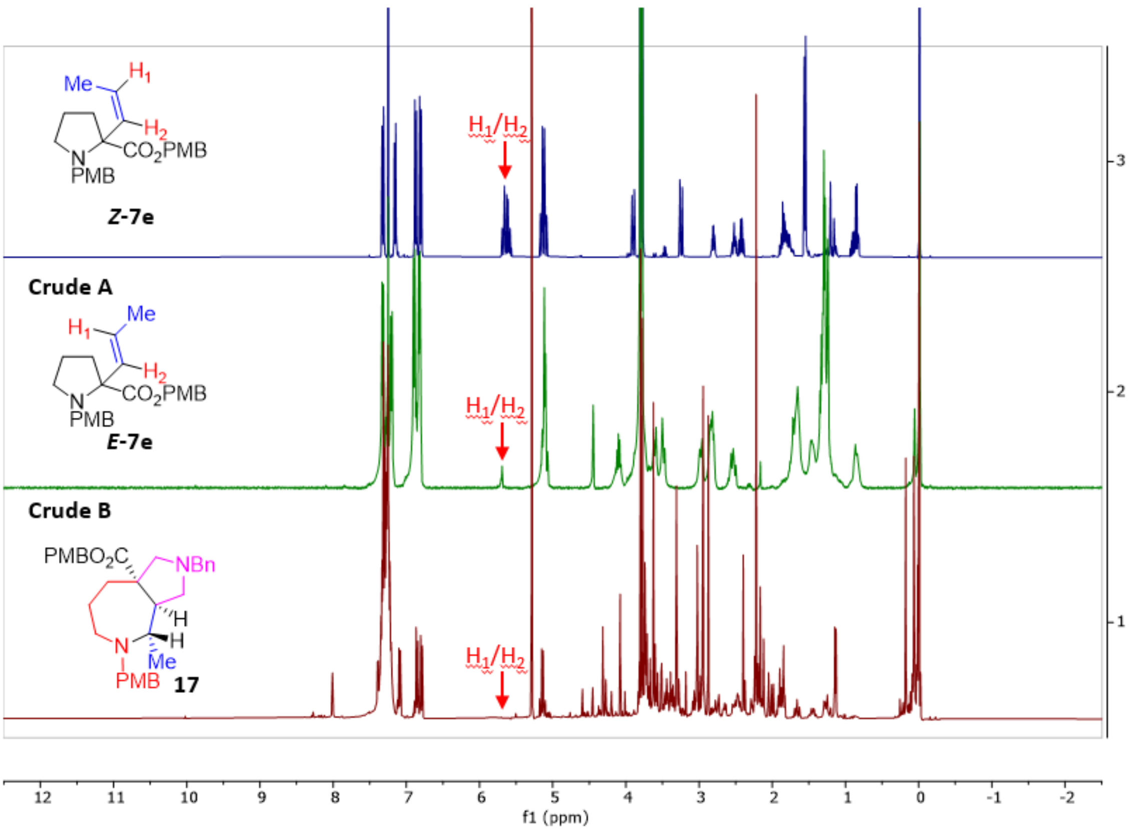

Crude ^{1}H NMR spectrum under conditions A

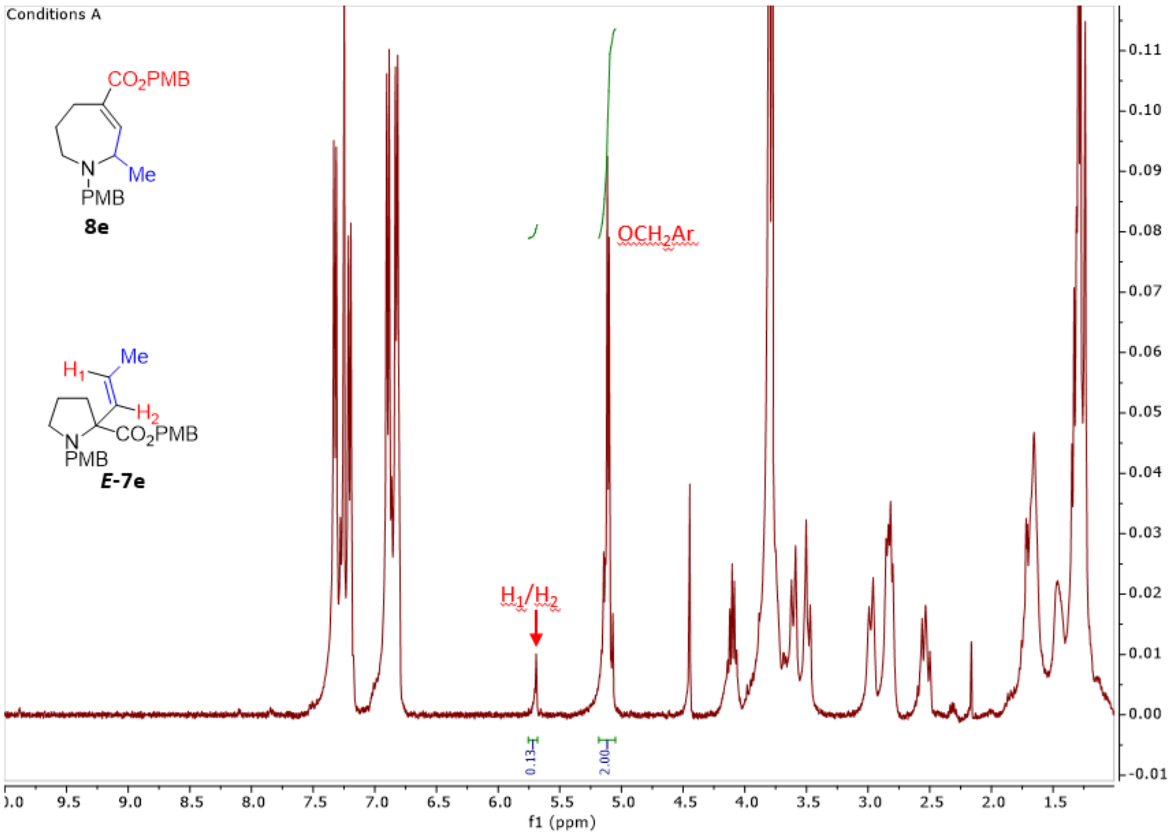

Crude ^{1}H NMR spectrum under conditions B

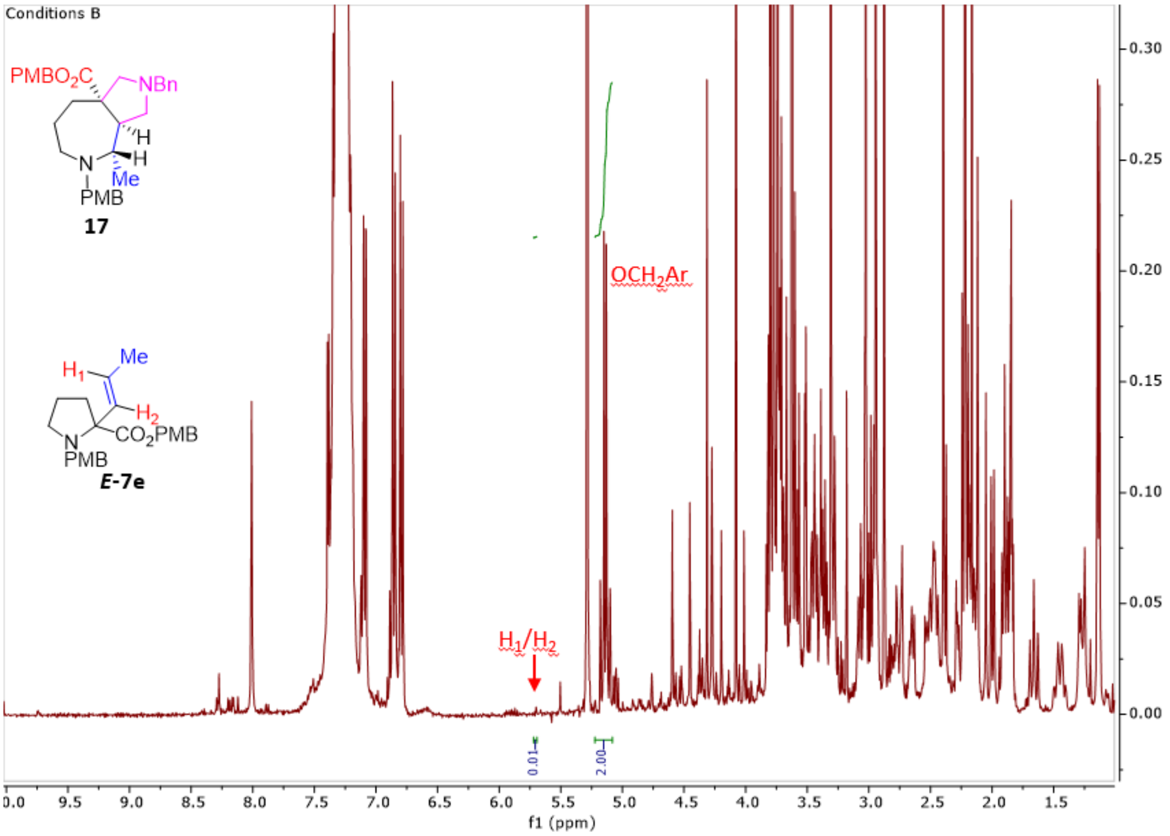

3.1 General Materials, Methods and Instrumentation

All palladium-catalysed processes were carried out using Schlenk technique under argon using either commercially available dry DCM at 42 ˚C or dry MeCN at 80 ˚C unless stated otherwise. Chemicals were obtained from commercial sources unless otherwise stated. DCE, THF, and toluene were dried over activated 3A molecular sieves for 3 days prior to use. Column chromatography was performed using 40-60 mesh silica powder. NMR spectroscopic analysis was performed using Jeol ECS 400 MHz instrument. Chemical shifts are reported in δ ppm. ^{13}C NMR are referenced to solvent as internal standard ($CDCl_3$ or DMSO). Data are reported as follows: chemical shift, multiplicity (s = singlet, d = doublet, t = triplet, q = quartet, p = pentent, h = hextet, hpt= heptet, dd = doublet of doublets, dt = doublet of triplets, td = triplet of doublets, tt = triplet of triplets, ddt = doublet of doublet of triplets, ddd = doublet of doublet of doublets, m = multiplet), coupling constants (Hz), and integration. Mass spectrometry analysis was performed using via electrospray ionisation in methanol.

3.2 Preparation of phosphonium salts and 4-methoxy benzyl bromide

Compound S3: Ethyltriphenylphosphonium bromide

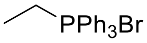

A 0.5 cm thick glass tube was charged with triphenylphosphine (4.50 g, 17.4 mmol), EtBr (2.7 mL, 36 mmol) and toluene (7 mL). The tube was sealed and heated to 110 ˚C for 17 h. The mixture was cooled to rt, the solids were filtered off and washed sequentially with toluene, hexane and ether (20 mL each) under a stream of nitrogen. The solids were dried under reduced pressure afforded product as fine white powder (6.0 g, 93%).

Compound S4: Benzyltriphenylphosphonium bromide

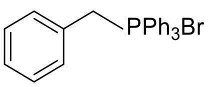

Following a literature procedure,[1] a stirred solution of PPh_3 (4.00 g, 15.3 mmol) and BnBr (1.5 mL, 13 mmol) in toluene (50 mL) were heated to 80 ˚C and stirred for 16 h. The mixture was allowed to reach rt and the white solid filtered off. The solids were washed with ether (60 mL) followed by hexane (60 mL) then dried under reduced pressure at 40 ˚C for 30 min afforded product as white crystalline solid (5.7 g, 100%). All spectra data was in accord with that reported.[1]

Compound S5: Phenylethyltriphenylphosphonium bromide

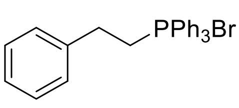

Following an adapted procedure,[2] a stirred solution of PPh_3 (4.7 g, 18 mmol) and 2-bromoethyl benzene (2.4 mL, 18 mmol) was refluxed in toluene (64 mL) for 4 days. The mixture was allowed to cool to rt and solvent decanted off. Hexane (50.0 mL) was added with stirring and cooled to 0 ˚C. The mixture was stirred for 15 mins before decanting hexane off and scratching at the gum with a spatula to induce mobility. This process was repeated once more. After decanting hexane, the gum-oil was dried under reduced pressure causing it to expand. The gum was scratched with a spatula vigorously and dried under reduced pressure at 45 ˚C. After 5 scratching/evaporation cycles, product was obtained as white crystalline powder (5.3 g, 66%). All spectra data was in accord with that reported.[2]

Compound S6: Isopentyltriphenylphosphonium bromide

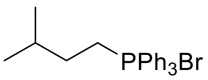

Following an adapted procedure,[3] to a stirred solution of PPh_3 (4.90 g, 18.7 mmol) in toluene (30 mL) at rt, in a flask fitted with a dry reflux condenser under nitrogen, 1-bromo-3-methyl butane (4.7 mL, 39 mmol) was added dropwise. The mixture was heated to 120 ˚C and stirred for 5 days. Alkyl halide (1 eq.) was added and the mixture stirred further for 18 h and cooled to 0 ˚C. The solid was filtered and washed with cold toluene (50 mL)

followed by cold Et_2O (4 × 30 mL). The solid was dried under reduced pressure to afford the title compound as a white powder (5.8 g, 75%). All spectra data was in accord with that reported.[3]

Compound S7: (Ethoxycarbonylmethylene)triphenylphosphorane.

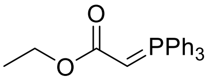

Following a literature procedure,[4] to a stirred solution of PPh_3 (2 g, 7.6 mmol) in EtOAc (16 mL), was added 2-bromo ethyl acetate (0.90 mL, 8.1 mmol) dropwise. The mixture was refluxed for 7.5 h and cooled to rt. The precipitate was filtered, washed with hexane (2 × 20 mL) and the solid dried under vacuum to give phosphonium salt as a fine white powder (3.2 g, 97%). The freshly made phosphonium salt (2.9 g, 6.9 mmol) was dissolved in DCM (44 mL) and washed with aq. KOH (1.3 g, 22.2 mmol in 44 mL). The phases were separated and the organic phase dried ($MgSO_4$) to give phosphorane **S7** as a white solid (2.7 g, quant.). All spectra data was in accord with that reported.[4]

Compound S8: 4-Methoxybenzyl bromide.

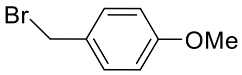

To stirred solution of 4-methoxy-benzyl alcohol (2.0 g, 15 mmol) in Et_2O (15 mL), cooled to 0 °C under nitrogen, was added PBr_3 (0.70 mL, 7.5 mmol) dropwise. The phases were separated, and organic phase washed further with saturated $NaHCO_3$ (2 × 15 mL) and brine (15 mL). The ether phase was dried ($MgSO_4$) and evaporated to give crude 4-methoxybenzyl bromide **S8** as a clear oil (2.0 g, 68%), which was used without further purification. All spectra data was in accord with that reported.[5]

3.3 Preparation of oxazolidone precursors

Compound (±)-S9: (2R, 5S)-2-trichloromethyl-1-aza-3-oxabicyclo[3.3.0]octan-4-one.

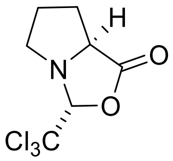

Following a literature procedure,[6] *rac*-proline (10.0 g, 86.9 mmol) and chloral hydrate (22 g, 131 mmol) were refluxed in $CHCl_3$ (160 mL) in a flask equipped with a reverse Dean-Stark condenser under argon for 6 h. The reaction was allowed to cool to rt and washed with saturated $NaHCO_3$ (120 mL) and the phases separated. The aq. phase was extracted further with $CHCl_3$ (30 mL). The combined organic phase was dried ($MgSO_4$) and evaporated to give crude brown crystalline solid. This was recrystallised from boiling EtOH (100 mL). The crystals were filtered off, washed with cold absolute EtOH, and dried under reduced pressure to give product (15.1 g, 73%) as a white crystalline solid. All spectral data was in accord with that reported.[6]

Compound (S)-S9: (2R, 5S)-2-trichloromethyl-1-aza-3-oxabicyclo[3.3.0]octan-4-one.

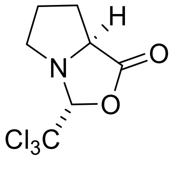

Following literature procedure,[6] L-proline (10.0 g, 86.9 mmol) and chloral hydrate (21.6 g, 131 mmol) were refluxed in $CHCl_3$ (160 mL) in a flask equipped with a reverse Dean-Stark condenser under argon for 6 h. The reaction was allowed to cool to rt and washed with saturated $NaHCO_3$ (120 mL) and the phases separated. The aq. phase was extracted further with $CHCl_3$ (30 mL). The combined organic phase was dried ($MgSO_4$) and evaporated to give crude brown crystalline solid. This was recrystallised from boiling EtOH (85 mL). The crystals were filtered off, washed with cold absolute EtOH, and dried under reduced pressure to give product (10.9 g, 51%) as a white crystalline solid. All spectral data was in accord with that reported.[6]

Compound (±)-S10: 4-oxo-2-(trichloromethyl)-1-aza-3-oxabicyclo[3.3.0]octane-5-carbaldehyde.

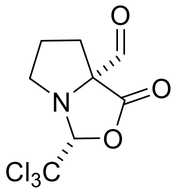

Following an adapted procedure,[7] to a stirred solution cooled to -78 ˚C, of dry $^{i}Pr_2NH$ (3.0 mL, 20 mmol) in dry THF (50 mL), $^{n}BuLi$ (8.5 mL of a 2.5M solution in hexane, 21 mmol) was added dropwise. The mixture was stirred for 30 min. A stirred solution of substrate **S9** (3.7 g, 14.9 mmol) in dry THF (24 mL) at 0 ˚C under argon, was then quickly added via syringe over 6 min to the freshly made LDA solution. The mixture was stirred for 30 min. Methyl formate (3.0 mL, 49 mmol) added dropwise and the mixture was stirred further at -78 ˚C for 25 min warmed to -40 ˚C and stirred for 25 min. The reaction was quenched with aq. 10% citric acid (50 mL) and extracted with DCM (180 mL). The phases were separated, and the aq. phase extracted further with DCM (110 mL). The combined organic phase was dried ($MgSO_4$) and evaporated. Purification by silica gel chromatography (Et_2O/petrol, 1:4 to 1:1 as eluent) afforded product as white solid (1.64 g, 48%). All spectra data was in accord with that reported.[6]

Compound (R)-S10: (2R, 5R)-4-oxo-2-(trichloromethyl)-1-aza-3-oxabicyclo[3.3.0]octane-5-carbaldehyde.

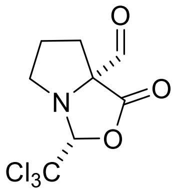

Following an adapted procedure,[7] to a stirred solution cooled to -78 ˚C, of dry $^{i}Pr_2NH$ (3.5 mL, 22.8 mmol) in dry THF (44 mL), $^{n}BuLi$ (9.3 mL of a 2.5M solution in hexane, 23 mmol) was added dropwise. The mixture was stirred for 30 min. A stirred solution of substrate **(S)-S9** (3.7 g, 14.9 mmol) in dry THF (30 mL) at 0 ˚C under argon, was then quickly added via syringe over 3 min to the freshly made LDA solution. The mixture was stirred for 30 min. Methyl formate (2.8 mL, 45 mmol) added dropwise over 30 sec. The mixture was stirred further at -78 ˚C for 25 min then warmed to -40 ˚C and stirred for 25 min. The reaction was quenched with aq. 10% citric acid (50 mL) and extracted with DCM (180 mL). The phases were separated, and the aq. phase extracted with DCM (110 mL). The combined organic phase was dried ($MgSO_4$) and evaporated. Purification by silica gel chromatography (Et_2O/petrol, 1:4 to 1:1 as eluent) afforded product as white solid (2.5 g, 74%). All spectra data was in accord with that reported.[6]

3.4 Preparation of vinyl oxazolidinone precursors

Compound (±)-S11: 2-Trichloromethyl-5-vinyl-1-aza-3-oxabicyclo[3.3.0]- octane-4-one.

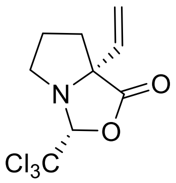

Following literature procedure,[6] to stirred suspension of $MePPh_3Br$ (1.3g, 3.6 mmol) in dry toluene (70 mL) under argon at rt, KO^{t}Bu (401 mg, 3.6 mmol) was added in one portion. The mixture was heated to 90 ˚C and stirred for 2 h until yellow solution had formed. The mixture was cooled to rt and a solution of aldehyde **(±)-S10** (852 mg, 3.1 mmol) in dry toluene (9.0 mL) added dropwise then stirred for 1 h. The mixture was cooled to 0 ˚C, diluted with ether (70 mL) and stirred vigorously for 5 min. The mixture was filtered over Celite, the solids washed with ether (50 mL) and the filtrate evaporated. Purification by silica gel chromatography (EtOAc/hexane, 5:95 to 1:9 as eluent) afforded the title compound as clear oil (466 mg, 55%). All spectral data was in accord with that reported.[6]

Compound (±)-S12: 2-Trichloromethyl-5-(1-(Z)-propenyl)-1-aza-3-oxabicyclo[3.3.0]- octane-4-one.

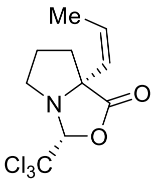

To a stirred suspension of $EtPPh_3Br$ (4.2 g, 11.3 mmol) in anhydrous THF (50 mL) at rt under argon was added sodium hydride (420 mg of a 60% dispersion in mineral oil, 10.5 mmol) in a single portion and the reaction heated to 55 ˚C. After 16 h the red/orange suspension was cooled to 0 °C and substrate (850 mg, 2.13 mmol) added portionwise until a tan suspension was formed. The reaction was warmed to rt, stirred for 17 min, cooled to 0 °C and petrol (120 mL) added. After stirring for 5 min the mixture was filtered through Celite, eluting with Et_2O/petrol (1:5, 50 mL). Evaporation gave a light yellow semi-

solid which was purified by silica gel chromatography (Et_2O/petrol, 1:19 to 1:9 as eluent) to afford the title compound (648 mg, 73%) as a pale yellow oil. ν_{max} /cm^{-1} (neat) 2950, 1801, 1448, 1365, 1321, 1217, 1208, 1186; δ_H ($CDCl_3$, 400 MHz) 1.78 (sextet, J = 7.0 Hz, 1H, NCH_2CH*H*), 1.86 (d, J = 6.8 Hz, Me), 1.92 (hept, J = 6.0 Hz. 1H, NCH_2CH*H*), 2.09 (quint, J = 7.0 Hz, 1H, NCH_2CH_2CH*H*), 2.32 (quint, J = 6.4 Hz, 1H, NCH_2CH_2CH*H*), 3.21 (quint, J = 5.8 Hz, 1H, NCH*H*CH_2), 3.45 (ddd, J = 11.5, 6.3, 6.1 Hz, 1H, NCH*H*CH_2), 5.05 (s, 1H, (Cq(CCl_3)), 5.50 - 5.59 (m, 1H, CH=C*H*Me), 5.62 (d, J = 13.2 Hz, 1H, C*H*=CHMe). δ_C ($CDCl_3$, 101 MHz) 13.8 (Me), 25.7 (NCH_2*C*H_2CH_2), 40.2 (NCH_2CH_2*C*H_2), 58.2 (N*C*$H_2CH_2CH_2$), 77.8 (*C*CH=$CHCH_3$), 100.5 (*C*Cl_3), 102.8 (*C*qCO_2), 127.7 (CH=*C*HCMe), 130.3 (*C*H=CHMe), 175.3 (Cq*C*O_2). m/z: molecular ion not found.

Compound (R)-S12: 2-Trichloromethyl-5-(1-(Z)-propenyl)-1-aza-3-oxabicyclo[3.3.0]- octane-4-one.

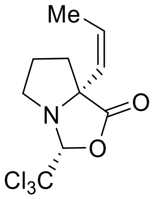

To stirred suspension of freshly made $EtPPh_3Br$ **S3** (1.7 g, 4.6 mmol) in dry THF (18 mL) at rt under argon, NaH (174 mg of 60% NaH in mineral oil, 4.4 mmol) was added portionwise. The mixture was heated to 60 °C and stirred for 3.5 h. The orange solution was cooled to 0 °C and aldehyde (R)-**S11** (547 mg, 2 mmol) was added portion wise until loss of colour occurred. The mixture warmed to rt, stirred for 10 min and recooled to 0 °C. Petrol (54 mL) was added and the mixture stirred vigorously for 10 min. The reaction was filtered over Celite, and solids washed with petrol/ether (5:1, 30 mL). The filtrate was evaporated to give a yellow oily solid. Purification by silica gel chromatography (Et_2O/petrol. 1:99 to 5:95 to 1:9 as eluent) afforded the product as a clear oil (390 mg, 69%).

Compound (±)-S13: 2-Trichloromethyl-5-(1-(Z)-(2-phenylethyl)vinyl)-1-aza-3-oxabicyclo[3.3.0]-octane-4-one.

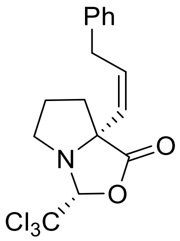

To stirred suspension phenylethyltriphenylphosphonium bromide **S5** (653.0 mg, 1.5 mmol) in dry THF (14 mL), cooled to -10 °C under argon, nBuLi (530.0 µL of 2.5M ^{n}BuLi, 1.3 mmol) was added dropwise. The mixture was stirred for 10 min by which time a dark red solution had formed. Aldehyde **S10** (200.0 mg, 0.7 mmol) was added portion wise until loss of colour occurred. The mixture was warmed to rt, stirred for 20 min then cooled to 0 °C. Petrol (45 mL) was added and stirred vigorously for 10 min. The mixture was filtered over celite, and solids rinsed with petrol/ether (5:1, 40 mL). The filtrate was evaporated to give crude orange oil. Purification by silica gel chromatography (Et_2O/petrol, 5:95 to 1:1 as eluent) afforded the product as a clear oil (154 mg 58%). ν_{max} /cm^{-1} (neat) 3027, 2957, 2807, 1718, 1495, 1453, 1166; δ_H ($CDCl_3$, 400 MHz) 1.80 (dq, *J* = 13.4, 7.3 Hz, 1H, NCH_2C*H_2*). 1.92 - 2.02 (m, 1H, NCH_2C*H_2*), 2.16 (ddd, *J* = 13.2, 8.0, 6.3 Hz, 1H NCH_2CH_2CH*H*), 2.41 (dt, *J* = 13.1, 6.6 Hz, 1H, NCH_2CH_2CH*H*), 3.22 (dt, *J* = 11.5, 5.9 Hz, 1H, NC*H_2*CH_2), 3.50 (ddd, *J* = 11.5, 8.0, 6.1 Hz, 1H, NC*H_2*CH_2), 3.71 - 3.86 (m, 2H, RCH=CHC*H_2*Ph), 5.01 (s, 1H, C*H*CCl_3), 5.65 (dt, *J* = 11.6, 7.3 Hz, 1H, RCH=C*H*CH_2Ph), 5.77 (dt, *J* = 11.5, 1.9 Hz, 1H, RC*H*=$CHCH_2$Ph), 7.17 - 7.32 (m, 5H, CH_{Ar}). δ_C ($CDCl_3$, 101 MHz) 25.7 (NCH_2*C*H_2), 34.0 (RCH=CH*C*H_2Ph), 40.6 (C_q*C*H_2CH_2), 58.5 (N*C*H_2CH_2), 72.9 (R_2N*C_q*CO), 100.5 (*C*Cl_3), 102.9 (*C*HCCl_3), 130.2 (R*C*H=$CHCH_2$Ph), 131.8 (RCH=*C*HCH_2Ph), 140.8 (Cq_{Ar}), 175.1 (*C*O_{ester}). HRMS (ESI^+) m/z: molecular ion not found.

Compound (±)-S14: 2-Trichloromethyl-5-(1-(Z)-(2-phenyl)vinyl)-1-aza-3-oxabicyclo[3.3.0]- octane-4-one.

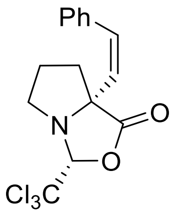

To stirred suspension of $PhCH_2PPh_3$ **S5** (2.1 g, 4.8 mmol) in dry THF (20 mL), cooled to 0 °C under nitrogen, NaH (172 mg of 60% NaH in mineral oil, 4.3 mmol) was added in one portion. The mixture was stirred at 0 °C for 1.5 h forming and orange solution. A solution of aldehyde **S10** (651 mg, 2.4 mmol) in dry THF (1 mL) was added dropwise. The mixture was stirred at 0 °C for 30 min, warmed to rt and stirred further for 30 min. The mixture was recooled to 0 °C, petrol (60 mL) was added, and the mixture stirred vigorously for 10 min. This was filtered over Celite, and the solids washed with petrol/ether (5:1, 50 mL). The filtrate was evaporated to give an orange solid. Purification by silica gel chromatography (Et_2O/petrol, 5:95 to 1:9 as eluent) afforded the product as a white crystalline solid (411 mg, 50%). Mpt 101-102 °C; ν_{max} /cm^{-1} (neat); δ_H ($CDCl_3$, 400 MHz) 1.45 – 1.61 (1H, m, NCH_2CH*H*), 1.63 – 1.76 (1H, m, NCH_2CH*H*)), 1.95 (1H, ddd, J = 13.2, 9.7, 6.6 Hz, $C_q(CO_2)CH$*H*), 2.17 (1H, ddd, J = 12.4, 7.5, 4.3 Hz, $C_q(CO_2)CH$*H*), 2.91 (1H, ddd, J = 12.0, 9.6, 5.7 Hz, NCH*H*), 3.02 (1H, ddd, J = 10.7, 6.7, 3.6 Hz, NCH*H*), 4.99 (1H, s, C*H*Cl_3), 5.99 (1H, d, J = 12.6 Hz, CH=C*H*Ph), 6.68 (1H, d, J = 12.6 Hz, C*H*=CHPh), 7.25 – 7.33 (3H, m, 3 x CH_{Ar}), 7.55 (2H, d, J = 7.4 Hz, 2 x CH_{Ar}); δ_C ($CDCl_3$, 101 MHz) 25.8 (NCH_2CH_2), 39.0 ($C_q(CO_2)CH_2$), 57.7 (N*C*H_2), 73.5 (N$C_q$$CO_2$), 100.5 (*C*$Cl_3$), 102.8 (*C*H$CCl_3$), 127.5 (*C*$H_{Ar}$), 127.9 (*C*$H_{Ar}$), 132.2 (CH=*C*HPh), 133.2 (*C*H=CHPh), 136.4 (*C*$_{qAr}$), 175.3 (*C*O_2). HRMS (ESI$^+$) m/z: molecular ion not found.

3.5 Preparation of proline-derived allylic amine substrates

Compound (±)-7a: N-benzyl-O-benzyl-pyrrolidine-(2-vinyl)-2-carboxylate.

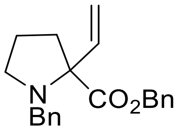

To stirred solution of **S11** (466 mg, 1.6 mmol) in 2-propanol (10 mL) at rt, aq. 6 M HCl (10 mL) was added, and mixture stirred for 6 days. The reaction was dried by azeotropic removal of toluene (6 x 20 mL) under reduced pressure at 70 °C to give pink-white crystalline solid. This was suspended in MeCN (7 mL) with stirring at rt. K_2CO_3 (680 mg, 4.9 mmol) was added followed by NaI (37 mg, 0.3 mmol) and BnBr (390 µL, 3.3 mmol). The mixture was heated to 80 °C and stirred for 12 h. The mixture was allowed to cool to rt and evaporated. The residue was taken up in water (10 mL) and extracted with Et_2O (3 x 10 mL). The combined organic phase was dried ($MgSO_4$) and evaporated. Purification by silica gel chromatography (Et_2O/petrol; 1:99 to 1:9 as eluent) afforded product as a clear oil (383 mg, 73% over two steps). ν_{max} /cm^{-1} (neat) 3029, 2957, 2831, 2807, 1721, 1495, 1454); δ_H ($CDCl_3$, 400 MHz) 1.76 – 1.86 (2H, m,NCH_2C*H*$_2$), 1.93 (ddd, *J* = 12.4, 9.9, 7.3 Hz, 1H, NCH_2CH_2CH*H*), 2.36 – 2.43 (m, 1H, NCH_2CH_2CH*H*), 2.68 (q, J = 8.2 Hz, NCH*H*), 2.90-2.97 (1H, NCH*H*), 3.60 (1H, d, J = 13.8 Hz, NCH*H*Ph), 3.92 (d, J = 13.8 Hz, NCH*H*Ph), 5.18 – 5.27 (m, 3H, CH=CH*H* and CO_2CH_2Bn), 5.39 (dd, J = 17.4, 1.4 Hz, 1H, CH=CH*H*), 6.15 (dd, J = 17.5, 10.7 Hz, 1H, C*H*=CH_2). 7.18 – 7.42 (m, 10H, 10 x CH_{Ar}). δ_C ($CDCl_3$, 101 MHz) 21.9 (NCH_2CH_2), 37.9 ($NCH_2CH_2CH_2$), 50.7 (N*C*H_2), 53.9 (N*C*H_2Bn), 66.4 (CO_2CH_2Bn), 73.0 (NC_qCO_2), 115.0 (CH=*C*H_2), 126.7 (*C*H_{Ar}), 128.2 – 128.8 (5 x *C*H_{Ar}), 136.2 (*C*q_{Ar}), 140.5 (*C*q_{Ar}), 173.9 (*C*O_2Bn). HRMS (ESI$^+$) m/z: $[M+H]^+$ Calcd for $C_{21}H_{23}NO_2$ 322.1807; found 322.1804.

Compound (±)-7b: N-benzyl-O-benzylpyrrolidine-2-(1-(Z)-propenyl)-2-carboxylate.

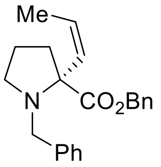

To stirred solution of (±)-**S12** (170 mg, 0.60 mmol) in 2-propanol (3 mL) was added aq. 6M HCl (3 mL) and mixture stirred for 4 days. The reaction was dried via azeotropic removal of toluene (3 x 8 mL) under reduced pressure at 60 °C. The white solid was suspended in MeCN (6 mL) with stirring at rt. K_2CO_3 (250 mg, 1.8 mmol) was added followed by NaI (15 mg, 0.10 mmol) and BnBr (0.15 mL, 1.25 mmol). The mixture was heated to 60 °C and stirred for 20 h. The mixture was cooled, diluted with water (20 mL) and extracted with ether (2 x 20 mL). The combined organic phase was dried ($MgSO_4$) and evaporated to give the crude product as yellow oil. Purification by silica gel chromatography (Et_2O/petrol, 1% to 5% as eluent) afforded the title compound (131 mg, 63%) as a clear oil. ν_{max}/cm^{-1} (neat) 2956, 2805, 1721, 1495, 1454, 1366 and 1169; δ_H ($CDCl_3$, 400 MHz) 1.57 (3H, dd, J 7.0, 1.3 Hz, Me), 1.75 – 1.93 (3H, m, NCH$_2$C*H*$_2$CH*H*), 2.46 (1H, td, J 8.6, 6.6 Hz, NCH*H*), 2.56 (1H, ddd, J 13.7, 9.4, 4.1 Hz, NCH$_2$CH$_2$C*H*H), 2.84 (1H, td, J 8.6, 3.8 Hz, NC*H*H), 3.33 (1H, d, J 13.4 Hz, NCH*H*Ph), 4.00 (1H, d, J = 13.6 Hz, NC*H*HPh), 5.14 – 5.25 (2H, AB q, OC*H*$_2$Ph), 5.63 (1H, dq, J = 11.4, 6.7 Hz, Me-C*H*=), 5.70 (1H, dd, J = 11.4, 1.6 Hz, C*H*=CH-Me) and 7.17 – 7.42 (10 H, m, CH_{Ar}); δ_C ($CDCl_3$, 101 MHz) 14.9 (Me), 21.9 (NCH$_2$*C*H$_2$), 36.8 (NCH$_2$CH$_2$*C*H$_2$), 49.8 (N*C*H$_2$CH$_2$), 54.1 (N*C*H$_2$Ph), 66.4 (O*C*H$_2$Ph), 71.0 (N*C*qCO$_2$), 126.8 (CH_{Ar}), 127.5 (=*C*H-Me), 128.25 (2 × CH_{Ar}), 128.33 (CH_{Ar}), 128.45 (2 × CH_{Ar}), 128.57 (2 × CH_{Ar}), 128.63 (2 × CH_{Ar}), 132.5 (*C*H=CH-Me), 136.1 (Cq_{Ar}), 140.4 (Cq_{Ar}) and 173.3 (*C*O$_2$Bn). HRMS (ESI$^+$) m/z: $[M+H]^+$ Calcd for $C_{22}H_{25}NO_2$ 336.1964; found 336.1962.

Compound (*R*)-7b: N-benzyl-O-benzylpyrrolidine-2-(1-(Z)-propenyl)-2-carboxylate

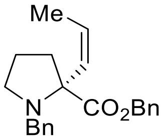

To stirred solution of (R)-**S12** (81.4 mg, 0.3 mmol) in 2-propanol (1.5 mL), aq. 6M HCl (1.5 mL) was added and mixture stirred for 6 days. The reaction was dried via azeotropic removal of toluene (4 x 4 mL) under reduced pressure at 60 °C. The white solid was suspended in MeCN (3 mL) with stirring at rt. K_2CO_3 (118.6 mg, 0.9 mmol) was added followed by NaI (7 mg, 0.05 mmol) and BnBr (68.0 µL, 0.6 mmol). The mixture was heated to 80 °C and stirred for 23 h. The mixture was cooled and evaporated. The residue was taken up in water (10 mL) and extracted with ether (2 x 10 mL). The combined organic phase was dried ($MgSO_4$) and evaporated to give crude product as yellow oil. Purification by silica gel chromatography (Et_2O/petrol, 0:1 to 1:9 as eluent) afforded the product as a clear oil (71.0 mg, 74% over two steps). $[\alpha]^{20}_D$ = +9.8 (c 0.924, DCM). Spectral data was as reported above.

Compound (±)-7c: N-(4-methoxy-benzyl)-O-(4-methoxy-benzyl)-pyrrolidine-2-(1-(Z)-phenylethyl vinyl)-2-carboxylate.

To a stirred solution of compound **S13** (229 mg, 0.6 mmol), in 2-propanol (3.0 mL) at rt, 6M HCl aq. (3.0 mL) was added. The mixture was heated to 50 °C and stirred for 2 days then allowed to cool to rt and stir further for 5 days. The reaction was dried by the azeotropic removal of toluene (4 x 8.0 mL) under reduced pressure at 60 °C. The resulting oily residue was suspended in MeCN (5 mL) with stirring at rt. K_2CO_3 (261.0 mg, 1.9 mmol) was added in one portion, followed by NaI (14.0 mg, 0.01 mmol) and PMBBr (182 µL, 1.3 mmol). The mixture was heated to 80 °C, stirred for 2 h, allowed to cool to rt and evaporated. The orange residue was washed with water (10 mL) and extracted with Et_2O (3 x 10 mL). The combined organic phase was dried ($MgSO_4$) and evaporated to give brown oil (355.0 mg). Purification by silica gel chromatography (Et_2O/petrol, 1:9 to 1:4 as eluent) afforded product as a clear oil (192 mg, 65% over two steps). ν_{max}/cm^{-1} (neat) 2956, 2834, 1718, 1612, 1512, 1454, 1245; δ_H ($CDCl_3$, 400 MHz) 1.75 - 2.03 (3H, m , NCH$_2$C*H*$_2$ and NCH$_2$CH$_2$CH*H*), 2.45 (1H, q, *J* = 8.2 Hz, NCH*H*), 2.61 (1H, ddd, *J* = 11.9, 8.0, 4.0 Hz, NCH$_2$CH$_2$CH*H*), 2.86 (1H, td, J = 9.1, 3.2 Hz, NCH*H*), 3.26 (1H, d, J = 13.3 Hz, NCH*H*Ph), 3.34 (2H, d, J = 7.4 Hz, CH=CHC*H*$_2$Ph), 3.78 and 3.79 (6H, 2 x s, *J* = 4.3 Hz, 2 x OMe), 3.99 (1H, d, J = 13.2 Hz, NCH*H*Ph), 5.11 (2H, s, CO$_2$C*H*$_2$Ph), 5.65 – 5.74 (1H, m, CH=*CH*CH$_2$), 5.84 (1H, d, J = 11.6 Hz, C*H*=CH), 6.79 – 6.88 (4H, m, 4 x C*H*$_{Ar}$),

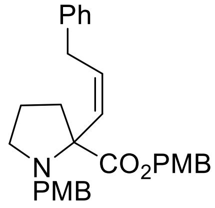

7.10 – 7.21 (5H, m, 5 x CH_{Ar}), 7.23 – 7.33 (4H, m, 4 x CH_{Ar}). δ_C ($CDCl_3$, 101 MHz) 22.0 (NCH_2CH_2), 35.3 (CH=CHCH_2), 37.2 ($NCH_2CH_2CH_2$), 49.8 (NCH_2), 53.6 (NCH_2Ph), 55.4 (OMe), 66.3 (CO_2CH_2Ph), 71.1, $NCqCO_2$), 113.7 (CH_{Ar}), 114.0 (CH_{Ar}), 128.2 (Cq_{Ar}), 128.4 and 128.5 (2 x CH_{Ar}), 129.6 (CH_{Ar}), 130.6 (CH_{Ar}), 131.3 (CH=CHCH_2) 132.3 (Cq_{Ar}), 132.6 (CH=CHCH_2), 140.4 (Cq_{Ar}), 158.6 (Cq_{Ar}), 159.7 (Cq_{Ar}), 173.1 (CO_2Bn). HRMS (ESI$^+$) m/z: $[M+H]^+$ Calcd for $C_{30}H_{33}NO_4$ 472.2488; found 472.2490.

Compound (±)-7d: N-(4-methoxy-benzyl)-O-(4-methoxy-benzyl)-pyrrolidine-2-(1-(Z)-phenylvinyl)-2-carboxylate.

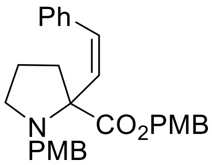

To stirred suspension at rt, of **S14** (572 mg, 1.7 mmol) in 2-propanol (10 mL), aq. 6 M HCl (10 mL) was added, and the oily suspension stirred at rt for 2 days. The mixture was heated to 50 ˚C and stirred further for 16 h by which time a clear solution had formed. The mixture was allowed to cool rt and dried via azeotropic removal of toluene (6 x 20 mL) under reduced pressure at 60 ˚C to give pink-white crystalline solid. This was suspended in MeCN (15 mL) with stirring at rt. K_2CO_3 (684 mg, 4.9 mmol), NaI (37 mg, 0.3 mmol) and PMBBr (476 µL, 3.3 mmol) were added sequentially. The mixture was heated to 80 ˚C and stirred for 15 h, allowed to cool to rt and evaporated. The brown-orange solid was taken up in water (10 mL) and extracted with Et_2O (3 x 10 mL). The combined organic phase was dried ($MgSO_4$) and evaporated to give crude product as brown-orange oil. Purification by silica gel chromatography (Et_2O/petrol, 1:99 to 3:7 as eluent) afforded the product as a clear oil (537 mg, 67% over two steps). ν_{max} /cm^{-1} (neat) 2954, 2843, 1716, 1612, 1511, 1462, 1365; δ_H ($CDCl_3$, 400 MHz) 1.69 - 1.99 (m, 3H, NCH_2CH_2 and NCH_2CH_2CH*H*), 2.33 (q, J = 8.2 Hz, 1H, NCH_2CH*H*), 2.57 (t, J = 9.6 Hz, 1H, NCH*H*), 2.82 (t, J = 8.4 Hz, 1H, NCH*H*), 3.12 (d, J = 13.2 Hz, 1H NCH*H*Ph), 3.12 (d, J = 13.2 Hz, 1H NCH*H*Ph), 3.78 (s, 6H, OMe), 3.99 (d, J = 13.2 Hz, 1H NCH*H*Ph), 4.43 (d, J = 12.1 Hz, CO_2CH*H*Ph), 4.76 (d, J = 12.1 Hz, CO_2CH*H*Ph), 5.90 (d, J = 12.5 Hz, 1H, CH=C*H*Ph), 5.62 (d, J = 12.5 Hz, 1H, C*H*=CHPh), 6.80 (d, J = 8.7 Hz, 4H, 4 x CH_{Ar}), 7.07 (m, 9H, 9 x CH_{Ar}). δ_C ($CDCl_3$, 101 MHz) 22.3 (NCH_2CH_2), 36.8 ($NCH_2CH_2CH_2$), 49.9 (NCH_2), 53.9 (NCH_2Ph), 55.4 (Ar-OMe), 66.0 (CO_2CH_2Ph), 71.1 (N*C*qCO_2), 113.6 and 113.8 (2 x CH_{Ar}), 127.0 (CH_{Ar}), 128.0 (CH_{Ar}), 128.6 (CH_{Ar}), 129.5 (CH_{Ar}), 130.2 (CH_{Ar}), 131.3 (*C*H=CHPh), 132.2 (Cq_{Ar}), 134.6 (CH=*C*HPh), 137.5 (Cq_{Ar}), 158.6 (Cq_{Ar}), 159.5 (Cq_{Ar}), 171.3 (CO_2Bn). HRMS (ESI$^+$) m/z: $[M+H]^+$ Calcd for $C_{29}H_{31}NO_4$ 458.233134; found 458.2334.

Compound (±)-7e: N-(4-methoxybenzyl-O-(4-methoxybenzyl)-pyrrolidine-2-(1-(Z)-propenyl)-2-carboxylate.

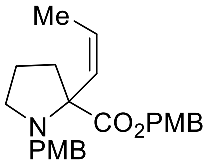

To a stirred solution of substrate **S12** (310 mg, 1.09 mol) in 2-propanol (6 mL) was added 6 M aq. HCl (6 mL) and the reaction stirred at rt. After 4 days the reaction was evaporated under vacuum at 45 °C and further dried by azeotropic removal of toluene (3 × 15 mL) at the same temperature to give a white solid. The crude material was suspended in MeCN (8 mL), potassium carbonate (250 mg, 1.8 mmol) added, stirred for 5 min and TBAI (50 mg, 0.14 mmol) and a solution of 4-methoxybenzyl bromide (475 mg, 2.4 mmol) in MeCN (3 mL) added. The reaction was heated to 60 °C. After 20 h the reaction was cooled to rt, evaporated and partitioned between water (20 mL) and Et_2O (20 mL). The aqueous phase was extracted with Et_2O (2 × 20 mL) and the combined organic phase dried ($MgSO_4$) and evaporated to give a yellow oil. Purification by silica gel chromatography (Et_2O/petrol, 10% to 20% as eluent) afforded the title compound (287 mg, 67% over two steps) as a clear oil. ν_{max} /cm^{-1} (film) 1301, 1462, 1511, 1612, 1718 and 2954; δ_H ($CDCl_3$, 400 MHz) 1.55 (3H, dd, *J* 6.9, 1.4 Hz, Me), 1.70 - 1.91 (3H, m, NCH_2CH_2CH*H*), 2.42 (1H, td, *J* 8.7, 6.9 Hz, NCH*H*CH_2), 2.52 (1H, ddd, *J* 13.7, 9.3, 4.0 Hz, NCH_2CH_2C*H*H), 2.81 (1H, td, *J* 8.6, 3.6 Hz, NC*H*HCH_2), 3.25 (1H, d, *J* 13.2 Hz, NCH*H*Ar), 3.77 (3H,

s, OMe), 3.80 (3H, s, OMe), 3.90 (1H, d, *J* 13.2 Hz, NC*H*HAr), 5.13 (2H, AB q, OCH_2Ar), 5.55 – 5.70 (2H, m, *H*C=C*H*), 6.78 – 6.83 (2H, m, 2 × CH_{Ar}), 6.84 – 6.90 (2H, m, 2 × CH_{Ar}), 7.11 – 7.19 (2H, m, 2 × CH_{Ar}) and 7.28 – 7.35 (2H, m, 2 × CH_{Ar}); δ_C ($CDCl_3$, 101 MHz) 14.9 (Me), 21.9 (NCH_2CH_2), 36.8 ($NCH_2CH_2CH_2$), 49.8 (NCH_2CH_2), 53.4 (NCH_2Ar), 55.3 (OMe), 55.4 (OMe), 66.1 (OCH_2Ar), 71.0 (C_q-CO_2), 113.6 (2 × CH_{Ar}), 113.9 (2 × CH_{Ar}), 127.3 (=*C*HMe), 128.4 (C_q), 129.6 (2 × CH_{Ar}), 130.4 (2 × CH_{Ar}), 132.5 (C_q), 132.6 (*C*H=CHMe), 158.5 (C_qOMe), 159.7 (C_qOMe) and 173.3 (CO_2Ar); HRMS (ESI^+) m/z: $[M+H]^+$ Calcd for $C_{24}H_{29}NO_4$ 396.2175; found 396.2174

Compound (±)-7f: N-(4-methoxy-benzyl)-N-(amido-4-methoxy-benzyl)-pyrrolidine-2-(1-(Z)-propenyl)-2-carboxylate amide.

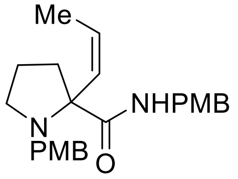

A stirred mixture of neat compound **S12** (110 mg, 0.4 mmol) and $PMBNH_2$ (101 µL, 0.8 mmol) was heated to 80 ˚C for 6 h. The mixture was allowed to cool to rt then passed through a silica plug (neat EtOAc as eluent) to remove excess $PMBNH_2$ to give a mixture of free amine/ formamide as an orange oil (92 mg). This was dissolved in EtOH (1 mL) with stirring at rt. 50% aq. NaOH (0.3 mL) was added, and the mixture heated to 80 ˚C for 4 h then allowed to cool to rt and stir for 2.5 h. The reaction was dissolved in water (10 mL) and extracted with DCM (3 x 10 mL). The combined organic phase was dried ($MgSO_4$) and evaporated to give crude free amine (75 mg, ca 0.3 mmol). This was dissolved in MeCN (1 mL) with stirring at rt. K_2CO_3 (37 mg, 0.3 mmol), NaI (3 mg, 0.02 mmol) and PMBBr (39 µL, 0.3 mmol) were added sequentially. The mixture was stirred for 6.3 h then evaporated. The brown residue was washed with water (10 mL) and extracted with ether (3 x 10 mL). The combined organic phase was dried ($MgSO_4$) and evaporated to give a yellow oil. Purification by silica gel chromatography (EtOAc/petrol, 1:9 to 1:1) afforded the product as a clear oil (75.0 mg, 49% over 3 steps). ν_{max} /cm^{-1} (neat) 3337, 2934, 2834, 1660, 1611, 1599, 1301, 1241. δ_H ($CDCl_3$, 400 MHz) δ 1.65 (3H, d, J = 7.2 Hz, *Me*). 1.69 - 1.84 (2H, m, NCH_2CH_2), 2.09 (1H, dt, J = 13.1, 8.4 Hz, NCH_2CH_2CHH), 2.40 (1H, ddd, J = 13.3, 8.7, 4.6 Hz, NCH_2CH_2CHH), 2.54 (1H, q, J = 8.2 Hz, NCH*H*), 2.83 (1H, td, J = 8.4, 3.8 Hz, NCH*H*), 3.37 (1H, d, J = 13.0 Hz, NCH*H*Ph), 3.63 (1H, d, J = 13.0 Hz, NCH*H*Ph), 3.76 (3H, s, ArO*Me*), 3.76 (3H, s, ArO*Me*), 4.31 (1H, dd, J = 14.2, 4.9 Hz, CONHCH*H*), 4.47 (1H, dd, J = 14.2, 6.3 Hz, CONHCH*H*), 5.50 (1H, d, J = 11.6 Hz, C*H*=CHMe), 5.79 (1H, dt, J = 14.3, 7.1 Hz, CH=C*H*Me), 6.74 (2H, d, J = 8.3 Hz, 2 x CH_{Ar}), 6.86 (2H, d, J = 8.2 Hz, 2 x CH_{Ar}), 7.00 (2H, d, J = 8.0 Hz, 2 x CH_{Ar}), 7.21 (2H, d, J = 8.1 Hz, 2 x CH_{Ar}), 7.83 (1H, t, J = 5.9 Hz, CON*H*CH_2). δ_C ($CDCl_3$, 101 MHz) 14.8 (*Me*), 22.9 (NCH_2CH_2), 39.7 ($NCH_2CH_2CH_2$), 43.2 ($CONHCH_2$), 51.9 (NCH_2), 54.5 (NCH_2Ph), 55.3 and 55.4 (2 x ArO*Me*), 113.8 (CH_{Ar}), 114.2 (CH_{Ar}), 129.3 (CH_{Ar}), 129.5 (CH_{Ar}), 129.9 (CH=*C*HMe), 130.2 (*C*H=CHMe), 130.7 (Cq_{Ar}), 132.0 (Cq_{Ar}), 158.6 (Cq_{Ar}), 159.1 (Cq_{Ar}), 174.8 (Cq_{Ar}). HRMS (ESI^+) m/z: $[M+H]^+$ Calcd for $C_{24}H_{30}N_2O_3$ 395.2335; found 395.2334.

Compound (±)-S15: O-(methyl-ester)-1H-pyrrolidine-2-(1-(*Z*)-propenyl)-2-carboxylate.

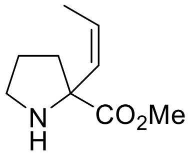

To a stirred solution of compound **S12** (886 mg, 3.1 mmol) in MeOH (13 mL) at rt under argon, Na (64.0 mg, 2.8 g atom) was added. The mixture was stirred for 2 h. The mixture was cooled to 0 ˚C. Acetyl chloride (5.8 mL, 81.6 mmol) was added dropwise. The mixture was heated to 50 ˚C and stirred for 4.5 h then allowed to cool to rt. The reaction was stirred for 15 h then heated to 50 ˚C, stirred for 3 h and evaporated under reduced pressure. The light brown residue was taken up in DCM (10 mL) and washed with saturated $NaHCO_3$ (10 mL). The phases were separated and the aq. phase extracted further with DCM (2 x 10.0 mL). The combined organic phase was dried ($MgSO_4$) and evaporated to give the crude free base amine (525.0 mg, ca. 3 mmol) which was used without further purification.

Compound (±)-7l: N-(5-methyl-2-furyl)-O-(methyl-ester)-pyrrolidine-2-(1-(Z)-propenyl)-2-carboxylate ester.

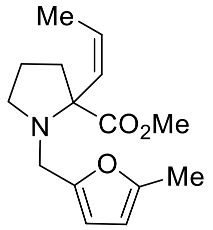

To stirred solution of crude amine **S15** (80 mg, 0.47 mmol) in DCM (1.5 mL) at rt, 5-methylfurfural (52 µL, 0.52 mmol) was added and the mixture stirred for 10 min. $NaBH(OAc)_3$ (148 mg, 0.7 mmol) was added in one portion. The mixture was stirred for 17.5 h, saturated $NaHCO_3$ (10 mL) was added, and the mixture stirred for 5 mins. The reaction was extracted with DCM (3 x 10 mL), the combined organic phase was dried ($MgSO_4$) and evaporated to give a red oil. Purification by silica gel chromatography (Et_2O/petrol, 5:95 to 1:9 as eluent) afforded product as a clear oil (47 mg, 38% over three steps). ν_{max} /cm^{-1} (neat) 2949, 2816, 1722, 1567, 1434, 1366. δ_H ($CDCl_3$, 400 MHz) 1.59 (3H, d, J = 5.9 Hz, CH=CH*Me*), 1.79 – 1.97 (3H, m, NCH_2CH_2 and NCH_2CH_2CHH), 2.25 (3H, s, Ar*Me*) 2.49 (1H, ddd, 12.2, 8.2 4.6 Hz, NCH_2CH_2CHH), 2.58 (1H, q, J = 8.3 Hz, NCH*H*), 2.99 (1H, td, J = 8.5, 4.6 Hz, NCH*H*), 3.44 (1H, d, J = 14.1 Hz, NCH*H*Ar), 3.72 (3H, s, CO_2*Me*), 3.84 (1H, d, J = 14.1 Hz, NCH*H*Ar), 5.58 – 5.69 (2H, m, C*H*=C*H*Me), 5.84 (1H, d, J = 3.7 Hz, CH_{Ar}), 6.01 (1H, d, J = 3.0 Hz, CH_{Ar}). δ_C ($CDCl_3$, 101 MHz) 13.8 (Ar*Me*), 14.6 (*Me*), 21.6 (NCH_2CH_2), 36.9 ($NCH_2CH_2CH_2$), 46.7 (NCH_2Ar), 50.3 (CO_2*Me*), 51.6 (NCH_2), 70.8 (NCqCO_2), 106.0 (CH_{Ar}), 108.3 (CH_{Ar}), 127.8 (*C*H=CHMe), 131.5 (CH=*C*HMe), 151.5 (Cq_{Ar}), 173.7 (*C*O_2Me). HRMS (ESI$^+$) m/z: $[M+H]^+$ Calcd for $C_{15}H_{21}NO_3$ 264.1600; found 264.1596.

Compound (±)-7m: N-(2-pyridyl)-O-(methyl-ester)-pyrrolidine-2-(1-(Z)-propenyl)-2-carboxylate.

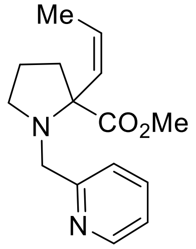

To a stirred solution of crude amine **S15** (159 mg, < 0.60 mmol), in MeCN (5 mL) at rt, K_2CO_3 (155 mg, 1.1 mmol), NaI (6.0 mg, 0.04 mmol) and 2-bromomethyl pyridine (96 mg, 0.6 mmol) were added sequentially. The mixture was stirred for 18 h then evaporated. The resulting red residue was dissolved in water (10 mL) and extracted with ether (3 x 10 mL). The combined organic phase was dried ($MgSO_4$) and evaporated to give a red oil. Purification by silica gel chromatography ($EtOH/NH_3$/DCM, 2:98 to 5:95 as eluent) afforded the product as a red oil (64 mg, 44% over 3 steps). ν_{max} /cm^{-1} (neat) 2950, 2878, 1723, 1652, 1589, 1433, 1463; δ_H ($CDCl_3$, 400 MHz) 1.58 (3H, d, J = 4.7 Hz, *Me*), 1.78 – 1.93 (3H, m, NCH_2CH_2 and NCH_2CH_2CHH), 2.48- 2.57 (2H,m, NCH*H* and NCH_2CH_2CHH), 3.61 (1H, d, J = 14.5 Hz, NCH*H*Pyr), 3.71 (3H, s, CO_2*Me*), 4.10 (1H, d, J = 14.5 Hz, NCH*H*Pyr), 5.60 – 5.67 (2H, m C*H*=C*H*Me), 7.10 (1H, t, J = 6.2 Hz, CH_{Ar}), 7.42 (1H, d, J = 7.8 Hz, CH_{Ar}), 7.61 (1H, t, J = 7.7 Hz, CH_{Ar}), 8.48 (1H, d, J = 4.7 Hz, CH_{Ar}). δ_C ($CDCl_3$, 101 MHz) 14.6 (Me), 22.0 (NCH_2CH_2), 36.7 ($NCH_2CH_2CH_2$), 50.1 (NCH_2), 51.7 (CO_2*Me*), 56.2 (NCH_2Pyr), 71.0 (NCqCO_2), 121.9 (CH_{Ar}), 122.9 (CH_{Ar}), 127.5 (CH=*C*HMe), 132.2 (*C*H=CHMe), 136.7 (CH_{Ar}), 149.0 (CH_{Ar}), 160.5 (Cq_{Ar}), 173.9 (*C*O_2Me). HRMS (ESI$^+$) m/z: $[M+H]^+$ Calcd for $C_{21}H_{20}N_2O_2$ 261.160303; found 261.1599.

Compound (±)-7n: N-(2-bromobenzyl)-O-(methyl-ester)-pyrrolidine-2-vinyl-2-carboxylate ester.

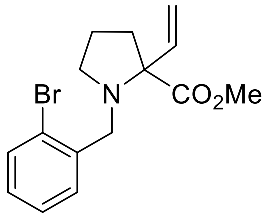

To a stirred solution of compound **S11** (180 mg, 0.7 mmol) in MeOH (5 mL) at rt under N_2, Na (25.0 mg, 1.1 g atom) was added. The mixture was stirred for 1 h. The mixture was cooled to 0 °C, acetyl chloride (1.3 mL, 18.2 mmol) was added dropwise. The mixture was heated to 50 °C, stirred for 17 h, allowed to cool to rt and evaporated. The resulting residue was suspended in MeCN (7 mL) at rt with stirring under N_2. K_2CO_3 (231 mg, 1.7 mmol), NaI (7 mg, 0.05 mmol) and 2-bromobenzyl bromide (185 mg, 0.7 mmol) were added sequentially, heated to 80 °C and stirred for 7 h. The mixture was allowed to cool to rt and evaporated. The residue was dissolved in water (20 mL) and extracted with DCM (2 x 20 mL). The combined organic phase was dried ($MgSO_4$) and evaporated to give crude orange oil. Purification by silica gel chromatography (Et_2O/petrol, 1:99 to 3:97 to 5:95 as eluent) afforded product as a clear oil (160 mg, 74% over 3 steps). ν_{max} /cm^{-1} (neat) 3061, 2836, 1726, 1462, 1439, 1169, 1024; δ_H ($CDCl_3$, 400 MHz)

1.89 – 2.00 (3H, m, NCH_2CH_2 and C_qCHH), 2.39 (1H, dt, J = 12.2, 6.5 Hz, C_qCHH), 2.74 (1H, q, J = 8.0 Hz, NCH*H*), 3.03 (1H, dt, J = 8.4, 6.4 Hz, NCH*H*), 3.77 (3H, s, CO_2Me), 3.89 (2H, s, NCH_2Ph), 5.18 (1H, d, J = 10.7 Hz, CH=CH*H*), 5.28 (1H, d, J = 17.4 Hz, CH=CH*H*), 6.09 (1H, dd, J = 17.4, 10.7 Hz, C*H*=CH_2), 7.07 (1H, t, J = 7.6 Hz, CH_{Ar}), 7.28 (1H, t, J = 7.8 Hz, CH_{Ar}), 7.50 (1H, d, J = 8.0 Hz, CH_{Ar}), 7.57 (1H, d, J = 7.6 Hz, CH_{Ar}); δ_C ($CDCl_3$, 101 MHz) 21.9 (NCH_2CH_2), 37.7 (C_qCH_2), 50.9 (NCH_2), 51.7 (CO_2Me), 53.5 (NCH_2Ph), 73.5 (C_qCO_2), 115.3 (CH=CH_2), 123.7 (C_{qAr}) 127.4 (CH_{Ar}), 128.0 (CH_{Ar}), 129.7 (CH_{Ar}), 132.6 (CH_{Ar}), 138.6 (*C*H=CH_2), 139.3 (C_{qAr}), 174.5 (CO_2Me); HRMS (ESI^+) m/z: $[M+H]^+$ Calcd for C15H19NO2Br 324.0599; found 324.0586.

3.6 Preparation of 2-phenylpyrrolidine allylic amine substrates

Compound (±)-S16: N-Boc-2-phenyl-pyrrolidine.

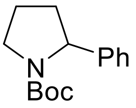

To a stirred solution of 2-phenylpyrrolidine (1.5 g, 10.2 mmol) in DCM (50 mL) under nitrogen was added Et_3N (2.8 mL, 20 mmol) and Boc_2O (4.4 g, 20 mmol) and the reaction stirred at rt. After 3.5 h the mixture was evaporated and purified by silica gel chromatography (two sequential columns employing Et_2O/petrol, 1:9 to 3:7 then Et_2O in DCM, 0% to 1% as eluent) to afford the title compound (2.10 g, 83%) as a clear oil which subsequently crystallised on standing. All data was in accord with that reported.[8]

Compound (±)-S17: 1-(Boc) 2-ethyl 2-phenylpyrrolidine-2-carboxylate.

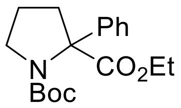

Following an adapted literature procedure,[9] to a dried flask under argon was added compound **S16** (500 mg, 2 mmol) and dry THF (24 mL) and the stirred solution degassed by sparging with argon for 4 min. The solution was cooled to 0 °C under argon and ^nBuLi (1.05 mL of a 2.5 M solution, 2.6 mmol) added dropwise over 3.5 min. The reaction was stirred for a further 3.5 min and ethyl chloroformate (410 µL, 4.3 mmol) added dropwise over 1 min. The reaction was stirred for 10 min, warmed to rt over 5 min and quenched by the addition of sat. aq. NH_4Cl (50 mL) and Et_2O (100 mL). The phases were separated, the aqueous phase extracted with Et_2O (50 mL) and the combined organic phase dried ($MgSO_4$) and evaporated to give a yellow oil. Purification by silica gel chromatography (EtOAc/petrol, 1:9 to 1:4 as eluent) afforded the title compound (574 mg, 89%) as a clear oil which partially crystallised on standing. Spectral data was in accord with that reported.[10]

Compound (±)-S18: 1-(benzyl) 2-ethyl 2-phenylpyrrolidine-2-carboxylate.

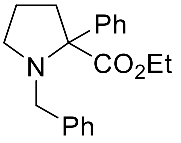

To stirred MeOH (7 mL) at 0 °C under argon was added acetyl chloride (1.0 mL) dropwise. The reaction was stirred for 10 min and a solution of compound **S17** (570 mg, 1.8 mmol) in MeOH (2 mL) added dropwise. The reaction was warmed to rt, stirred for 23 h and evaporated to give a yellow oil. The crude hydrochloride was redissolved in MeCN (15 mL) and potassium carbonate (650 mg, 4.7 mmol), sodium iodide (30 mg, 0.20 mmol) and benzyl bromide (275 µL, 2.3 mmol) added sequentially. The reaction was stirred at rt for 22 h, filtered and evaporated. Purification by silica gel chromatography (Et_2O/petrol, 5:95 to 1:9 as eluent) afforded the title compound (434 mg, 78%) as a clear oil. ν_{max} /cm^{-1} (neat) 2978, 1745, 1699, 1447, 1388 and 1246; δ_H ($CDCl_3$, 400 MHz) 1.33 (3H, t, J 7.2, Me), 1.82 – 1.97 (3H, m, NCH_2CH_2 and C_q-CH*H*), 2.54 (dt, J 9.4, 8.8, Cq-C*H*H), 2.66 – 2.74 (1H, m, NCH*H*CH_2), 3.13 (ddd, J 9.3, 8.8, 3.3, $NCHHCH_2$), 3.46 (1H, d, J 14.2, NCH*H*Ph), 3.93 (1H, d, J 14.1, NC*H*HPh), 4.31 (2H, q, J 7.1, CH_2Me), 7.21 – 7.40 (8H, m) and 7.45 (2H, d, J 7.7); δ_C ($CDCl_3$, 100 MHz); 14.7 (CH_2Me), 21.8 (NCH_2CH_2), 40.1 (C_qPhCH_2), 50.3 (NCH_2), 54.2 (NCH_2Ph), 60.7 (CO_2CH_2), 75.8 (NC_qCO_2), 126.7 and 126.8 (2 x CH_{Ar}), 127.3 (CH_{Ar}), 128.2 – 128.4 (3 x CH_{Ar}), 140.4 (C_{qAr}), 142.0 (C_{qAr}); HRMS (ESI^+) m/z: $[M+H]^+$ calcd for $C_{20}H_{23}NO_2$ 310.1808; found 310.1805.

Compound (±)-7g: 1-(benzyl) 2-vinyl 2-phenylpyrrolidine**.**

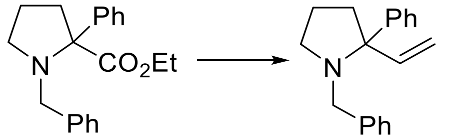

To a stirred solution of compound **S18** (542 mg, 1.8 mmol) in dry THF (17.5 mL) at 0 °C under argon was added $LiAlH_4$ (166 mg, 4.4 mmol) in two portions. The mixture was stirred for 5 min and heated to 60 °C. After 1 h the reaction was cooled to 0

°C and was quenched by the sequential dropwise addition of water (170 µL), 15% aq. NaOH (170 µL), and water (540 µL),). The mixture was stirred at rt for 10 min, diluted with Et_2O (10 mL) and $MgSO_4$ added. After stirring for a further 10 min the mixture was filtered, washing with EtOAc (10 mL) and DCM (10 mL). Evaporation gave the desired amino alcohol as a pale-yellow oil (453 mg, <1.7 mmol). SO_3.Pyr complex (504 mg, 3.2 mmol) was dissolved in dry DMSO (2.8 mL) under argon and stirred for 15 min at rt. The resulting solution was added dropwise to a stirred solution of amino-alcohol (<0.1.7 mmol) and triethylamine (1.2 mL, 8.4 mmol) in dry DMSO (5.8 mL) at rt under argon. The reaction was stirred for 1h and quenched by the addition of saturated $NaHCO_3$ (80 mL). The mixture was extracted with Et_2O (2 × 60 mLl) and the combined organic phase washed with saturated $NaHCO_3$ (40 mL). Drying ($MgSO_4$) and evaporation gave the crude aldehyde (370 mg, <1.7 mmol) as a yellow oil used without further purification. Ca. 30% of this crude aldehyde (113 mg, < 0.4 mmol) was used in the subsequent step. To a suspension of $MePPh_3Br$ (660 mg, 1.9 mmol) in toluene (10 mL) was added KO^tBu (208 mg, 1.9 mmol) and the stirred mixture heated to 70 °C under argon. After 2 h the reaction was cooled to 0 °C and a solution of the crude aldehyde (370 mg, >0.4 mmol) in toluene (1 mL) added dropwise. The reaction was stirred for 10 min, warmed to rt, stirred for a further 10 min and the excess ylide quenched by the dropwise addition of MeOH (60 µL),) then cooled to 0 °C. The mixture was diluted with Et_2O (10 mL) and petrol (10 mL) and stirred for 5 min. The mixture was filtered over Celite, the solids washed with petrol/ether (5:1, 20 mL) and the filtrate evaporated to give crude light yellow oil. Purification by silica gel chromatography (Et_2O/petrol, 1:9 as eluent) afforded product as a clear oil (58 mg, 51% over three steps) as a clear oil. ν_{max} /cm^{-1} (film) 2956, 1603, 1495, 1442; δ_H ($CDCl_3$, 400 MHz) 1.71 – 1.96 (3H, m, NCH_2CH_2 and C_qCHH), 2.24 – 2.33 (1H, m, C_qCHH), 2.42 (1H, dd, J 8.4, 7.9, $NCHHCH_2$), 3.02 (1H, td, J 8.7, 3.4, $NCHHCH_2$), 3.26 (1H, d, J 13.5, NCHHPh), 3.76 (1H, d, J 13.4, NC*H*HPh), 5.27 (1H, d, J 17.6, CH=CH*H*), 5.49 (1H, d, J 10.8, CH=C*H*H), 6.07 (1H, dd, J 17.6, 10.9, C*H*=CH_2), 7.21 – 7.27 (2H, m), 7.30 – 7.43 (6H, m) and 7.62 (2H, d, 7.7); δ_C ($CDCl_3$, 100 MHz) 21.8 (NCH_2CH_2), 38.6 (C_qCH_2), 49.9 (NCH_2CH_2), 53.3 (NCH_2Ph), 71.1 (NC_q), 116.0 (CH=CH_2), 126.7 (CH_{Ar}), 127.1 (CH_{Ar}), 128.1 (2 × CH_{Ar}), 128.2 (2 × CH_{Ar}), 128.3 (2 × CH_{Ar}), 137.8 (2 × CH_{Ar}), 140.7 (C_q) and 145.7 (C_q); JK6-100: HRMS (ESI$^+$) m/z: $[M+H]^+$ Calcd for $C_{19}H_{21}N$ 264.1752; found 264.1749.

Compound (±)-*Z*-7h: 1-(benzyl) 2-(1-(Z)-propenyl)-2-phenylpyrrolidine.

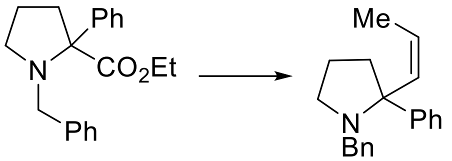

To a stirred solution of ester **S18** in dry THF (5.4 mL) at 0 °C under argon, $LiAlH_4$ (49 mg, 1.3 mmol) was added in one portion. The mixture was heated to 60 °C for 1 h then cooled 0 °C. The reaction was quenched sequentially with H_2O (50 µL), 15% aq NaOH (50 µL) and H_2O (150 µL). The mixture was stirred for 10 min, diluted with Et_2O (10 mL) and dried over $MgSO_4$ for 10 min. The mixture was filtered through Celite and eluted with Et_2O, DCM and EtOAc (10 mL each) and the filtrate evaporated to give the crude alcohol as clear oil (217 mg, <0.5 mmol). This was dissolved in dry DCM (830 µL) and added dropwise to a 15min pre-mixed solution of dry DMSO (55 µL, 0.8 mmol) and oxalyl chloride (66 µL, 0.8 mmol) in dry DCM (1.7 mL) at -78 °C under argon. The mixture was stirred for 1 h, Et_3N (457 µL, 3.3 mmol) was added dropwise. The mixture was stirred further for 2 h then allowed to warm to rt over 15 mins and quenched with saturated $NaHCO_3$ (15mL). The reaction was extracted with DCM (2 x 15mL), drying ($MgSO_4$) and evaporation organic of the organic phase afforded the crude aldehyde as a yellow oil (135.0 mg, <0.5 mmol). To a stirred solution of freshy made $EtPPh_3Br$ (756 mg, 2.0 mmol) in dry THF (6.6 mL) at rt under argon, NaH (76.0 mg of 60% NaH in mineral oil, 1.9 mmol) was added in one portion. The mixture was heated to 60 °C and stirred for 4 h. The orange solution was cooled to 0 °C, a solution of crude aldehyde in dry THF (2 mL) was added dropwise. The mixture was allowed to reach rt, stirred for 15 min then cooled to 0 °C. Petrol (26 mL) was added and stirred vigorously for 10 min. The mixture was filtered over Celite and the solids washed with petrol/ether (5:1, 20 mL). The filtrate was evaporated to give a yellow oil. Purification by silica gel chromatography (Et_2O/petrol, 0:100 to 3:97 as eluent) afforded the product as a clear oil (86.0 mg, 57% over 3 steps). ν_{max} /cm^{-1} (neat) 3060, 3023, 2963, 2912, 2799, 1492, 1445; δ_H ($CDCl_3$, 400 MHz) 1.35 (3H, d, J = 6.8 Hz, =CH*Me*), 1.92 (2H, h, J = 7.5 Hz, NCH_2CH_2), 2.26 (2H, t, J = 7.8 Hz, C_qCH_2), 2.59 (1H, q, J = 8.0 Hz, $NCHHCH_2$), 2.70 (1H, q, J = 8.0 Hz, $NCHHCH_2$), 3.29 (1H, d, J = 13.2 Hz, NCH*H*Ph), 3.49 (1H, d, J = 13.2 Hz, NCH*H*Ph), 5.71 – 5.90 (2H, m, C*H*=C*H*Me), 7.18 – 7.41 (8H, m, 8 x CH_{Ar}), 7.54 (2H,

d, J = 7.6 Hz, 2 x C*H*$_{Ar}$). δ_C ($CDCl_3$, 101 MHz) 16.1 (=CH*Me*), 22.0 (NCH_2*C*H_2), 41.5 (C_q*C*H_2), 49.7 (N*C*H_2), 54.0 (N*C*H_2Ph), 70.3 (N*C$_q$*Ph), 126.4 (*C*H$_{Ar}$), 126.7 (*C*H$_{Ar}$), 127.5 (*C*H$_{Ar}$), 127.8 (=C*H*Me), 128.0 – 128.6 (3 x *C*H$_{Ar}$), 133.2 (*C*H=CHMe), 140.7 (C_{qAr}), 144.1 (C_{qAr}); HRMS (ESI^+) m/z: $[M+H]^+$ calcd for $C_{23}H_{23}N$ 278.1910; found 278.1909.

Compound (±)-*E*-7h: 1-(benzyl)-2-(1-(E)-propenyl)-2-phenylpyrrolidine.

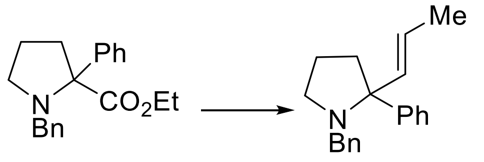

To a stirred solution of compound **S18** (304 mg, 1 mmol) in dry THF (10 ml) at 0 ˚C under argon, $LiAlH_4$ (94 mg, 2.5 mmol) was added in one portion. The mixture was stirred at 0 ˚C for 30 mins then quenched sequentially with water (100 µL), 15% aq. NaOH (100 µL) and water (300 µL) then stirred for 10 min. The mixture was diluted with Et_2O (15 ml) and dried over $MgSO_4$ for 15 min. The mixture was filtered over Celite and the product eluted with Et_2O, DCM and EtOAc (10 ml of each) and the filtrate evaporated to give the crude alcohol as a clear oil (268mg). This was dissolved in dry DCM (1.5 ml) and added dropwise to a 15 min pre-mixed solution of dry DMSO (98.6 µL, 1.4 mmol) and oxalyl chloride (120 µL, 1.4 mmol) in dry DCM at -78 ˚C under argon. The mixture was stirred for 1 h, Et_3N (806 µL, 5.8 mmol) was added dropwise and the mixture stirred further for 1 h then allowed to warm to rt over 15 min. The reaction was quenched with saturated $NaHCO_3$ (32 mL) and extracted with DCM (2 x 27 mL). The combined organic phase was dried ($MgSO_4$) and evaporated to give the crude aldehyde as a clear oil (274 mg) which was dissolved in toluene (11 mL) with stirring at rt. Freshly made phosphorane **S7** (1.3 g, 3.7 mmol) was added and the mixture heated to 90 ˚C. This was stirred for 15 h, allowed to cool to rt and evaporated. The residue was passed through a silica plug (Et_2O/petrol, 4:96 as eluent) to afford the crude allylic ester as clear oil (207 mg). This was dissolved dry THF (6 mL) at rt with stirring under argon and cooled to -10 ˚C. $LiAlH_4$ (48 mg, 1.3 mmol) was added in one portion, stirred for 30 mins then quenched sequentially with water (50 µL), 15% aq. NaOH (50 µL) and water (150 µL) then stirred for 10 min. The mixture was diluted with Et_2O (24 ml) and dried over $MgSO_4$ for 15 min. The mixture was filtered over Celite and the product eluted with Et_2O, DCM and EtOAc (15ml of each) and the filtrate evaporated to give the crude allylic alcohol as a clear oil (268mg). 72mg (>2.5 x 10^{-1} mmol) of the allylic alcohol wad used directly. To a stirred solution of crude allylic alcohol (72 mg, >2.5 x 10^{-1} mmol), LiBr (426 mg, 4.9 mmol) and Et_3N (177 µL, 1.3 mmol) in dry THF (2 mL) at – 78 ˚C, MsCl (25 µL, 0.32 mmol) was added. The mixture was stirred at – 78 ˚C for 1.5 hr then warmed to -40 ˚C and stirred further 1.5 h. Further MsCl (5 µL, 0.065 mmol) was added, the mixture was stirred further for 1 h 40 min then warmed to -10 ˚C and stirred further for 1 h. The mixture was quenched with saturated $NaHCO_3$ (15 mL) and extracted with DCM (2 x 20 mL). The combined organic phase was dried ($MgSO_4$) and evaporated to give the crude allylic bromide as a clear oil (140 mg). This was dissolved in dry THF (2.5 mL) at rt with stirring under argon and cooled to 0 ˚C, $LiALH_4$ (23 mg, 0.6 mmol) was added in one portion and the mixture heated to 60 ˚C. This was stirred for 3 h and cooled to 0 ˚C. The reaction was quenched with saturated $NaHCO_3$ (10 mL) and extracted with DCM (3 x 15 mL). The combined organic phase was dried ($MgSO_4$) and evaporated to give a crude oil. Purification by silica gel chromatography (Et_2O/petrol, 1:99 to 2:98 as eluent) afforded the *E*-alkene product as a clear oil (17 mg, 16% over 6 steps). ν_{max} /cm^{-1} (neat) 3026, 2962, 2914, 2878, 2801, 1600, 1493, 1446; δ_H ($CDCl_3$, 400 MHz) 1.73 – 2.09 (6H, m, CH=CH*Me* and NCH_2C*H_2* and C_qCH*H*), 2.26 (1H, t, J = 8.4 Hz, C_qCH*H*) 2.40 (1H, q, J = 8.3 Hz, NCH*H*), 2.99 (1H, td, J = 8.7, 3.6 Hz, NCH*H*), 3.25 (1H, d, J = 13.3 Hz, NCH*H*Ph), 3.75 (1H, d, J = 13.3 Hz, NCH*H*Ph), 5.71 (2H, s, C*H*=C*H*Me), 7.19 – 7.49 (8H, m, 8 x C*H*$_{Ar}$), 7.62 (2H, d, J = 7.7 Hz, 3 x C*H*$_{Ar}$); δ_C ($CDCl_3$, 100 MHz) 18.4 (CH=CH*Me*), 21.8 (NCH_2*C*H_2), 39.4 (C_q*C*H_2), 50.0 (N*C*H_2), 53.5 (N*C*H_2Ph), 70.6 (*C*$_q$Ph), 126.5 (*C*H$_{Ar}$), 126.6 (*C*H$_{Ar}$), 126.7 (*C*H=CHMe), 127.2 (*C*H$_{Ar}$), 128.1 (*C*H$_{Ar}$), 128.3 (*C*H$_{Ar}$), 128.4 (*C*H$_{Ar}$), 130.8 (CH=*C*HMe), 140.9 (C_{qAr}), 146.5 (C_{qAr}). HRMS (ESI^+) m/z: $[M+H]^+$ Calcd for $C_{20}H_{23}N$ 278.1910; found 278.1907.

Compound (±)-7i: 1-(methyl)-2-(1-(Z)-propenyl)-2-phenylpyrrolidine.

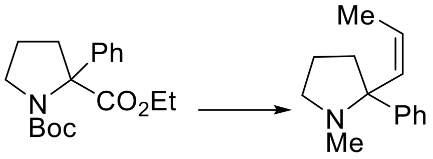

To a stirred solution of compound **S18** (279 mg, 0.9 mmol) in dry THF (7 ml) at 0 °C under argon, $LiAlH_4$ (80 mg, 2.1 mmol) was added in one portion. The mixture was heated to 60 °C and stirred for 4 h and cooled to 0 °C. The reaction was quenched sequentially with water (80 µL), 15% aq. NaOH (80 µL), and water (240 µL). The mixture was stirred for 10 mins, diluted with Et_2O (10 mL) and dried over $MgSO_4$ further for 10 min. The mixture was filtered over Celite and evaporated to give crude *N*-methyl alcohol as a clear oil (158 mg). This was dissolved in dry DCM (1.4 mL) under argon and added dropwise to a 15 min pre-mixed solution of dry DMSO (88.4 µL, 1.2 mmol) and oxalyl chloride (106.6 µL, 1.2 mmol) in dry DCM (2.7 ml) at -78 °C under argon and stirred for 1 h. Et_3N (732 µL, 5.3 mmol) was added dropwise, stirred for 10 min at -78 °C then warmed to rt and stir further for 30 min. The reaction was quenched with saturated $NaHCO_3$ (25 mL) and extracted with DCM (2 x 20 mL). The combined organic phase was dried ($MgSO_4$) and evaporated to give crude aldehyde as a pink oil (174 mg). To a stirred suspension of freshly made $EtPPh_3$ (1.3 g, 3.5 mmol) in dry THF (11 mL) at rt under argon, NaH (132 mg of 60% NaH dispersed in mineral oil, 3.3 mmol) was added in one portion. The mixture was heated to 60 °C and stirred for 4 h to give an orange solution. The solution was cooled to 0 °C and a solution of the crude aldehyde (< 0.9 mmol) in dry THF (2 mL) added dropwise. The mixture was warmed to rt and stirred for 15 min then cooled to 0 °C. Petrol (33 mL) was added and the mixture stirred vigorously for 10 min then filtered over Celite and the solids washed with petrol/Et_2O (20 mL). The filtrate was evaporated to give crude brown solid. Purification by silica gel chromatography (EtOH.NH_3/DCM, 0.5:99.5 to 4:96 as eluent) afforded product as a clear oil (103 mg, 59% over 3 steps). ν_{max}/cm^{-1} (neat) 3018, 2963, 2936, 2785, 1599, 1488, 1446, 1234; δ_H ($CDCl_3$, 400 MHz) 1.22 (3H, d, J = 6.5 Hz, CH=CH*Me*), 1.86 – 2.03 (2H, m, NCH_2CH_2), 2.08 (3H, s, N*Me*), 2.12 – 2.21 (1H, m, C_qCHH), 2.31 (1H, ddd, J = 12.8, 9.8, 6.7 Hz, C_qCHH), 2.63 – 2.79 (2H, m, NCH_2), 5.63 – 5.78 (2H, m, C*H*=C*H*Me), 7.17 – 7.39 (5H, m, 5 x CH_{Ar}); δ_C ($CDCl_3$, 100 MHz) 15.8 (CH=CH*Me*), 21.9 (NCH_2CH_2), 35.5 (N*Me*), 40.8 (C_qCHH), 52.8 (NCH_2), 69.6 (C_qPh), 126.3 (CH_{Ar}) , 127.5 (CH_{Ar}), 127.6 (CH=*C*HMe), 127.8 (CH_{Ar}) 133.0 (*C*H=CHMe), 142.6 (C_{qAr});); HRMS (ESI^+) m/z: [$M-H^+$] calcd for $C_{14}H_{17}N$ 200.1440; found 200.1438.

Compound (±)-7j: 1-(benzyl) 2-(1-(Z)-4-methyl-pentenyl)-2-phenylpyrrolidine.

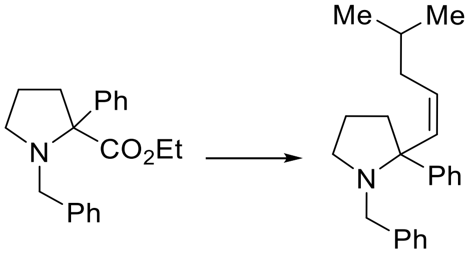

To a solution of **S18** (282 mg, 0.9 mmol) in dry THF (8 mL) with at 0 °C under argon was added $LiAlH_4$ (109 mg, 2.8 mmol) in one portion. The reaction was stirred at 0 °C for 5 min, warmed to 60 °C and stirred for 30 min. The reaction was cooled to 0 °C and water (110 µL), 15% aq. NaOH (110 µL) and water (330 µL) were added sequentially. The mixture was stirred for 10 min and Et_2O (50 mL) and $MgSO_4$ were added and the mixture filtered through Celite. Evaporation gave the product (272 mg, 97%) as a clear oil which was used directly. SO_3.py (303.0 mg, 1.9 mmol) was dissolved in dry DMSO (1.7 mL) and stirred at rt for 15 min. The resulting solution was added dropwise to a stirred solution of the crude alcohol and Et_3N (0.7 mL, 5 mmol) dissolved in DMSO (3.4 mL). was added and stirred at rt. The reaction was stirred at rt for 45 min and partitioned between Et_2O (10 mL) and sat. aq. $NaHCO_3$ (10 mL). The aq. phase was extracted further with Et_2O (10 mL). Drying ($MgSO_4$) and evaporation gave the crude aldehyde (237 mg, 87%) as a yellow oil, which was used directly without further purification. Isopentylphosphonium bromide **S6** (740 mg, 1.8 mmol) was dissolved/suspended in dry THF (5 mL) under argon at 0 °C. nBuLi (2.5 M, 680 µL of 2.5M nBuLi in hexanes, 1.7 mmol) was added dropwise over 2 min and the solution was warmed to rt. After 45 min the deep orange solution was cooled to 0 °C and a solution of crude aldehyde (237 mg, <0.89 mmol) in dry THF (2 mL) was added dropwise. The mixture was stirred for 10 min and acetaldehyde (50 µL) added to quench the residual ylide. Et_2O (20 mL) and petrol (20 mL) were added and the mixture filtered through Celite and evaporated. Purification by silica gel chromatography (5-20% Et_2O/petrol as eluent)

afforded the title compound (180 mg, 67% over 3 steps) as a clear oil. ν_{max} /cm^{-1}: 3040, 2954, 2870, 2800, 1493, 1453 and 1170; δ_H ($CDCl_3$, 400 MHz) 0.77 (6H, app. d, J 5.9, C*Me*$_2$), 1.49 – 1.67 (3H, m, C*H*Me_2 and allylic C*H*$_2$), 1.83 – 1.97 (2H, m, NCH_2C*H*$_2$), 2.21 – 2.30 (2H, m, NCH_2CH_2C*H*$_2$), 2.54 – 2.68 (2H, m, NC*H*$_2CH_2$), 3.18 (1H, d, J 13.3, NCH*H*Ph), 3.52 (1H, d, J 13.3, NC*H*HPh), 5.67 (1H, dt, J 11.8, 6.9, =C*H*-CH_2), 5.86 (1H, d, J 11.8, =C*H*-C_q), 7.14 – 7.35 (8H, m) and 7.49 (2H, d, J 7.6); δ_C ($CDCl_3$, 100 MHz) 22.1 (NCH_2*C*H_2), 22.5 (Me), 22.6 (Me), 28.7 (*C*HMe_2), 38.7 (allylic CH_2), 41.1 (NCH_2CH_2*C*H_2), 49.6 (N*C*H_2CH_2), 54.0 (N*C*H_2Ph), 70.4 (N*C*$_q$), 126.4 (CH_{Ar}), 126.7 (CH_{Ar}), 127.7 (2 × CH_{Ar}), 127.8 (2 × CH_{Ar}), 128.2 (2 × CH_{Ar}), 128.5 (2 × CH_{Ar}), 132.7 (=*C*H-CH_2) , 133.1 (=*C*H-C_q), 140.7 (C_q) and 143.9 (C_q); HRMS (ESI$^+$) m/z: [M+H]$^+$ Calcd for $C_{23}H_{29}N$ 320.2378; found 320.2376.

Compound (±)-S19: 1-(benzyl) 2-aceto-2-phenylpyrrolidine.

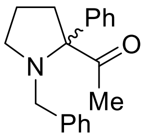

To a stirred solution of compound **S18** (304 mg, 0.98 mmol) in dry THF (8 ml) at 0 ˚C under argon, $LiAlH_4$ (90 mg, 2.4 mmol) was added in one portion. The mixture was heated to 60 ˚C and stirred for 4 h then cooled to 0 ˚C. The reaction was quenched sequentially with water (90 µL), 15% aq. NaOH (90 µL), and water (360 µL). The mixture was stirred for 10 mins, diluted with Et_2O (10 mL) and dried over $MgSO_4$ further for 10 min. The mixture was filtered over Celite and evaporated to give the crude alcohol as a clear oil (158 mg). To a stirred solution of anhydrous DMSO (0.10 mL, 1.4 mmol) in DCM (3 mL) at – 78 °C under argon was added oxalyl chloride (0.12 mL, 1.4 mmol) dropwise. The mixture was stirred for 15 min and a solution of crude alcohol (<0.98 mmol) in DCM (1.5 mL) dropwise. The reaction was stirred at – 78 °C for a further 1 h and triethylamine (0.83 mL, 6.0 mmol) added dropwise. The reaction was stirred for a further 3.5 h, warmed to rt and quenched by the addition of sat. aq. $NaHCO_3$ (30 mL). The mixture was extracted with DCM (2 × 25 mL) and the combined organic phase dried ($MgSO_4$) and evaporated to give the crude aldehyde (263 mg) as a yellow oil, which was used directly. To a solution of crude aldehyde (<0.98 mmol) in dry THF (5 mL) at 0 °C was added MeMgBr (0.60 mL of a 3 M solution in THF, 1.8 mmol) dropwise. The reaction was stirred at 0 °C for 1 h and further MeMgBr (0.90 mL of a 3 M solution in THF, 2.7 mmol) added dropwise. The reaction was stirred for 30 min, warmed to rt, stirred for 10 min and quenched by the addition of sat. aq. $NaHCO_3$ (20 mL). The mixture was extracted with EtOAc (2 × 25 mL) and the combined organic phase dried ($MgSO_4$) and evaporated to give the crude alcohol (240 mg) as a yellow oil, which was directly. To a stirred solution of anhydrous DMSO (0.10 mL, 1.4 mmol) in DCM (3 mL) at – 78 °C under argon was added oxalyl chloride (0.12 mL, 1.4 mmol) dropwise. The mixture was stirred for 15 min and a solution of crude alcohol (<0.98 mmol) in DCM (1.5 mL) dropwise. The reaction was stirred at – 78 °C for a further 1 h and triethylamine (0.83 mL, 6.0 mmol) added dropwise. The reaction was stirred for a further 30 min and warmed to rt over 15 min. The reaction was quenched by the addition of sat. aq. $NaHCO_3$ (25 mL). The mixture was extracted with DCM (2 × 25 mL) and the combined organic phase dried ($MgSO_4$) and evaporated to give the crude product as an orange oil. Purification by silica gel chromatography (EtOAc/petrol, 5:95 to 1:4 as eluent) afforded the title compound (152 mg, 56% over 4 steps) as a pale yellow oil. ν_{max} /cm^{-1} (film) 2958, 1711, 1672, 1601, 1494, 1448 and 1351; δ_H ($CDCl_3$, 400 MHz) 1.87 – 1.96 (2H, m, NCH_2C*H*$_2$), 2.14 – 2.26 (4H, m, NCH_2CH_2CH*H* and Me), 2.51 (1H, dt, J 14.6, 7.7, NCH_2CH_2C*H*H), 2.65 - 2.77 (2H, m, NC*H*$_2CH_2$), 3.28 (1H, d, J 13.2, NCH*H*Ph), 3.78 (1H, d, J 13.3, NC*H*HPh), 7.18 – 7.42 (10H, 2 × Ph); δ_C ($CDCl_3$, 101 MHz) 21.9 (NCH_2*C*H_2), 28.2 (Me), 36.3 (NCH_2CH_2*C*H_2), 50.4 (N*C*H_2CH_2), 54.6 (N*C*H_2Ph), 79.6 (N*C*$_q$Ph), 126.9 (CH_{Ar}), 127.48 (CH_{Ar}), 127.53 (2 × CH_{Ar}), 128.35 (2 × CH_{Ar}), 128.37 (2 × CH_{Ar}), 128.5 (2 × CH_{Ar}), 139.7 (Cq), 140.1 (Cq) and 209.8 (C=O); HRMS (ESI$^+$) m/z: [M+H]$^+$ calcd for $C_{19}H_{22}NO$ 280.1702; found 280.1698.

Compound (±)-7k: 1-(benzyl) 2-(3-methyl-ethenyl)-2-phenylpyrrolidine.

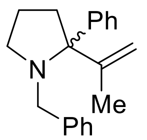

To a stirred suspension of $MePPh_3Br$ (770 mg, 2.15 mmol) in toluene (11 mL) at rt under argon was added KO^{t}Bu (240 mg, 2.14 mmol) and the mixture heated to 80 °C. After 80 min the yellow reaction was cooled to 0 °C and a solution of substrate **S20** (140 mg, 0.50 mmol) in toluene (2 mL) added dropwise. The stirred reaction was heated to 60 °C, stirred for 4 h and cooled to 0 °C. The excess ylide was quenched by the dropwise addition of acetaldehyde

(0.10 mL), the reaction stirred for 10 min and diluted with Et_2O/petrol (1:1, 35 mL). After stirring for a further 10 min the reaction was filtered though Celite and the filtrate evaporated to give a yellow oil. Purification by silica gel chromatography (2% Et_2O in petrol as eluent) to afford the title compound (85 mg, 61%) as a clear oil. ν_{max} /cm^{-1} (film) 2970, 1643, 1599, 1493, 1445 and 1363; δ_H ($CDCl_3$, 400 MHz) 1.70 (3H, s, Me), 1.79 -2.00 (2H, m, NCH_2CH_2), 2.19 (1H, td, J 12.1, 6.5, NCH_2CH_2CHH), 2.36 (1H, app. q, J 8.8, $NCHHCH_2$), 2.46 (1H, ddd, J 14.1, 10.0, 5.0, NCH_2CH_2CHH), 2.67 (1H, d, J 13.7, NCH*H*Ph), 2.88 (1H, td, J 9.3, 3.3, $NCHHCH_2$), 3.82 (1H, d, J 13.7, NC*H*HPh), 5.06 (1H, s, =CH*H*), 5.35 (1H, s, =C*H*H) and 7.17 – 7.40 (10H, m, 2 × Ph); δ_C ($CDCl_3$, 101 MHz) 21.56 (Me), 21.64 (NCH2*C*H2), 37.8 (NCH2CH2*C*H2), 50.7 (N*C*H2CH2), 54.9 (N*C*H2Ph), 74.2 (NC_qPh), 111.3 (=*C*H2), 126.5 (CH_{Ar}), 126.6 (CH_{Ar}), 127.7 (2 × CH_{Ar}), 128.20 (2 × CH_{Ar}), 128.22 (2 × CH_{Ar}), 128.3 (2 × CH_{Ar}), 140.80 (C_q), 140.85 (C_q) and 148.3 (C_q); HRMS (ESI$^+$) m/z: $[M+H]^+$ calcd for $C_{20}H_{24}N$ 278.1909; found 278.1907.

3.7 Preparation of 2-phenyl-octahydroindole allylic amine substrates

Compound S20: Methyl (2S,3aS,7aS)-1-[(phenyl)- carbamoyl]-octahydro-1H-indole-2-carboxylate.

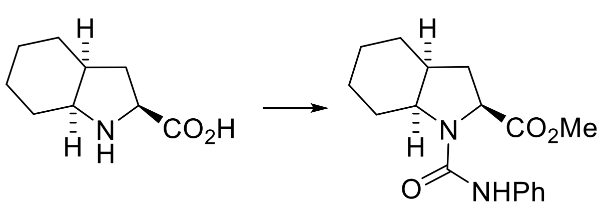

Using an adapted literature procedure,[11] a stirred suspension of (2S,3aS,7aS)-octahydro-1H-indole carboxylic acid (2.2 g, 13 mmol) in MeOH (33 mL) at 0 ˚C under argon, thionyl chloride (2.2 mL, 28 mmol) was added dropwise. The mixture was warmed to rt and stirred for 23 h. The mixture was evaporated, and the residue partitioned between saturated $NaHCO_3$ (45 mL) and DCM (90 mL). The phases were separated, and the aq. phase extracted further with DCM (2 x 90 mL). The combined organic phase was dried ($MgSO_4$) and evaporated to give the free-base amine ester as a clear oil (2.2 g). All spectra data was in accord with that reported.[11] This was dissolved in DCM (58 mL) at rt under argon, phenyl isocyanate (1.5 mL, 13 mmol) was added dropwise. The mixture was stirred for 1.5 h and quenched with water (60 mL). The phases were separated, and the aq. phase was extracted further with DCM (2 × 60 mL). The combined organic phase was dried ($MgSO_4$) and evaporated to give a white solid (5 g). Purification by silica gel chromatography (EtOAc/petrol, 1:9 to 1:1 as eluent) afforded the product as a fine white powder (3.7 g, 93% over 2 steps). All spectral data was in accord with that reported.[12]

Compound S21: (2R,3aS,7aS)-2-phenyl-octahydro-1H-indole-2-carboxylic acid.

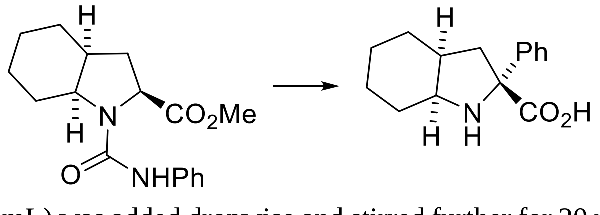

Following an adapted procedure,[12] to a stirred solution/suspension of compound **S20** (1.3 g, 4.1 mmol) in dry DCM (20.8 mL) at 0 ˚C under argon, TMSCl (3.8 mL, 29.9 mmol) was added dropwise. The mixture was warmed to rt and stirred for 15 h. The mixture was cooled to 0 ˚C, dry MeOH (7.2 mL) was added dropwise and stirred further for 20 min. The mixture was cooled to -78 ˚C and Et_3N (4.3 mL, 31 mmol) added dropwise. The mixture was added dropwise to a vigorously stirred solution of saturated $NaHCO_3$ (60 mL) at 0 ˚C and extracted with DCM (3 x 60 mL). The combined organic phase was dried ($MgSO_4$) and evaporated to give the crude product (1.6 g, ca. 88% conversion of by ^1H NMR to the MOM protected urea). To a stirred solution of crude MOM protected urea (1.6g, <4.1 mmol) in dry THF (41 mL) and LiCl (438 mg, 10.3 mmol) at -78 ˚C under argon, 20% KHMDS in THF (11.8 mL, 10.3 mmol) was added dropwise. The mixture was stirred at -78 ˚C for 30 min then warmed to rt and stirred further for 4 h. The mixture was poured over saturated $NaHCO_3$ (150 mL) and extracted with EtOAc (3 x 200 mL). The combined organic phase was dried ($MgSO_4$) and evaporated to give crude the hydantoin as a dark brown oil (1.1g). This was dissolved in dioxane (5.3 mL) with stirring at rt, 4 M aq. NaOH (32 mL) was added dropwise. The mixture was heated to 120 ˚C for 4 days. The

mixture was allowed to cool to rt and acidified with 3M HCl. The excess insoluble solids were filtered off. The acidic medium containing the product was loaded onto DOWEX50WX4 ion exchange resin (pre-washed with aq. 3M HCl). Once loaded the column was washed sequentially with one column length of water, dioxane and then water. The product was eluted with 35% NH_3 and the eluent evaporated under reduced pressure at 70 ˚C. The residual water was removed by azeotropic removal of toluene to afford the product as a yellow solid (400 mg, 40% over 3 steps). All spectral data was in accord with that reported.[12]

Compound S22: (2R,3aS,7aS)-1-benzyl-2-O-methyl-2-phenyl-octahydroindole-2-carboxylate.

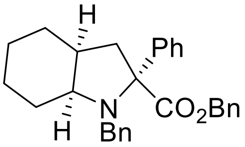

To a stirred suspension of amino acid **S21** (259 mg, 1 mmol) in MeCN (10 mL), K_2CO_3 (337 mg, 2.4 mmol), NaI (24 mg, 0.2 mmol) and BnBr (264 µL, 2.2 mmol) were sequentially added. The mixture was heated 60 ˚C and stirred for 22 h. The mixture was cooled to rt and evaporated. The resulting residue was partitioned between water (15 mL) and DCM (15 mL). The phases were separated, and the aq. phase extracted further with DCM (2 x 15 mL). The combined organic phase was dried ($MgSO_4$) to give an orange oil. Purification by silica gel chromatography (Et_2O/petrol, 0:100 to 5:95 as eluent) afforded product as a clear oil (266 mg, 59%). ν_{max} /cm^{-1} (neat) 3029, 2935, 2852, 1722, 1494, 1446. δ_H ($CDCl_3$, 400 MHz) 1.23 – 1.60 (8H, m, 4 x C*H₂*) 2.08 (1H, dd, J = 13.1, 7.8 Hz, C_qPhC*H*H), 2.27 (1H, m, NCHC*H*), 2.99 (1H, dd, J = 13.1, 10.9 Hz, C_qPhCH*H*), 3.79 (1H, d, J = 14.7 Hz, NCH*H*Ph), 3.91 (1H, d, J = 14.7 Hz, NCH*H*Ph) 5.19 (1H, d, J = 12.1 Hz, CO_2CH*H*Ph), 5.29 (1H, d, J = 12.1 Hz, CO_2CH*H*Ph), 7.15 – 7.40 (15H, m, 15 x CH_{Ar}). δ_C ($CDCl_3$, 101 MHz) 21.6 (*C*H_2), 23.0 (*C*H_2), 25.2 (*C*H_2), 26.8 (*C*H_2), 35.6 (NCH*C*H), 42.9 (C_qPh*C*H_2), 50.7 (N*C*H_2Ph), 59.9 (N*C*HCH), 66.8 (CO_2*C*H_2), 74.4 (N*C*$_q$Ph), 126.5 - 126.9 (3 x *C*H_{Ar}), 128.0 – 128.7 (5 x *C*H_{Ar}), 135.9 (C_{qAr}), 141.0 (C_{qAr}), 145.2 (C_{qAr}), 175.8 (*C*O_2Bn); HRMS (ESI$^+$) m/z: $[M+H]^+$ calcd for $C_{29}H_{31}NO_2$ 426.2435; found 426.2434.

Compound 7o: (2R,3aS,7aS)-1-benzyl-2-vinyl-2-phenyl-octahydroindole.

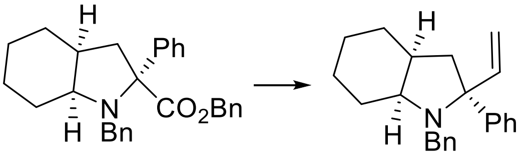

To a stirred solution of compound **S22** (486 mg, 1.1 mmol) in dry THF (11.5 mL) at 0 ˚C, $LiAlH_4$ (95 mg, 2.5 mmol) was added in one portion. The mixture was warmed to rt, stirred for 50 min then cooled to 0 ˚C. The reaction was quenched sequentially with water (100 µL), 15% aq. NaOH (100 µL) and water (300 µL), stirred for 10 min then diluted with Et_2O (10 mL) and dried over $MgSO_4$ for 10 min. The mixture was filtered through Celite and product eluted with Et_2O, DCM and EtOAc (10 mL of each) and the filtrate evaporated to give a crude 1:1 mixture of the secondary alcohol and BnOH (498 mg). This was dissolved in dry DCM (3.6 mL) under argon and added dropwise to a 15 min pre-mixed solution of dry DMSO (234 µL, 3.3 mmol) and oxalyl chloride (285 µL, 3.3 mmol) in dry DCM (7 mL) at – 78 ˚C. The mixture was stirred for 1 h, Et_3N (1.9 mL, 13.6 mmol) was added dropwise and the mixture stirred further for 1 h. The mixture was allowed to warm to rt over 15 min, quenched with saturated $NaHCO_3$ (20 mL) and extracted with DCM (2 x 15 mL). The combined organic phase was dried ($MgSO_4$) and evaporated to give a crude 1:1 mixture of aldehyde product and PhCHO (599 mg). To a stirred suspension of $MePPh_3Br$ (1.6 g, 4.6 mmol) in toluene (15 ml) at rt, ^{t}BuOK (511 mg, 4.6 mmol) was added in one portion. The mixture was heated to 90 ˚C and stirred for 1.5 h, forming a yellow solution. This was cooled to 0 ˚C and a solution of the crude aldehyde in toluene (3 mL) added dropwise. The mixture was warmed to rt and stirred for 30 min then cooled to 0 ˚C. The excess ylide was quenched with MeOH (1 mL), Et_2O (18 mL) was added and the mixture stirred vigorously for 10 min then filtered over Celite. The solids were washed with Et_2O (25 mL) and the filtrate evaporated to give an orange oil. Purification by silica gel chromatography (Et_2O/petrol, 0.5:99.5 to 3:97 as eluent) afforded the product as a clear oil (96 mg, 53% over 3 steps). ν_{max} /cm^{-1} (neat) 3081, 3060, 3026, 2921, 2851, 2806, 1493, 1453; δ_H ($CDCl_3$, 400 MHz) 1.18 – 1.68 (8H, m, 4 x C$H_{2(cyclohexyl)}$), 1.94 (1H, dd, J = 12.3, 7.2 Hz, C_qPhCH*H*), 2.23 – 2.33 (1H, m, NCHC*H*), 2.48 (1H, t, J

= 12.2 Hz, NC_qPhCHH), 3.07 (1H, dt, 10.9, 5.6 Hz, NC*H*C), 3.68 (1H, d, J = 14.5 Hz, NCH*H*Ph), 3.84 (1H, d, J = 14.5 Hz, NCH*H*Ph), 5.08 (1H, d, 17.4 Hz, CH=CH*H*), 5.28 (1H, d, J = 10.6 Hz, CH=CH*H*), 6.22 (1H, dd, J = 17.4, 10.8 Hz, C*H*=CH_2), 7.12 – 7.24 (2H, m 2 x CH_{Ar}), 7.31 (4H, td, J = 7.2, 2.3, 4 x CH_{Ar}), 7.45 (2H, d, J = 7.5 Hz, 2 CH_{Ar}), 7.58 (2H, d, J = 7.5 Hz, 2 CH_{Ar}). δ_C ($CDCl_3$, 101 MHz) 21.4 ($CH_{2(cyclohexyl)}$), 23.5 ($CH_{2(cyclohexyl)}$), 26.0 ($CH_{2(cyclohexyl)}$), 26.6 ($CH_{2(cyclohexyl)}$), 34.9 (NCHCH), 43.4 (C_qPhCH_2), 50.4 (NCH_2Ph), 59.0 (N*C*HCH), 70.3 (NC_qPh), 114.9 (CH=CH_2), 126.1 (CH_{Ar}), 126.5 (CH_{Ar}), 127.6 and 127.8 (2 x CH_{Ar}), 128.2 and 128.4 (2 x CH_{Ar}), 145.6 (*C*H=CH_2) 141.3 (C_{qAr}), 147.8 (C_{qAr}); HRMS (ESI^+) m/z: $[M+H]^+$ Calcd for $C_{23}H_{27}N$ 318.2223; found 318.2220.

Compound 7p: (2R,3aS,7aS)-1-benzyl-2-(1-(Z)-propenyl)-2-phenyl-octahydroindole.

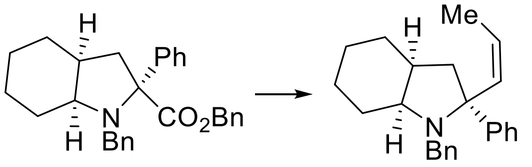

To a stirred solution of compound **S22** (337 mg, 0.8 mmol) in dry THF (8 mL) at 0 °C under argon, $LiALH_4$ (66 mg, 1.7 mmol) was added in one portion. The mixture was warmed to rt and stirred 2.5 h. The reaction was quenched sequentially with water (70 µL,), 15% aq. NaOH and water (210 µL). The mixture was stirred for 10 min, diluted with Et_2O (10 mL) and dried over $MgSO_4$ for 10 min. The mixture was filtered through Celite and eluted sequentially with Et_2O (10 mL), DCM (10.0 mL) and EtOAc (10 mL). The filtrate was evaporated to give a 1:1 mixture of the secondary alcohol product and benzyl alcohol (338 mg). A solution of the crude alcohol mixture in DCM (2.5 mL) was added dropwise to a 15 min pre-mixed solution of oxalyl chloride (198 µL, 2.3 mmol) and dry DMSO (162 µL, 2.3 mmol) in DCM (4.9 mL) at -78 °C under argon. The mixture was stirred for 1 h, Et_3N (1.3 mL, 9.5 mmol) was added dropwise and stirred further for 1 h. The mixture was allowed to warm to rt over 15 min, quenched with saturated $NaHCO_3$ (15 mL) and extracted with DCM (3 x 10 mL). The combined organic phase was dried ($MgSO_4$) and evaporated to give a 1:1 mixture of the aldehyde product and benzaldehyde as an orange oil (339 mg, of which 75% was used in the subsequent reaction, <0.59 mmol). To a stirred suspension of $EtPPh_3Br$ (2 g, 5.4 mmol) in dry THF at rt under argon, NaH in mineral oil (60% dispersion, 194 mg, 4.9 mmol) was added portionwise. The mixture was heated to 60 °C for 5 h forming a bright orange solution. This was cooled to 0 °C, a solution of the crude aldehyde mixture (ca. 0.59 mmol) in dry THF (3 mL) was added dropwise. The mixture was warmed to rt and stirred for 20 min then quenched with MeOH (200 µL). The mixture was cooled to 0 °C, petrol (57 mL) wad added and stirred vigorously for 10 min. The mixture was filtered over celite, and solids washed with petrol/Et_2O (5:1, 20 mL). The filtrate was evaporated to give crude product as orange oil. Purification by silica gel chromatography (Et_2O/Petrol, 0% 1% as eluent) afforded the product as a clear oil (155 mg, 59% over 3 steps). $[\alpha]^{20}_D$ = +61.2 (*c* 0.12, DCM). ν_{max} /cm^{-1} (neat) 3060, 3023, 2924, 2852, 1493, 1145, 1362; δ_H ($CDCl_3$, 400 MHz) 1.20 – 1.73 (11H, m, 4 x $CH_{2(cyclohexyl)}$ and =CH*Me*), 2.23 – 2.49 (3H, m, C_qCH_2 and NCHC*H*), 2.96 (1H, dt, J = 9.9, 5.4 Hz, NC*H*) 3.30 (1H, d, J = 14.2 Hz, NCH*H*Ph), 3.82 (1H, d, 14.2 Hz, NCH*H*Ph). 5.64 (1H, m, CH=C*H*Me), 6.06 (1H, d, J = 11.4 Hz, C*H*=CHMe), 7.15 – 7.39 (8H, m, 8 x CH_{Ar}), 7.58 (2H, d, J = 7.7 Hz, 2 x CH_{Ar}); δ_C ($CDCl_3$, 101 MHz) 16.0 (=CH*Me*), 21.4 ($CH_{2(cyclohexyl)}$), 22.0 ($CH_{2(cyclohexyl)}$), 22.8 ($CH_{2(cyclohexyl)}$), 27.8 ($CH_{2(cyclohexyl)}$), 36.4 (NCH*C*H), 48.0 (C_qPhCH_2), 49.8 (NCH_2Ph), 59.0 (N*C*H), 69.5 (C_qPh), 125.8 (CH=*C*HMe), 126.0 (CH_{Ar}), 126.4 (CH_{Ar}), 127.7 (CH_{Ar}), 128.4 (CH_{Ar}), 138.8 (*C*H=CHMe), 141.7 (C_{qAr}), 147.9 (C_{qAr}); HRMS (ESI^+) m/z: $[M+H]^+$ calcd for $C_{24}H_{29}N$ 332.2380; found 332.2378.

3.8 Preparation of 2-phenylpiperidine allylic amine substrates

Compound (±)-S23: N-Boc-2-phenylpiperidine.

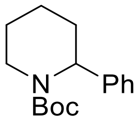

To a stirred solution of 2-phenylpiperidine (1.00 g, 6.2 mmol) in THF (15 mL) at rt under argon was added Boc_2O (1.60 g, 7.3 mmol) in one portion. The reaction was stirred for 1 h, evaporated and purified by silica gel chromatography (EtOAc/petrol, 1:19 to 5:95 as eluent) to afford the title compound (1.42 g, 88%) as a clear oil which crystallised on prolonged standing. All spectral data was in accord with that reported.[10]

Compound (±)-S24: 1-(tert-butyl) 2-ethyl 2-phenylpiperidine-2-carboxylate.

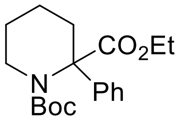

Following an adapted literature procedure,[9] a stirred solution of substrate **S23** (500 mg, 1.92 mmol) in dry THF (12 mL) was degassed by sparging with argon and cooled to -40 °C. ^{n}BuLi (0.98 mL of a 2.5 M solution in hexanes, 2.4 mmol) was added dropwise over 2 min and the resulting yellow solution stirred for 30 min. Ethyl chloroformate (0.64 mL, 6.7 mmol) was added dropwise to give a blood red solution. The reaction was allowed to warm to rt over 3 h and the colourless reaction was quenched by the addition of methanol (5 mL) and evaporated. The residue was purified by silica gel chromatography (EtOAc/petrol, gradient elution: 5:95 to 1:4 as eluent) to afford the title compound (500 mg, 78%) as a clear oil. All spectral data was in accord with the literature.

Compound (±)-S25: 1-(benzyl)-2-ethyl-2-phenylpiperidine-2-carboxylate.

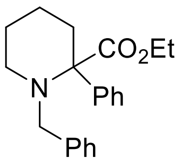

To a stirred solution of compound **S24** (470 mg, 1.4 mmol) in DCM (7 mL) at 0 °C under argon was added TFA (7 mL, 91 mmol) dropwise. The reaction was warmed to rt, stirred for 2.5 h and evaporated to give a yellow oil which was redissolved in MeCN (10 mL). Potassium carbonate (550 mg, 4.0 mmol), sodium iodide (20 mg, 0.13 mmol) and benzyl bromide (210 µL, 1.8 mmol) were added, and the reaction heated to 60 °C. After 14 h the reaction was cooled and concentrated in vacuo. The residue was partitioned between water (20 mL) and DCM (15 mL), the phases separated, and the aqueous phase extracted with DCM (15 mL). Drying ($MgSO_4$) and evaporation gave the crude product as a pink oil. Purification by silica gel chromatography (Et_2O/petrol, 1% to 5% as eluent) afforded product as a clear oil (315 mg, 69% over two steps) as a clear oil. ν_{max} /cm^{-1} (film) 2939, 1724, 1493, 1445 and 1222; δ_H ($CDCl_3$, 400 MHz) 1.40 (3H, t, J 7.2, Me), 1.46 – 1.69 (3H, m, NCH_2CH_2 and C_q-CH_2CHH), 1.74 (1H, d, J 13.3, C_q-CH_2CH*H*), 1.85 (1H, td, J 12.8, 3.7, C_q-C*H*H), 2.36 (1H, d, J 13.2, C_q-CH*H*), 2.49 (1H, app. t, J 11.9, NCH*H*CH_2), 2.81 (d, J 12.4, NC*H*HCH_2), 3.69 (1H, d, J 15.6, NCH*H*Ph), 3.80 (1H, d, J 15.6, NC*H*HPh), 4.39 (2H, app. q, J 7.3, CH_2Me), 7.15 – 7.23 (2H, m), 7.24 – 7.36 (6H, m) and 7.38 – 7.48 (2H, m); δ_C ($CDCl_3$, 100 MHz) 14.7 (Me), 23.0 (C_q-CH_2CH_2), 25.9 (NCH_2CH_2), 39.8 (C_q-CH_2), 47.9 (NCH_2CH_2), 56.0 (NCH_2Ph), 60.5 (OCH_2Me), 73.2 (NC_q), 126.3 (CH_{Ar}), 126.7 (2 × CH_{Ar}), 127.5 (CH_{Ar}), 127.7 (2 × CH_{Ar}), 128.2 (2 × CH_{Ar}), 128.4 (2 × CH_{Ar}), 141.5 (C_q), 143.8 (C_q) and 174.2 (CO_2Et); HRMS (ESI$^+$) m/z: [M+H]$^+$ calcd for $C_{21}H_{25}NO_2$ 324.1965; found 324.1965.

Compound 7q: 1-(benzyl)-2-vinyl-2-phenylpiperidine.

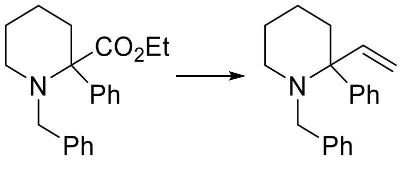

To a stirred solution of substrate **S25** (190 mg, 0.6 mmol) in dry THF (5.5 mL) at 0 °C under argon was added $LiAlH_4$ (70 mg, 1.8 mmol) in one portion. The mixture was stirred for 80 min, heated to 60 °C, stirred for an additional 30 min and cooled to 0 °C. Water (70 µL), 15% aq. NaOH (70 µL) and water (210 µL) were added sequentially, and the mixture stirred for 10 min, diluted with Et_2O (15 mL) and dried over $MgSO_4$ for a further 10 min. The mixture was filtered through Celite, eluting with EtOAc and DCM, and the filtrate evaporated to give a clear oil (180 mg). The crude alcohol and triethylamine (410 µL, 2.9 mmol) were dissolved in dry DMSO (2 mL) at rt under argon. A solution of SO_3.py (175 mg, 1.1 mmol) in dry DMSO (1 mL, solution pre-mixed for 15 min) was added dropwise and the reaction stirred at rt. After 13 h the reaction was partitioned between Et_2O (25 mL) and half-saturated aq. $NaHCO_3$ (40 mL). The phases were separated, the aqueous phase extracted with Et_2O (20 mL) and the combined organic phase

dried ($MgSO_4$) and evaporated to give the crude aldehyde (153 mg) as a red oil which was used directly without further purification. To a stirred suspension of methyltriphenylphosphonium bromide (830 mg, 2.3 mmol) in dry toluene (12 mL) at rt under argon was added potassium *tert*-butoxide (260 mg, 2.3 mmol) in one portion and the reaction heated to 75 °C. After 2 h the yellow mixture was cooled to 0 °C and a solution of crude aldehyde (<0.54 mmol) in toluene (2 mL) added dropwise. The reaction was stirred at 0 °C for 10 min, warmed to rt, stirred for an additional 10 min and the excess ylide quenched by the addition of acetaldehyde (0.10 mL). The reaction was cooled to 0 °C and diluted with Et_2O/petrol (15 mL, 1:2). After stirring for a further 5 min the mixture was filtered through Celite and the filtrate evaporated. The residue was purified by silica gel chromatography (Et_2O/petrol, 5:95 as eluent) to afford the title compound (87 mg, 53% over 3 steps) as a clear oil. ν_{max} /cm^{-1} (film) 2934, 2798, 1600, 1492 and 1444; δ_H ($CDCl_3$, 400 MHz) 1.55 – 1.76 (4H, m, $NCH_2CH_2CH_2$), 1.83 (1H, app. t, J 12.2, C_qCHH), 2.05 (1H, dd, J 13.6, 4.3, C_qCHH), 2.40 (1H, dt, J 12.1, 6.4, $NCHHCH_2$), 2.71 (1H, d, J 12.1, $NCHHCH_2$), 3.24 (1H, d, J 14.4, NCH*H*Ph), 3.74 (1H, d, J 14.4, NC*H*HPh), 5.31 (1H, d, J 17.9, =CH*H*), 5.64 (1H, d, J 11.2, =C*H*H), 6.09 (1H, dd, J 18.0, 11.2, $CH=CH_2$), 7.15 – 7.23 (2H, m), 7.26 – 7.34 (4H, m), 7.38 (2H, d, J 7.8) and 7.69 (2H, d, J 7.7); δ_C ($CDCl_3$, 100 MHz) 22.0 (CH_2), 26.0 (CH_2), 37.9 (Cq-CH_2), 47.3 (NCH_2CH_2), 54.6 (NCH_2Ph), 66.5 (N*C*q), 118.7 (=CH_2), 126.4 (CH_{Ar}), 126.6 (CH_{Ar}), 127.1 (2 × CH_{Ar}), 128.0 (2 × CH_{Ar}), 128.2 (4 × CH_{Ar} – *two environments overlap*), 137.0 ($CH=CH_2$), 140.8 (C_q) and 147.8 (C_q); HRMS (ESI$^+$) m/z: $[M+H]^+$ Calcd for $C_{23}H_{29}N$ 278.190874; found 278.1905.

Compound (±)-7r: 1-(benzyl)-2-(1-(Z)-propenyl)-2-phenylpiperidine.

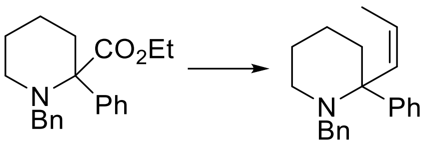

To a stirred solution of compound **S25** (336.0 mg, 1.0 mmol) in dry THF (9.7 mL) at 0 °C under argon, $LiALH_4$ (124 mg, 3.3 mmol) was added in one portion. The mixture was heated to 60 °C for 1 h then cooled to 0 °C. Water (130 µL), 15% aq. NaOH (130 µL) and water (390 µL) were added sequentially, and the mixture stirred for 10 min, diluted with Et_2O (26 mL) and dried over $MgSO_4$ for a further 15 min. The mixture was filtered through Celite, eluting with EtOAc (10 mL) and DCM (10 mL). The filtrate was evaporated to give crude mono-alcohol as clear oil (254 mg, <0.9 mmol). This was dissolved in dry DMSO (2.5 mL) at under argon and Et_3N (760 µL, 5.5 mmol) added. To this, a 15 min premixed solution of SO_3.Py (313.0 mg, 2.00 mmol) was added dropwise, and the mixture stirred for 18 h. The mixture was partitioned between saturated $NaHCO_3$ (50 mL) and Et_2O (40 mL). The phases were separated, and the aq. phase extracted further with Et_2O (40 mL). The combined phase was dried ($MgSO_4$) and evaporated to give crude red oil (244.0 mg, 75% conversion of SM by H-NMR). The crude oil was re-dissolved in dry DMSO (2.5 mL) at under argon and Et_3N (380 µL, 2.7 mmol) added. To this, a 15 min premixed solution of SO_3.Py (157 mg, 1. mmol) was added dropwise, and the mixture stirred for 18 h. The mixture was partitioned between saturated $NaHCO_3$ (30 mL) and Et_2O (30 mL). The phases were separated, and the aq. phase extracted with Et_2O (30 mL). The combined organic phase was dried ($MgSO_4$) and evaporated to give the crude aldehyde as a red oil (186 mg, <0.6 mmol). To a stirred suspension of $EtPPh_3Br$ (958 mg, 2.6 mmol) in dry THF (12 mL) at -10 °C under argon, nBuLi (970 µL of a 2.5M solution in hexane, 2.4 mmol) was added dropwise. The mixture was stirred for 30 min until orange solution had formed. The crude aldehyde was dissolved in dry THF (2 mL) under argon and added to the ylide solution. The mixture was warmed to rt and stir for 20 min. The mixture was quenched with MeOH (2 mL), cooled to 0 °C and diluted with petrol (30 mL). The mixture was vigorously stirred for 10 min then filtered over Celite. The solids were washed with petrol/Et_2O (5:1, 20 mL) and the filtrate evaporated to give crude oil. Purification by silica gel chromatography (Et_2O/petrol, 1:99 to 1:9) afforded the product as a clear oil (136 mg, 52% over 3 steps). ν_{max} /cm^{-1} (neat) 3019, 2933, 2857, 2798, 1600, 1492, 1444; δ_H ($CDCl_3$, 400 MHz) 1.53 – 1.69 (7H, m, =CH*Me* and NCH_2CH_2 and $C_qCH_2CH_2$), 1.86 – 1.97 (1H, m, C_qCHH), 2.17 (1H, d, J = 13.2 Hz, C_qCHH), 2.48 (1H, td, J = 13.3, 6.5 Hz, NCH*H*), 2.71 (1H, d, J = 11.6 Hz), 3.08 (1H, d, J = 14.1 Hz, NCH*H*Ph), 3.57 (1H, d, J = 14.1 Hz, NCH*H*Ph), 5.74 (1H, d, J = 12.3 Hz, C*H*=CHMe), 5.92 – 6.04 (1H, m, CH=C*H*Me), 7.14 – 7.36 (8H, m, 8 x CH_{Ar}), 7.71 (2H, d, J = 7.7 Hz, 2 x CH_{Ar}). δ_C ($CDCl_3$, 101 MHz) 16.3 (=CH*Me*), 22.1 ($C_qCH_2CH_2$), 26.0 (NCH_2CH_2), 40.3 (C_qCH_2), 47.3 (NCH_2), 54.9 (NCH_2Ph), 67.0 (NC_qPh), 126.4 (CH_{Ar}), 127.0 (CH_{Ar}), 127.6 (CH_{Ar}), 128.1 – 128.2 (3 x CH_{Ar}), 128.4 (=*C*HMe), 128.9 (*C*H=CHMe), 140.9 (C_{qAr}), 146.2 (C_{qAr}). HRMS (ESI$^+$) m/z: $[M+H]^+$ Calcd for $C_{21}H_{25}N$ 292.2065; found 292.2063.

3.9 Preparation of trans-alkene isomer 7r

Compound (*R*)-S26: 2-Trichloromethyl-5-(1-(allyl)-1-aza-3-oxabicyclo[3.3.0]- octane-4-one.

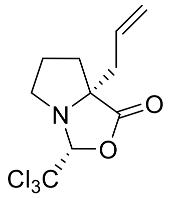

Following an adapted procedure.[7] To a stirred solution of $^iPr_2NH$ (2.40 mL, 17.4 mmol) in dry THF (35 mL) at -78 °C under argon, ^nBuLi (9.0 mL of a 2.0 M solution in hexane, 18 mmol) was added dropwise. The mixture was stirred for 30 min and a pre-cooled solution at 0 °C of protected proline compound **(S)-S9** (3.00 g, 12.3 mmol) in dry THF (25 mL) under argon added to the stirred LDA solution over 4 min. The mixture was stirred for 30 min and allyl bromide (1.9 mL, 22 mmol) was added dropwise. The mixture was warmed to -40 °C and stirred for a further 30 min. The reaction was poured over water (70 mL) and extracted with DCM (3 × 70 mL). The combined organic phase was dried ($MgSO_4$) and evaporated to give the crude product as a brown oil. Purification by silica gel chromatography (EtOAc/petrol, 5:95 to 1:4 as eluent) afforded the title compound as a clear oil (1.7 g, 49%). All spectral data was in accord with that reported.[7]

Compound (*R*)-S27: N-benzyl-O-benzylpyrrolidine-2-(1-(allyl)-2-carboxylate.

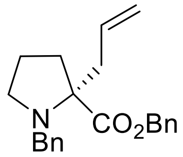

To a stirred solution of compound **S26** (1.7 g, 6.0 mmol) in 2-propanol (28 mL) at rt, aq. HCl (6 M, 28 mL) was added. The mixture was stirred at rt for 5 days, heated 50 °C for 6 h and cooled to rt. The reaction was evaporated under reduced pressure and the residual water removed by azeotropic removal of toluene (4 × 60 mL) to give a white oily solid. This was dissolved in MeCN (42 mL) at rt with stirring. K_2CO_3 (2.6 g, 19.2 mmol), NaI (135 mg, 0.9 mmol) and BnBr (1.50 mL, 12.6 mmol) were added sequentially and the mixture heated to 90 °C. After 17 h the reaction was cooled to rt and evaporated. The residue was dissolved in water (90 mL) and extracted with DCM (2 × 90 mL). The combined organic phase was dried ($MgSO_4$) and evaporated to give a brown oil. Purification by silica chromatography (Et_2O/petrol, 1:99 to 1:9 as eluent) afforded the product as a clear oil (1.1 g, 57% over 2 steps). $[\alpha]^{20}_D$ = +47.8 (*c* 0.680, DCM). ν_{max}/cm^{-1} (neat) 3065, 3030, 2949, 2806, 1720, 1512, 1454; δ_H ($CDCl_3$, 400 MHz) 1.64 – 1.86 (3H, m, NCH_2CH_2 and NCH_2CH_2CHH), 2.18 (1H, ddd, J = 12.2, 8.9, 4.9 Hz, NCH_2CH_2CHH), 2.44 – 2.61 (2H, m, NCH*H* and CH*H*CH=CH_2), 2.71 (1H, dd, J = 14.2, 7.6 Hz, CH*H*CH=CH_2)), 2.86 (1H, td, J = 8.4, 3.3 Hz, NCH*H*), 3.30 (1H, d, J = 13.4 Hz, NCH*H*Ph), 3.97 (1H, d, J = 13.5 Hz, NCH*H*Ph),), 5.05 – 5.25 (4H, m, CO_2CH_2Ph and CH=CH_2), 5.91 (1H, ddt, J = 17.1, 10.1, 7.1 Hz, C*H*=CH_2), 7.16 – 7.45 (10H, m, 10 ×CH_{Ar}); δ_C ($CDCl_3$, 101 MHz) 21.8 (NCH_2CH_2), 33.9 ($NCH_2CH_2CH_2$, 39.3 (CH_2CH=CH_2), 51.6 (NCH_2), 53.4 (NCH_2Ph), 66.2 (CO_2CH_2Ph), 70.3 (N*C*CO_2), 118.1 (CH=CH_2), 126.8 (CH_{Ar}), 128.3 – 128.8 (5 × CH_{Ar}), 134.3 (*C*H=CH_2), 136.2 (C_{qAr}), 140.3 (C_{qAr}) 174.3 (CO_2Bn); HRMS (ESI$^+$) m/z: $[M+H]^+$ Calcd for $C_{22}H_{25}NO_2$ 336.1965; found 336.1959.

Compound (*R*)-7r: N-benzyl-O-benzylpyrrolidine-2-(1-(*E*)-propenyl)-2-carboxylate.

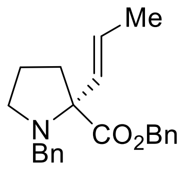

To a flame-dried Schlenk tube under argon, $Ru(PPh_3)_3H(CO)Cl$, (145.5 mg, 0.15 mmol) was added followed by dry THF (5 mL). A solution of compound **S27** (512.6 mg, 1.5 mmol) in dry THF (10 mL) was added and the stirred mixture heated to 70 °C. After 16.5 h the mixture was allowed to cool to rt and evaporated. Purification by silica gel chromatography (petrol/$CHCl_3$, 4:6 to 1:9 as eluent) afforded the product as a clear oil (304 mg, 59%). $[\alpha]^{20}_D$ = +4.25 (*c* 0.561, DCM). ν_{max}/cm^{-1} (neat) 3063, 3030, 2960, 2831, 1722, 1495, 1453; δ_H ($CDCl_3$, 400 MHz) 1.72 (3H, d, J = 4.8 Hz, CH=CH*Me*), 1.73 – 1.93 (3H, m, NCH_2CH_2 and NCH_2CH_2CHH), 2.36 (1H, dt, J = 12.5, 6.7 Hz, NCH_2CH_2CHH), 2.62 (1H, q, J = 7.5 Hz, NCH*H*), 2.88 (1H, dt, J = 8.7, 6.7 Hz), 3.91 (1H, d, J = 13.9 Hz, NCH*H*Ph), 5.16 (2H, A/B q, CO_2CH_2Ph), 5.64 – 5.78 (2H, m, C*H*=C*H*Me), 7.16 – 7.41 (10H, m, 10 × CH_{Ar}); δ_C ($CDCl_3$, 101 MHz) 18.3 (CH=CH*Me*), 21.7 (NCH_2CH_2), 37.9 ($NCH_2CH_2CH_2$), 50.5 (NCH_2), 53.8 (NCH_2Ph), 66.3 (CO_2CH_2Ph), 77.6 (N*C*CO_2), 126.4 (*C*H=CHMe), 126.7 (CH_{Ar}), 128.3 – 128.6 (3 × CH_{Ar}), 131.5 (CH=*C*HMe), 136.2 (C_{qAr}), 140.8 (C_{qAr}), 174.3 (CO_2Bn); HRMS (ESI$^+$) m/z: $[M+H]^+$ Calcd for $C_{22}H_{25}NO_2$ 336.1965; found 336.1957.

3.10 Palladium-catalysed ring expansion reactions

General procedure 1 for ring expansion reactions

To a dried Schlenk tube was added [Pd(allyl)Cl]$_2$ (5 mol%) followed by the sequential addition of dry solvent, P(OEt)$_3$ (20 mol%) and morpholine (40 mol%). The mixture was stirred at rt for 5 min, forming a yellow solution. Substrate (1 equiv.) in dry solvent was added followed by TFA (1 equiv). The Schlenk tube was sealed and the stirred mixture heated to the specified temperature. After 16 h the reaction was cooled and the mixture was taken up in DCM (15 mL) and washed with saturated $NaHCO_3$ (15 mL). The phases were separated, and the aq. phases extracted with DCM (15 mL). The combined organic phase was dried over ($MgSO_4$) and evaporated to give crude product which was purified by silica gel chromatography.

Compound 8a: 2,3,4,7-tetrahydro-N-benzyl-azepane-O-benzyl-5-carboxylate.

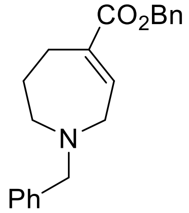

Following general procedure 1 employing [Pd(allyl)Cl]$_2$ (2.75 mg, 7.5 x 10^{-3} mmol) DCM, (0.7 mL), P(OEt)$_3$ (5 µL, 0.029 mmol), morpholine (5 µL, 0.057 mmol), substrate **7a** (40 mg, 0.15 mmol) and TFA (11 µL, 1.4 x 10^{-1} mmol). Purification by silica gel chromatography (Et_2O/petrol containing 1% Et_3N, 5% to 20% as eluent) afforded product as a clear oil (38 mg, 95%). ν_{max} /cm^{-1} (neat) 3030, 2829, 2807, 2775, 1703, 1453; δ_H ($CDCl_3$, 400 MHz) 1.74 (p, J = 6.0 Hz, 2H, NCH_2CH_2), 2.71 – 2.61 (m, 2H, $NCH_2CH_2CH_2$), 2.85 (t, J = 6.1 Hz, 2H, NCH_2CH_2), 3.29 (d, J = 5.4 Hz, 2H, $NCH_2CH{=}CCO_2$), 3.63 (s, 2H, NCH_2Ph), 5.17 (s, 2H, CO_2CH_2Ph), 6.98 (t, J = 5.6 Hz, 1H, C*H*=C)), 7.42 – 7.19 (m, 10H, CH_{Ar}). δ_C ($CDCl_3$, 101 MHz) 25.6 (NCH_2CH_2) 26.4 $NCH_2CH_2CH_2$), 53.1 ($NCH_2CH{=}C$), 57.6 (NCH_2CH_2), 61.4 (NCH_2Ph), 66.6 (CO_2CH_2Ph), 127.2 (CH_{Ar}), 128.9 - 128.2 (4 x CH_{Ar}), 136.4 (CH_{Ar}), 136.5 (CH_{Ar}), 139.0 (CH=*C*qCO_2) 141.2 (*C*H=CCO_2), 167.8 (CO_2Bn). HRMS (ESI$^+$) m/z: $[M+H]^+$ Calcd for $C_{21}H_{23}NO_2$ 322.1807; found 322.1808.

Compound (±)-8b: 7-methyl-2,3,4,7-tetrahydro-N-benzyl-azepane-O-benzyl-5-carboxylate.

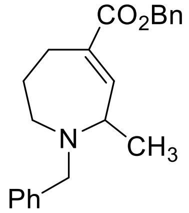

Following general procedure 1, substrate **7b (**79 mg, 0.24 mmol) was heated and stirred at 40 °C for 17 h in DCM. Purification by silica gel chromatography (Et_2O/petrol, 1:19 to 1:4 as eluent) afforded the title compound (63 mg, 80%) as a clear oil. ν_{max} /cm^{-1} (neat) 3031, 2927, 2849, 1703, 1495, 1369; δ_H ($CDCl_3$, 400 MHz) 1.31 (d, J = 7.1 Hz, Me), 1.43 - 1.58 (1H, m , NCH_2CHH), 1.62 - 1.77 (m, 1H, NCH_2CHH), 2.57 (1H, t, J = 12.0 Hz, NCH_2CH_2CHH) 2.80 – 2.90 (2H, m , NCH*H* and NCH_2CH_2CHH), 3.00 (1H, td, J = 14.0, 4.1 Hz, NCH*H*) 3.56 (1H, d, J = 13.8 Hz, NCH*H*Ph), 3.68 (1H, d, J = 13.8 Hz, NCH*H*Ph), 3.77 (1H, pent, J = 7.1 Hz, $NCHCH_3$), 5.18 (2H, AB q, CO_2CH_2Ph), 6.86 (1H, d, J = 6.0 Hz, C*H*=CCO_2), 7.18 – 7.41 (10H, 10 x CH_{Ar}). δ_C ($CDCl_3$, 101 MHz) 17.7 (Me), 21.3 (NCH_2CH_2), 26.5 ($NCH_2CH_2CH_2$), 52.1 (NCH_2), 53.4 (NCH_2Ph), 56.9 ($NCHCH_3$), 66.7 (CO_2CH_2Ph), 126.9 (CH_{Ar}), 128.3 – 128.7 (4 x CH_{Ar}), 134.2 (Cq_{Ar}), 136.4 (Cq_{Ar}), 140.2 R(CH=*C*CO_2), 147.1 (*C*H=$CHCO_2$), 167.9 (*C*O_2Bn). HRMS (ESI$^+$) m/z: $[M+H]^+$ Calcd for $C_{22}H_{25}NO_2$ 336.1964; found 336.1962.

Compound (±)-8c: 7-benzyl-2,3,4,7-tetrahydro-N-(4-methoxybenzyl)-azepane-O-(4-methoxybenzyl)-5-carboxylate.

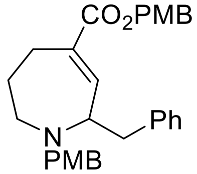

Following general procedure 1, substrate **(**60 mg, 0.13 mmol) was heated and stirred at 40 °C for 3 days in DCM. Purification by silica gel chromatography (Et_2O/petrol, 1:9 to 3:7 as eluent) afforded product as a clear oil (25.6 mg, 43%). ν_{max} /cm^{-1} (neat) 2930, 2835, 1703, 1612, 1511, 1453, 1245. δ_H ($CDCl_3$, 400 MHz) δ 1.38 – 1.47 (1H, m, NCH_2CHH), 1.70 (1H, q, J = 12.1 Hz, NCH_2CHH), 2.53 (1H, t, J = 12.1 Hz, NCH_2CH_2CHH), 2.79 – 2.93 (3H, m, $NCHHCH_2$ and CHCH*H* and NCH_2CH_2CHH), 3.02 – 3.12 (2H, m, $NCHHCH_2$ and CHCH*H*), 3.53 (1H, d, J = 13.6 Hz, NCH*H*Ph), 3.70 (1H, d, J = 13.6 Hz, NCH*H*Ph),

3.78 (3H, s, O*Me*), 3.81 (3H, s, O*Me*), 3.89 (1H, q, J = 7.4 Hz, NCH) 5.10 (2H, s, CO_2CH_2), 6.80 (2H, d, J = 8.0 Hz, 2 x CH_{Ar}), 6.85 – 6.92 (3H, m, C*H*=CCO_2 and 2 x CH_{Ar}), 7.12 (2H, d, J = 7.9 Hz, 2 x CH_{Ar}), 7.17 – 7.32 (7H, m, 7 x CH_{Ar}). δ_C ($CDCl_3$, 101 MHz) 20.4 (NCH_2CH_2), 26.9 ($NCH_2CH_2CH_2$), 39.0 ($CHCH_2Ph$), 52.2 (NCH_2Ph), 52.7 (NCH_2), 55.3 and 55. 4 (2 x O*Me*), 62.9 (NCH), 66.4 (CO_2CH_2), 113.7 `, 114.0 (CH_{Ar}), 126.2 (CH_{Ar}), 128.4 (CH_{Ar}), 129.3 (CH_{Ar}), 129.9 (CH_{Ar}), 131.9 (Cq_{Ar}), 135.4 (CH=CCO_2), 139.5 (Cq_{Ar}), 145.6 (CH=CCO_2), 158.6 (Cq_{Ar}), 159.6 (Cq_{Ar}), 167.8 (CO_2). HRMS (ESI^+) m/z: $[M+H]^+$ Calcd for $C_{30}H_{33}NO_4$ 472.2488; found 472.2492.

Compound (±)-8e: 7-methyl-2,3,4,7-tetrahydro-N-(4-methoxybenzyl)-azepane-O-(4-methoxybenzyl)-5-carboxylate.

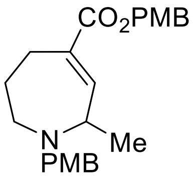

Following general procedure 1, compound **7e** (317 mg, 0.77 mmol) was heated to 40 °C for 15 h in dry DCM. Purification by silica gel chromatography (Et_2O/petrol; 1:9 to 3:7 as eluent) afforded product as a clear oil (240 mg, 76%). δ_H ($CDCl_3$, 400 MHz) 1.28 (3H, d, *J* 7.0 Hz, *Me*), 1.41 – 1.50 (1H, m, CH_2CHHCH_2), 1.55 – 1.73 (1H, m, CH_2CHHCH_2), 2.53 (1H, dd, J 13.4, 10.5, CH*H*-C_q=), 2.77 – 2.86 (2H, m, $NCHHCH_2$ and C*H*H-C_q=), 2.97 (1H, dt, *J* 13.8, 4.3 Hz, $NCHHCH_2$), 3.60 (1H, d, *J* 13.6 Hz, NCH*H*Ar), 3.49 (1H, d, *J* 13.7 Hz, NC*H*HAr), 3.71 – 3.82 (7H, m, 2 × OMe and C*H*Me), 5.06 – 5.15 (2H, AB q, OCH_2Ar), 6.80 – 6.84 (3H, m, =CH and 2 × CH_{Ar}), 6.87 – 6.92 (2H, m, 2 × CH_{Ar}), 7.18 – 7.22 (2H, m, 2 × CH_{Ar}) and 7.29 – 7.34 (2H, m, 2 × CH_{Ar}); δ_C ($CDCl_3$, 101 MHz) 17.6 (CH*Me*), 21.3 ($CH_2CH_2CH_2$), 26.5 (CH_2-C_q=), 52.1 (NCH_2CH_2), 52.7 (NCH_2Ar), 55.4 (OMe), 56.3 (OMe), 66.5 (OCH_2Ph), 113.7 (2 × CH_{Ar}), 114.0 (2 × CH_{Ar}), 128.5 (C_q), 129.8 (2 × CH_{Ar}), 130.1 (2 × CH_{Ar}), 132.2 (C_q), 134.7 (CH=C_q), 147.0 (=CH), 158.6 (C_qOMe), 159.6 (C_qOMe) and 168.0 (CO_2PMB); HRMS (ESI^+) m/z: $[M+H]^+$ calcd for $C_{24}H_{30}NO_4$ 396.2176; found 396.2170.

2 mmol scale ring expansion with reduced (5 mol% Pd) catalyst loading

Following a modified general procedure 1, compound **7e** (884 mg, 2.20 mmol) was heated to 40 °C for 15 h in dry DCM (11 + 18 mL) using $[Pd(allyl)Cl]_2$ (20.5 mg, 0.56 mmol), $P(OEt)_3$ (38 µL, 0.22 mmol), morpholine (78.6 µL, 0.91 mmol) and TFA (172 µL, 2.2 mmol). Purification by silica gel chromatography (Et_2O/petrol; 1:9 to 1:4 to 3:7 as eluent) afforded the product as a clear oil (646 mg, 73%). Spectral data was as reported above.

Compound (±)-8f: 7-methyl-2,3,4,7-tetrahydro-N-(4-methoxybenzyl)-azepane-O-(4-methoxybenzyl)-5-carboxylate amide.

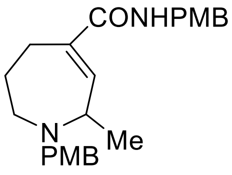

Following general procedure 1, substrate **7e (**57.7 mg, 0.15 mmol) was stirred at 40 °C for 15 h in MeCN. Purification by silica gel chromatography (NH_3/EtOH/DCM, 1:99 to 4:96 as eluent) afforded product as a clear oil (31 mg, 54%). ν_{max}/cm^{-1} (neat) 3315, 2928, 2835, 1651, 1612, 1510, 1463, 1300, 1244. δ_H ($CDCl_3$, 400 MHz) 1.27 (3H, d, J = 7.2 Hz, CH*Me*), 1.42 – 1.51 (1H, m, NCH_2CHH), 1.73 (1H, q, J = 11.5 Hz, NCH_2CHH), 2.53 (1H, t, J = 12.2 Hz, NCH_2CH_2CHH), 2.72 (1H, dd, J = 15.2 Hz, 7.0 Hz, NCH_2CH_2CHH), 2.82 (1H, ddd, J = 14.2, 10.9, 4.1 Hz, NCH*H*), 2.98 (1H, td, J = 14.1, 4.2 Hz, NCH*H*), 3.49 (1H, d, J = 13.4 Hz, NCH*H*Ph), 3.59 (1H, d, J = 13.4 Hz, NCH*H*Ph), 3.69 – 3.83 (7H, m, 2 x ArO*Me* and C*H*Me), 4.35 – 4.47 (2H, m, $CONHCH_2$), 5.97 (1H, t, J = 5.7 Hz, CON*H*), 6.18 (C*H*=CCON), 6.82 (2H, d, J = 8.2 Hz, 2 x CH_{Ar}), 6.87 (2H, d, J = 8.2 Hz, 2 x CH_{Ar}), 7.17 – 7.27 (4H,m, 4 x CH_{Ar}). HRMS (ESI^+) m/z: $[M+H]^+$ Calcd for $C_{24}H_{30}N_2O_3$ 395.2335; found 395.2333.

Compound (±)-8g: 2,3,4,7-tetrahydro-N-(benzyl)-5-phenylazepane.

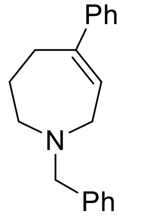

Following general procedure 1, substrate **7f** (40 mg, 0.15 mmol) was heated to 75 °C for 17.5 h in dry MeCN (2.0 mL). Purification by silica gel chromatography (Et_2O in petrol containing 0.5% Et_3N, 5:95 to 1:4 as eluent) afforded product as a clear oil (38 mg, 95%). ν_{max} /cm^{-1} (film) 2970, 2802, 1601, 1493, 1446 and 1365; δ_H ($CDCl_3$, 400 MHz) 1.81 (2H, dd, 5.5, 5.3, NCH_2CH_2), 2.72 – 2.78 (2H, m, $NCH_2CH_2CH_2$), 2.99 (2H, t, J 5.6, NCH_2CH_2), 3.33 (2H, d, J 6.1, NCH_2=CH=), 3.70 (2H, s, NCH_2Ph), 5.93 (1H, t, J 6.0, =CH), 7.20 – 7.28 (2H, m) and 7.28 – 7.38 (8H, m); δ_C ($CDCl_3$, 100 MHz) 25.2 ($NCH_2C\mathit{H_2}$), 32.2 ($NCH_2CH_2C\mathit{H_2}$), 52.8 (NCH_2=CH=), 58.9 ($NC\mathit{H_2}CH_2$), 60.3 (NCH_2Ph), 125.9 (2 × CH_{Ar}), 126.8 (CH_{Ar}), 127.0 (=*C*H), 127.0 (CH_{Ar}), 128.3 (2 × CH_{Ar}), 128.3 (2 × CH_{Ar}), 129.1 (2 × CH_{Ar}), 139.3 (C_q=CH), 144.3 (C_q) and 146.4 (C_q); HRMS (ESI^+) m/z: $[M+H]^+$ Calcd for $C_{19}H_{21}N$ 264.1752; found 264.1750.

Compound (±)-8h: 7-methyl-2,3,4,7-tetrahydro-N-(benzyl)-5-phenylazepane.

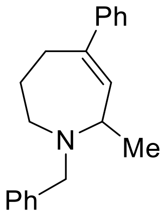

Following general procedure 1, substrate *Z*-**7g** (57 mg, 0.2 mmol) was heated and stirred at 80 °C for 15 h in MeCN. Purification by silica gel chromatography (EtOAc/petrol containing 2% Et_3N, 1:9 to 3:7 as eluent) afforded product as a clear oil (40 mg, 70%). ν_{max} /cm^{-1} (neat) 3024, 2922, 2845, 1599, 1493, 1451. δ_H ($CDCl_3$, 400 MHz) 1.35 – 1.49 (4H, m, CH*Me* and $NCH_2CH\mathit{H}$), 1.94 (1H, q, J = 12.1 Hz, $NCH_2CH\mathit{H}$), 2.77 (1H, dd, J = 14.8, 6.6 Hz, =C_qPhCH*H*), 2.83 – 2.93 (1H, m, =C_qPhCH*H*), 3.00 (1H, t, J = 12.9 Hz, NCH*H*), 3.11 (1H, dt, J = 14.3, 8.3 Hz, NCH*H*), 3.60 (1H, d, J = 13.8 Hz, NCH*H*Ph), 3.74 (1H, d, J = 13.8 Hz, NCH*H*Ph), 3.94 (1H, p, J = 6.9 Hz, NC*H*Me), 5.79 (1H, d, J = 5.7 Hz, C*H*=C_qPh), 7.19 – 7.47 (10H, m, 10 x $C\mathit{H}_{Ar}$). δ_C ($CDCl_3$, 101 MHz) 19.8 ($NCH_2\mathit{C}H_2$), 20.3 (CH*Me*), 32.5 (C_qPh*C*H_2), 50.7 (N*C*H_2Ph), 53.6 (N*C*H_2), 56.5 (NC*H*Me), 125.9 ($\mathit{C}H_{Ar}$), 126.8 and 126.9 (2 x $\mathit{C}H_{Ar}$), 128.3 and 128.4 and 128.9 (3 x $\mathit{C}H_{Ar}$), 133.6 (*C*H=C_qPh), 140.9 (CH=C_qPh), 144.0 and 144.1 (2 x C_{qAr}). HRMS (ESI^+) m/z: $[M+H]^+$ Calcd for $C_{20}H_{23}N$ 278.1909; found 278.1906.

Compound (±)-8i: 7-methyl-2,3,4,7-tetrahydro-N-(benzyl)-5-phenylazepine.

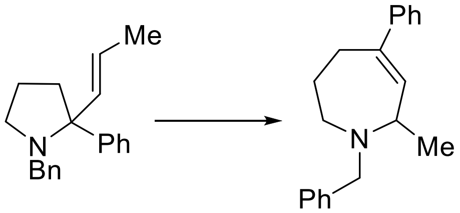

A flame dried Schlenk tube underwent three vacuum/argon cycles. To this tube $[Pd(allyl)Cl]_2$ (2.8 mg, 7.5 × 10^{-3} mmol) was added followed by one more vacuum/argon cycle before adding dry MeCN (0.7 mL) with stirring. $P(OEt)_3$ (5 µL, 2.9 × 10^{-2} mmol) was added followed by morpholine (5 µL, 5.7 × 10^{-2} mmol). The mixture was stirred at rt for 5 min until a yellow solution had formed. 400 µL (~60%) of the catalyst mixture was removed. Substrate ***E*-7g** (17 mg, 0.061 mmol) was dissolved in dry MeCN (1 mL) under argon and added to the Schlenk tube. TFA (4.7 µL, 0.061 mmol) was added. The mixture was heated to 80 °C. after 15 h the reaction was allowed to cool to rt. Standard work up gave a dark oil. ^{1}H NMR yield using DMF as internal standard showed a 66% yield.

Compound (±)-8i: 7-methyl-2,3,4,7-tetrahydro-N-(methyl)-5-phenylazepane.

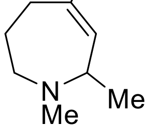

Following general procedure 1, substrate **7h** (44.2 mg, 0.22 mmol) was heated to 80 °C in MeCN for 15 h. Purification by silica gel chromatography ($EtOH/NH_3$/DCM + 0.5% Et_3N, 1:99 to 5:95 as eluent) afforded the product as a yellow oil (30 mg. 68%). ν_{max} /cm^{-1} (neat) 3022, 2959, 2926, 2842, 2793, 1598, 1492, 1445; δ_H ($CDCl_3$, 400 MHz) 1.28 (3H, d, J = 6.9 Hz, CH*Me*), 1.41 – 1.51 (1H, m, $NCH_2CH\mathit{H}$), 1.84 – 1.97 (1H, m, $NCH_2CH\mathit{H}$), 2.34 (3H, s, N*Me*), 2.65 – 2.87 (2H, m, =$C_qC\mathit{H_2}$), 3.15 – 3.22 (2H, m, $NC\mathit{H_2}$), 3.79 (1H, p, J = 6.8 Hz, C*H*=C_qPh), 7.18 – 7.36 (5H, m, 5 x $C\mathit{H}_{Ar}$); δ_C ($CDCl_3$, 101 MHz) 19.9 (CH*Me*), 20.6 ($NCH_2\mathit{C}H_2$), 32.5 (C_q(Ph)*C*H_2), 35.4 (N*Me*), 56.7 (N*C*HMe), 59.2 (N*C*H_2CH_2), 125.9 ($\mathit{C}H_{Ar}$), 126.8 ($\mathit{C}H_{Ar}$), 128.3 ($\mathit{C}H_{Ar}$), 133.4 (*C*H=C_qPh), 144.1 (C_{qAr}), 144.5 (CH=C_qPh); HRMS (ESI^+) m/z: $[M+H]^+$ Calcd for $C_{14}H_{19}N$ 202.1597; found 202.1593.

Compound (±)-8j: 7-isobutyl-2,3,4,7-tetrahydro-N-(benzyl)-5-phenylazepine.

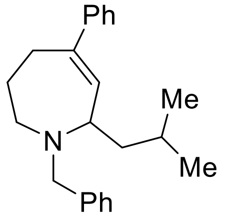

Following general procedure 1, Substrate **7i** (58 mg, 0.18 mmol) was heated to 80 ˚C for 16.5 h in MeCN. Purification by silica gel chromatography (Et_2O/petrol, 1:99 to 1:4 as eluent) afforded the product as a clear oil (30 mg, 52%). ν_{max} /cm^{-1} (film): 3050, 2952, 2923, 1452 and 1365; δ_H ($CDCl_3$, 400 MHz) 0.94 (3H, d, J 7.4, CHMe*Me*), 0.96 (3H, d, J 7.5, CH*Me*Me), 1.28 – 1.38 (1H, m, NCH_2CH*H*), 1.47 (1H, dd, J 14.0, 7.1, NCHCH*H*), 1.70 (1H, dt, J 14.3, 7.2, NCHC*H*H), 1.80 – 1.97 (2H, m, C*H*Me_2 and NCH_2C*H*H), 2.76 (1H, dd, J 14.7, 6.3, NCH_2CH_2CH*H*), 2.86 (1H, app. t, J 13.3, NCH_2CH_2C*H*H), 2.97 (1H, dd, J 14.6, 12.1, NCH*H*CH_2), 3.13 (1H, d, J 14.6, NC*H*HCH_2), 3.57 (1H, d, J 13.8, NCH*H*Ph), 3.69 (1H, d, J 14.0, NC*H*HPh), 3.79 (1H, q, J 7.2, NC*H*), 5.76 (1H, d, J 5.8, =CH), 7.18 – 7.36 (8H, m) and 7.39 (2H, d, J 7.6); δ_C ($CDCl_3$, 101 MHz) 19.1 (NCH_2*C*H_2), 22.8 (Me), 23.1 (Me), 25.1 (*C*HMe_2), 32.8 (NCH_2CH_2*C*H_2), 43.7 (NCH*C*H_2), 50.0 (N*C*H_2Ph), 54.4 (N*C*H_2CH_2), 58.6 (N*C*H), 125.9 (2 × CH_{Ar}), 126.7 (CH_{Ar}), 126.9 (CH_{Ar}), 128.3 (2 × CH_{Ar}), 128.3 (2 × CH_{Ar}), 128.9 (2 × CH_{Ar}), 133.3 (=*C*H), 141.0 (*C*$_q$=CH), 144.1 (C_q) and 144.5 (C_q); HRMS (ESI$^+$) m/z: $[M+H]^+$ Calcd for $C_{23}H_{29}N$ 320.2378; found 320.2374.

Compound (±)-8k: 6-methyl-2,3,4,7-tetrahydro-N-(benzyl)-5-phenylazepine.

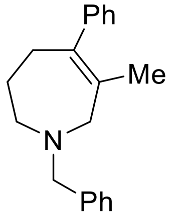

Following general procedure 1, Substrate **7i** (58 mg, 0.18 mmol) was heated to 80 ˚C for 16.5 h in MeCN. Purification by silica gel chromatography (Et_2O/petrol, 5:95 to 4:6 as eluent) afforded the title compound (30 mg, 40%) as a pale yellow semi-solid. ν_{max} /cm^{-1} (film) 2922, 1599, 1492, 1453 and 1440; δ_H ($CDCl_3$, 400 MHz) 1.55 (3H, s, Me), 1.79 (2H, p, *J* 5.7 Hz, NCH_2C*H*$_2$), 2.54 – 2.64 (2H, m, NCH_2CH_2C*H*$_2$), 2.94 (2H, t, J 5.5, NC*H*$_2CH_2$), 3.32 (2H, s, NC*H*2-CMe), 3.73 (2H, s, NC*H*$_2$Ph), 7.11 – 7.41 (10H, m, 2 × Ph); δ_C ($CDCl_3$, 101 MHz) 22.1 (Me), 26.0 (NCH_2*C*H_2), 35.3 (NCH_2CH_2*C*H_2), 58.7 (N*C*H_2CH_2), 59.5 (N*C*H_2CMe), 60.6 (N*C*H_2Ph), 126.0 (1 × CH_{Ar}), 127.1 (1 × CH_{Ar}), 128.1 (2 × CH_{Ar}), 128.2 (2 × CH_{Ar}), 128.3 (2 × CH_{Ar}), 129.1 (2 × CH_{Ar}), 132.4 (C_q), 139.3 (C_q), 139.8 (C_q) and 145.3 (C_q); HRMS (ESI$^+$) m/z: $[M+H]^+$ calcd for $C_{21}H_{26}N$ 292.2067; found 292.2059.

Compound (±)-8l: 7-methyl-2,3,4,7-tetrahydro-N-(5-methyl-2-furyl)-azepane-O-methyl-5-carboxylate.

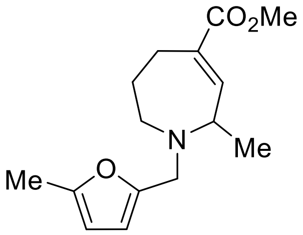

Following general procedure 1, substrate **7k (**31 mg, 1.2 x 10^{-1} mmol) was heated and stirred at 80 ˚C for 15 h in MeCN. General work up gave a yellow oil. Purification by silica gel chromatography (Et_2O/petrol, 1:9 to 1:1 as eluent) afforded product as a clear oil (17.6 mg, 57%). ν_{max} /cm^{-1} (neat) 2925, 2850, 1708, 1567, 1435, 1369, 1255. δ_H ($CDCl_3$, 400 MHz) 1.30 (3H, d, J = 7.0 Hz, CH*Me*), 1.39 – 1.50 (1H, m, NCH_2CH*H*), 1.68 (1H, q, J = 12.8 Hz, NCH_2CH*H*), 2.25 (3H, s, Ar*Me*), 2.50 (1H, dd, J = 15.4. 10.9 Hz, NCH_2CH_2CH*H*), 2.82 (1H, ddd, J = 15.6, 7.6, 2.4 Hz, NCH_2CH_2CH*H*), 2.94 (1H, ddd, 14.3, 10.6, 3.6 Hz, NCH*H*), 3.14 (1H, dt, J = 14.1, 4.1 Hz, NCH*H*), 3.55 (1H, d, J =14.2 Hz, NCH*H*Ar), 3.64 (1H, d, J =14.2 Hz, NCH*H*Ar), 3.73 (3H, s, CO_2*Me*), 3.81 (1H, p, J = 7.0 Hz, C*H*Me), 5.84 (1H, d, J = 2.9 Hz, C*H*$_{Ar}$), 6.03 (1H, d, J = 3.0 Hz, C*H*$_{Ar}$), 6.77 (1H, d, J = 5.8 Hz, C*H*=CCO_2). δ_C ($CDCl_3$, 101 MHz) 13.8 (Ar*Me*), 18.5 (CH*Me*), 20.3 (NCH_2*C*H_2), 26.7 (NCH_2CH_2*C*H_2), 45.3 (N*C*H_2Ar), 52.1 (CO_2*Me*), 53.2 (N*C*H_2), 56.0 (*C*HMe), 107.0 (*C*H_{Ar}), 109.1 (*C*H_{Ar}), 135.1 (CH=*C*CO_2), 146.5 (*C*H=CCO_2), 151.1 (Cq_{Ar}), 151.9 (Cq_{Ar}), 168.4 (*C*O_2Me). HRMS (ESI$^+$) m/z: $[M+H]^+$ Calcd for $C_{15}H_{21}NO_3$ 264.1600; found 264.1595.

Compound (±)-8m: 7-methyl-2,3,4,7-tetrahydro-N-(2-pyridyl)-azepane-O-methyl-5-carboxylate.

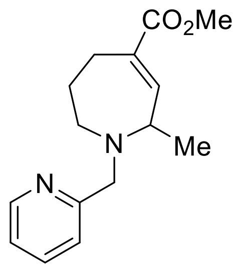

Following general procedure 1, substrate **7l (**49 mg, 0.19 mmol) was heated and stirred at 45 °C for 15 h in DCM. Purification by silica gel chromatography (NH_3.EtOH/DCM, 1:99 to 5:95 as eluent) afforded product as a clear oil (24 mg, 49%). ***(51)*** ν_{max} /cm^{-1} (neat) 2929, 2851, 1706, 1650, 1589, 1433, 12588. δ_H ($CDCl_3$, 400 MHz) 1.30 (3H, d, J = 7.3Hz, *Me*), 1.42 – 1.53 (1H, m, NCH_2CH*H*), 1.65 – 1.79 (1H, m, NCH_2CH*H*), 2.54 (1H, t, J = 12.0 Hz, NCH_2CH_2CH*H*), 2.78 – 2.97 (2H, m, NCH*H* and NCH_2CH_2CH*H*), 3.04 (1H, dt, J = 13.9, 4.3 Hz, NCH*H*), 3.73 (3H, s, CO_2*Me*), 3.74 – 3.84 (3H, m, NCH_2Pyr and NC*H*Me), 6.8 (1H, d, J = 5.7 Hz, C*H*=CCO_2), 7.11 (1H, t, J = 6.8 Hz, CH_{Ar}), 7.43 (1H, d, J = 7.8 Hz, CH_{Ar}), 7.62 (1H, t, J = 7.6 Hz, CH_{Ar}), 8.49 (1H, d, J = 4.8 Hz, CH_{Ar}). δ_C ($CDCl_3$, 101 MHz) 18.0 (*Me*), 21.5 (NCH_2CH_2), 26.5 ($NCH_2CH_2CH_2$), 52.0 (CO_2*Me*), 53.2 (NCH_2), 55.5 (NCH_2Pyr), 56.8 (NCHMe), 121.9 (CH_{Ar}), 122.7 (CH_{Ar}), 134.9 (CH=CCO_2), 136.6 (CH_{Ar}), 146.7 (CH=CCO_2), 149.2 (CH_{Ar}), 160.9 (Cq_{Ar}), 168.5 (CO_2Me). HRMS (ESI$^+$) m/z: $[M+H]^+$ Calcd for $C_{15}H_{20}N_2O_2$ 261.160303; found 261.1601.

9.9.2 Compound (±)-8n: 2,3,4,7-tetrahydro-N-(2-bromobenzyl)-azepane-O-methyl-5-carboxylate.

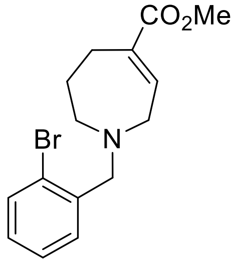

Following general procedure 1, substrate **7m** (42 mg, 0.13 mmol) was heated to 70 °C in MeCN for 15 h. Purification by silica gel chromatography (Et_2O/petrol, 1:99 to 1:9 as eluent) afforded product as a clear oil (35mg, 83%). ν_{max} /cm^{-1} (neat) 2929, 2844, 2806, 2774, 17-6, 1434, 1261, 1225; δ_H ($CDCl_3$, 400 MHz) 1.75 (2H, p, J = 5.6 Hz, NCH_2CH_2), 2.65 – 2.68 (2H, m, =C_qCH_2), 2.89 (2H, t, J = 6.9 Hz, NCH_2CH=), 3.32 (1H, d, J = 6.0 Hz, NCH_2CH_2), 3.69 (2H, s, NCH_2Ph), 3.72 (CO_2*Me*), 6.93 (1H, t, J = 5.8 Hz, C*H*=CCO_2), 7.10 (1H, t, J = 8.1 Hz, CH_{Ar}), 7.26 (1H, t, J = 6.7 Hz, CH_{Ar}), 7.45 (1H, d, J = 7.2 Hz, CH_{Ar}), 7.51 (1H, d, J = 7.2 Hz, CH_{Ar}); δ_C ($CDCl_3$, 101 MHz) 25.8 (NCH_2CH_2), 26.2 ($C_q(CO_2)CH_2$), 52.0 (CO_2*Me*), 53.2 (NCH_2CH=), 57.6 (NCH_2CH_2), 60.3 (NCH_2Ph), 124.4 (C_{qAr}), 127.5 (CH_{Ar}), 128.5 (CH_{Ar}), 130.5 (CH_{Ar}), 132.8 (CH_{Ar}), 136.5 (=C_qCO_2), 138.5 (C_{qAr}), 140.8 (CH=CCO_2), 168.5 (CO_2Me); HRMS (ESI$^+$) m/z: $[M+H]^+$ Calcd for $C_{15}H_{19}NO_2Br$ 324.0599; found 324.0591.

Compound 8o.

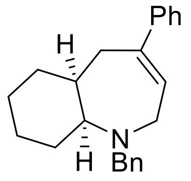

Following general procedure 1, substrate **7n** (52.8 mg, 0.17 mmol) was heated to 80 °C for 16 h in MeCN. Purification by silica gel chromatography (Et_2O/petrol, 1:9 to 1:4 as eluent) afforded the product as a clear oil (38 mg, 72%). ν_{max} /cm^{-1} (neat) 3081, 3058, 3025, 2854, 2796, 1598, 1493, 1446; δ_H ($CDCl_3$, 400 MHz) 1.18 – 1.52 (4H, m, $CH_{2(cyclohexyl)}$ and 2 x $CHH_{(cyclohexyl)}$), 1.62 – 1.98 (4H, m, $CH_{2(cyclohexyl)}$ and 2 x $CHH_{(cyclohexyl)}$), 2.22 (1H, d, J = 15.6 Hz, C_qPhCH*H*), 2.45 (1H, dq, 11.5 Hz, J = 11.5, 3.9 Hz, NCHC*H*), 2.76 (1H, dt, J = 11.6, 3.6 Hz, NC*H*CH), 3.16 (1H, dd, J = 15.9, 5.3 Hz, NCH*H*), 3.26 (1H, t, J = 13.4 Hz, C_qPhCH*H*), 3.50 (1H, dd, 16.1, 6.4 Hz, NCH*H*), 3.73 (1H, d, J = 14.1 Hz, NCH*H*Ph), 3.83 (1H, d, J = 14.1 Hz, NCH*H*Ph), 5.87 (1H, t, J = 6.1 Hz, C*H*=C_qPh), 7.19 – 7.40 (10H, m, 10 x CH_{Ar}); δ_C ($CDCl_3$, 100 MHz) 21.4 ($CH_{2(cyclohexyl)}$), 26.3 ($CH_{2(cyclohexyl)}$), 27.2 ($CH_{2(cyclohexyl)}$), 33.0 (NCHCH), 33.2 (C_qPhCH_2), 34.1 ($CH_{2(cyclohexyl)}$), 46.8 (NCH_2), 57.7 (NCH_2Ph), 63.9 (NCH), 125.9 (CH_{Ar}), 126.8, 128.3 (CH_{Ar}), 128.8 (CH_{Ar}), 140.3 (CH=C_qPh), 144.0 (C_{qAr}), 144.9 (C_{qAr}); HRMS (ESI$^+$) m/z: $[M+H]^+$ Calcd for $C_{23}N_{20}N$ 318.2222; found 318.2212.

Compound 8p.

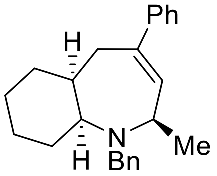

Following general procedure 1, substrate **7o** (56 mg, 0.17 mmol) was heated to 80 °C in DCE for 63 h. Purification by silica gel chromatography (Et_2O/petrol, 0.5:99.5 to 1:9 as eluent) afforded the title compound as a clear oil (18.4 mg, 33%, d.r. = 9:1). $[\alpha]^{20}_D$ = -108.4 (*c* 0.19, DCM). ν_{max} /cm^{-1} (neat) 3061, 3025, 2928, 2856, 1492, 1481; δ_H ($CDCl_3$, 400 MHz) 1.13 – 1.38 (7H, m, CH*Me* and $CH_{2(cyclohexyl)}$ 2 x $CHH_{(cyclohexyl)}$), 1.49 (1H, d, J = 12.8 Hz, NCHCH*H*), 1.66 (1H, d, J = 8.0 Hz, $CHCHCHH_{(cyclohexyl)}$), 1.75 (1H, d, J = 12.5 Hz, $NCHCH_2CHH$), 2.11 (1H, q, J = 12.0 Hz, NC_qCHH), 2.23 (1H, d, J = 14.9 Hz, $=C_qCHH$), 2.34 (1H, d, J = 12.1 Hz, NCHC*H*), 2.67 (1H, dt, J = 12.3, 3.5 Hz, NC*H*), 3.27 (1H, t, J = 13.3 Hz, $=C_qCHH$), 3.59 (1H, d, J = 15.3 Hz, NCH*H*Ph), 3.59 (1H, d, J = 15.3 Hz, NCH*H*Ph), 4.05 (1H, p, J = 6.8 Hz, C*H*Me), 5.75 (1H, d, J = 5.7 Hz, $CH=C_qPh$), 7.12 – 7.42 (10H, m, 10 x CH_{Ar}); δ_C ($CDCl_3$, 100 MHz) 21.3 (CH*Me*), 21.6 ($CH_{2(cyclohexyl)}$), 26.9 ($CH_{2(cyclohexyl)}$), 27.3 (NCH*C*H), 27.7 ($CH_{2(cyclohexyl)}$), 33.1 ($=C_qCH_2$), 48.2 (*C*HMe), 50.0 (NCH_2Ph), 60.1 (N*C*H), 125.8 (CH_{Ar}), 126.4 (CH_{Ar}), 126.9 (CH_{Ar}), 128.2 (CH_{Ar}), 128.4 (CH_{Ar}), 142.2 (C_{qAr}), 143.5 ($=C_qPh$), 144.4 (C_{qAr}); HRMS (ESI^+) m/z: $[M+H]^+$ calcd for $C_{24}H_{29}N$ 332.238; found 332.2377. Stereochemistry was assigned via NMR spectroscopy as shown in S81-85.

Compound (±)-8q: 2,3,4,5,8-pentahydro-N-(benzyl)-6-phenyl-azacylco-oct-6-ene.

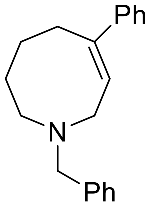

58

Following general procedure 1, substrate **7p** (78 mg, 0.28 mmol) was heated to 80 °C in MeCN for 15 h. Purification by silica gel chromatography ($EtOH/NH_3$:DCM, 1:99 to 5:95 as eluent) afforded the title compound as a light-yellow oil. (53 mg, 68%) ***(58)*** ν_{max} /cm^{-1} (film) 2919, 2803, 1598, 1493, 1451 and 1347; δ_H ($CDCl_3$, 400 MHz) 1.67 – 1.80 (4H, m, 2 × CH_2), 2.70 – 2.75 (2H, m, NCH_2CH_2), 2.87 (2H, t, J 5.5, $=C_q-CH_2$), 3.32 (2H, d, J 7.1, NCH_2-CH=), 3.66 (2H, s, NCH_2Ph), 5.93 (1H, t, J 7.0, =C*H*) and 7.21 – 7.42 (10H, m); δ_C ($CDCl_3$, 100 MHz) 24.3 (CH_2), 28.4 (CH_2), 29.6 ($CH_2-C_q=$), 51.3 (NCH_2-CH=), 54.6 (NCH_2CH_2), 61.2 (NCH_2Ph), 124.5 (=*C*H), 126.0 (2 × CH_{Ar}), 126.9 (CH_{Ar}), 127.0 (CH_{Ar}), 128.3 (2 × CH_{Ar}), 128.4 (2 × CH_{Ar}), 129.1(2 × CH_{Ar}), 139.8 (C_q=CH), 142.6 (C_q) and 143.9 (C_q); HRMS (ESI^+) m/z: $[M+H]^+$ Calcd for $C_{20}H_{23}N$ 278.1909; found 278.1906.

Compound (±)-8r: 8-methyl-2,3,4,5,8-pentahydro-N-(benzyl)-6-phenyl-azacylco-oct-6-ene.

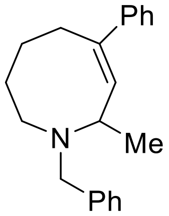

Following general procedure 1, substrate **7q** (41.8 mg, 0.14 mmol) was heated to 80 °C for 16 h in MeCN. Purification by silica gel chromatography (Et_2O/petrol, 1:99 to 1:1 as eluent) afforded the product as a clear oil (8.4 mg, 20%). ν_{max} /cm^{-1} (neat); δ_H ($CDCl_3$, 400 MHz); 1.37 (3H, d, J = 6.1 Hz, CH*Me*), 1.46 – 1.63 (2H, m, $NCH_2CH_2CH_2$), 1.79-1.90 (2H, m, NCH_2CH_2), 2.53 – 2.66 (2H, m, NCH*H* and CPhCH*H*), 2.71 – 2.81 (1H, m, CPhCH*H*), 3.07 (1H, t, J = 12.0 Hz, NCH*H*), 3.50 91H, d, J = 13.3 Hz, NCH*H*Ph), 3.79 – 3.94 (2H, m, NCH*H*Ph and NC*H*Me), 5.75 (1H, J = 8.0 Hz, C*H*=CPh), 7.13 – 7.46 (10H, m, 10 × CH_{Ar}); δ_C ($CDCl_3$, 100 MHz); 19.1 (CH*Me*), 22.9 (NCH_2 CH_2CH_2),, 28.7 (NCH_2CH_2), 29.0 (C_qPhCH_2), 51.4 (N*C*HCH=), 53.1 (NCH_2), 65.9 (NCH_2Ph), 126.2 ($CH=C_qPh$), 126.8 and 126.9 (2 ×CH_{Ar}), 128.3 and 128.4 (2 ×CH_{Ar}), 129.0 (2 ×CH_{Ar}), 140.6 ($CH=C_qPh$), 143.7 (C_{qAr}); HRMS (ESI^+) m/z: $[M+H]^+$ Calcd for $C_{21}H_{26}N$ 292.2067 ; found 292.2059.

Compound (*S*)-8b: 7-methyl-2,3,4,7-tetrahydro-N-benzyl-azepane-O-benzyl-5-carboxylate.

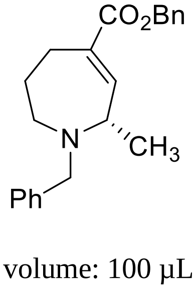

Following general procedure 1, substrate (*R*)-**7b (**80 mg, 0.25 mmol) was heated and stirred at 40 °C for 15 h in DCM. Purification by silica gel chromatography (Et_2O/petrol, 1:9 as eluent) to afford the title compound as clear oil (63 mg, 79%). $[\alpha]^{20}_D$ = -8.6 (*c* 0.224, DCM). Analytical data was as reported above. Samples suitable for XRD were produced by slow evaporation from petrol. The compound was analysed by HPLC under the following conditions: Mobile phase: 98 % Water + 1 % acetic acid, 2 % acetonitrile + 1 % acetic acid. Flow rate: 0.75 mL/min. Injection volume: 100 µL. Detector: 254 nm. Column: (R,R)-WHELK-01, 30 cm. Column temperature: 26 °C.

Racemic standard of **8b**.

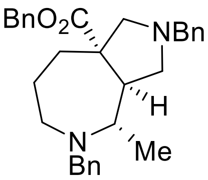

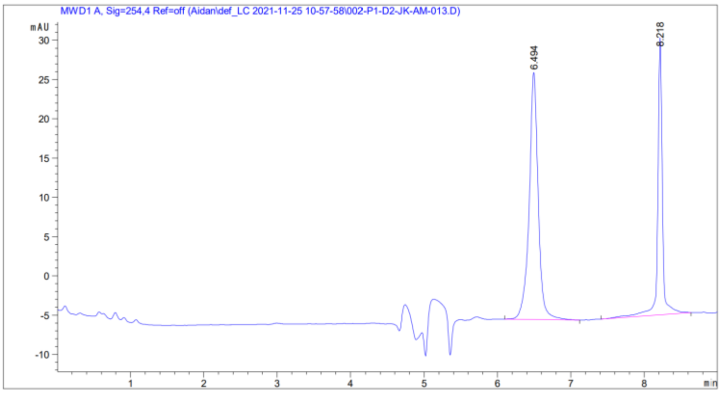

Signal 1: MWD1 A, Sig=254,4 Ref=off

Peak #	RetTime [min]	Type	Width [min]	Area [mAU*s]	Height [mAU]	Area %
1	6.494	BB	0.1265	271.20667	31.49378	63.3991
2	8.218	BB	0.0658	156.57019	35.29240	36.6009
Totals :				427.77686	66.78618	

Enantio-enriched **8b** from the above reaction.

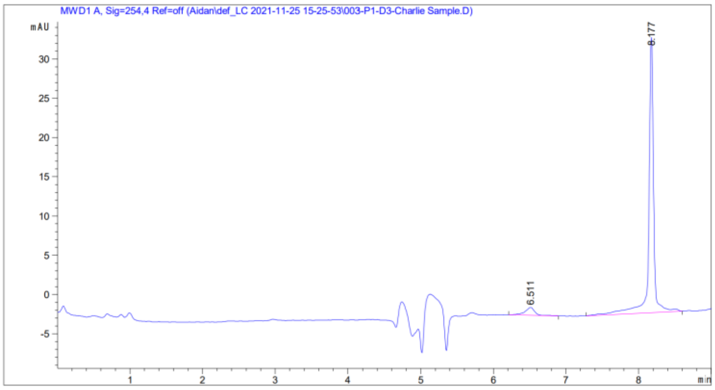

Signal 1: MWD1 A, Sig=254,4 Ref=off

Peak #	RetTime [min]	Type	Width [min]	Area [mAU*s]	Height [mAU]	Area %
1	6.511	BB	0.1291	9.07326	9.88302e-1	5.2562
2	8.177	BV R	0.0684	163.54776	35.08807	94.7438
Totals :				172.62102	36.07637	

Compound (*R*)-8b: 7-methyl-2,3,4,7-tetrahydro-N-benzyl-azepane-O-benzyl-5-carboxylate

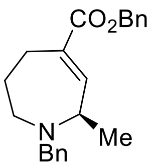

Following general procedure 1, the substrate (*R*)-**7s** (86.8 mg, 0.26 mmol) was heated at 40 °C in DCM for 16 h. Purification of crude by silica gel chromatography (Et_2O/Petrol, 1:9 to 3:7 as eluent) afforded the title compound as a clear oil (66 mg, 76%). $[\alpha]^{20}_{D}$ = +8.3 (*c* 0.124, DCM). All other spectral data was as given above.

3.11 Derivatisation reactions.

Compound (±)-16a

To a stirred solution of compound **8b** (40 mg, 0.12 mmol) and *N*-(methoxymethyl)-*N*-(trimethylsilylmethyl)benzylamine **15** (100 µL, 90% purity, 0.35 mmol) in DCM (3 mL) at 0 °C under nitrogen was added TFA (10 µL, 0.13 mmol) and the reaction allowed to warm to rt overnight. After 22 h further *N*-(methoxymethyl)-*N*-(trimethylsilylmethyl)benzylamine (50 µL, 90% purity, 0.18 mmol) was added and the reaction stirred for a further 14 h. The mixture was diluted with DCM (15 mL) and

washed with sat. aq. $NaHCO_3$ (15 mL). The aqueous phase was extracted with DCM (15 mL) and the combined organic phase dried ($MgSO_4$) and evaporated to give a yellow oil. Purification by silica gel chromatography (Et_2O/petrol, 15:85 to 3:7 as eluent) afforded the product as a clear oil (50 mg, 87%). ν_{max} /cm^{-1} (film) 2926, 1728, 1494, 1454, 1364 and 1190; $\underline{\delta_H}$ ($CDCl_3$, 400 MHz) 1.15 (3H, d, *J* 6.2, Me), 1.27-1.35 (1H, m, CH_2CHHCH_2), 1.49 (1H, ddd, *J* 14.3, 13.8, 10.2, CH_2C*H*HCH_2), 1.69 (1H, app. t, J 12.9, NCH_2CH_2CH*H*), 1.88 (1H, t, J 9.3, NCH*H*CH), 2.02 (1H, d, J 9.6, NCH*H*C_q), 2.18 (1H, dd, J 14.2, 6.4, NCH_2CH_2C*H*H), 2.51 (1H, ddd, J 14.5, 11.0, 3.0, NCH*H*CH_2), 2.78 (1H, dt, J 14.5, 3.7, NC*H*HCH_2), 2.98 (1H, dq, J 10.9, 6.4, C*H*Me), 3.08 (1H, t, J 8.3, NC*H*HCH), 3.31 (1H, dt, J 10.2, 8.0, NCHC*H*), 3.34 (1H, d, J 9.5, NC*H*HC_q), 3.39 (1H, d, J 13.2, NCH*H*Ph), 3.49 (1H, d, J 14.3, NCH*H*Ph), 3.59 (1H, d, J 13.2, NCH*H*Ph), 3.80 (1H, d, J 14.4, NC*H*HPh), 5.15 – 5.26 (2H, AB-q, OCH_2), 7.15 – 7.29 (10H, m, 10 × CH_{Ar}) and 7.31 – 7.36 (5H, m, 5 × CH_{Ar}); δ_C ($CDCl_3$, 101 MHz 20.6 (Me), 22.6 (CH_2*C*H_2CH_2), 33.9 (NCH_2CH_2*C*H_2), 46.7 (NCH*C*H), 48.9 (N*C*H_2Ph), 52.2 (N*C*H_2CH_2), 56.5 (C_q), 59.2 (N*C*HMe), 59.8 (N*C*H_2Ph), 61.0 (N*C*H_2CH), 66.9 (O*C*H_2), 67.3 (N*C*H_2C_q), 126.5 (1 × CH_{Ar}), 127.0 (1 × CH_{Ar}), 128.1 (2 × CH_{Ar}), 128.2 (1 × CH_{Ar}), 128.3 (4 × CH_{Ar}), 128.58 (2 × CH_{Ar}), 128.63 (4 × CH_{Ar}), 136.4 (C_q), 138.8 (C_q), 141.3 (C_q) and 176.9 (*C*O_2Bn); HRMS (ESI^+) m/z: $[M+H]^+$ Calcd for $C_{31}H_{36}N_2O_2$ 469.2855; found 469.2858. Stereochemistry was determined as shown on pages S86-90.

Compound (±)-16b

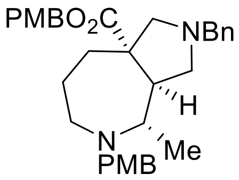

To a stirred solution of compound **8e** (51.5 mg, 0.13 mmol) and *N*-(methoxymethyl)-*N*-(trimethylsilylmethyl)benzylamine **15** (73.2 µL, 90% purity, 0.26 mmol) in DCM (3.2 mL) at rt under argon, TFA (11 µL, 0.14 mmol) was added. The mixture was stirred for 4 h, diluted with DCM (15 mL) and washed with saturated $NaHCO_3$ (15 mL). The aq. phase was extracted further with DCM (15 mL), the combined organic phase was dried ($MgSO_4$) and evaporated to give crude yellow oil (136 mg). Purification by silica gel chromatography (Et_2O/$CHCl_3$, 0:100 to 1:4 as eluent) afforded the product as a clear oil (55 mg, 80%). ν_{max} /cm^{-1} (neat); 2960, 2928, 2834, 2787, 1725, 1611, 1510, 1453, 1364; δ_H ($CDCl_3$, 400 MHz) 1.15 (3H, d, J = 6.4 Hz, CH*Me*), 1.23 – 1.32 (1H, m, NCH_2CH*H*), 1.38 – 1.51 (1H, m, NCH_2CH*H*), 1.67 (1H, t, J = 13.1 Hz, NCH_2CH_2CH*H*), 1.88 (1H, t, J = 9.2 Hz, CHCH*H*NBn), 2.00 (1H, d, J = 9.5 Hz, $C_q(CO_2)CH$*H*), 2.12 - 2.22 (1H, m, NCH_2CH_2CH*H*), 2.48 (1H, t, J = 12.6 Hz, NCH*H*CH_2), 2.76 (1H, dt, 14.6, 4.0 Hz, , NCH*H*CH_2), 2.96 (1H, dq, J = 12.7, 6.6, Hz, C*H*Me), 3.08 (1H, t, J = 8.2 Hz, CHCH*H*NBn), 3.23 – 3.42 (4H, m, $C_q(CO_2)CH$*H* and CHCH*H*N and NCH*H*ArOMe) and NCH*H*Ph), 3.59 (1H, d, J = 13.2 Hz, NCH*H*Ph), 3.72 (1H, d, J = 13.2 Hz, NCH*H*ArOMe), 3.77 (3H, s, ArO*Me*), 3.80 (3H, s, ArO*Me*), 5.15 (2H, A/B q, CO_2C*H_2*), 6.80 (2H, d, J = 8.1 Hz, 2 x C*H*$_{Ar}$), 6.86 (2H, d, J = 8.2 Hz, 2 x C*H*$_{Ar}$), 7.10 (2H, d, J = 8.1 Hz, 2 x C*H*$_{Ar}$), 7.22 – 7.32 (7H, m , 7 x C*H*$_{Ar}$); δ_C ($CDCl_3$, 100 MHz) 20.7 (CH*Me*), 22.5 (NCH_2*C*H_2), 33.9 (NCH_2CH_2*C*H_2), 46.6 (*C*HCH_2), 48.1 (N*C*H_2PhOMe), 51.9 (N*C*H_2CH_2), 55.4 (ArO*Me*), 56.3 (*C*$_qCO_2$), 59.2 (*C*HMe), 61.0 ($C_q(CO_2)$*C*H_2N), 66.7 (CO_2*C*H_2), 67.3 (CH*C*H_2N), 113.5 (*C*H_{Ar}), 113.9 (*C*H_{Ar}), 127.0 (*C*H_{Ar}), 128,3 (*C*H_{Ar}), 128.5 (*C*$_{qAr}$), 128.6 (*C*H_{Ar}), 129.8 (*C*H_{Ar}) 130.3 (*C*H_{Ar}), 133.0 (*C*$_{qAr}$), 138.8 (*C*H_{Ar}), 158.4 (*C*$_{qAr}$), 159.6 (*C*$_{qAr}$), 176.9 (*C*O_2); HRMS (ESI^+) m/z: $[M+H]^+$ calcd for $C_{33}H_{40}N_2O_2$ 529.3068; found 529.3070. Stereochemistry was determined in an analogous manner to the compound above as shown in S91-94.

Compound 16b: via in situ [3 +2] trapping

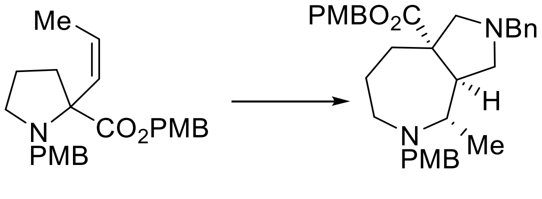

A flame dried Schlenk tube underwent three vacuum/argon cycles. To this tube $[Pd(allyl)Cl]_2$ (2.0 mg, 5.5 x 10^{-3} mmol) was added followed by one more vacuum/argon cycle before adding dry DCM (0.5 mL) with stirring. $P(OPh)_3$ (5.8 µL, 0.022 mmol) was added followed by morpholine (3.7 µL, 0.042 mmol). The mixture was stirred at rt for 5 mins until yellow solution had formed. Substrate **7e** (42 mg, 1.1 x 10^{-1} mmol) was dissolved in dry DCM (1.1 mL) under argon and added to the Schlenk tube. TFA (8.4 µL, 0.11 mmol) was added, the tube sealed and

heated to 40 °C. After 2.5 h the mixture was cooled to 30 °C, and *N*-(methoxymethyl)-*N*-(trimethylsilylmethyl)benzylamine **15** (62.5 µL, 90% purity, 0.22 mmol) and TFA (4.2 µL, 0.055 mmol) added. These additions were repeated after a further 2 h and 4 h. After 16 h the mixture was cooled to rt, washed with saturated $NaHCO_3$ (15 mL) and extracted with DCM (2 x 15 mL). The combined organic phase was dried ($MgSO_4$) and evaporated to give an orange oil (^{1}H NMR yield using DMF as internal standard. 79%). Purification by silica gel chromatography ($Et_2O/CHCl_3$, 1:99 to 1:3 as eluent) afforded analytically pure material as a clear oil (38 mg, 65%).

Compound (±)-17

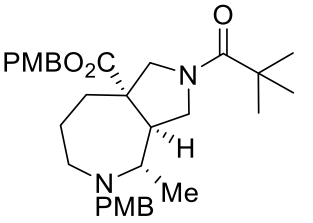

To a stirred solution of compound **16b** (54 mg, 0.10 mmol) in $CHCl_3$ (2.6 mL) at rt under argon, 1-chloroethyl chloroformate (47 µL, 0.4 mmol) was added dropwise. The mixture was stirred for 3 h then evaporated. The residue was redissolved in MeOH (3.1 mL) under argon and stirred for 20 h then evaporated. The resulting secondary amine salt was dissolved in DCM (3 mL) at rt with stirring under argon, Et_3N (56 µL, 0.40 mmol) was added and the mixture cooled to 0 °C. Pivaloyl chloride (18.4 µL, 0.15 mmol) was added dropwise and the mixture allowed to warm to rt over 4 h. The mixture was diluted with DCM (15 mL), washed with saturated $NaHCO_3$ (15 mL). The aq. phase was extracted once more with DCM (15 mL) and the combined organic phase dried ($MgSO_4$) to give a clear oil. Purification by silica gel chromatography ($EtOH/NH_3/DCM$, 1:99 to 4:96 as eluent) afforded the product as a clear oil (35 mg, 67% over 3 steps). ν_{max} /cm^{-1} (neat) 2957, 2933, 2836, 1724, 1611, 1511, 1409, 1245; δ_H ($CDCl_3$, 400 MHz) (rotameric) 1.08 (3H, d, J = 6.4 Hz, CH*Me*), 1.18 (9H, s, COC(*Me*)$_3$), 1.48 – 1.67 (2H, m, NC*H*$_2$), 1.89 (1H, t, J =12.3 Hz, NCH_2CH_2CH*H*), 2.00 – 2.08 (1H, m, NCH_2CH_2CH*H*), 2.53 – 2.66 (2H, m, NC*H*$_2$CH$_2$), 2.81 – 2.93 (2H, m, NC*H*Me and C$_q$(CO$_2$)CH*H*N), 3.39 – 3.63 (5H, m, NC*H*$_2$Ph and C*H*CH$_2$N and CHCH*H*N and C$_q$(CO$_2$)CH*H*N), 3.75 – 3.85 (7H, m, 2 x ArO*Me* and CHCH*H*N), 5.08 (2H, A/B q, CO$_2$C*H*$_2$Ph), 6.80 (2H, d, J = 8.5 Hz, 2 x C*H*$_{Ar}$), 6.86 (2H, d, J = 8.6 Hz, 2 x C*H*$_{Ar}$), 7.12 (2H, d, J = 8.6 Hz, 2 x C*H*$_{Ar}$), 7.27 (2H, d, J = 8.5 Hz, 2 x C*H*$_{Ar}$); δ_C ($CDCl_3$, 100 MHz); 15.1 (NCH*Me*), 23.9 (NCH$_2$*C*H$_2$), 27.6 (C$_q$(*Me*)$_3$), 38.9 (*C*$_q$(Me)$_3$), 39.4 (CHMe*C*H), 50.2 (C$_q$(CO$_2$)*C*H$_2$), 50.6 (N*C*H$_2$CH$_2$), 51.2 (CH*C*H$_2$N), 52.5 (N*C*H$_2$Ph), 55.2 (ArO*Me*), 56.4 (*C*$_q$CO$_2$), 59.7 (N*C*HMe), 66.7 (CO$_2$*C*H$_2$Ph), 113.7 (*C*H$_{Ar}$), 114.0 (*C*H$_{Ar}$), 127.9 (*C*H$_{Ar}$), 130.1 (*C*H$_{Ar}$), 130.4 (*C*H$_{Ar}$), 132.2 (*C*H$_{Ar}$), 158.7 (*C*$_{qAr}$), 159.8 (*C*$_{qAr}$), 175.3 (N*C*O$_2$), 176.3 (C$_q$*C*O$_2$); HRMS (ESI$^+$) m/z: $[M+H]^+$ calcd for $C_{31}H_{42}N_2O_5$ 523.3174; found 523.3176.

Compound 18: Using enantioenriched **8e**

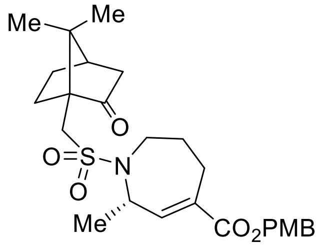

To a solution of **8e** (32 mg, 0.081 mmol) in $CHCl_3$ (3 mL) at rt under argon was added 1-chloroethyl chloroformate (40 µL, 0.37 mmol) and the reaction stirred at rt. After 3 h the mixture was concentrated *in vacuo* and the residue dissolved in methanol (2.5 mL). After stirring for a further 3.5 h the reaction was concentrated in vacuo and the residue dissolved in $CHCl_3$ (2.5 mL) under argon. Et_3N (75 µL, 0.054 mmol) and (+)-CSA-Cl (37 mg, 0.15 mmol) were added and the reaction stirred at rt. After 3 h the reaction was worked up (DCM/sat. aq. $NaHCO_3$) and silica gel chromatography (EtOAc/petrol, 1:19 to 1:4 as eluent) afforded the title compound (29 mg, 73%) as a yellow oil. *d.r.* > 9:1. $[\alpha]^{20}_D$ = +62.6 (*c* 0.032, DCM). ν_{max} /cm^{-1} (film) 2954, 1745, 1707, 1613, 1515, 1455, 1334 and 1240; δ_H ($CDCl_3$, 400 MHz) 0.86 (3H, s, CMe*Me*), 1.12 (3H, s, C*Me*Me), 1.36 – 1.42 (1H, m, C$_q$CH$_2$CH*H*CH), 1.39 (3H, d, J 7.0, NCH*Me*), 1.62 (1H, ddd, J 13.8, 9.3, 4.5, C$_q$CH*H*CH$_2$CH), 1.69 – 1.80 (1H, m, NCH$_2$CH*H*), 1.92 (1H, d, J 18.4, CH*H*C=O), 1.96 – 2.06 (2H, m, NCH$_2$C*H*H and C$_q$CH$_2$C*H*HCH), 2.08 (1H, app. t, J 4.4, C*H*CH$_2$C=O), 2.36 (1H, dt, J 18.7, 3.8, C*H*HC=O), 2.46 – 2.55 (1H, m, C$_q$C*H*HCH$_2$CH), 2.55 - 2.65 (1H, m, NCH$_2$CH$_2$CH*H*), 2.75 (1H, ddd, J 16.7, 6.5, 2.9, NCH$_2$CH$_2$C*H*H), 2.84 (1H, d, J 14.5, CH*H*SO$_2$), 3.30 (1H, d, J 14.5, C*H*HSO$_2$), 3.28 – 3.35 (1H, m, NCH*H*), 3.72 – 3.79 (1H, m, NC*H*H), 4.82 – 4.90 (1H, m, NC*H*Me), 5.04 – 5.12 (2H, AB-q, C*H*$_2$OAr), 6.78 (1H, dd, J 5.1, 1.9, =C*H*), 6.86 –

6.90 (2H, m, 2 × C*H*$_{Ar}$) and 7.28- 7.31 (2H, m, 2 × C*H*$_{Ar}$); δ$_C$ (CDCl$_3$, 101 MHz) 18.5 (CH*Me*), 19.9 (CMe*Me*), 20.2 (C*Me*Me), 23.4 (NCH$_2$CH$_2$*C*H$_2$), 25.4 (C$_q$CH$_2$CH$_2$CH), 27.0 (C$_q$CH$_2$*C*H$_2$CH), 27.3 (NCH$_2$*C*H$_2$), 42.7 (*C*HHC=O), 42.8 (N*C*H$_2$), 43.0 (*C*HCH$_2$C=O), 47.9 (*C*Me$_2$), 49.7 (*C*H$_2$SO$_2$), 52.6 (N*C*HMe), 55.4 (OMe), 58.6 (*C*$_q$CH$_2$SO$_2$), 66.8 (*C*H$_2$OAr), 114.0 (2 × *C*H$_{Ar}$), 128.2 (C$_{qAr}$), 130.2 (2 × *C*H$_{Ar}$), 133.3 (*C*$_q$=CH), 142.8 (=*C*H), 159.7 (*C*$_q$OMe), 167.6 (*C*O$_2$CH$_2$Ar) and 215.4 (ketone); HRMS (ESI$^+$) m/z: [M+H]$^+$ calcd for $C_{26}H_{36}NO_6S$ 490.2265; found 490.2261.

Compound (±)-19

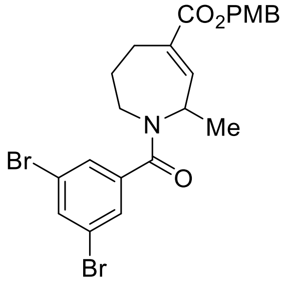

To a solution of (±)-**8e** (80 mg, 0.20 mmol) in CHCl$_3$ (6 mL) at rt under argon was added 1-chloroethyl chloroformate (80 μL, 0.74 mmol) and the reaction stirred at rt. After 4 h the mixture was concentrated *in vacuo* the residue dissolved in methanol (2.5 mL). After stirring for a further 3.5 h the reaction was concentrated in vacuo and the residue dissolved in DCM (3 mL). The solution was cooled to 0 °C under argon and Et$_3$N (120 μl, 0.86 mmol) and 3,5-dibromobenzoyl chloride (95 mg, 0.32 mmol) added to the stirred mixture. After 75 min the reaction was partitioned between DCM and sat. aq. NaHCO$_3$ and the organic phase dried (MgSO$_4$) and evporated. Purification by silica gel chromatography (EtOAc/petrol, 1:9 to 3:7 as eluent) afforded the title compound (108 mg, 78%) as a clear oil. ν$_{max}$ /cm^{-1}: 2935, 1706, 1632, 1515, 1408 and 1245; δ$_H$ (CDCl$_3$, 400 MHz) *3:2 mixture of rotamers* 1.33 (3H, d, J 6.7, Me, (minor)), 1.40 (3H, d, J 7.2, Me (major)), 1.59 – 1.86 (2H, m, NCH$_2$C*H*$_2$ (major and minor, plus C$_q$CH*H* (minor)), 2.18 – 2.30 (1H, m, C$_q$C*H*H (minor)), 2.31 – 2.43 (1H, m, C$_q$CH*H* (major)), 2.79 (1H, d, J 15.1, C$_q$C*H*H (major)), 3.10 (1H, dt, *J* 14.3, 7.3, NCH*H* (minor)), 3.27 – 3.48 (2H, m, NCH$_2$ (major)), 3.81 (3H, s, OMe (major and minor), 4.31 (1H, dd, *J* 13.6, 6.6 Hz, NC*H*H (minor)), 4.48 – 4.58 (1H, m, NC*H* (minor)), 5.05 – 5.18 (2H, AB-q, OC*H*$_2$ (major and minor)), 5.45 – 5.55 (1H, m, NCH (major)), 6.58 (1H, s, C*H*$_{Ar}$ (minor)), 6.80 (1H, m, C*H*$_{Ar}$ (major)), 6.85 – 6.92 (1H, m, =C*H* (major and minor) and CH$_{Ar}$ (major and minor)), 7.27 – 7.35 (2H, m, 2 × C*H*$_{Ar}$ (major and minor)), 7.37 – 7.45 (2H, m, 2 × C*H*$_{Ar}$ (major and minor)) and 7.70 (1H, s, CH$_{Ar}$ (major and minor)); δ$_C$ (CDCl$_3$, 101 MHz) 18.4 (Me (major)), 19.4 (Me (minor)), 22.3 (C$_q$*C*H$_2$ (major)), 25.3 (C$_q$*C*H2 (minor)), 26.5 (NCH$_2$*C*H$_2$ (major and minor)), 39.7 (NCH$_2$ (minor)), 43.8 (NCH$_2$ (major)), 50.7 (NCH (major)), 55.4 (OMe (major and minor)), 55.5 (NCH (minor)), 66.9 (OCH$_2$ (major)), 67.0 (OCH$_2$ (minor)), 114.1 (=CH (major and minor)), 123.4 (=*C*$_q$ (major)), 123.5 (=*C*$_q$ (minor)), 128.0 (2 × CH$_{Ar}$ (major)), 128.2 (2 × CH$_{Ar}$ (minor)), 130.3 (2 × CH$_{Ar}$ (major and minor)), 131.5 (C$_q$), 133.1 (C$_q$), 135.2 (CH$_{Ar}$ (major and minor)), 139.7 (C$_q$), 140.0 (C$_q$), 141.1 (*C*H$_{Ar}$ (minor)), 142.5 (*C*H$_{Ar}$ (major)), 159.8 (C$_q$OMe) (major and minor)), 167.1 (C=O (major)), 167.3 (C=O (minor)), 168.1 (C=O (minor)) and 168.3 (C=O (major)); HRMS (ESI$^+$) m/z: [M+H]$^+$ calcd for $C_{23}H_{24}NO_4Br_2$ 538.0053; found 538.0051.

Compound (±)-20

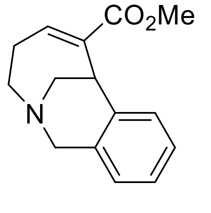

To a flame-dried Schlenk tube under argon were added Pd(OAc)$_2$ (5.0 mg, 0.022 mmol), PPh$_3$ (11.7 mg, 0.044 mmol), K$_2$CO$_3$ (48.4 mg, 0.35 mmol) and tetrabutylammonium chloride (17 mg, 0.061 mmol). The flask was evacuated and backfilled with argon (3×) and dry MeCN (3 mL) was added. The mixture was stirred for 2 min, a solution of substrate **8n** (74.0 mg, 0.228 mmol) in dry MeCN (6 mL) added and the mixture heated to 80 °C. After 15 h the mixture was cooled, evaporated, and filtered through Celite, eluting with EtOAc. The filtrate was evaporated to give the crude product as a brown oil. Purification by silica gel chromatography (EtOH/NH$_3$ in DCM, 1% to 8% as eluent) afforded the title compound as a yellow oil (22.4 mg, 40%). ν$_{max}$ /cm^{-1} (neat) 3057, 3018, 2924, 2851, 1703, 1435, 1231; δ$_H$ (CDCl$_3$, 400 MHz) 2.09 (1H, dddd, J = 17.0, 7.8, 4.9, 2.3, NCH$_2$CH*H*), 2.48 (1H, ddt, J = 17.1, 10.8, 3.9 Hz, NCH$_2$CH*H*)), 3.11 – 3.25 (2H, m, NC*H*$_2$), 3.44 (2H, app. d, J = 3.1 Hz, NC*H*$_2$CH), 3.80 (3H, s, CO$_2$*Me*), 3.91 (1H, d, J = 17.4 Hz, NCH*H*Ph), 4.16 (1H, C*H*C$_q$CO$_2$), 4.43 (1H, d, J = 17.4 Hz, NCH*H*Ph), 7.00 – 7.09 (2H, m, 1 × C*H*$_{Ar}$ and C*H*=C$_q$CO$_2$) 7.11 – 7.23 (3H, m, 3 × C*H*$_{Ar}$); δ$_C$ (CDCl$_3$, 101 MHz); 26.9 (NCH$_2$*C*H$_2$), 35.1 (NCH$_2$*C*H), 51.2 N*C*H$_2$CH), 52.4 (CO$_2$*Me*),

53.2 (N*C*H_2Ph), 53.7 (N*C*H_2CH_2), 125.4 (*C*H_{Ar}), 126.9 (*C*H_{Ar}), 127.0 (*C*H_{Ar}), 129.4 (*C*H_{Ar}), 133.2 (C_{qAr}), 135.2 (C_q=CH), 140.0 (C_{qAr}) 143.2 (C_q=*C*H) and 168.4 (*C*O_2); HRMS (ESI^+) m/z: $[M+H]^+$ calcd for $C_{15}H_{18}NO_2$ 244.1338; found 244.1332.

4.0 NMR Spectra

^{1}H NMR ($CDCl_3$ 400 MHz) of compound S11

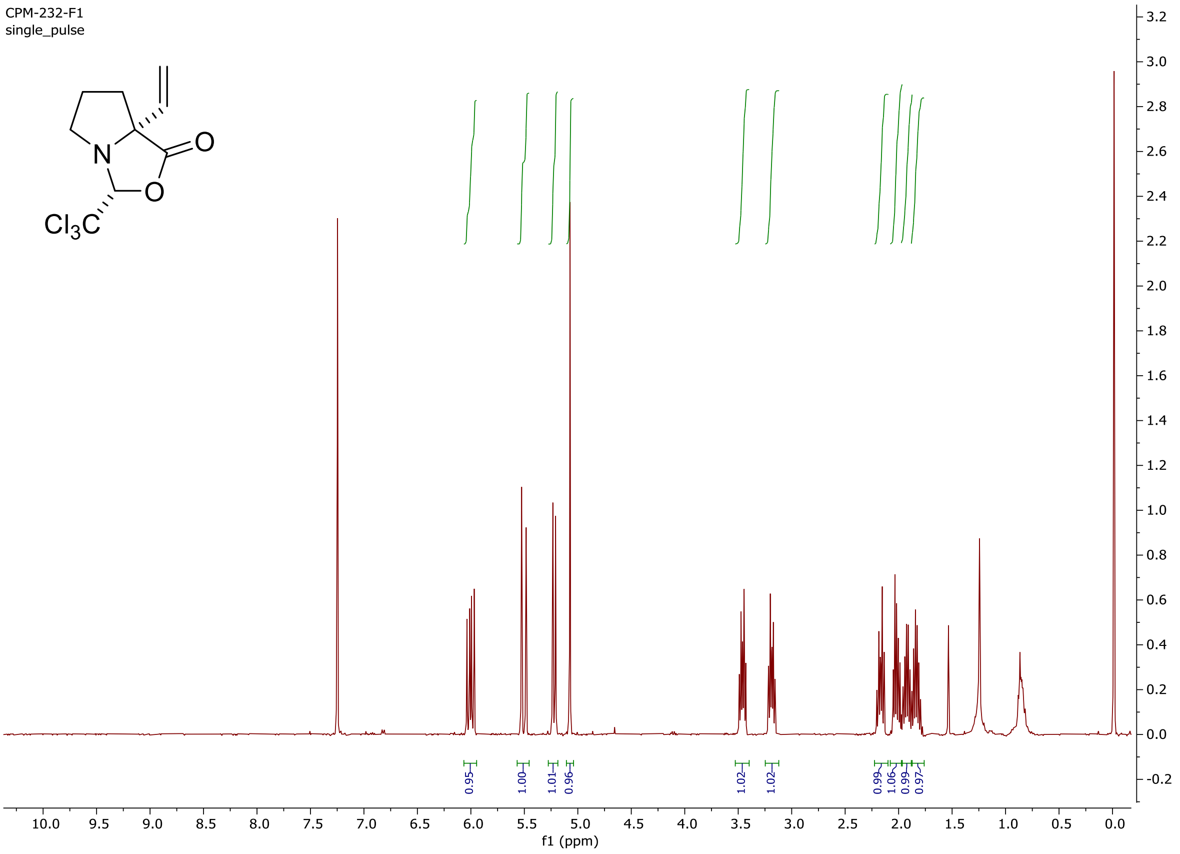

^{13}C NMR ($CDCl_3$ ($CDCl_3$ 100 MHz) of compound S11

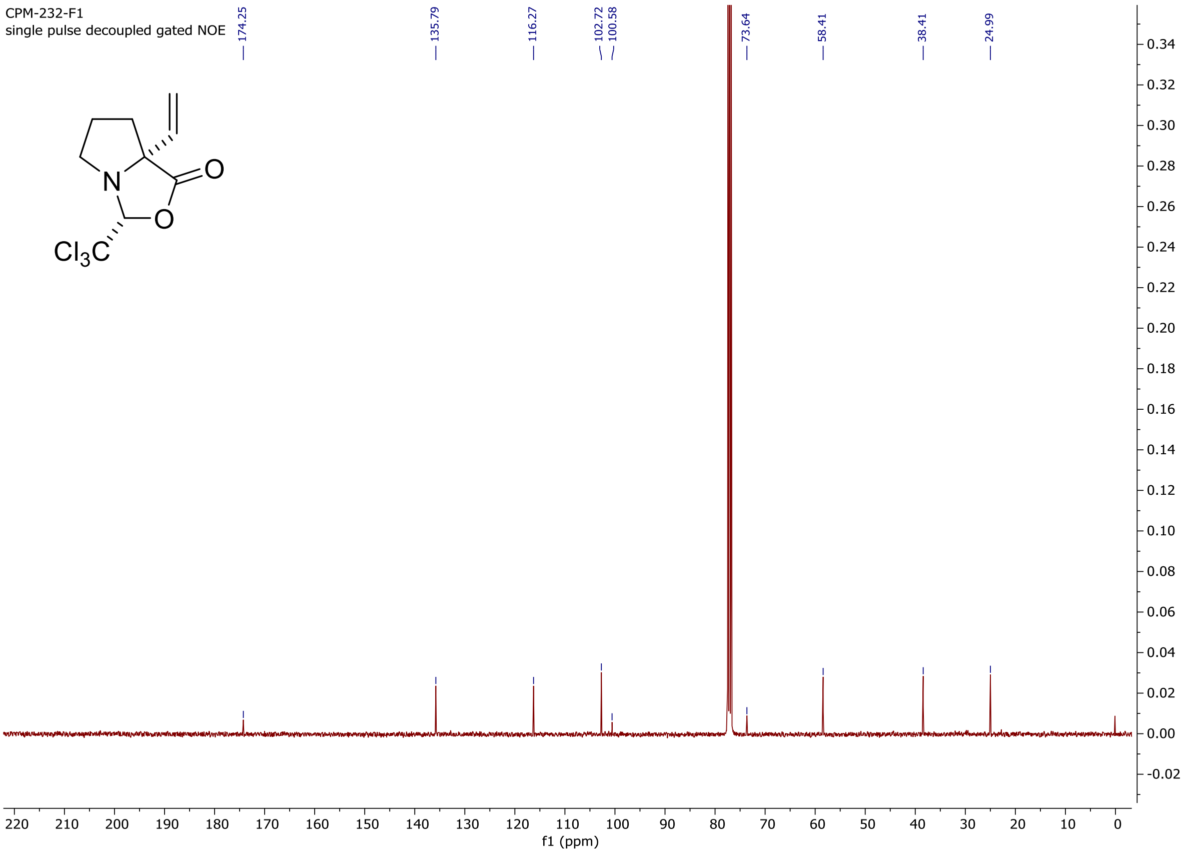

^{1}H NMR (CDCl3 400 MHz) of compound S12

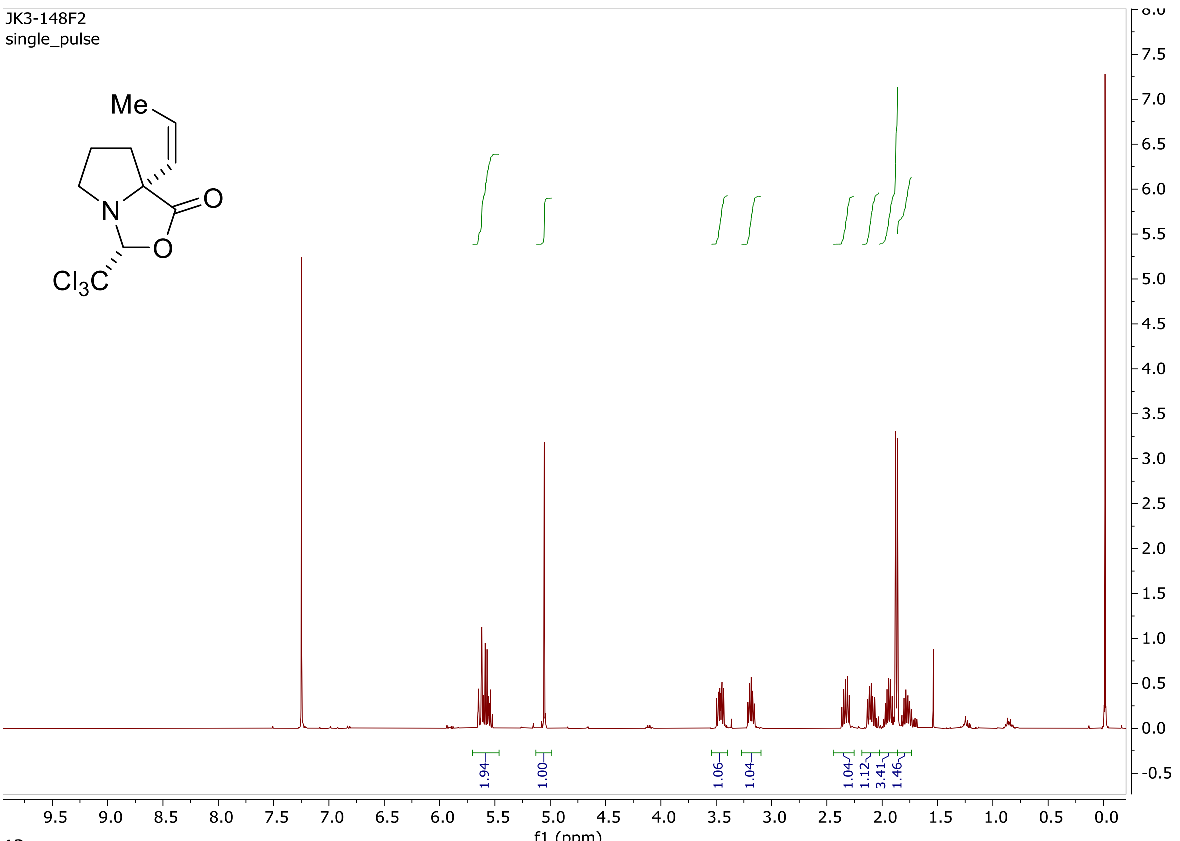

^{13}C NMR (CDCl3 (CDCl3 100 MHz) of compound S12

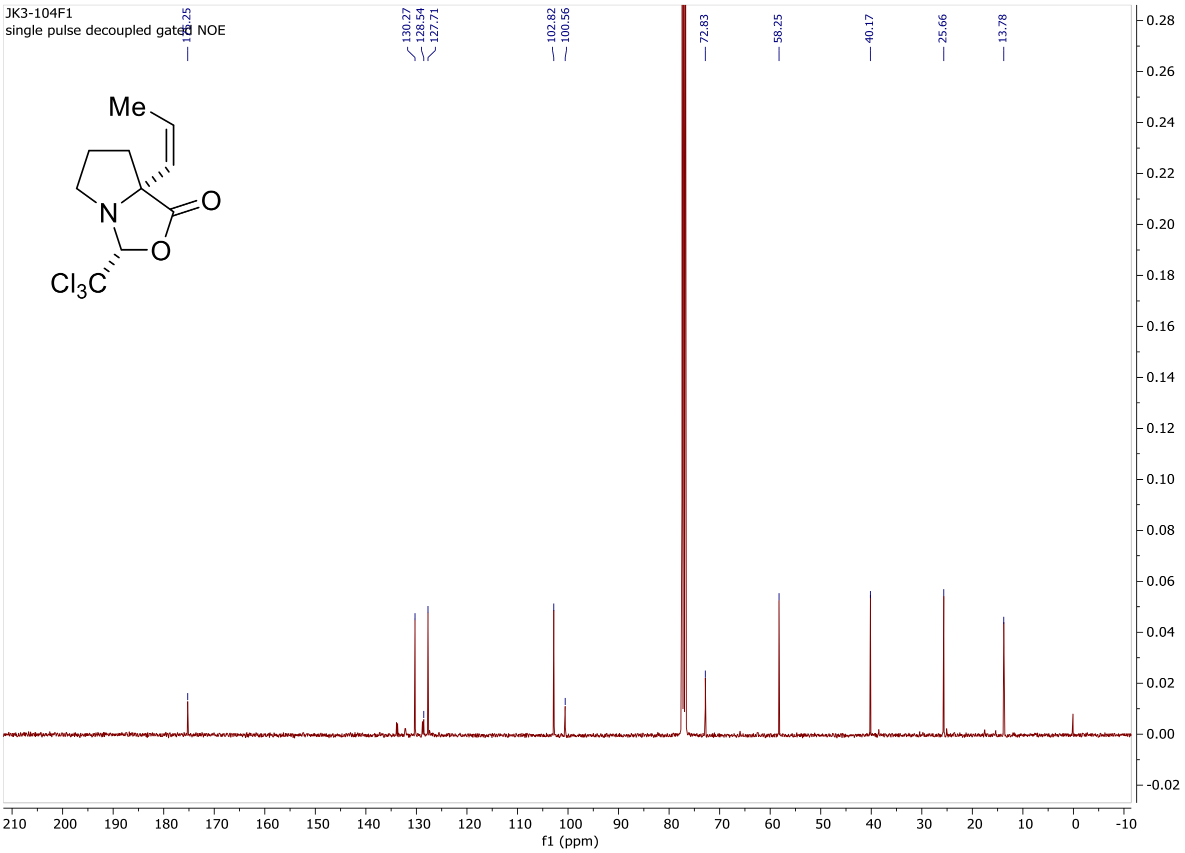

^{1}H NMR ($CDCl_3$ 400 MHz) of compound S13

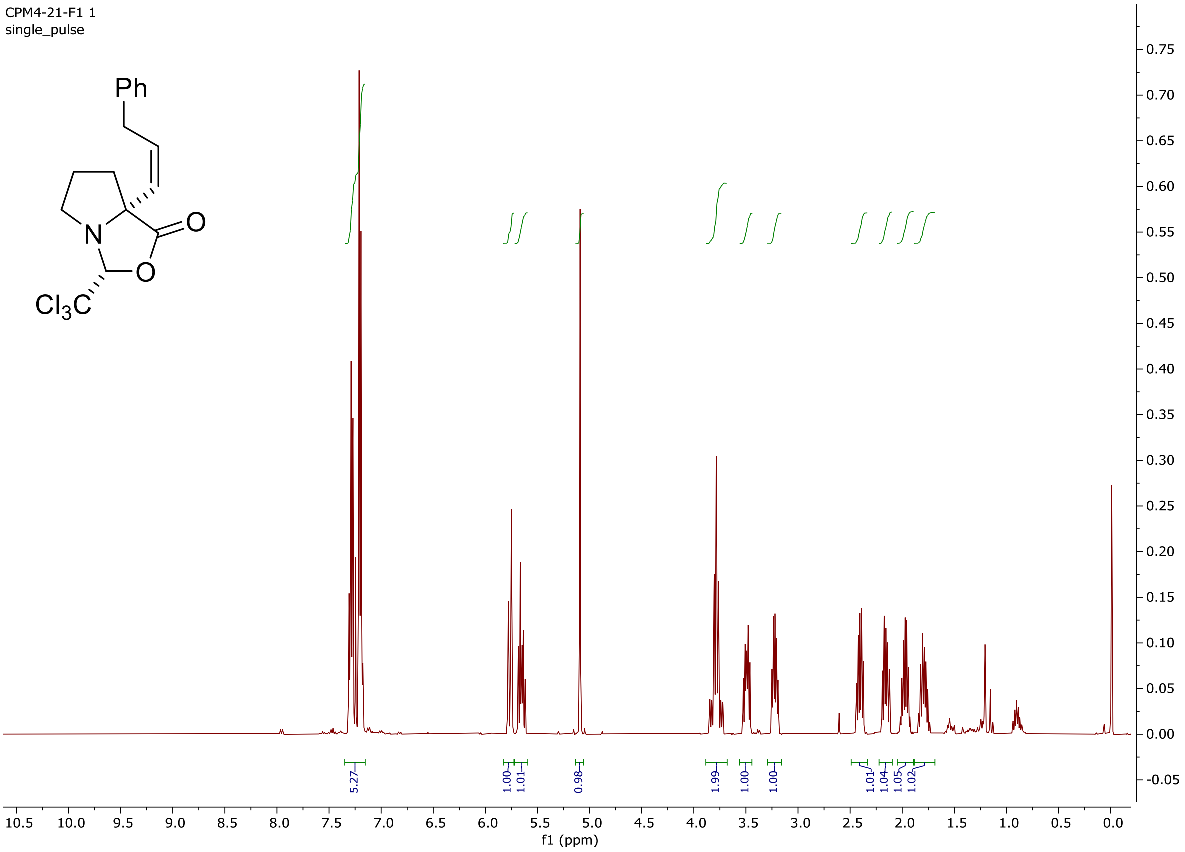

^{13}C NMR ($CDCl_3$ ($CDCl_3$ 100 MHz) of compound S13

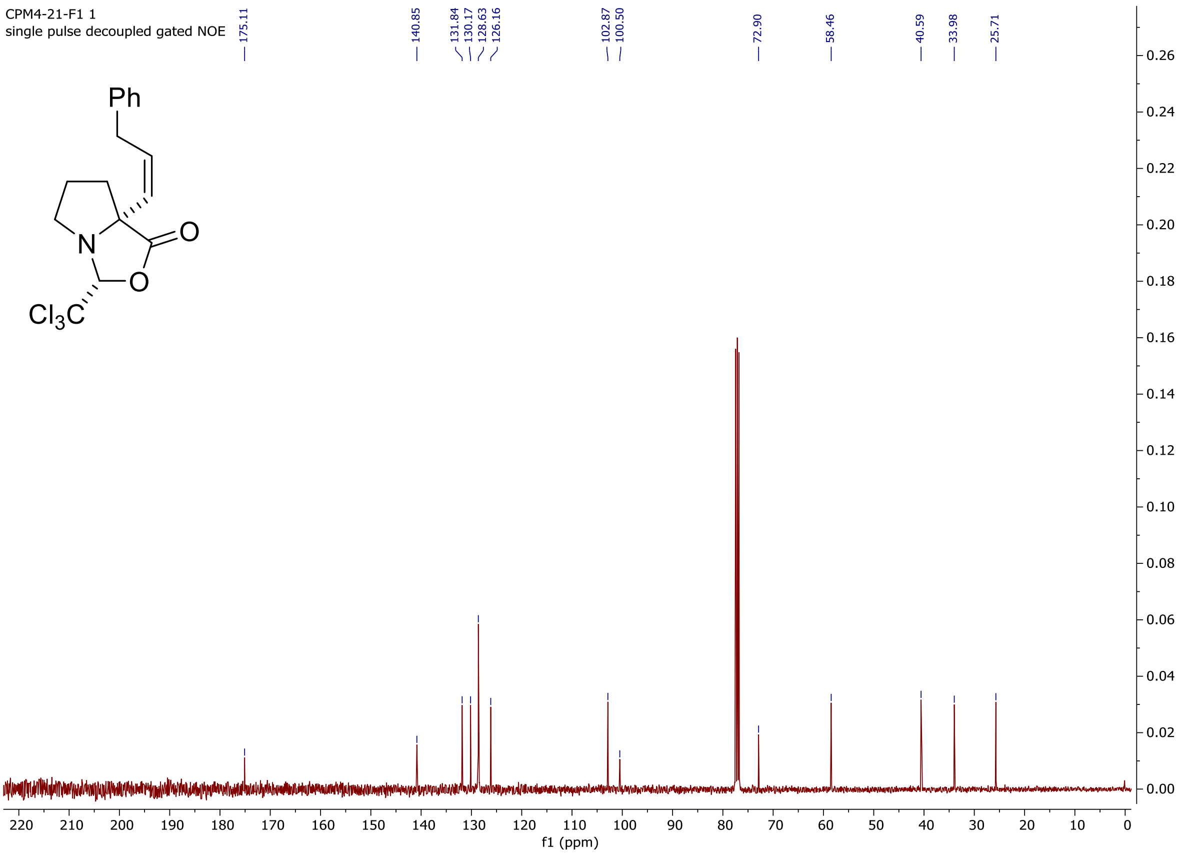

^{1}H NMR ($CDCl_3$ 400 MHz) of compound 7a

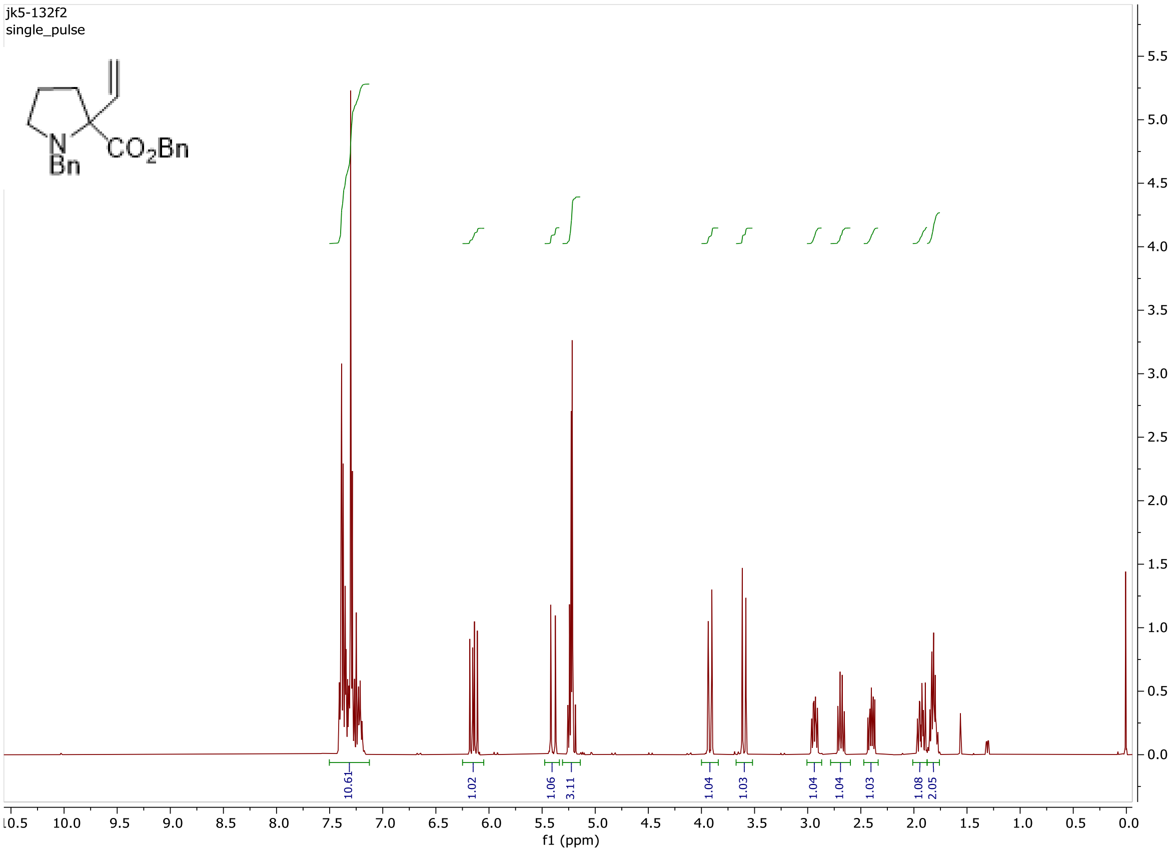

^{13}C NMR ($CDCl_3$ ($CDCl_3$ 100 MHz) of compound 7a

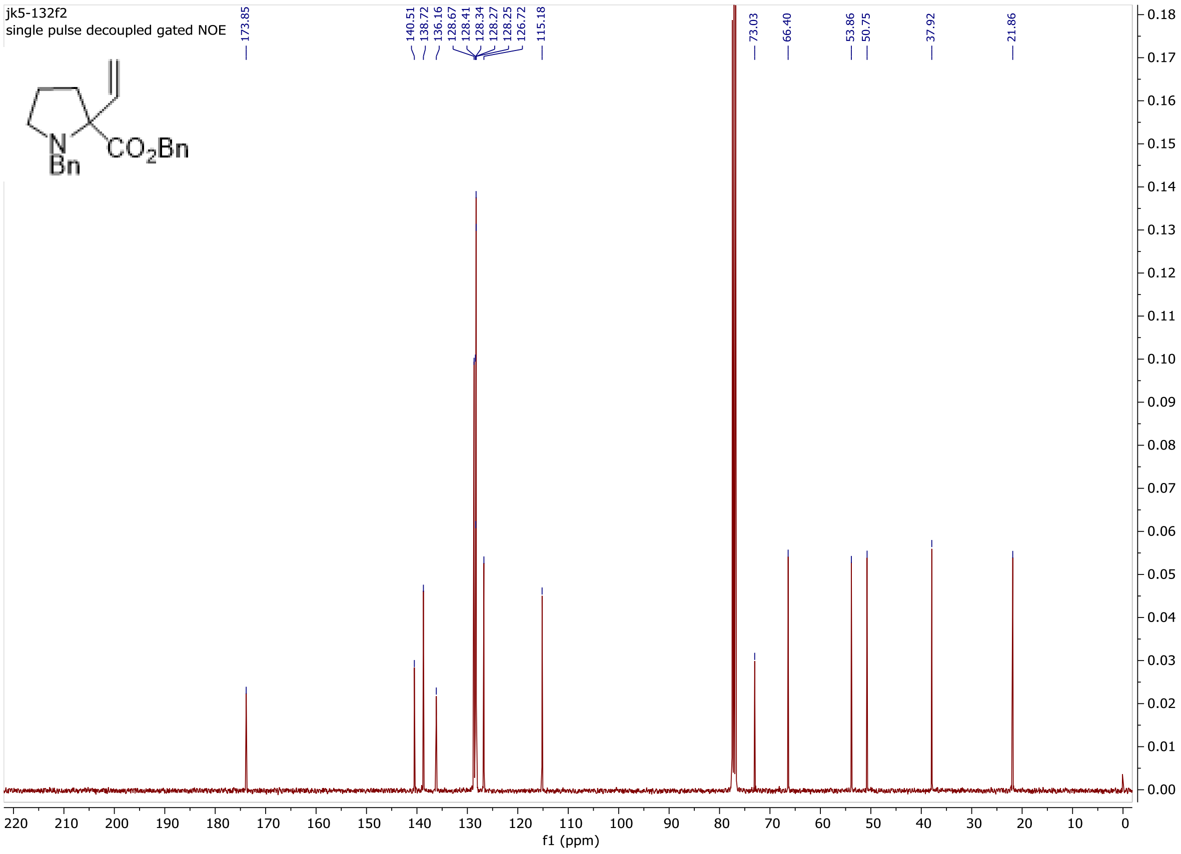

^{1}H NMR ($CDCl_3$ 400 MHz) of compound 7b

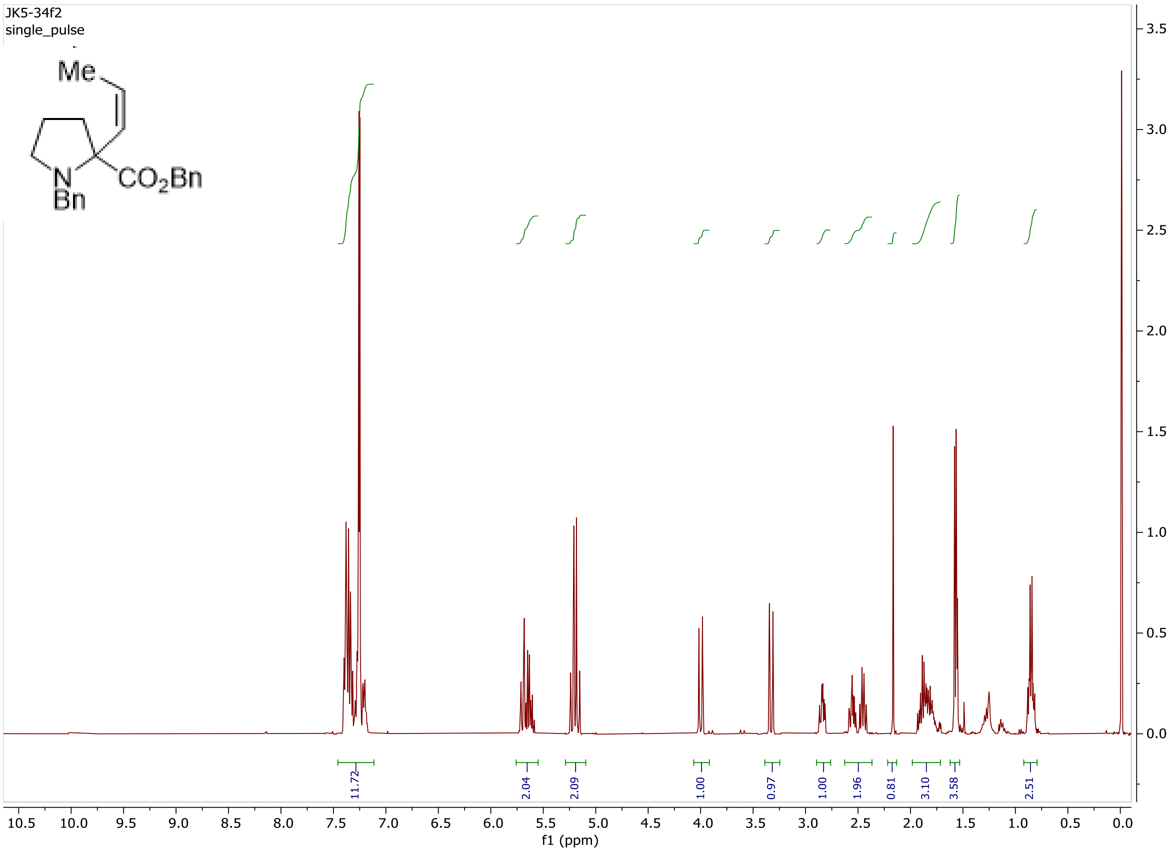

^{13}C NMR ($CDCl_3$ ($CDCl_3$ 100 MHz) of compound 7b

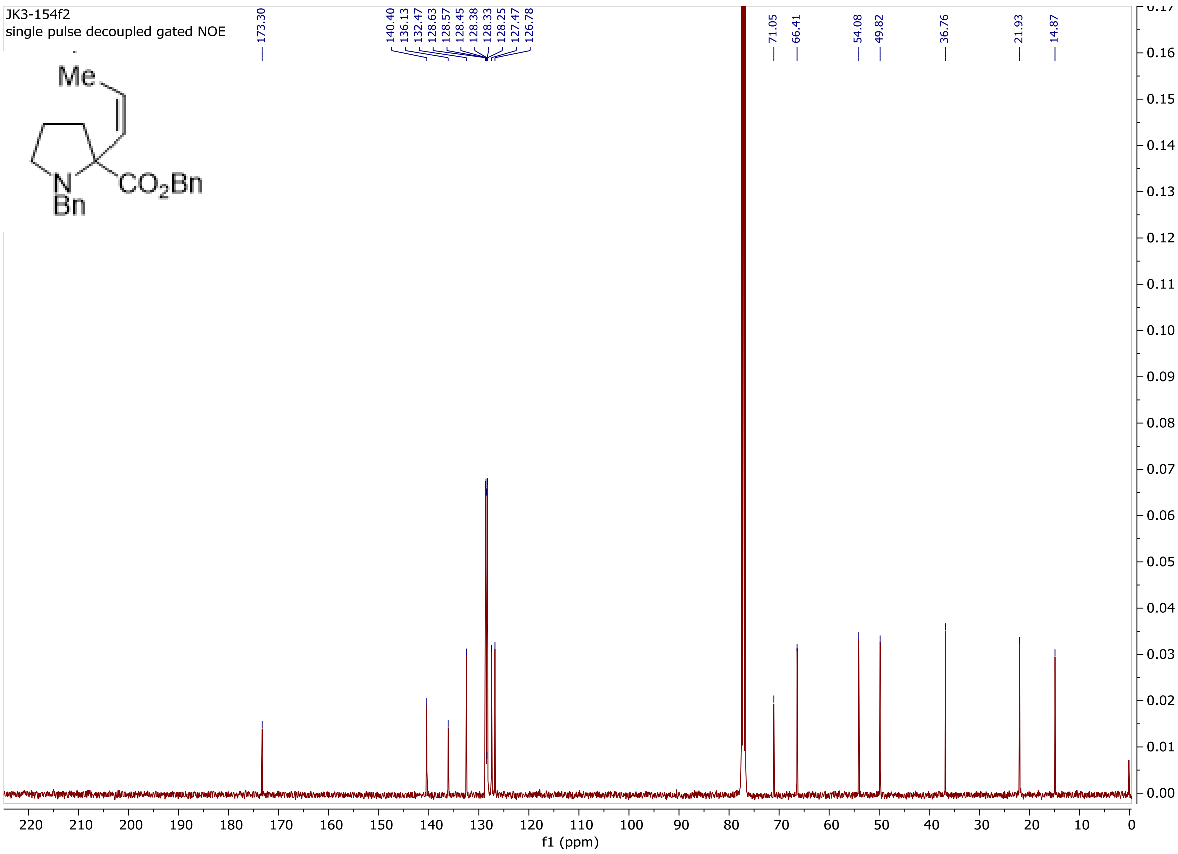

^{1}H NMR ($CDCl_3$ 400 MHz) of compound 7c

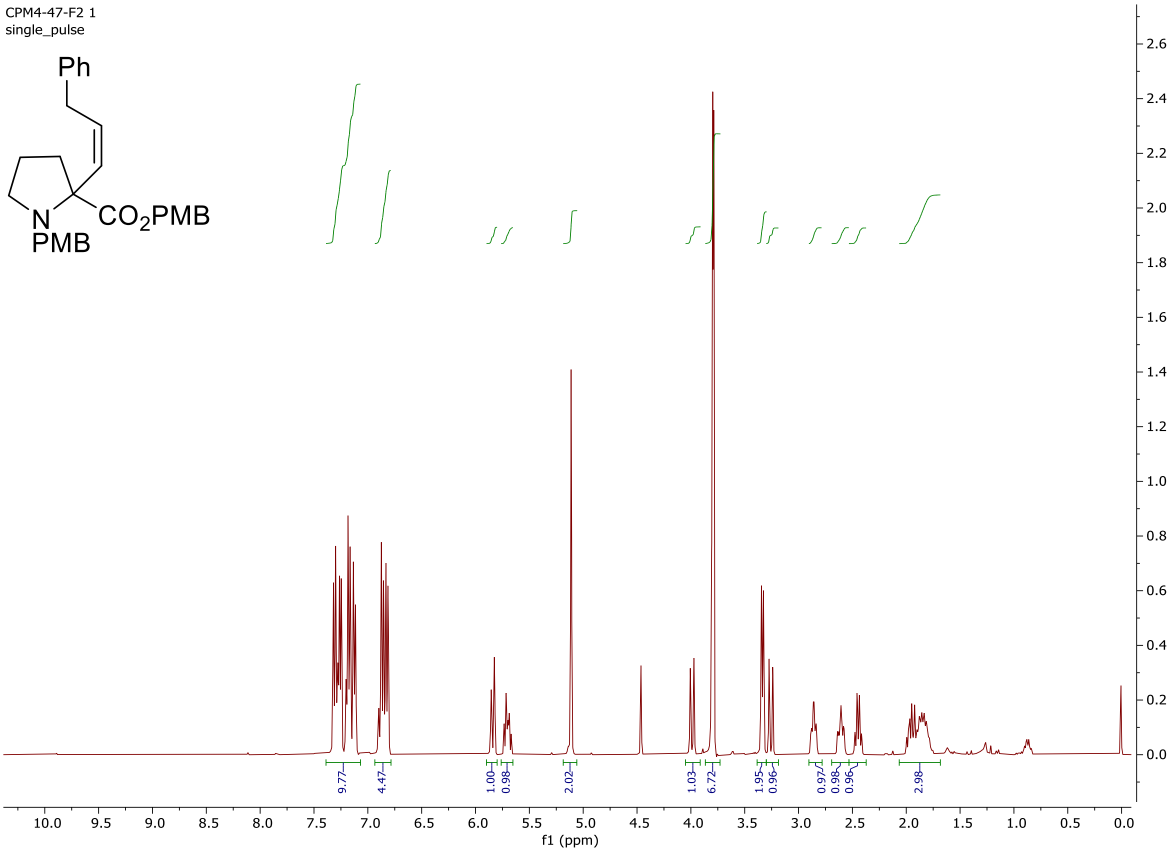

^{13}C NMR ($CDCl_3$ 100 MHz) of compound 7c

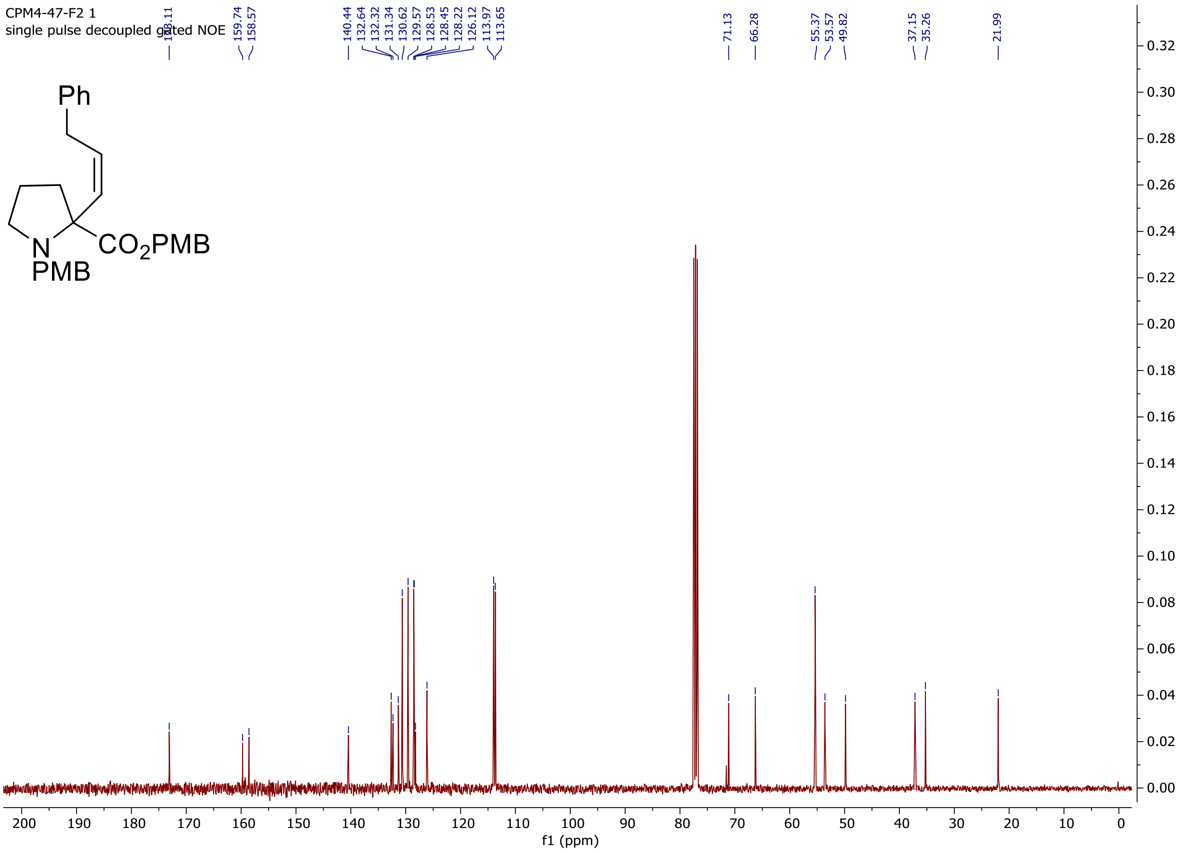

^{1}H NMR (CDCl$_3$ 400 MHz) of compound 7d

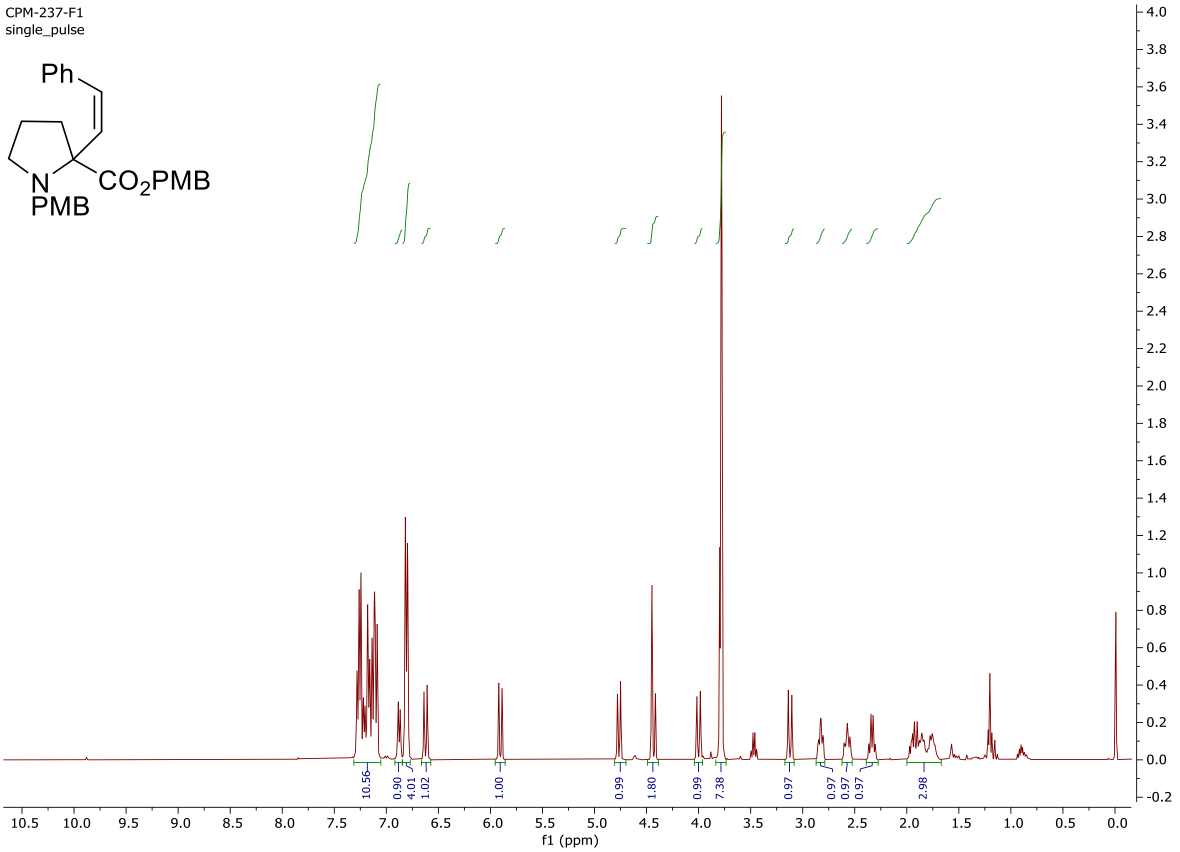

^{13}C NMR (CDCl$_3$ (CDCl$_3$ 100 MHz) of compound 7d

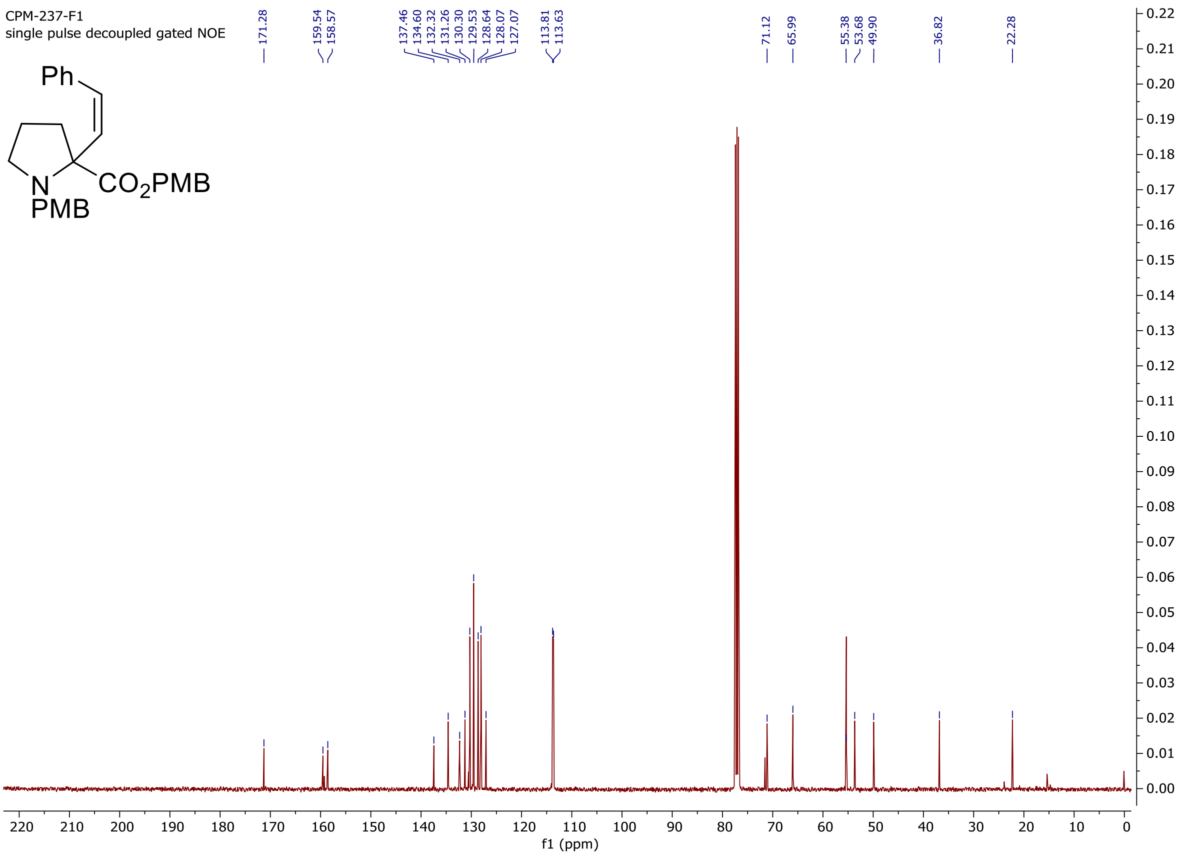

^{1}H NMR ($CDCl_3$ 400 MHz) of compound 7e

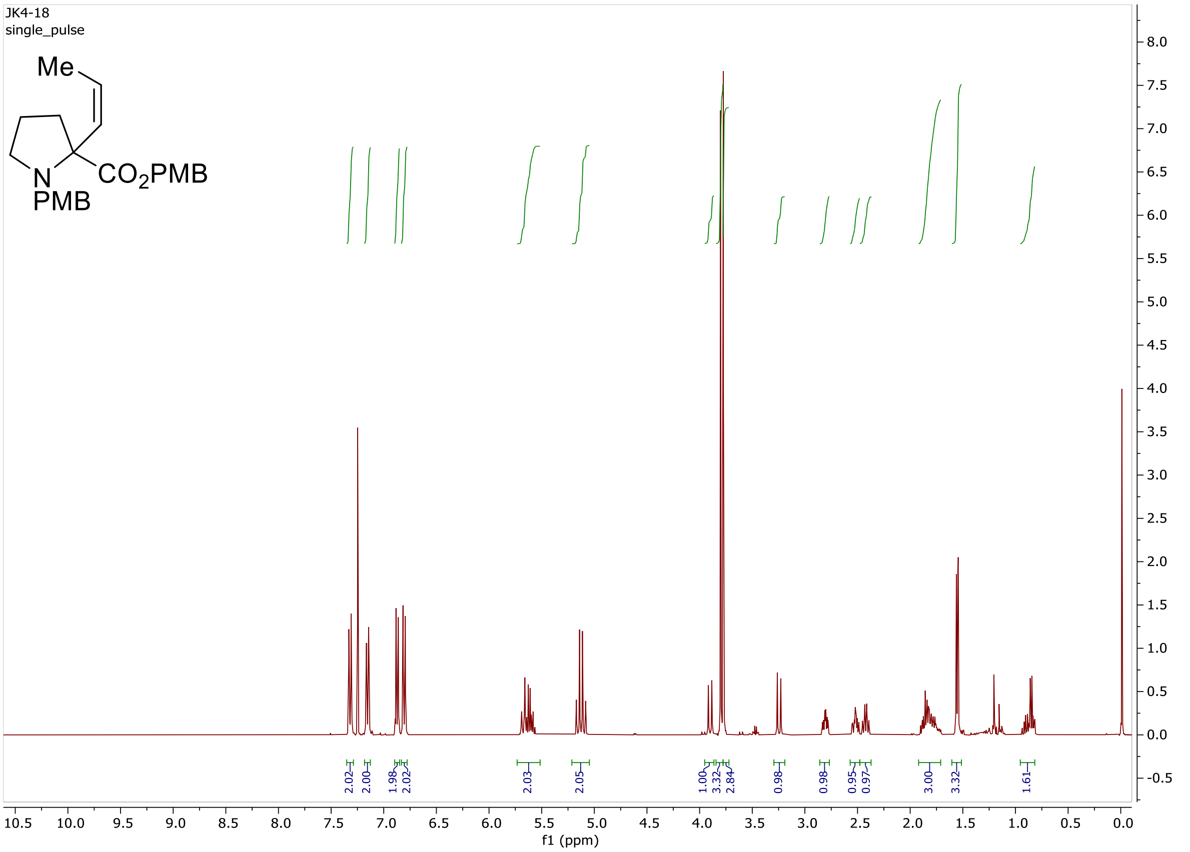

^{13}C NMR ($CDCl_3$ ($CDCl_3$ 100 MHz) of compound 7e

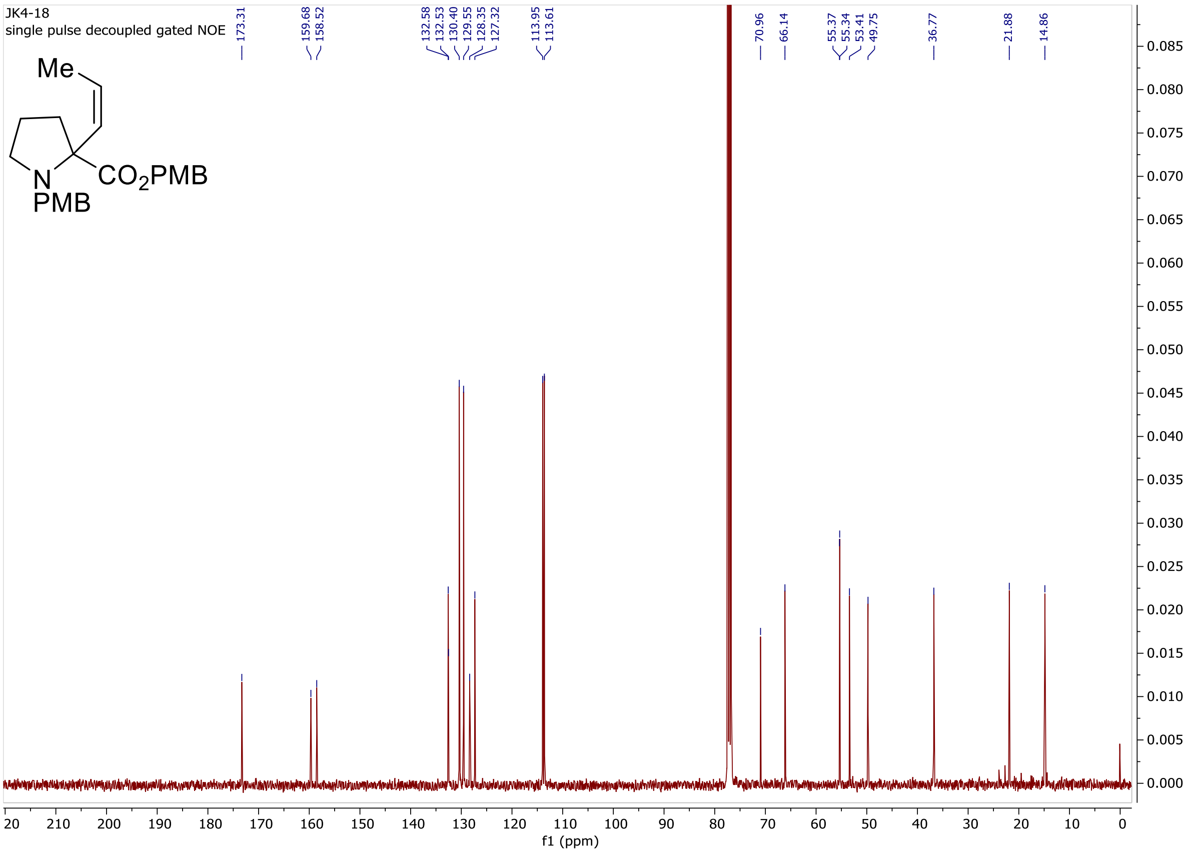

^{1}H NMR ($CDCl_3$ 400 MHz) of compound 7f

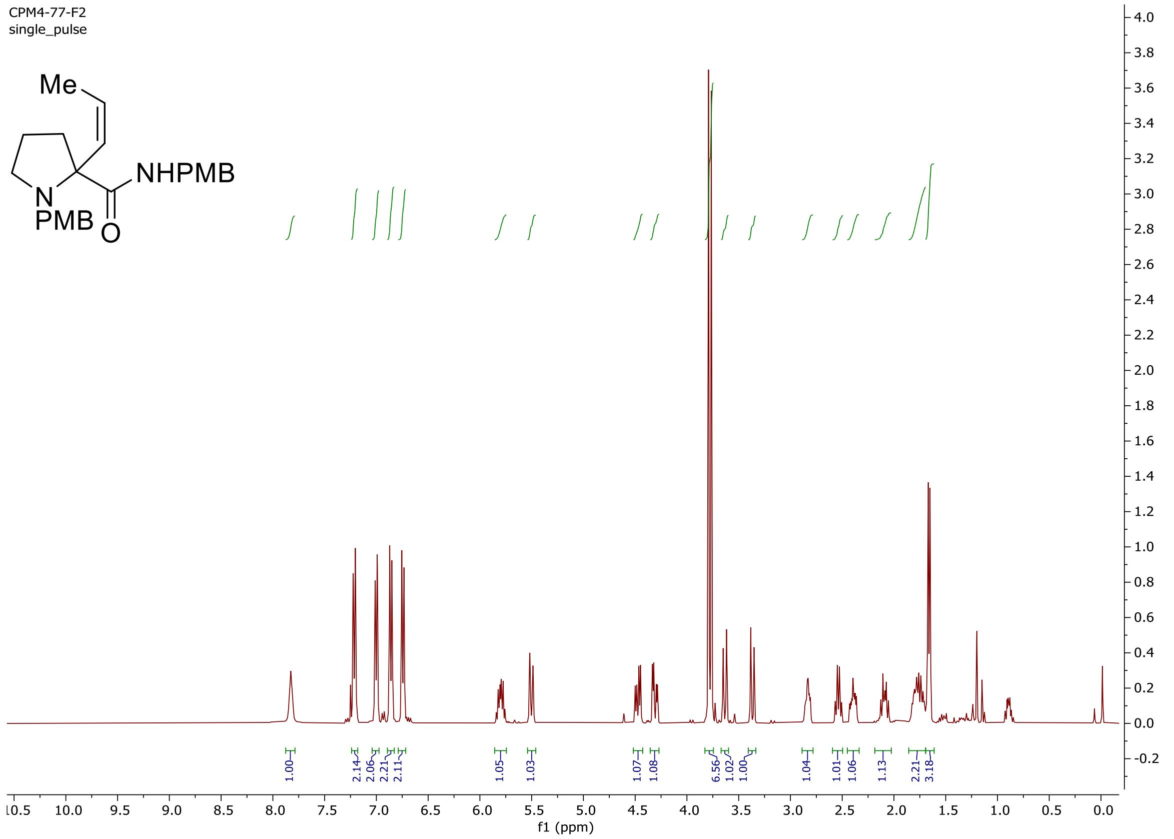

^{13}C NMR ($CDCl_3$ 100 MHz) of compound 7f

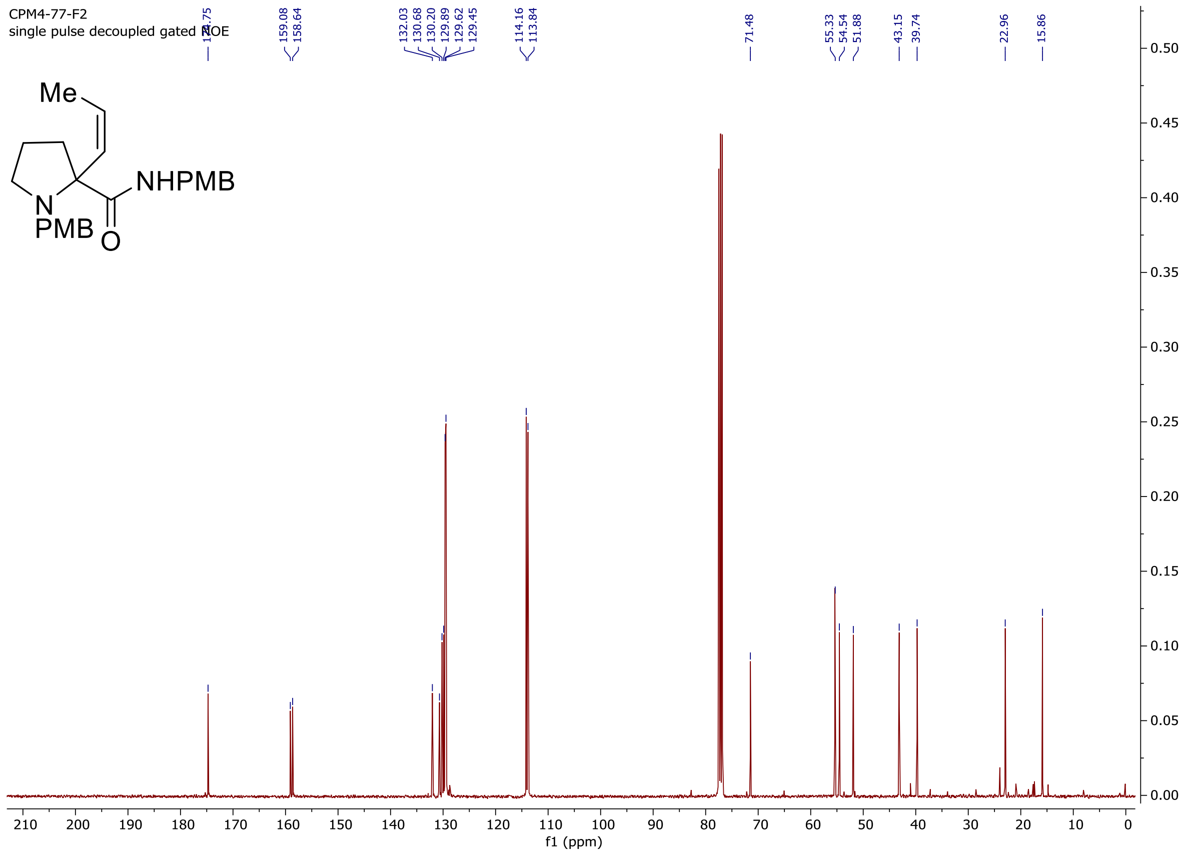

^{1}H NMR ($CDCl_3$ 400 MHz) of compound 7l

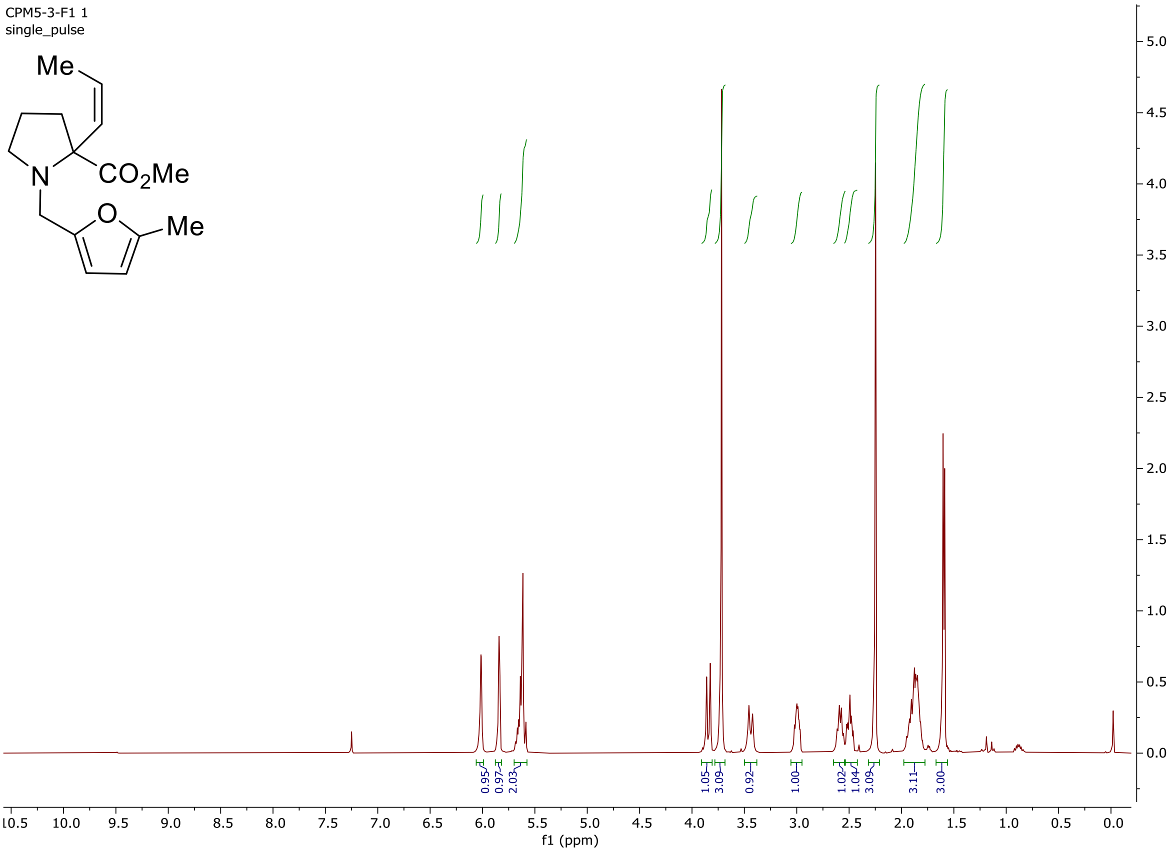

^{13}C NMR ($CDCl_3$ 100 MHz) of compound 7l

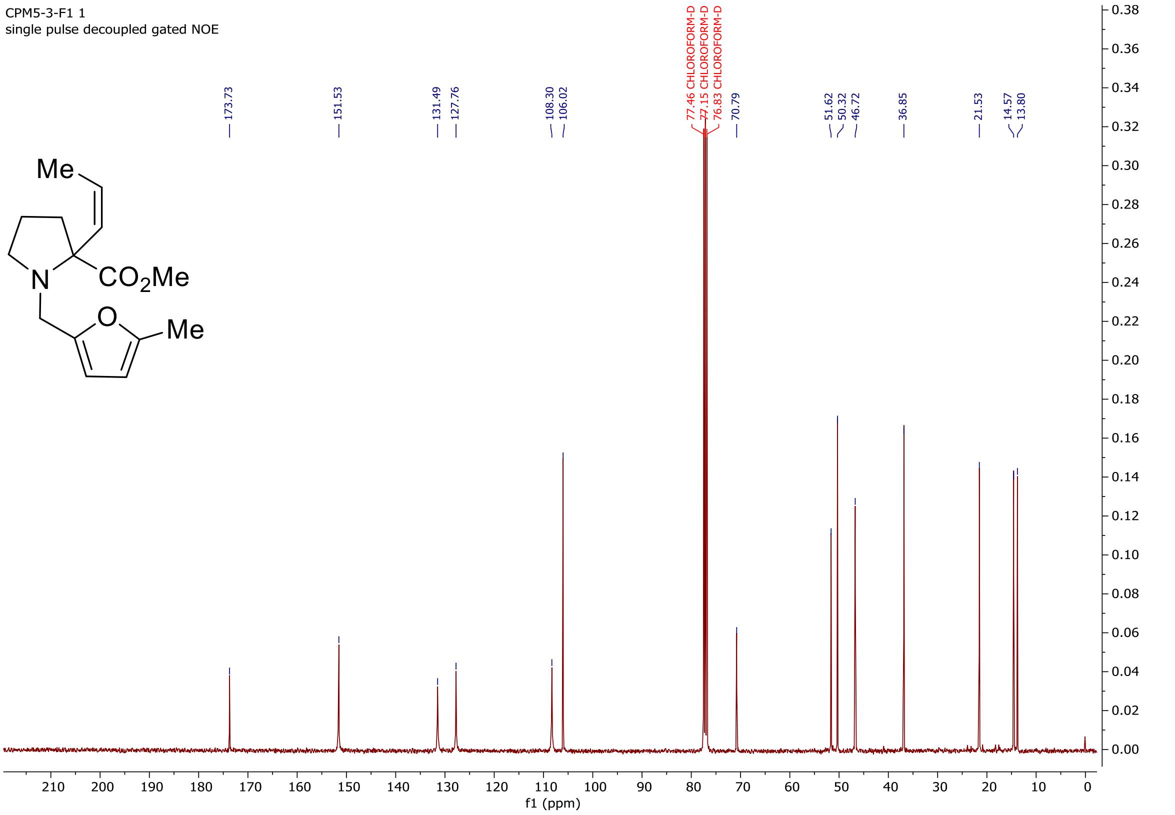

^{1}H NMR ($CDCl_3$ 400 MHz) of compound 7m

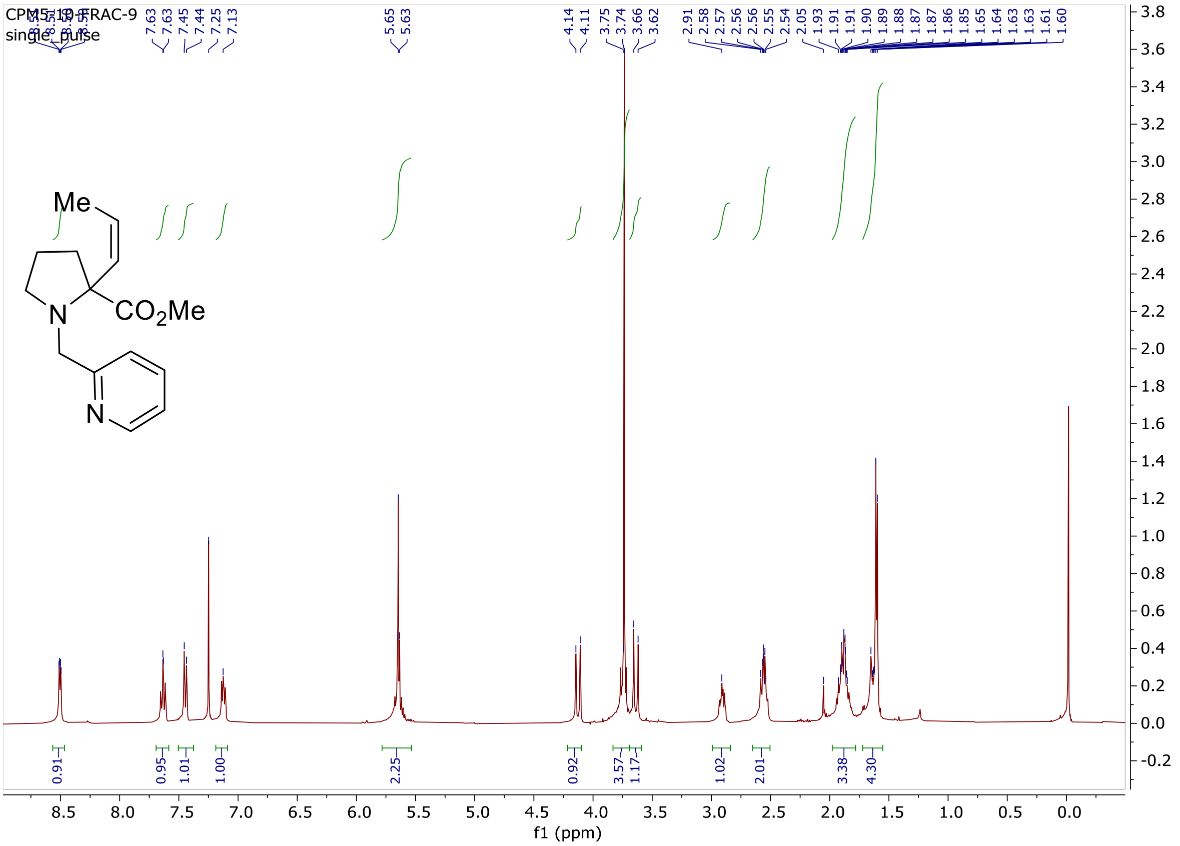

^{13}C NMR ($CDCl_3$ ($CDCl_3$ 100 MHz) of compound 7m

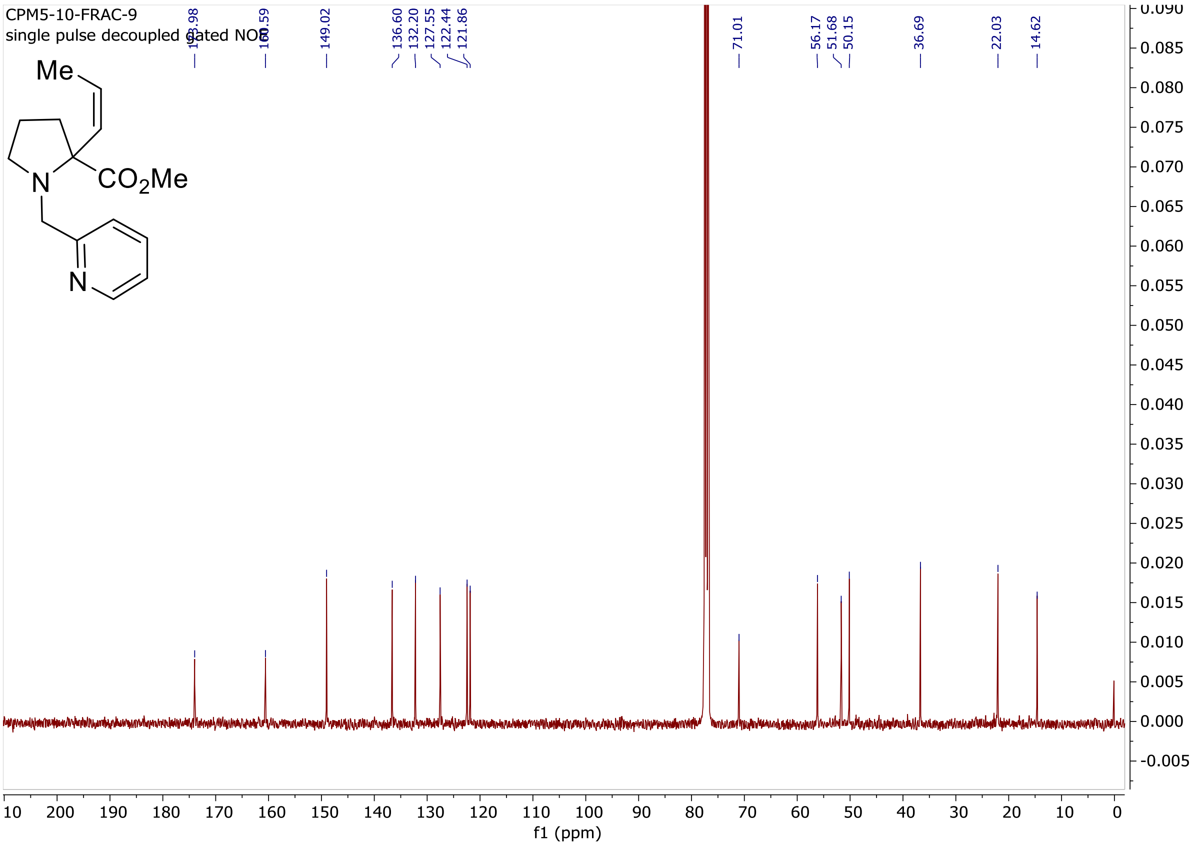

^{1}H NMR ($CDCl_3$ 400 MHz) of compound 7n

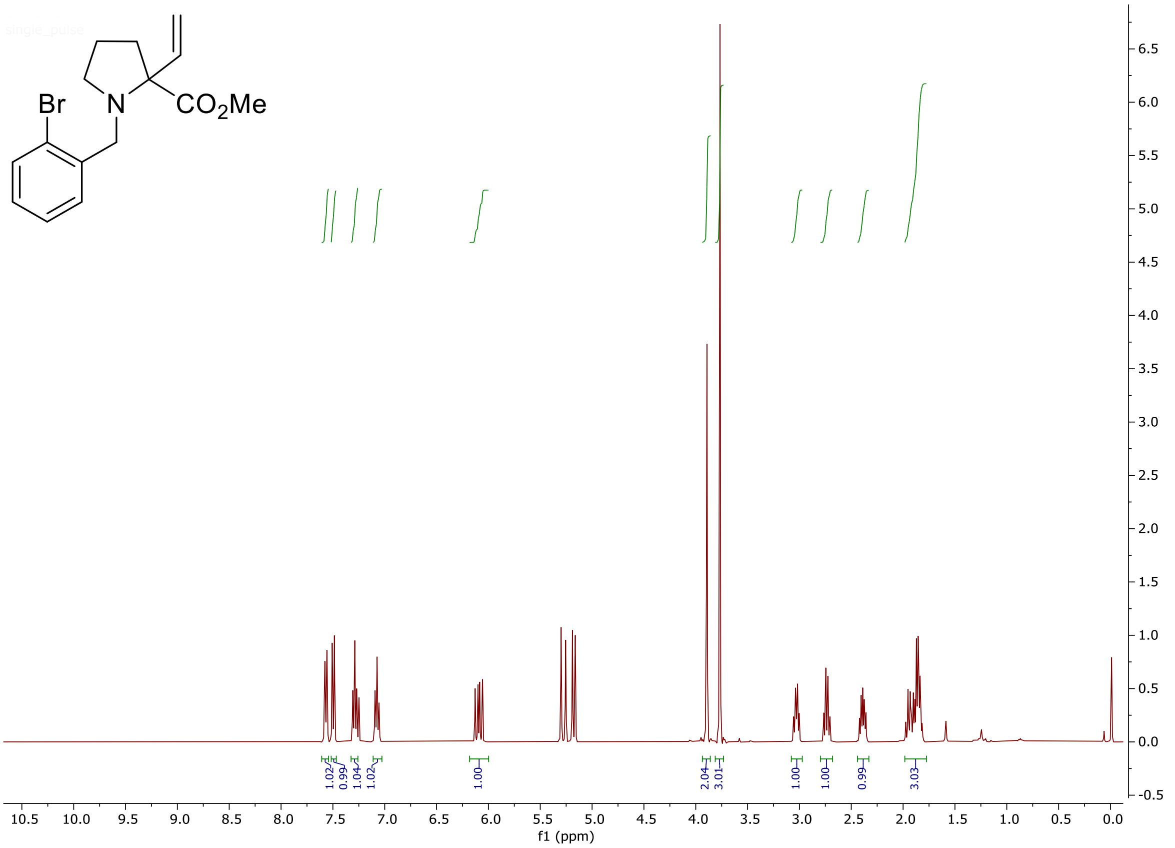

^{13}C NMR ($CDCl_3$ 100 MHz) of compound 7n

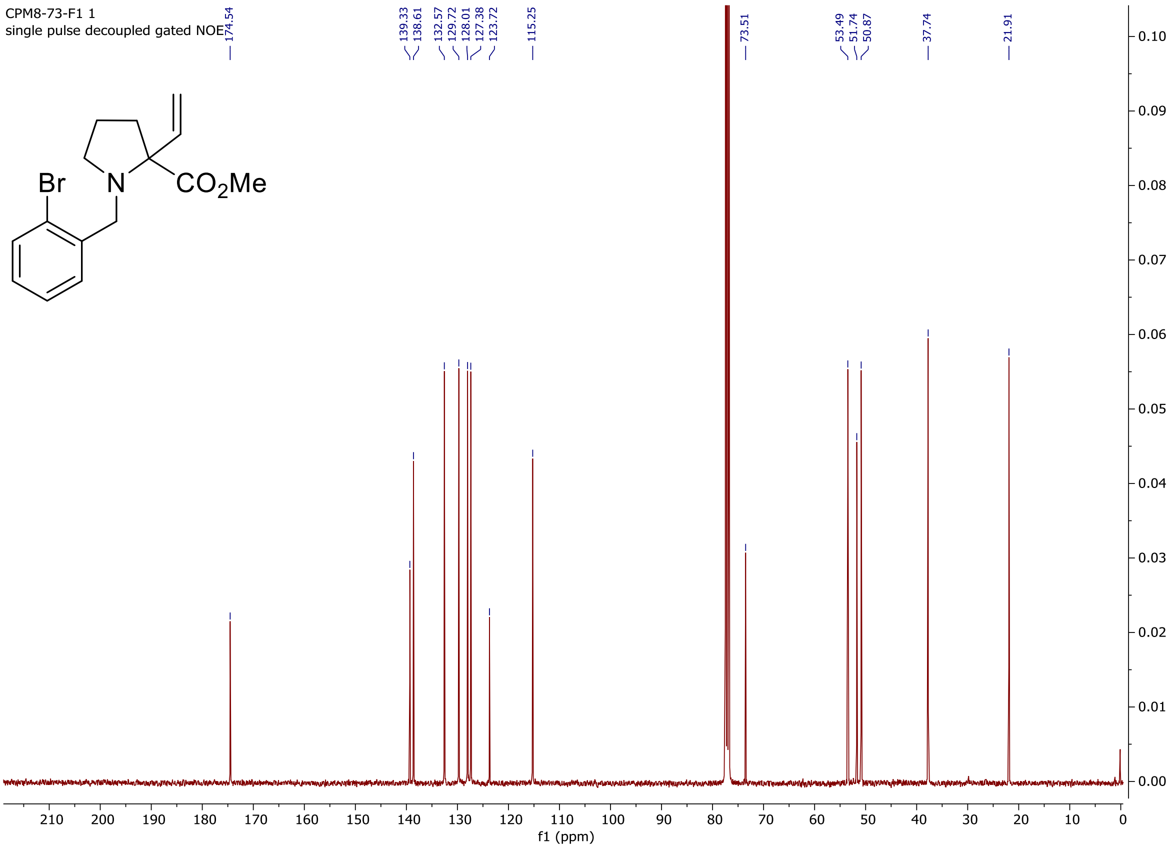

^{1}H NMR ($CDCl_3$ 400 MHz) of compound S18

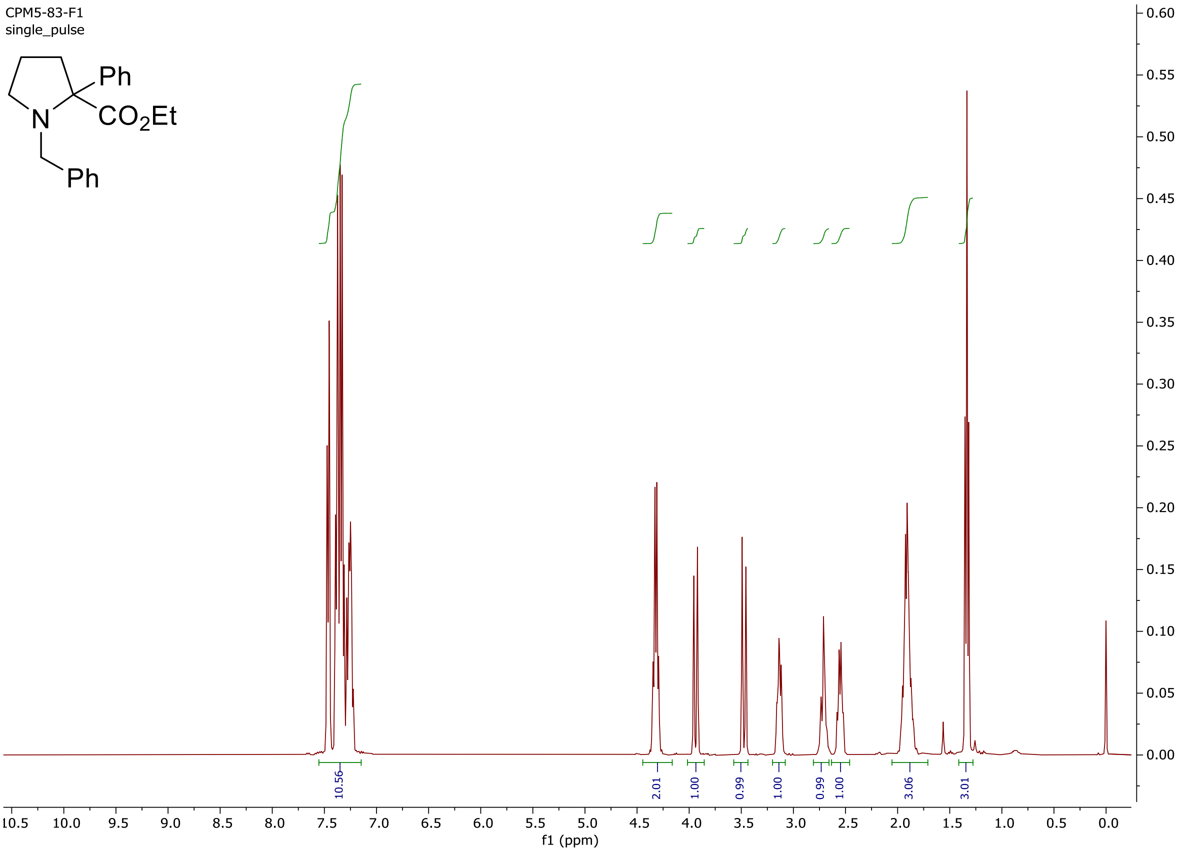

^{13}C NMR ($CDCl_3$ 100 MHz) of compound S18

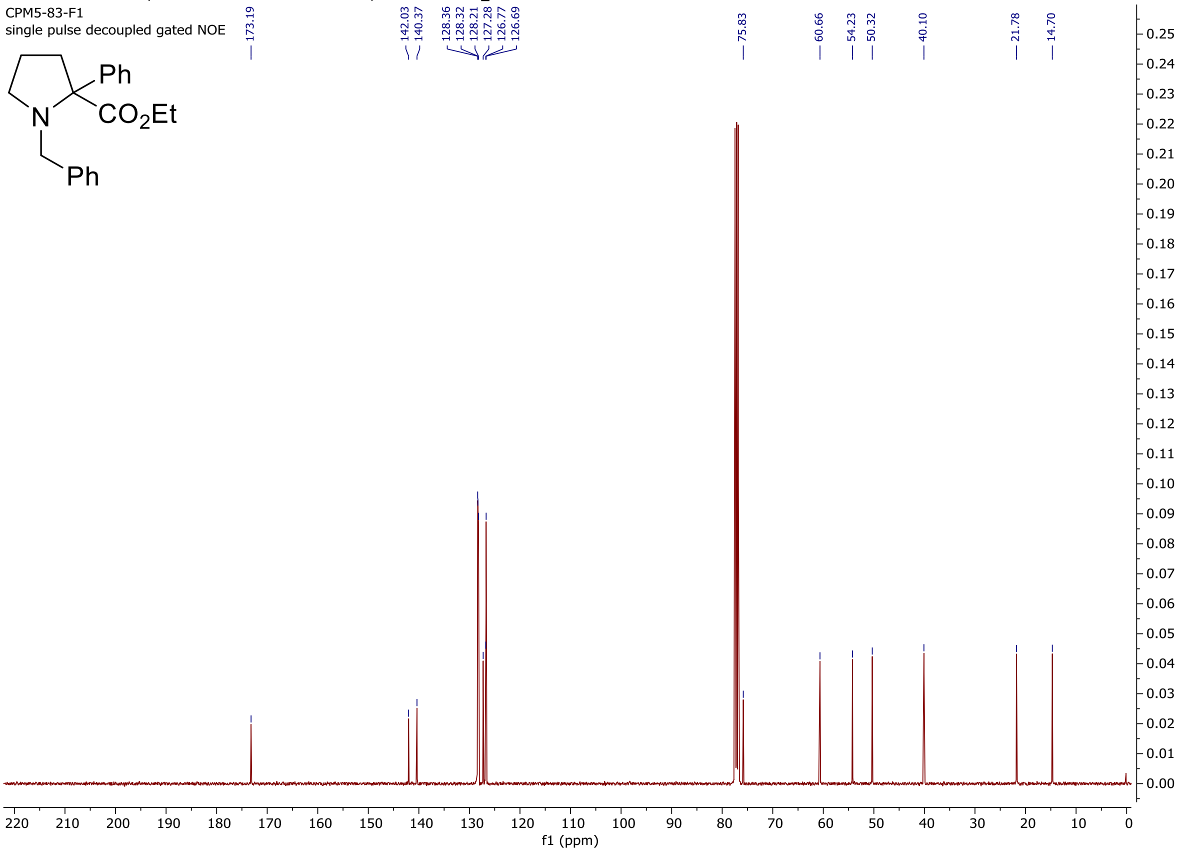

^{1}H NMR ($CDCl_3$ 400 MHz) of compound 7g

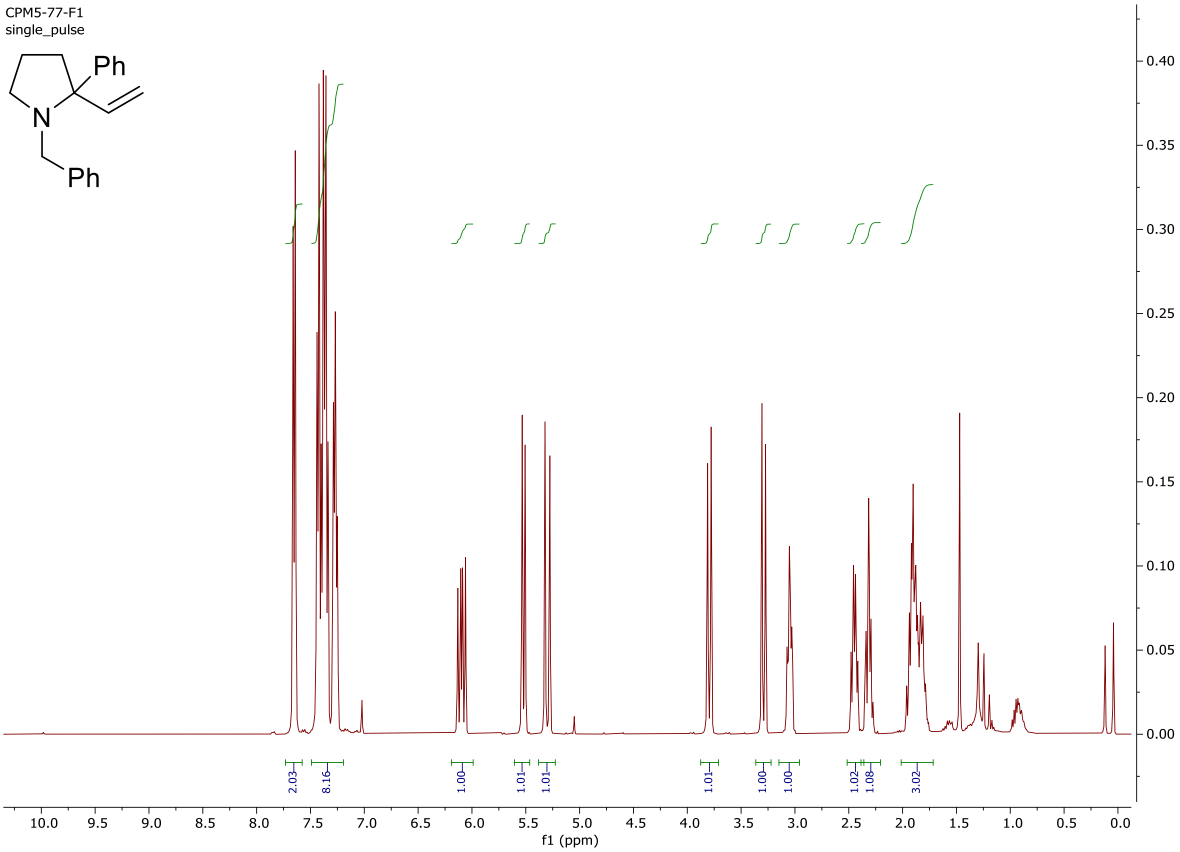

^{13}C NMR ($CDCl_3$ 100 MHz) of compound 7g

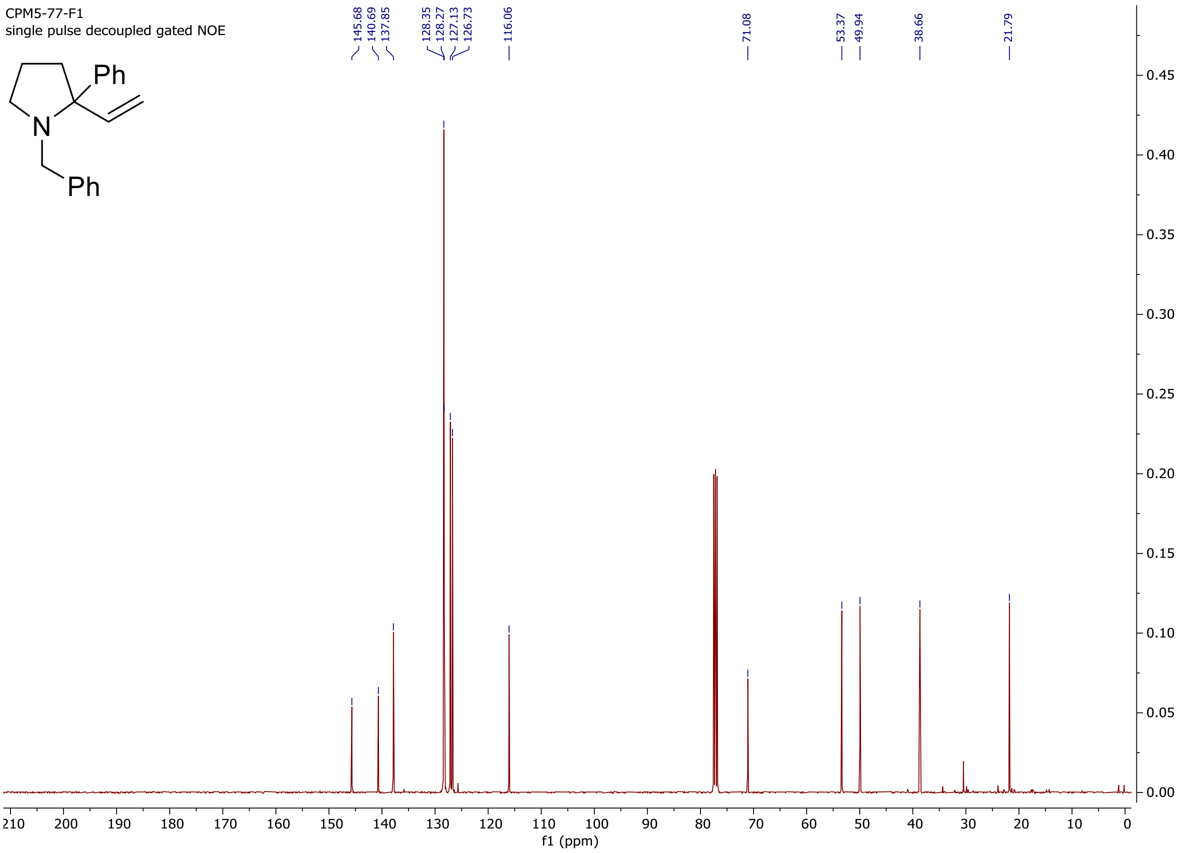

^{1}H NMR ($CDCl_3$ 400 MHz) of compound *Z*-7h

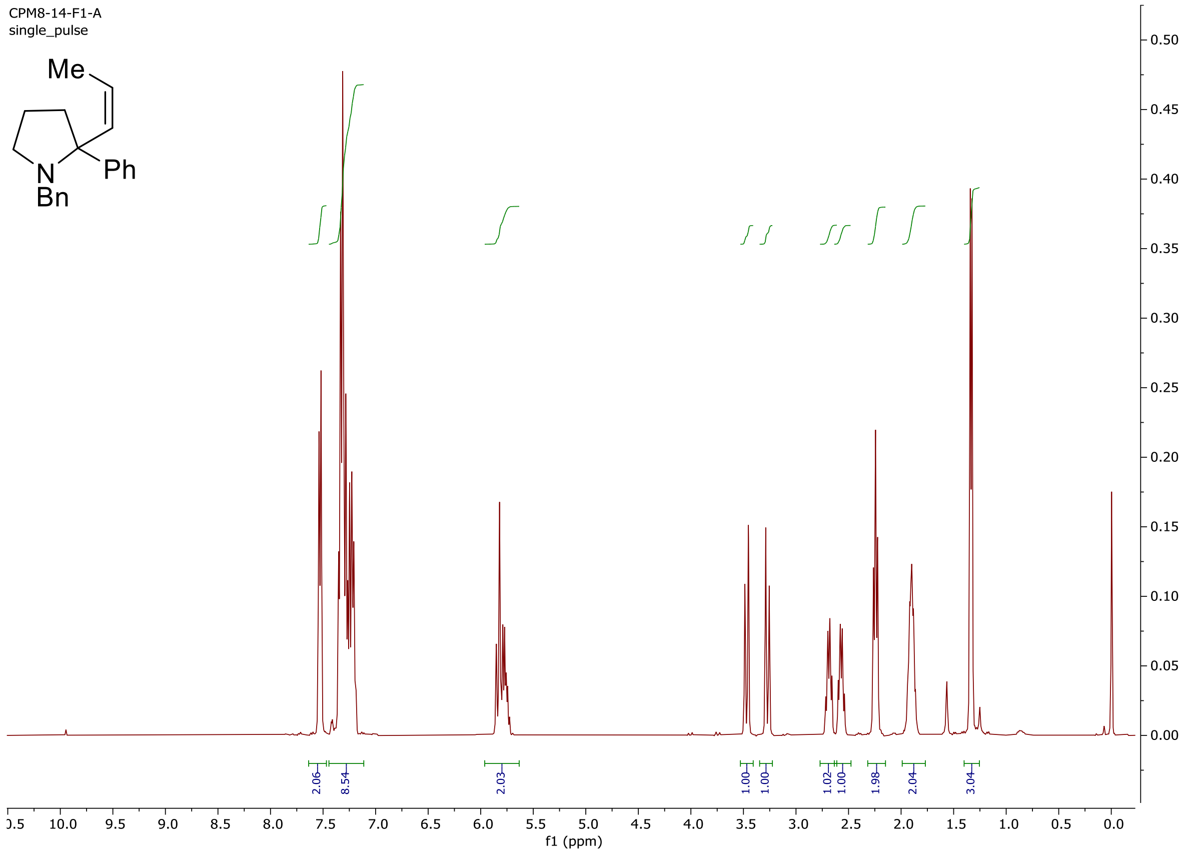

^{13}C NMR ($CDCl_3$ 100 MHz) of compound *Z*-7h

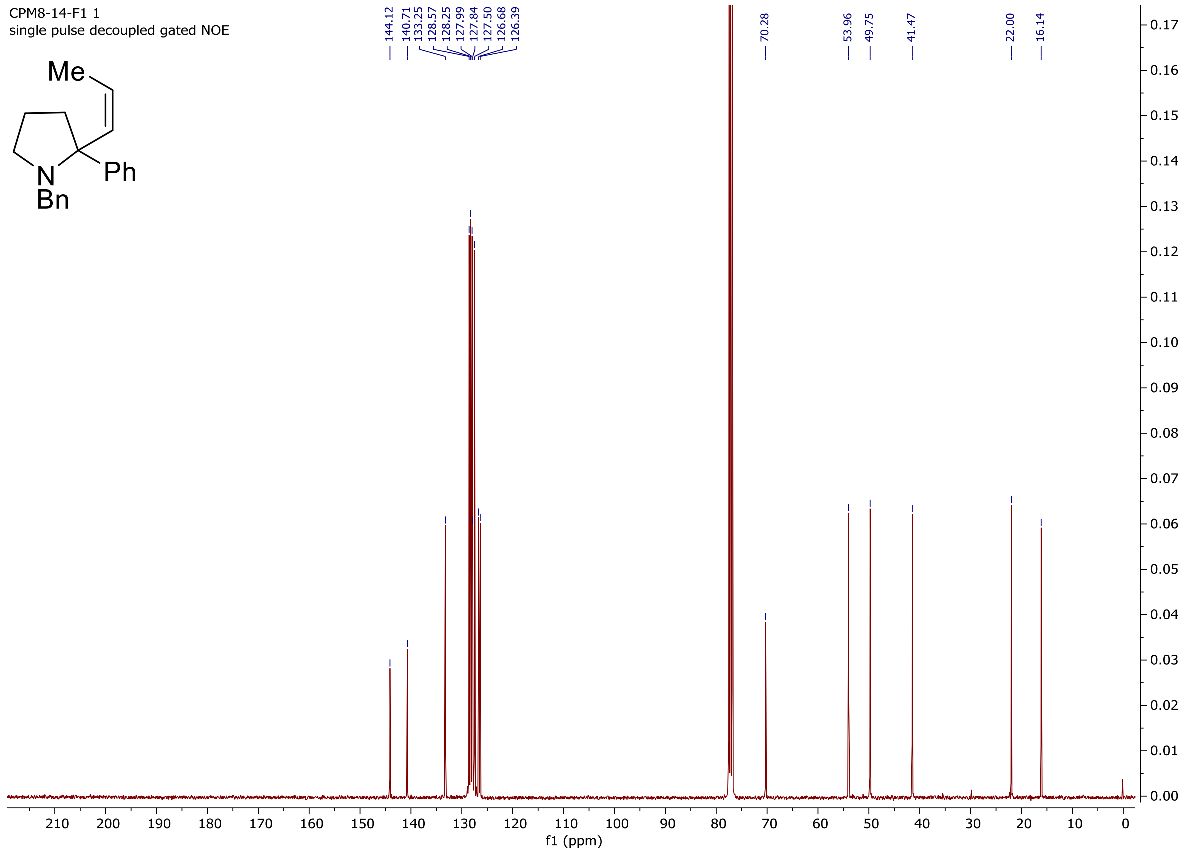

^{1}H NMR (CDCl₃ 400 MHz) of compound *E*-7h

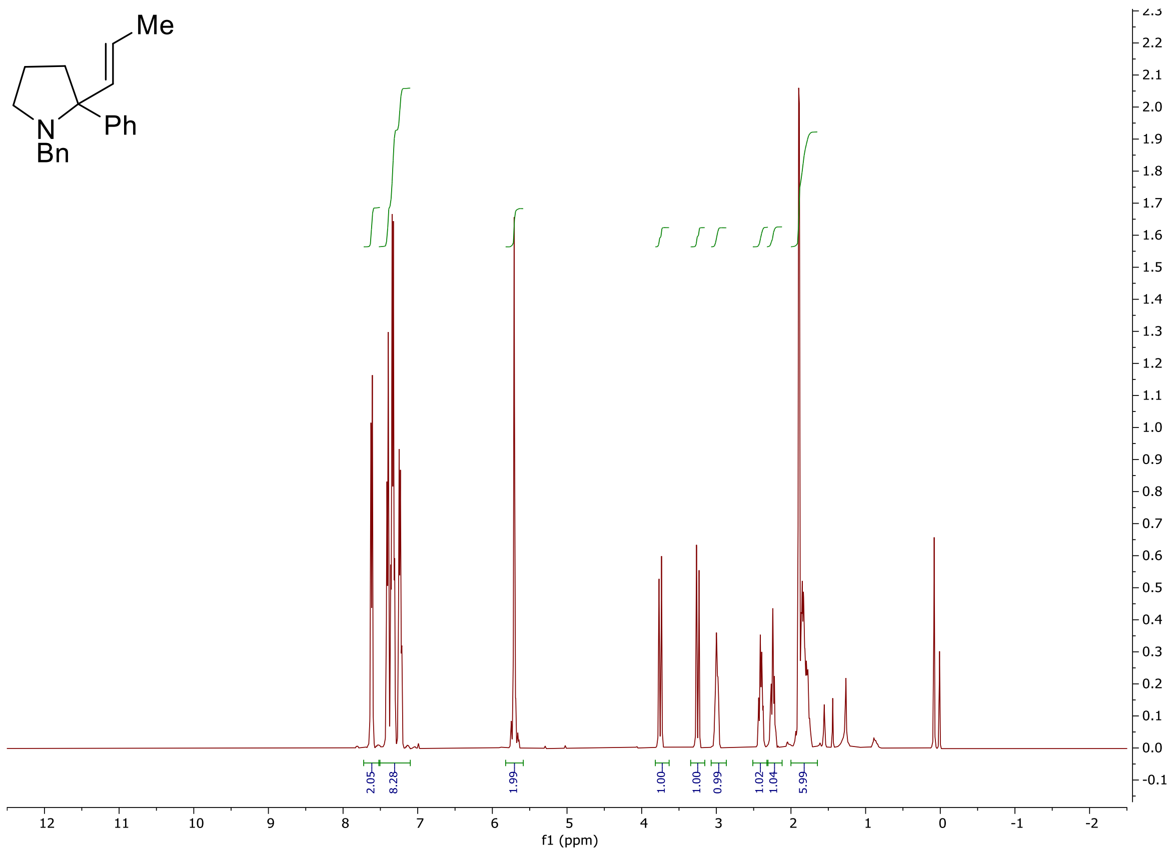

^{13}C NMR (CDCl₃ 100 MHz) of compound *E*-7h

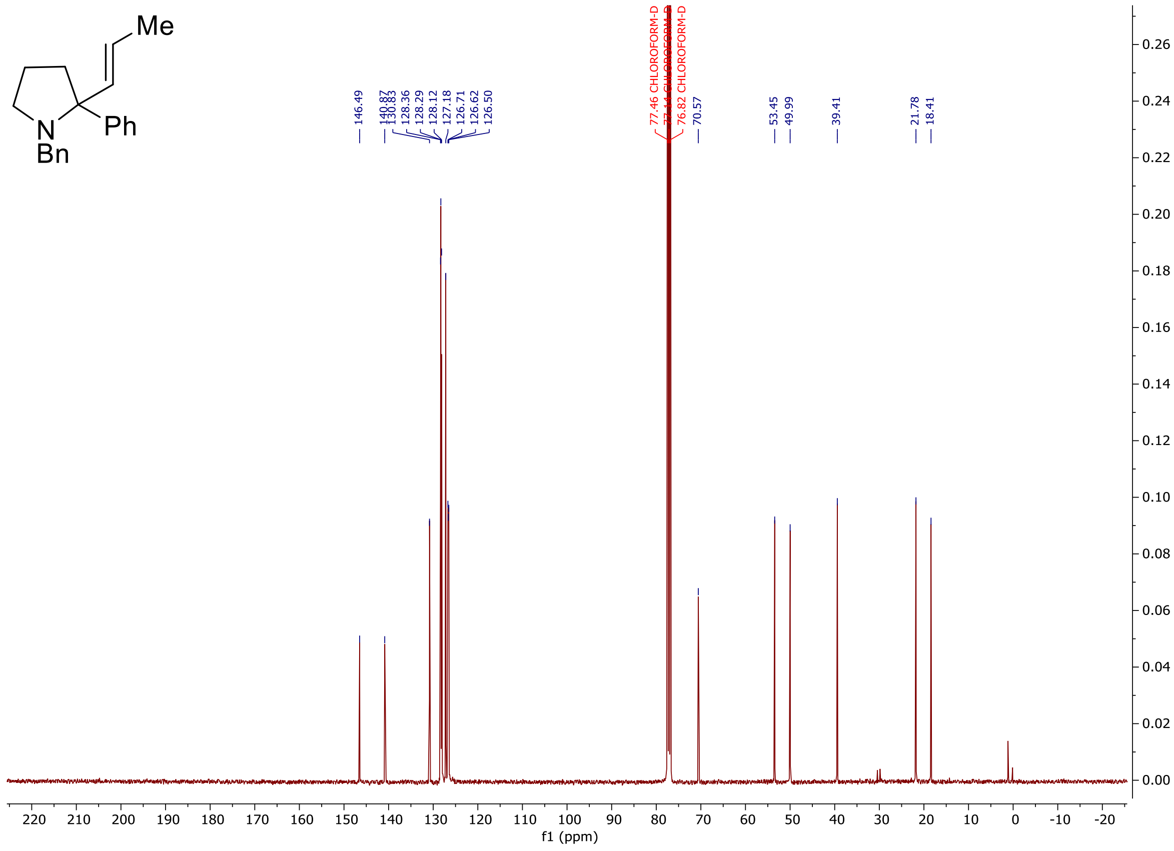

^{1}H NMR ($CDCl_3$ 400 MHz) of compound 7i

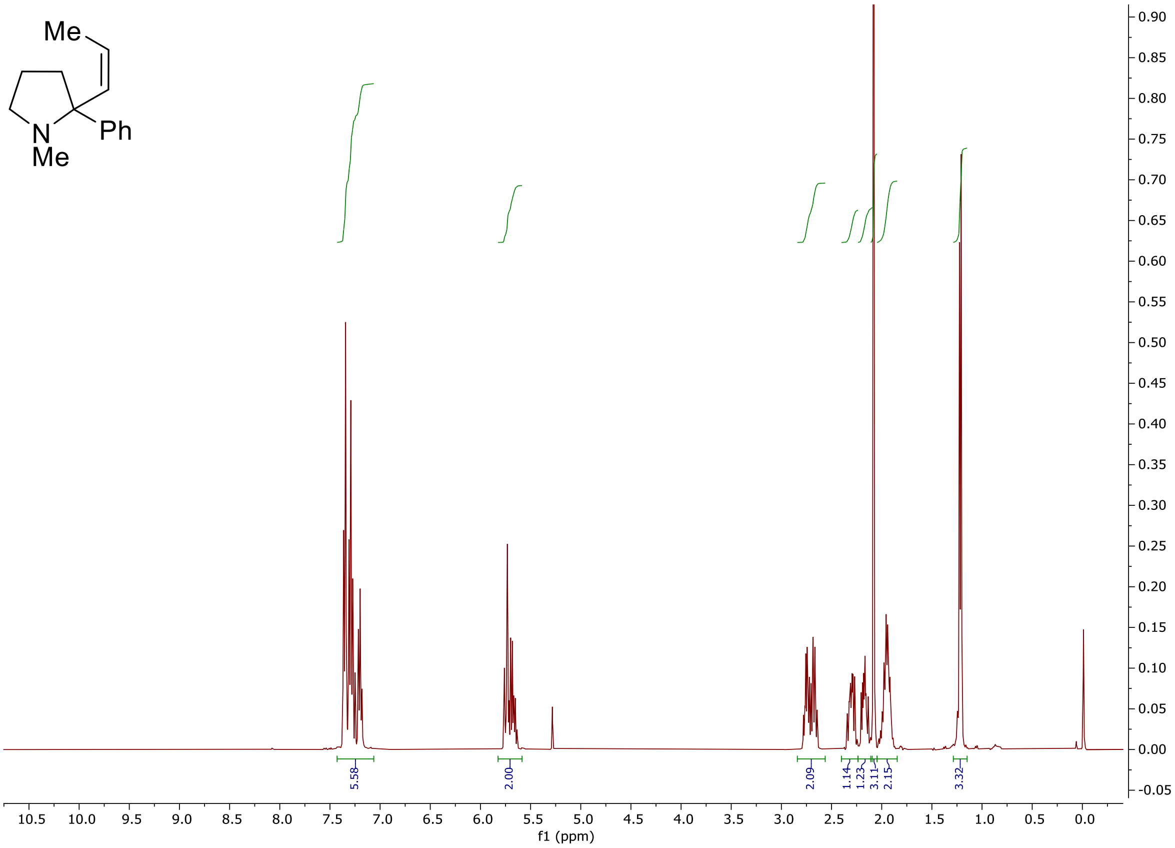

^{13}C NMR ($CDCl_3$ 100 MHz) of compound 7i

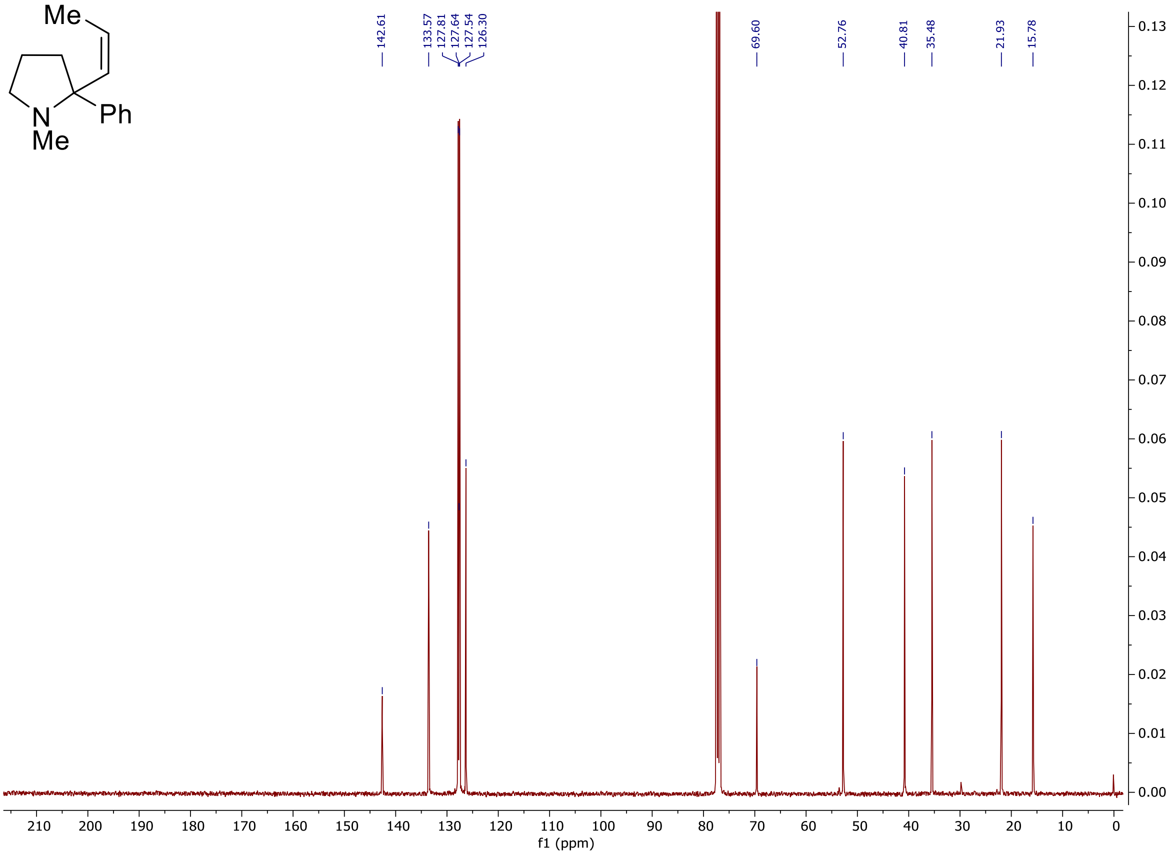

^{1}H NMR (CDCl$_3$ 400 MHz) of compound 7j

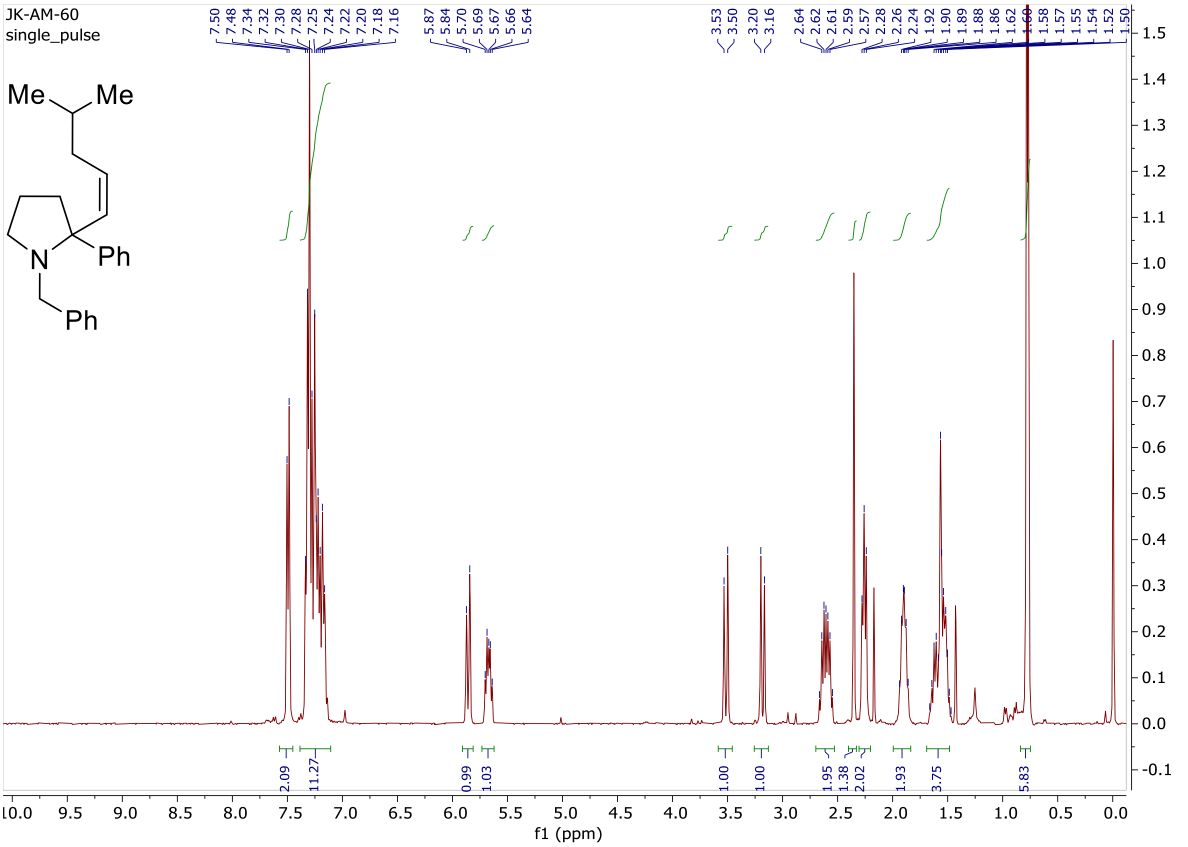

^{13}C NMR (CDCl$_3$ 100 MHz) of compound 7j

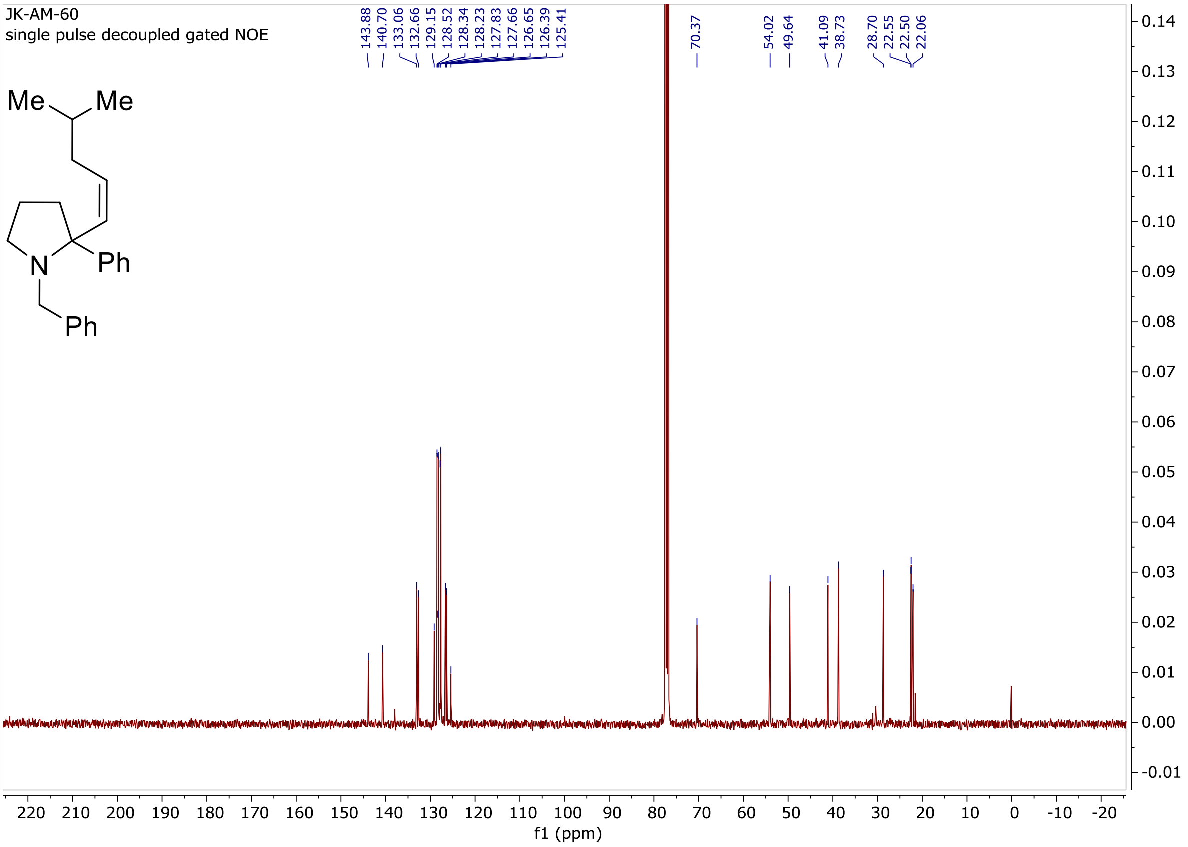

^{1}H NMR (CDCl$_3$ 400 MHz) of compound S19

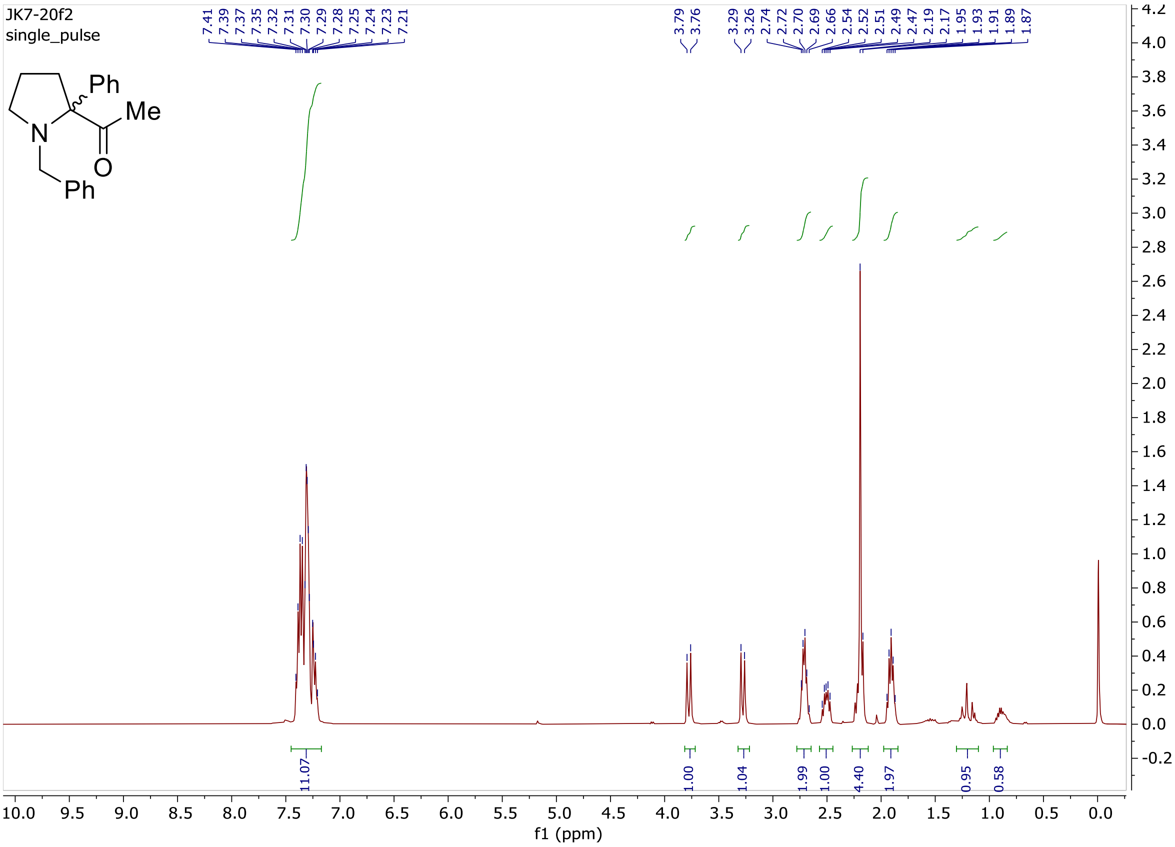

^{13}C NMR (CDCl$_3$ 100 MHz) of compound S19

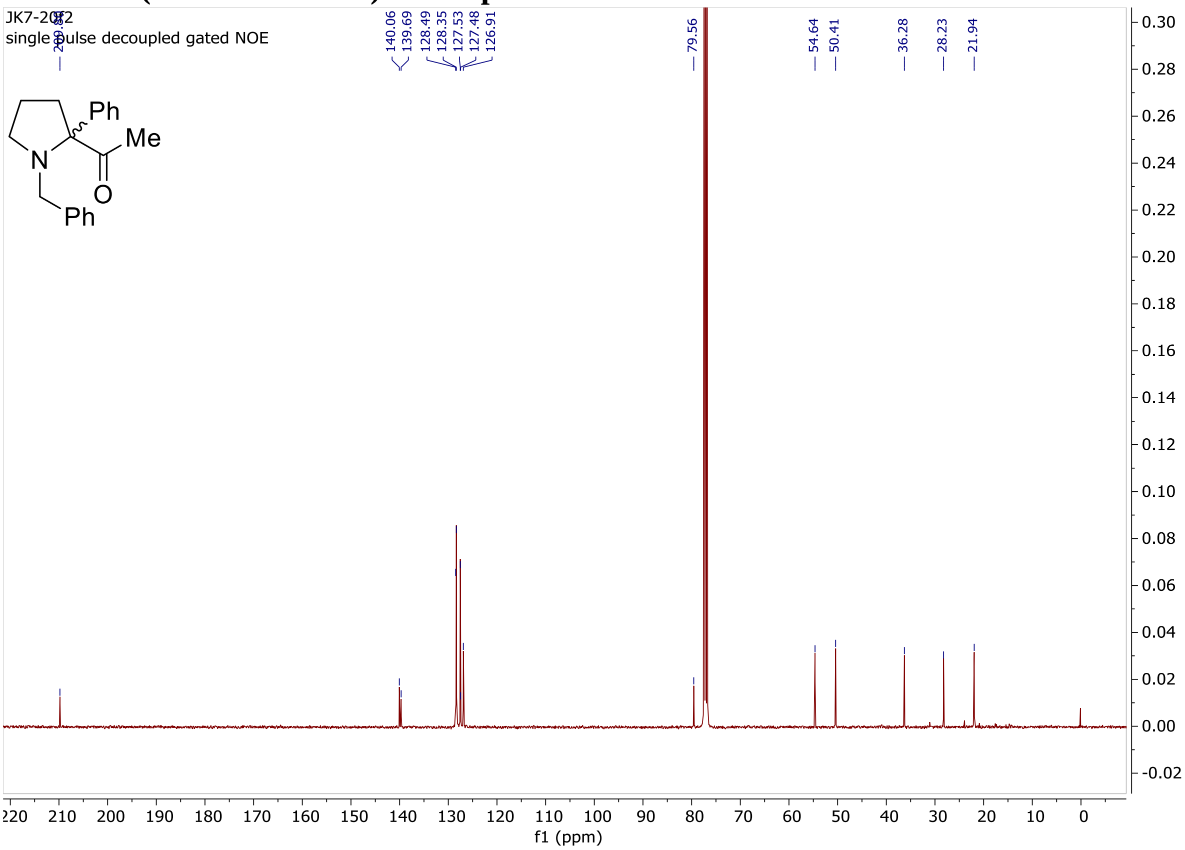

^{1}H NMR ($CDCl_3$ 400 MHz) of compound 7k

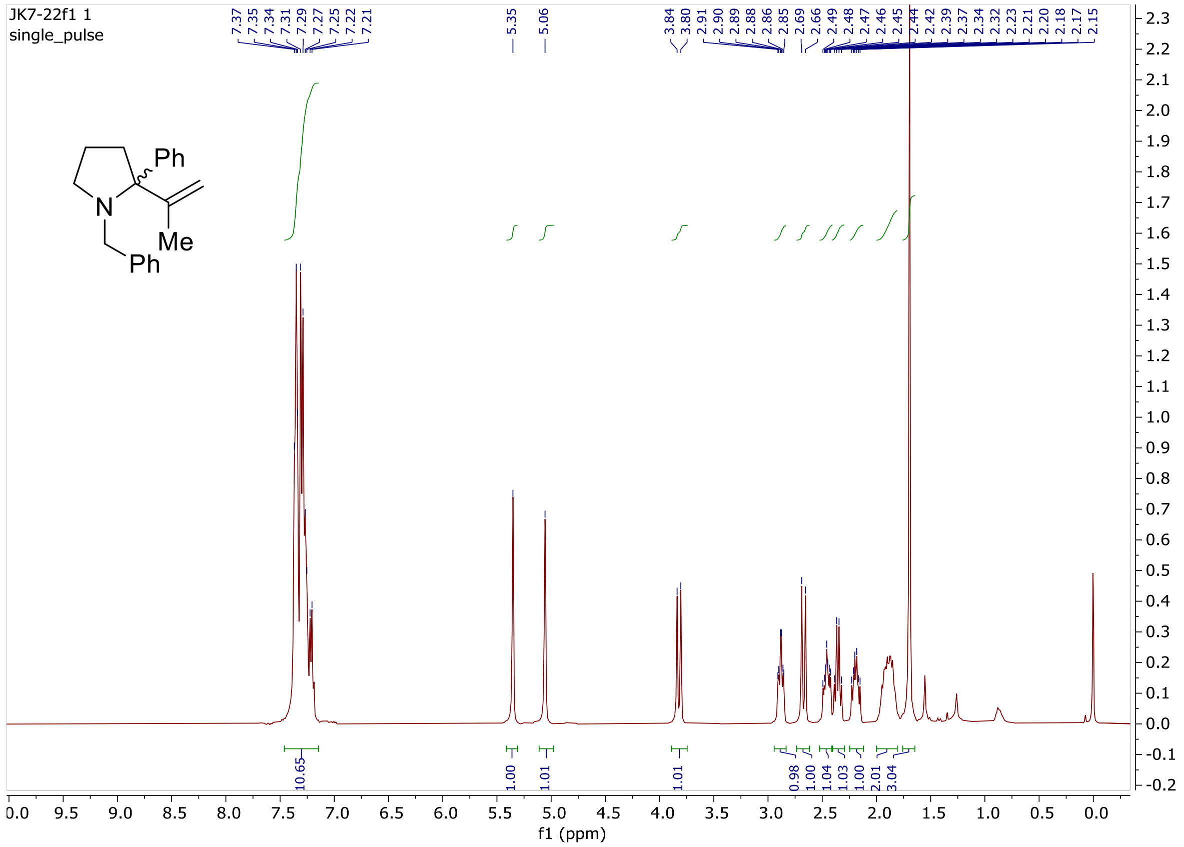

^{13}C NMR ($CDCl_3$ 100 MHz) of compound 7k

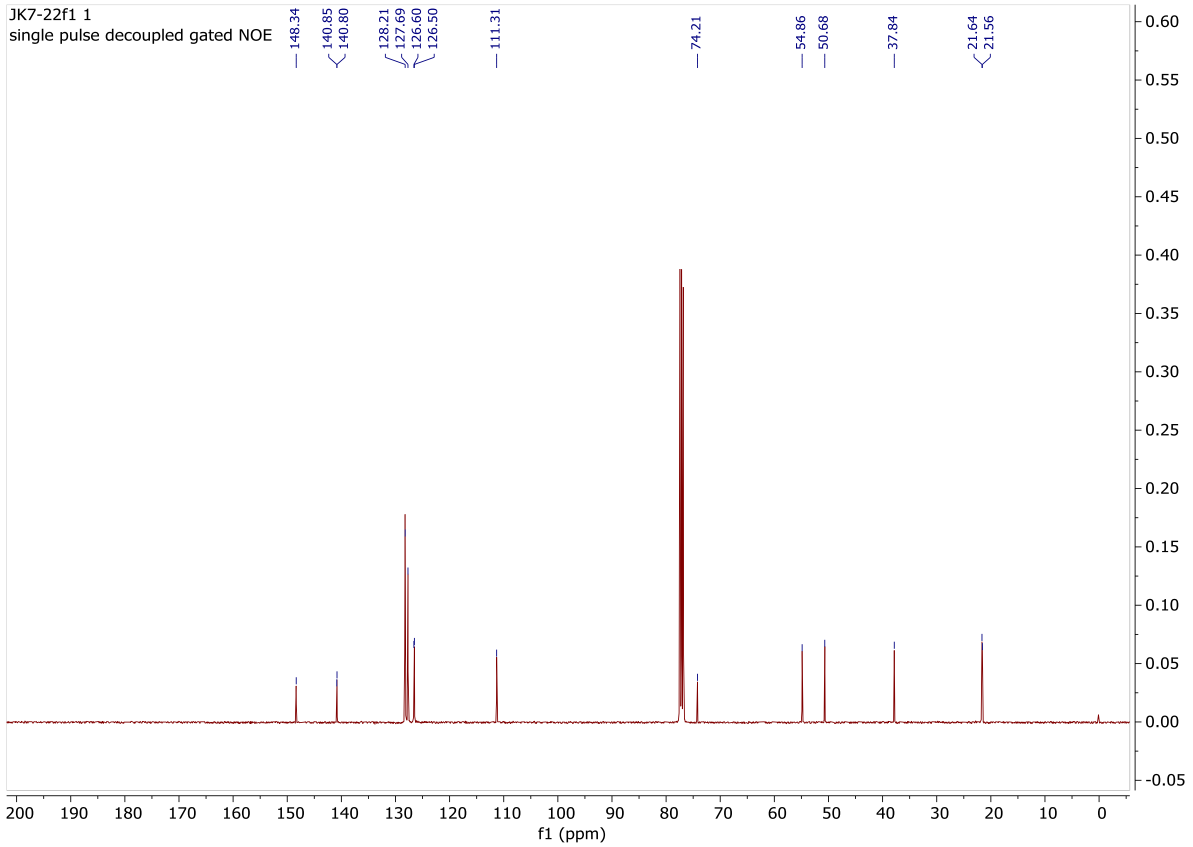

^{1}H NMR (CDCl$_3$ 400 MHz) of compound S25

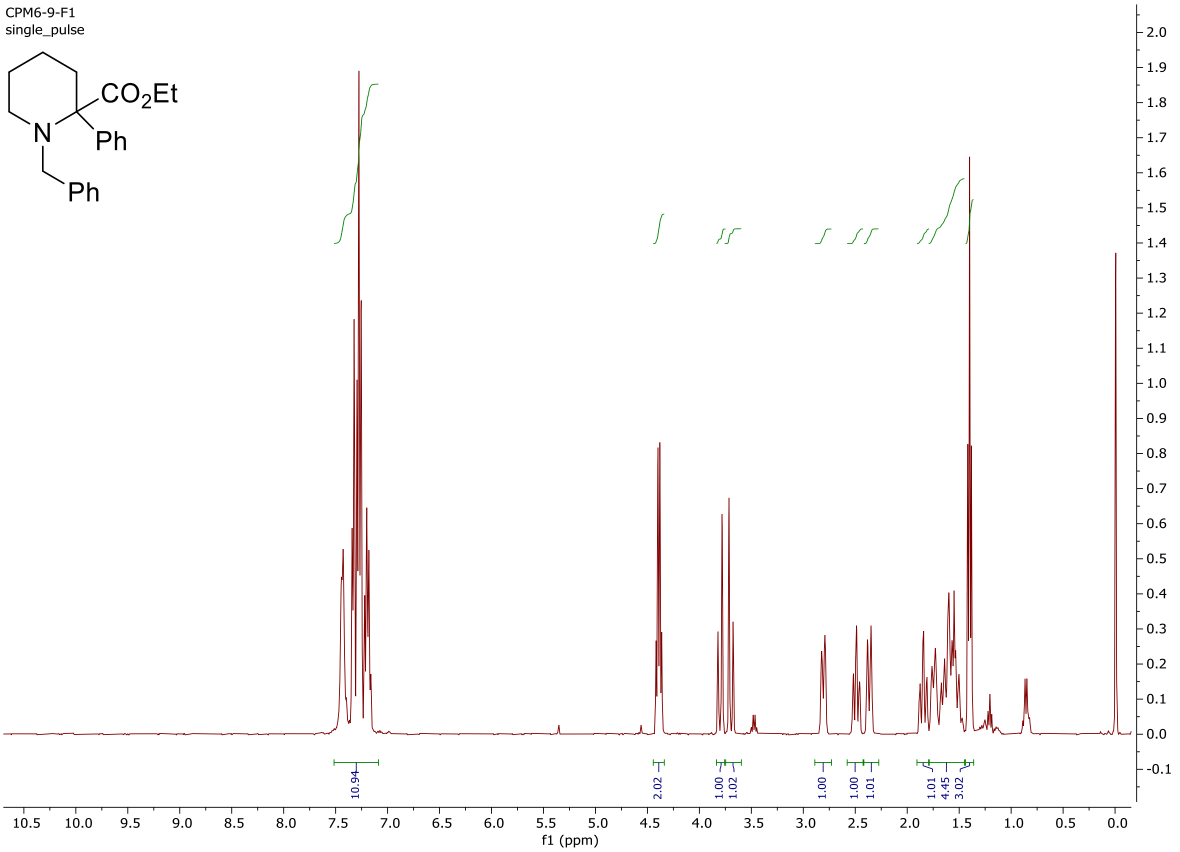

^{13}C NMR (CDCl$_3$ 100 MHz) of compound S25

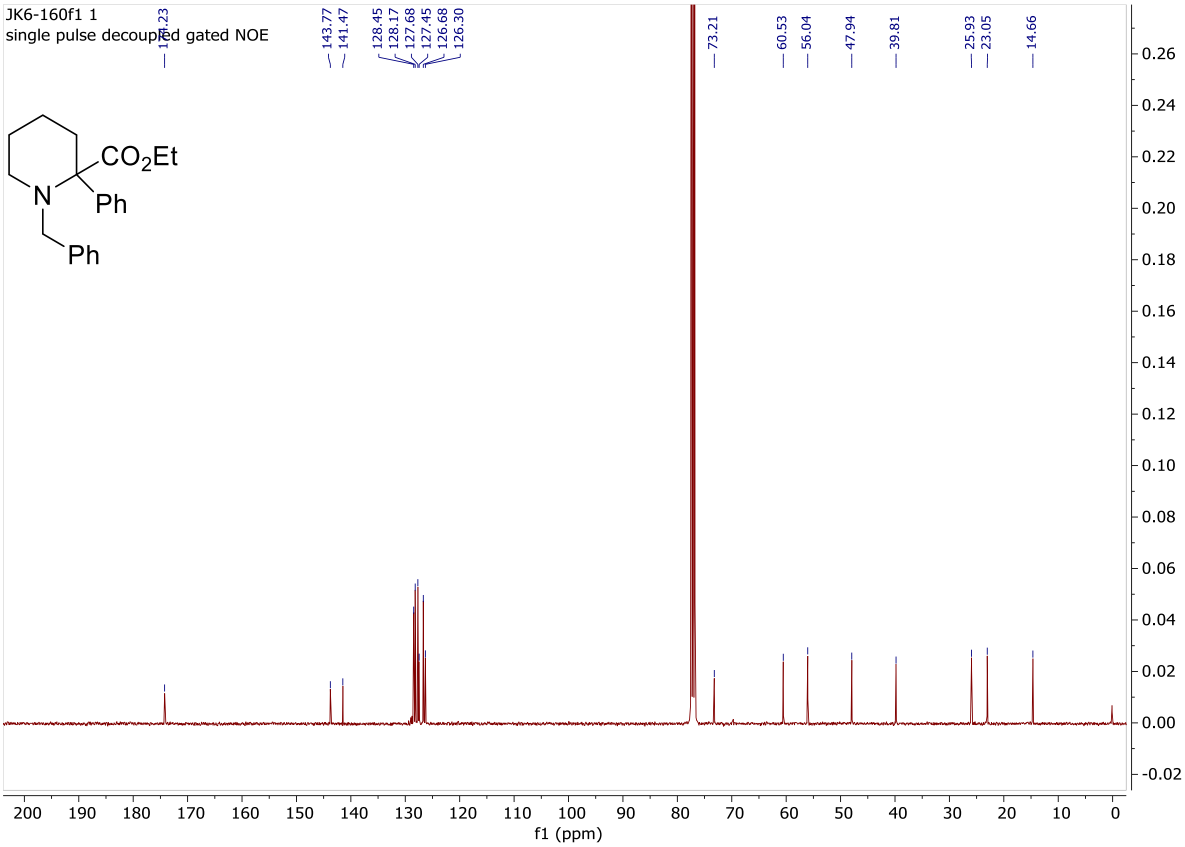

^{1}H NMR ($CDCl_3$ 400 MHz) of compound S22

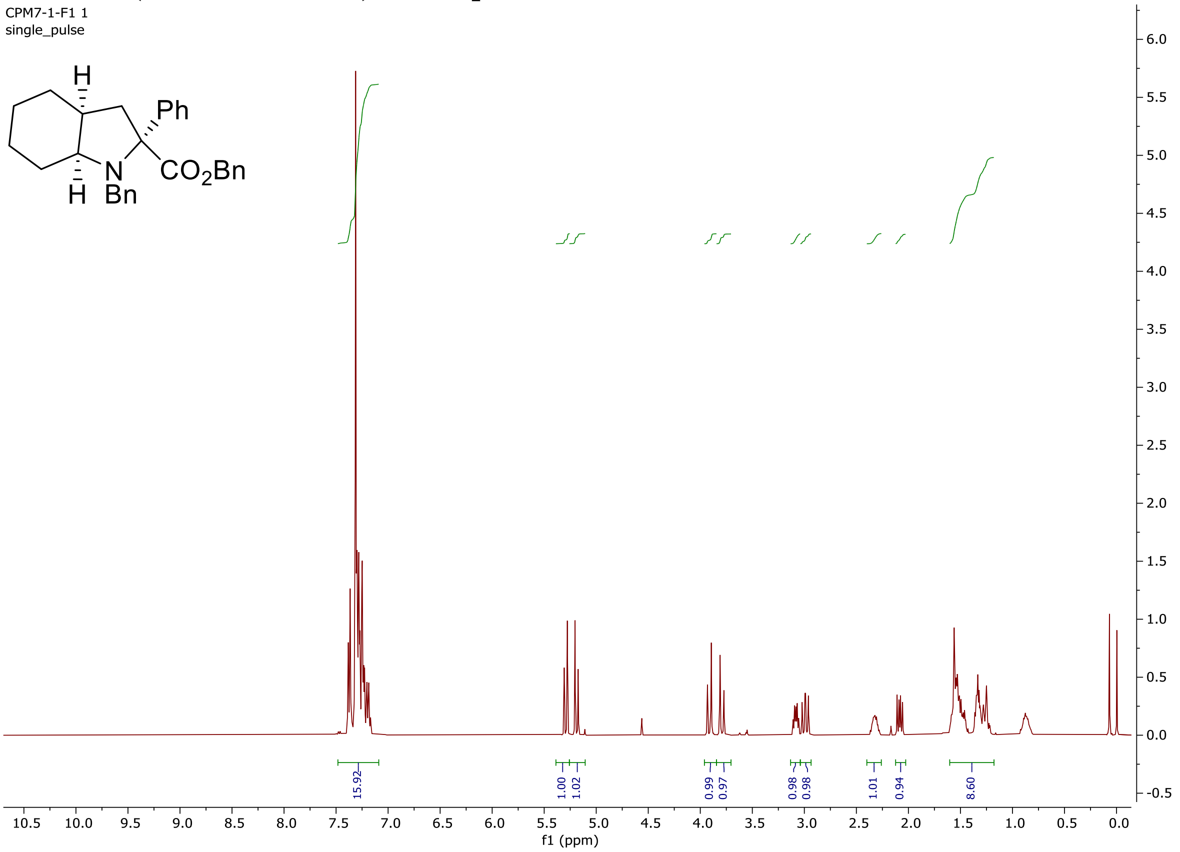

^{13}C NMR ($CDCl_3$ 100 MHz) of compound S22

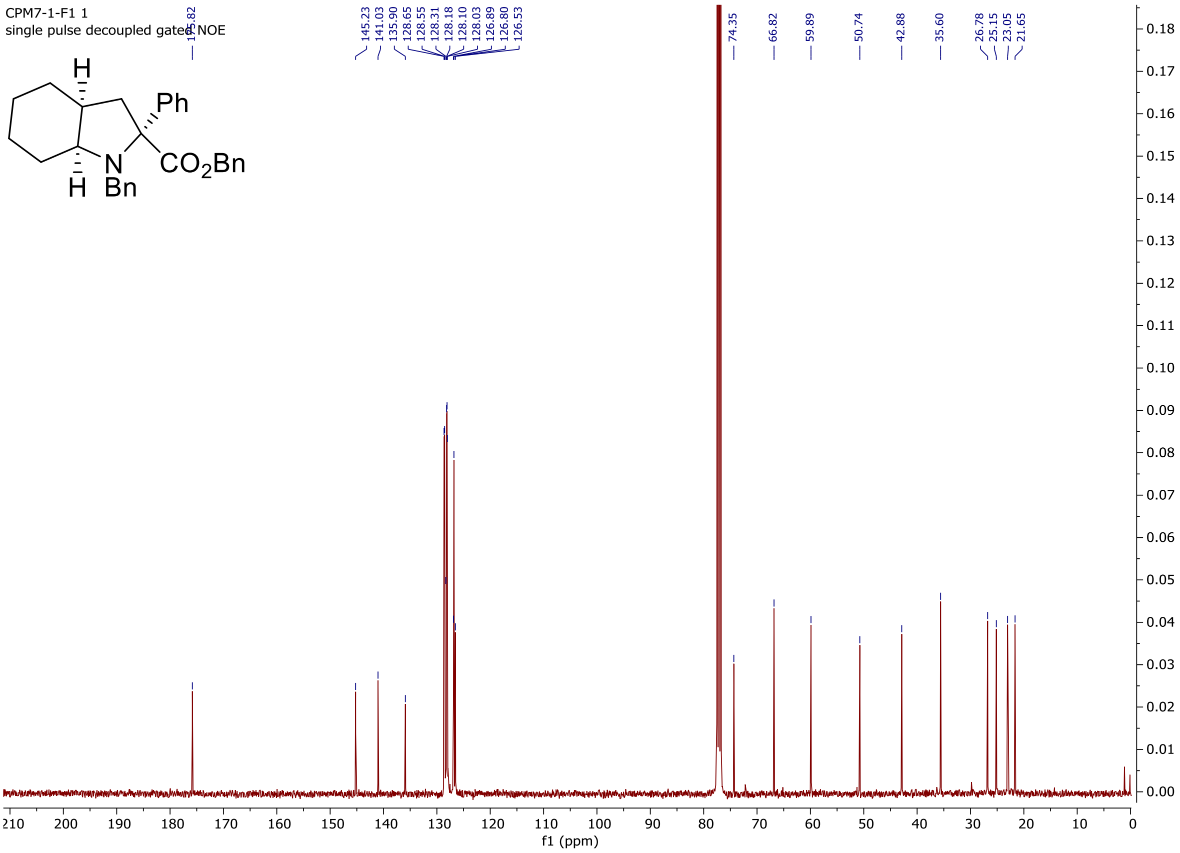

^{1}H NMR ($CDCl_3$ 400 MHz) of compound 7o

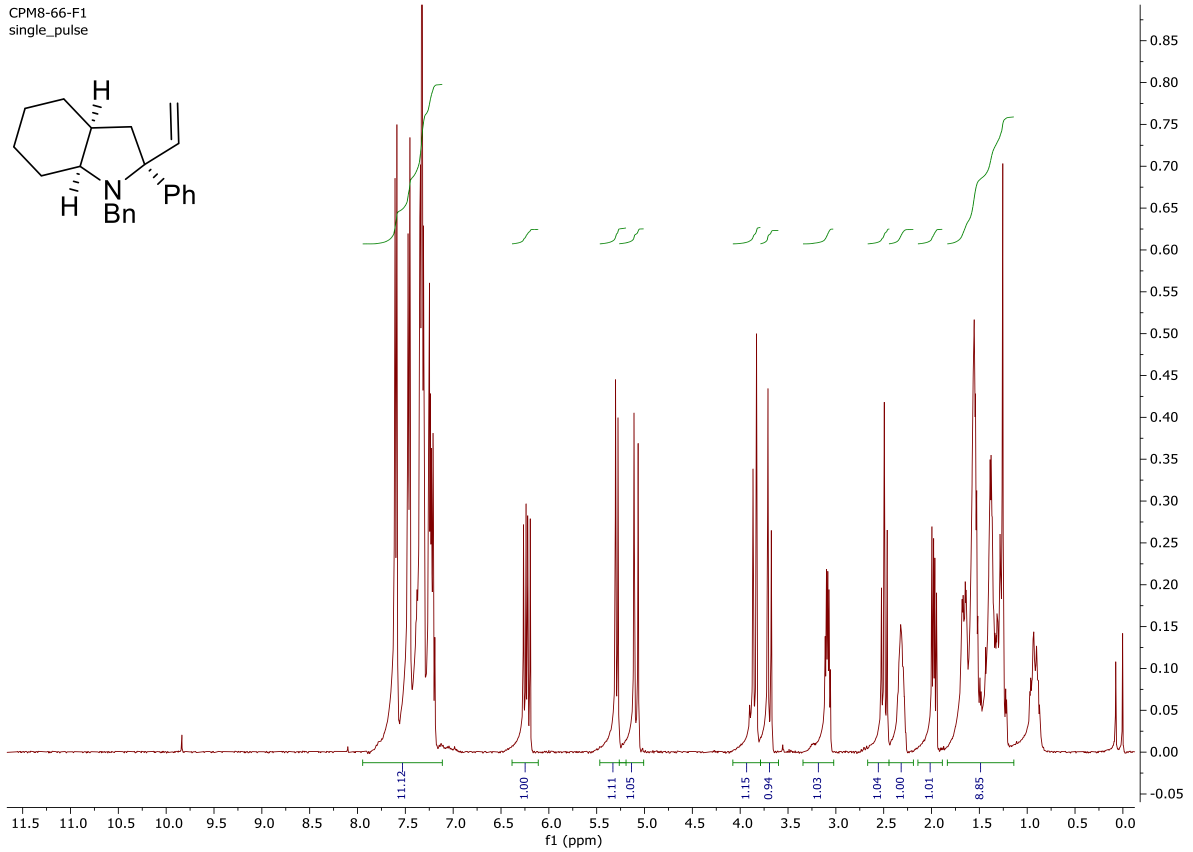

^{13}C NMR ($CDCl_3$ 100 MHz) of compound 7o

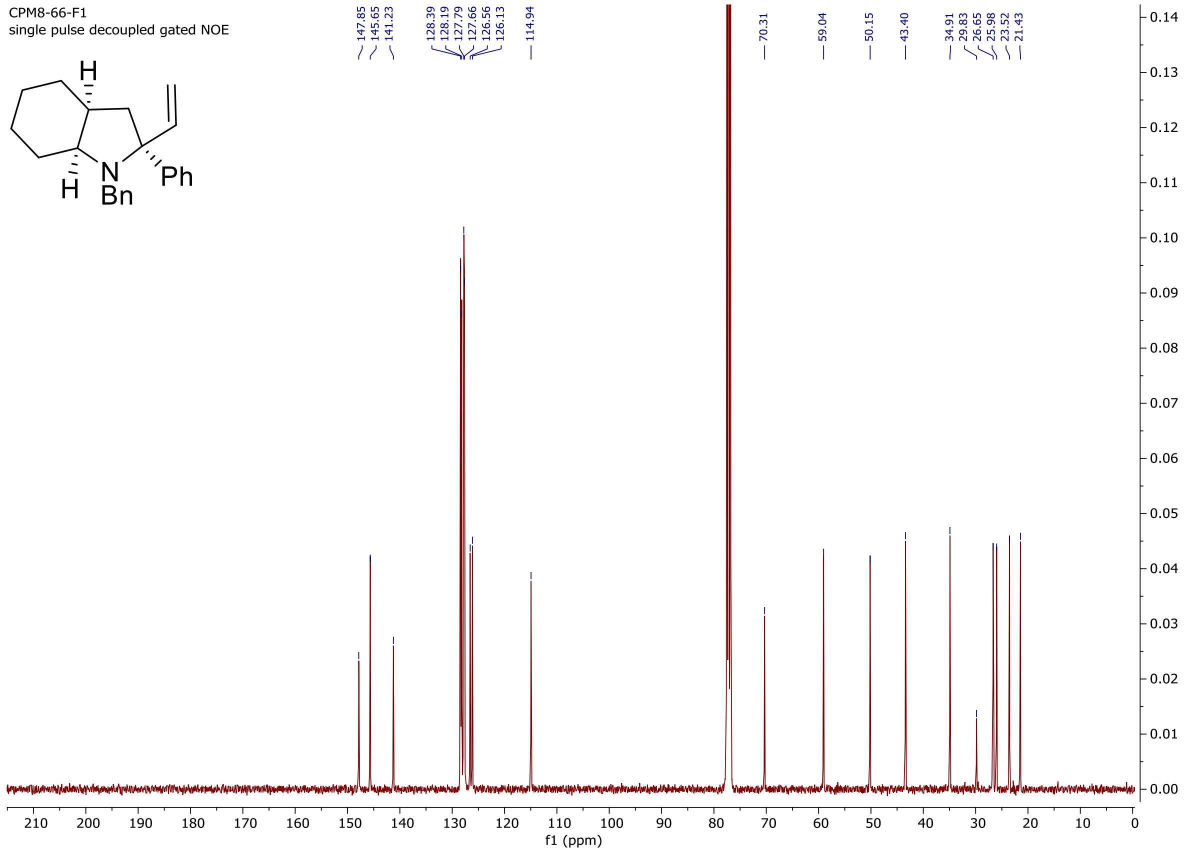

^{1}H NMR ($CDCl_3$ 400 MHz) of compound 7p

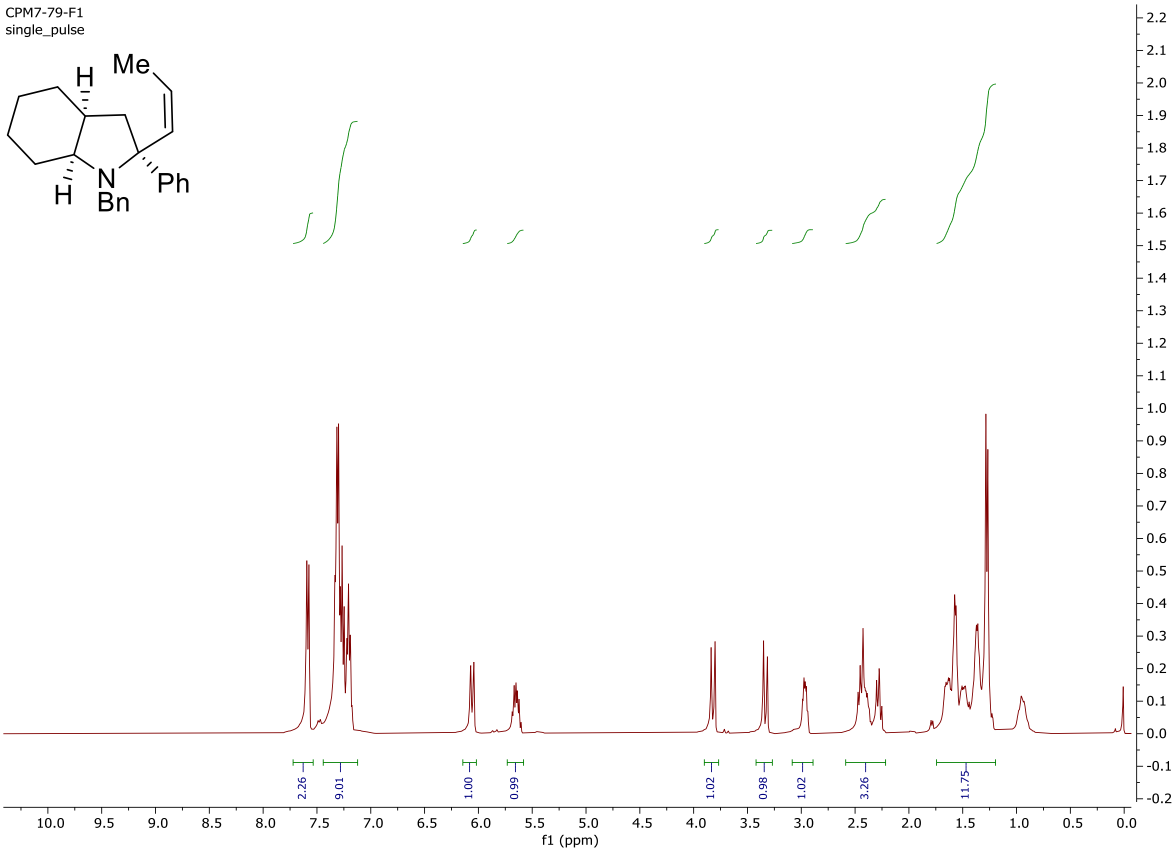

^{13}C NMR ($CDCl_3$ 100 MHz) of compound 7p

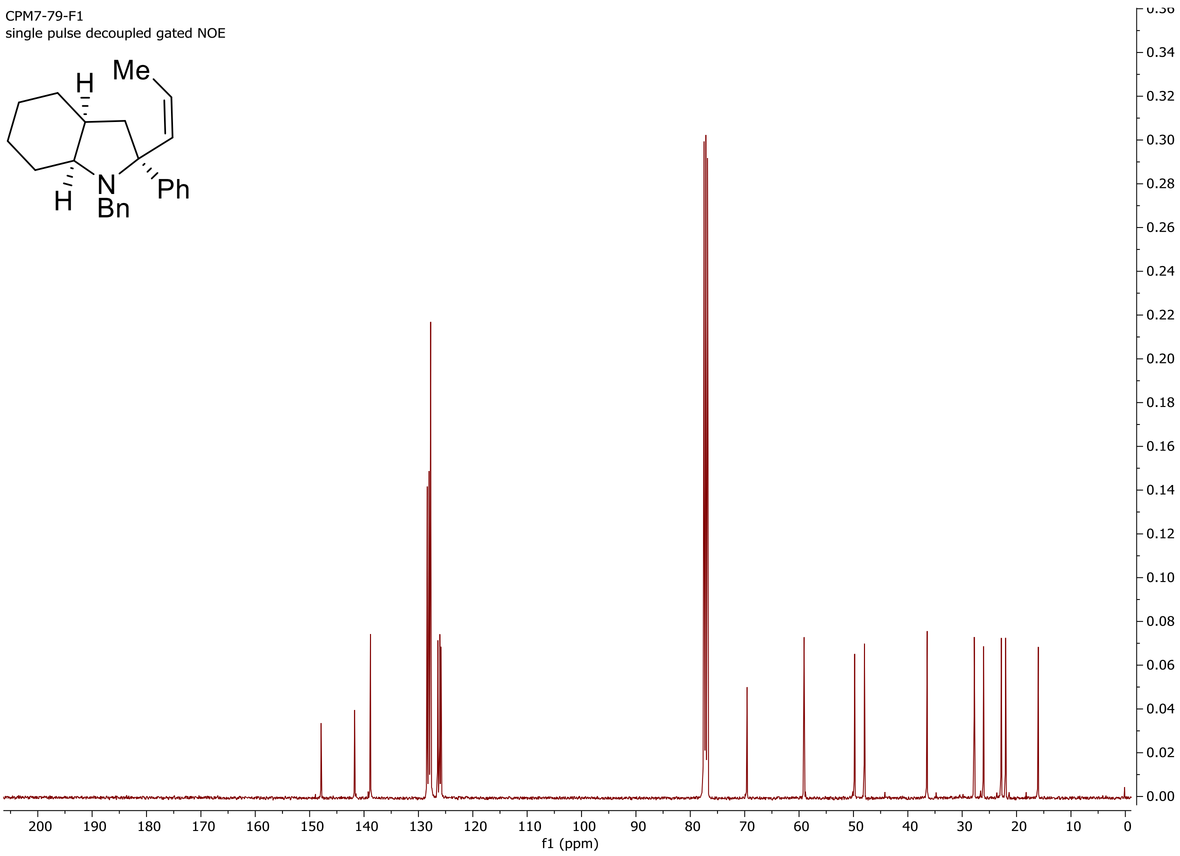

^{1}H NMR ($CDCl_3$ 400 MHz) of compound 7q

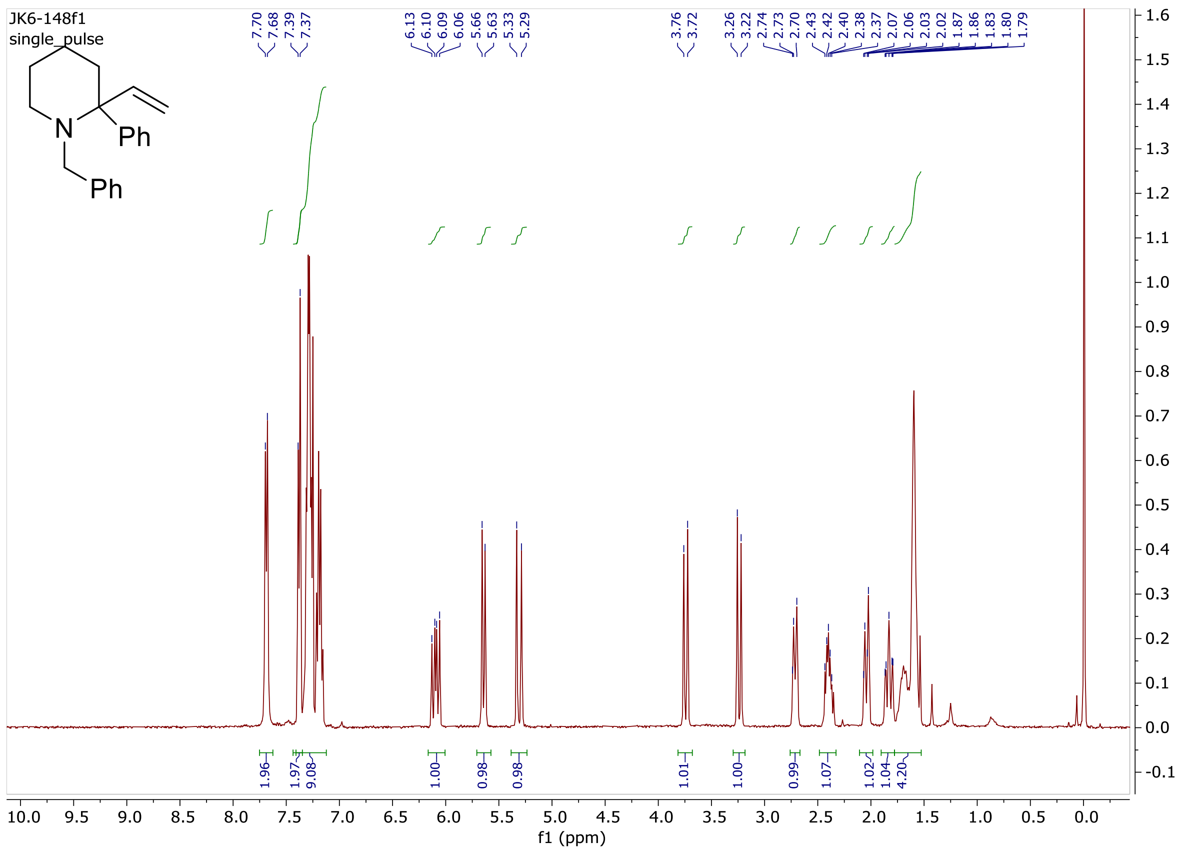

^{13}C NMR ($CDCl_3$ 100 MHz) of compound 7q

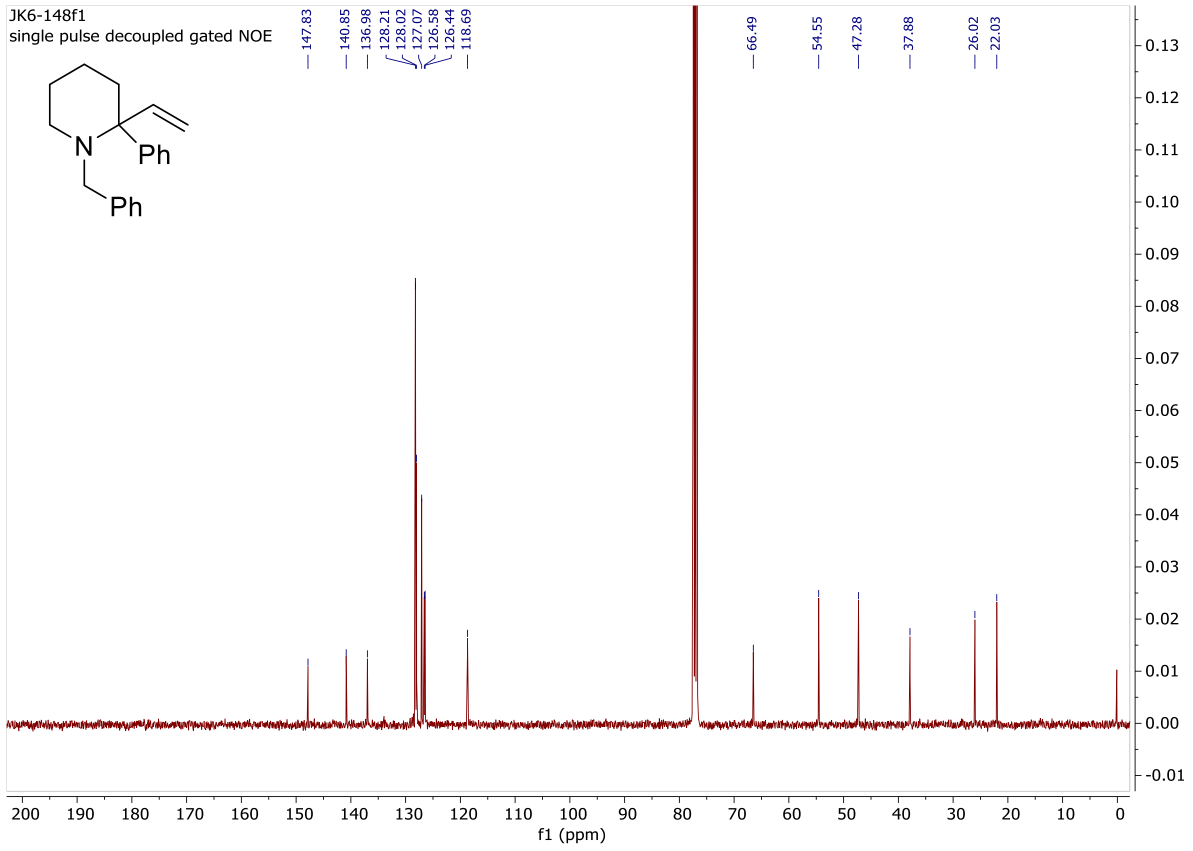

^{1}H NMR (CDCl3 400 MHz) of compound 7r

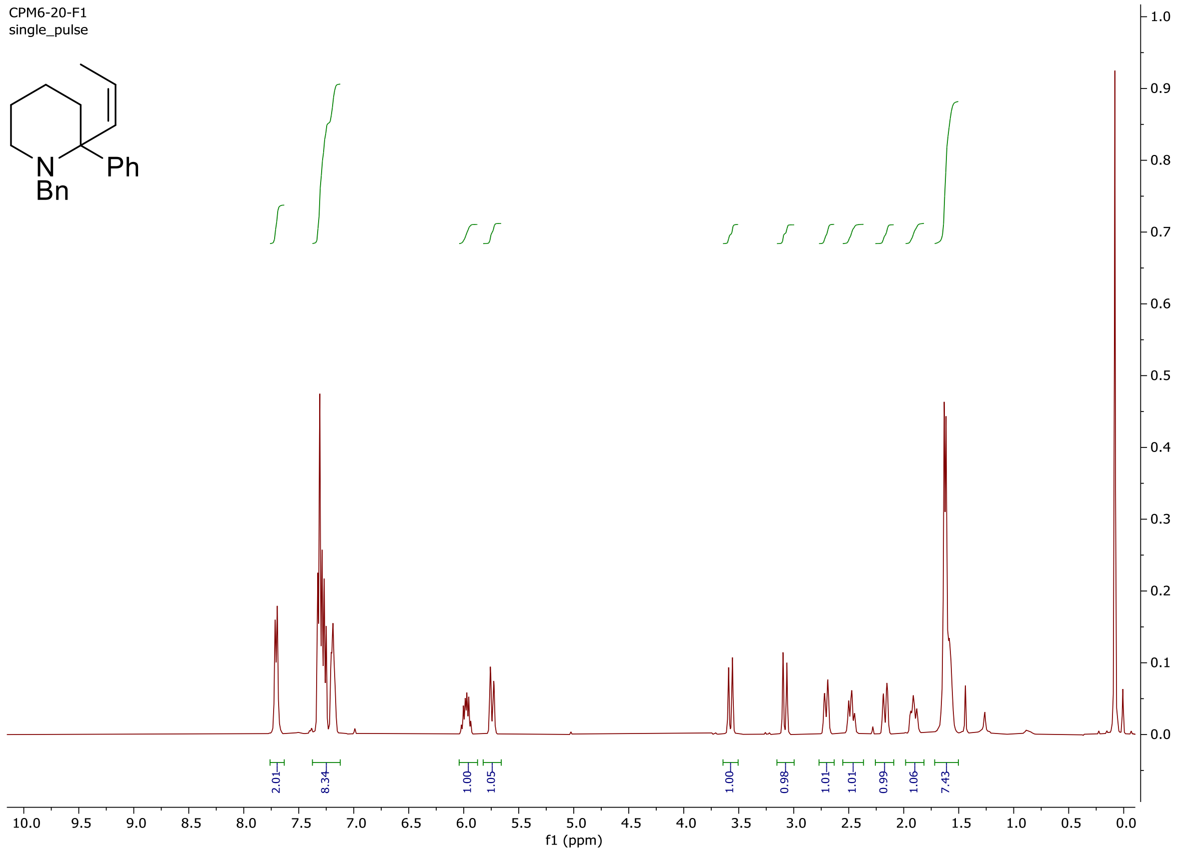

^{13}C NMR (CDCl3 100 MHz) of compound 7r

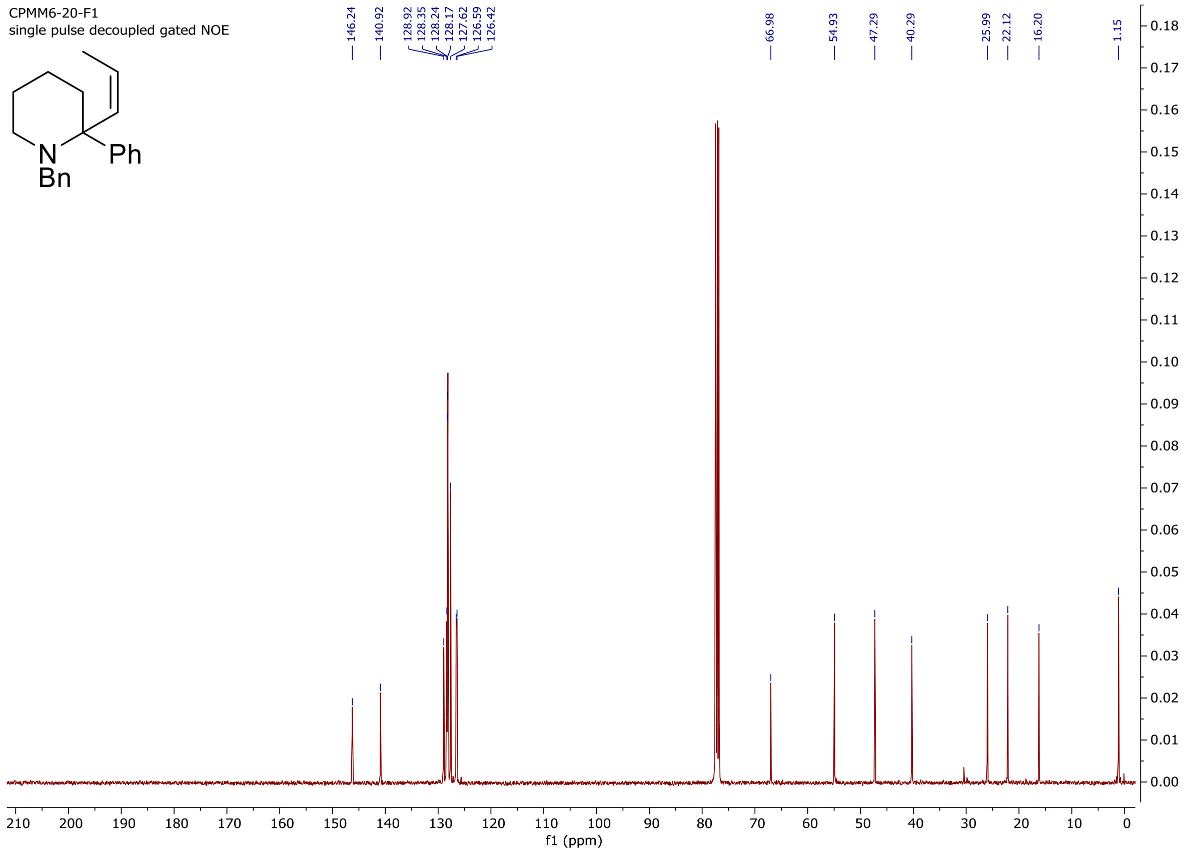

^{1}H NMR ($CDCl_3$ 400 MHz) of compound S27

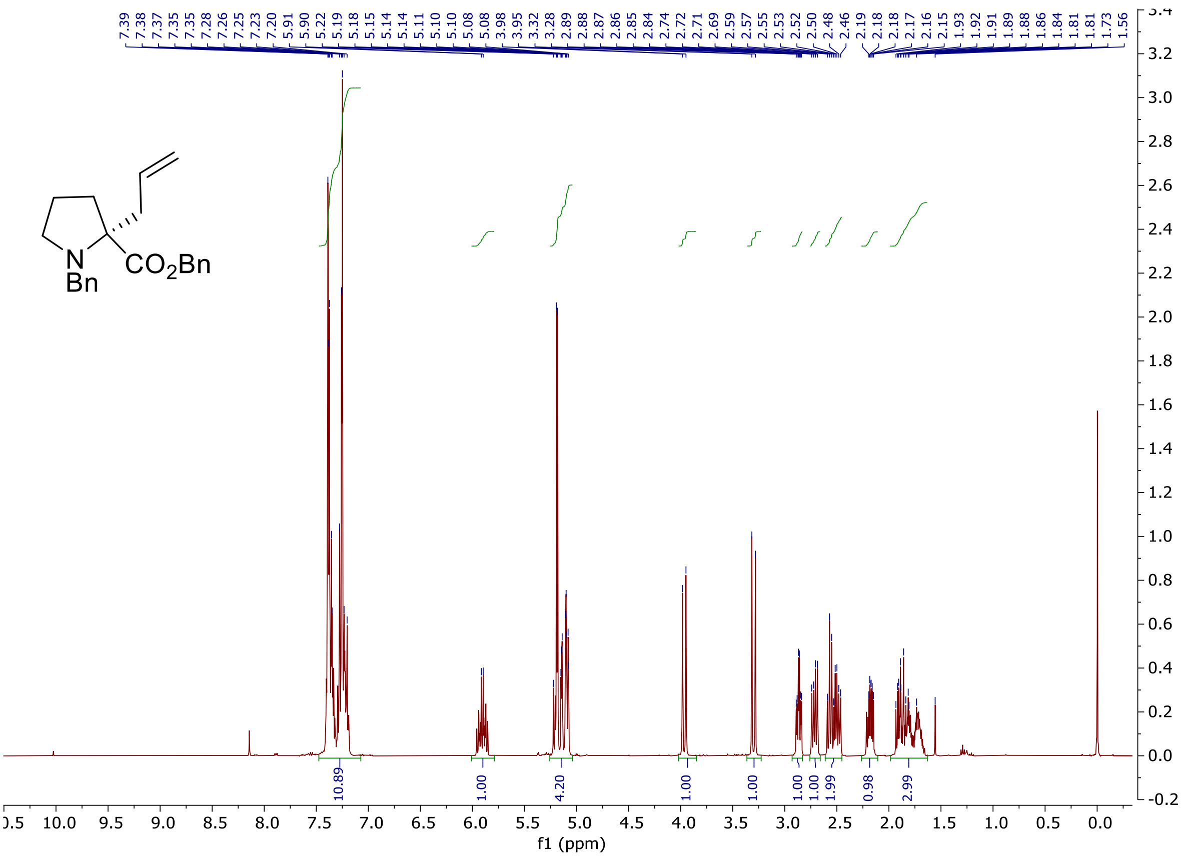

^{13}C NMR ($CDCl_3$ 100 MHz) of compound S27

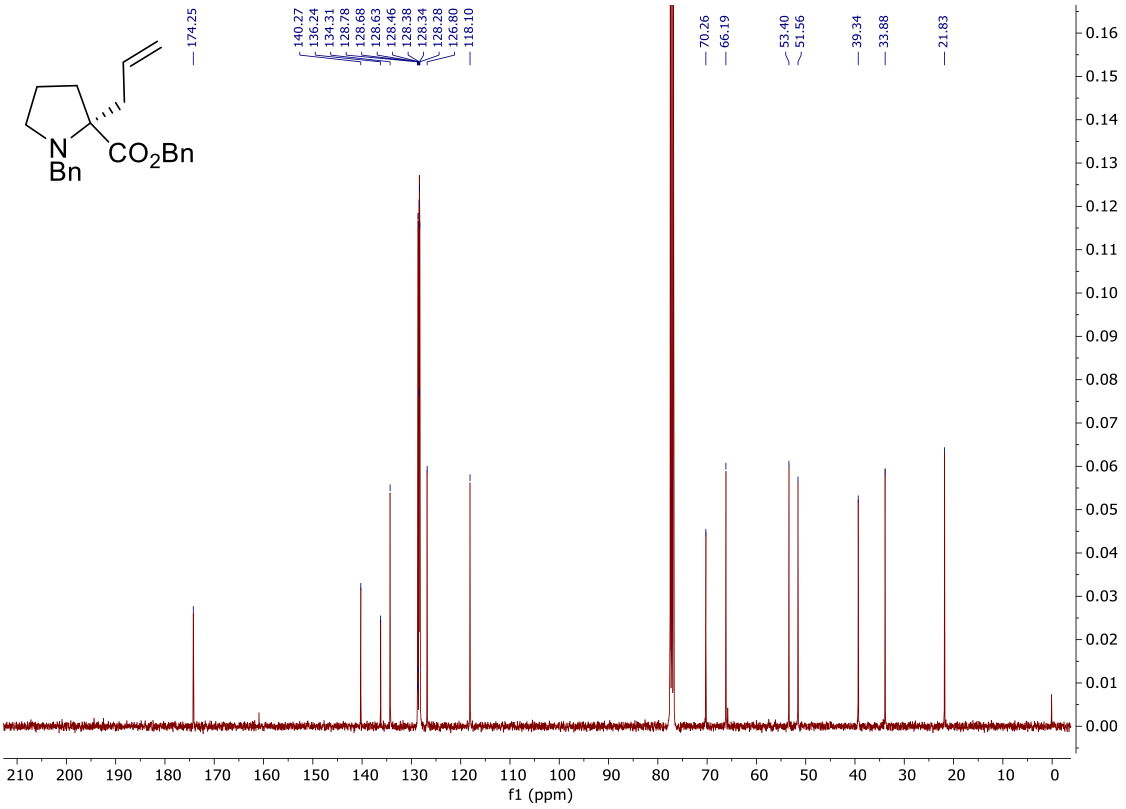

^{1}H NMR ($CDCl_3$ 400 MHz) of compound 7s

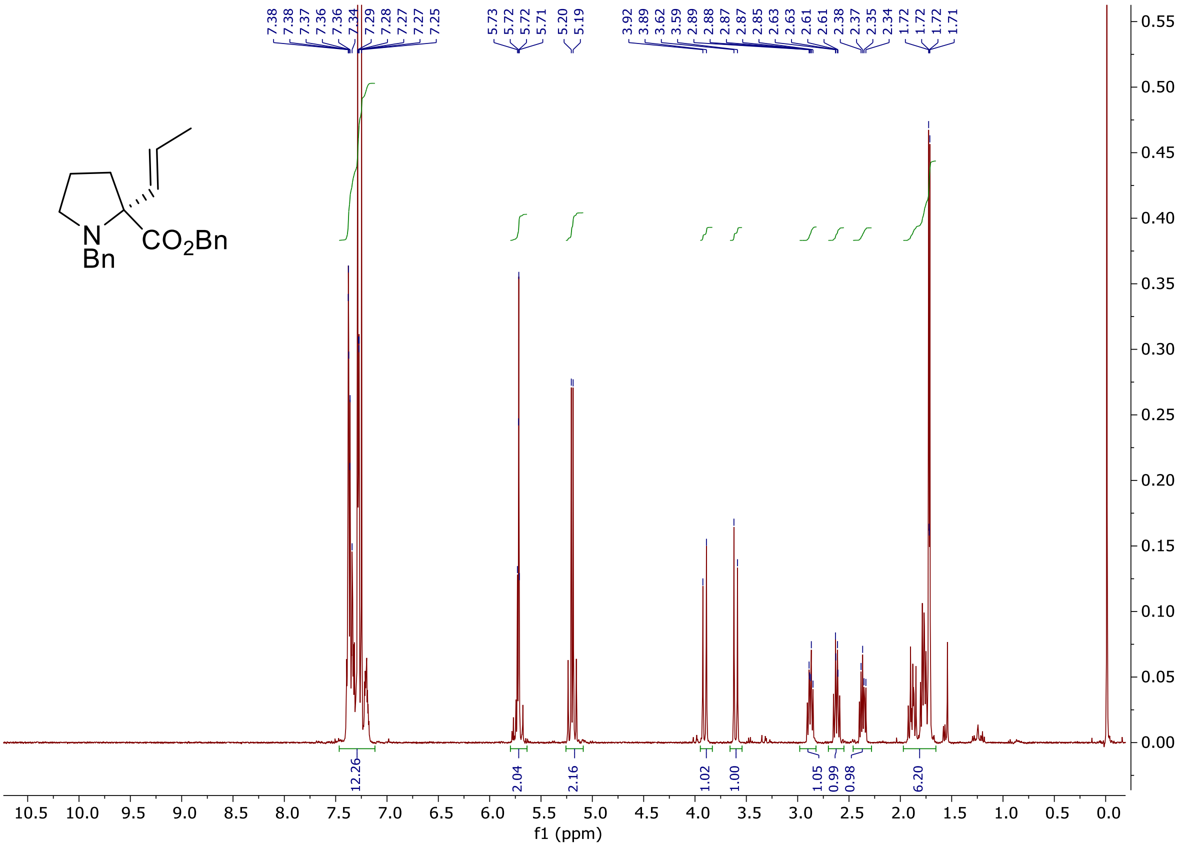

^{13}C NMR ($CDCl_3$ 100 MHz) of compound 7s

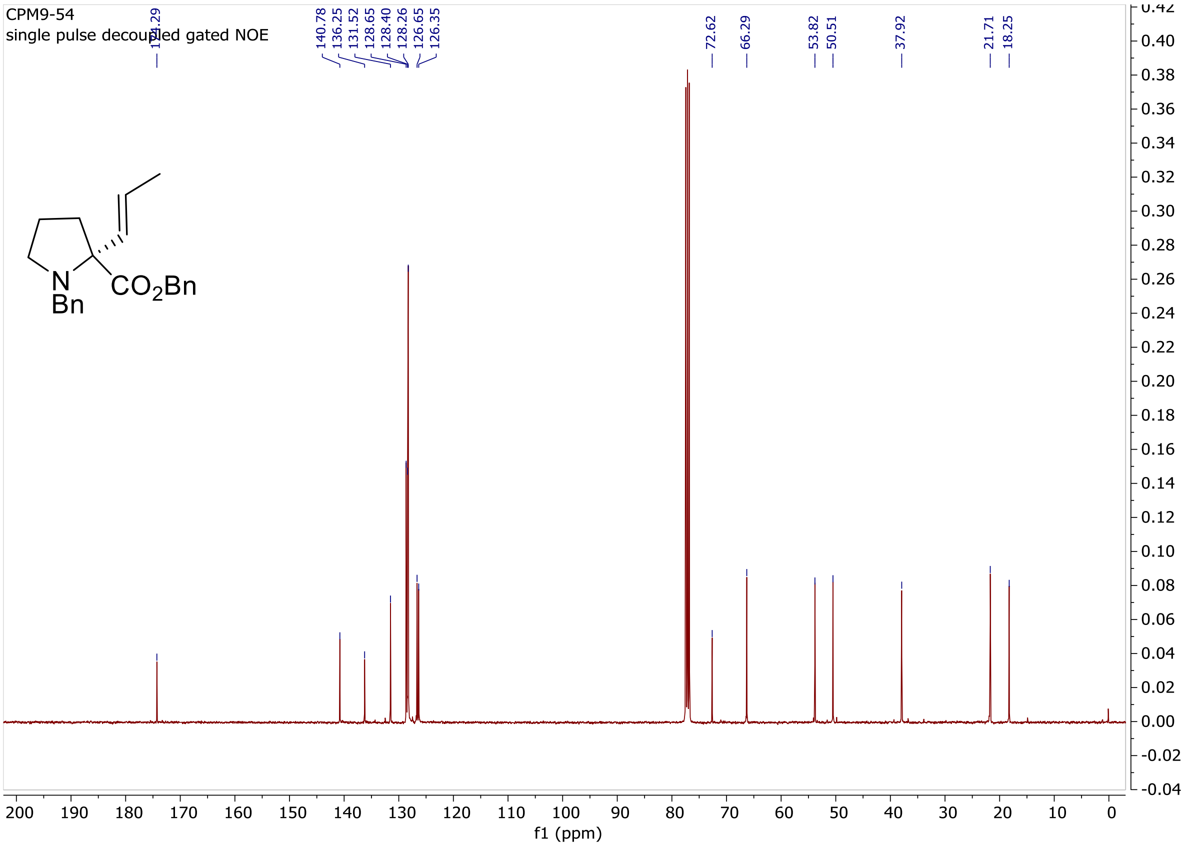

^{1}H NMR ($CDCl_3$ 400 MHz) of compound 8a

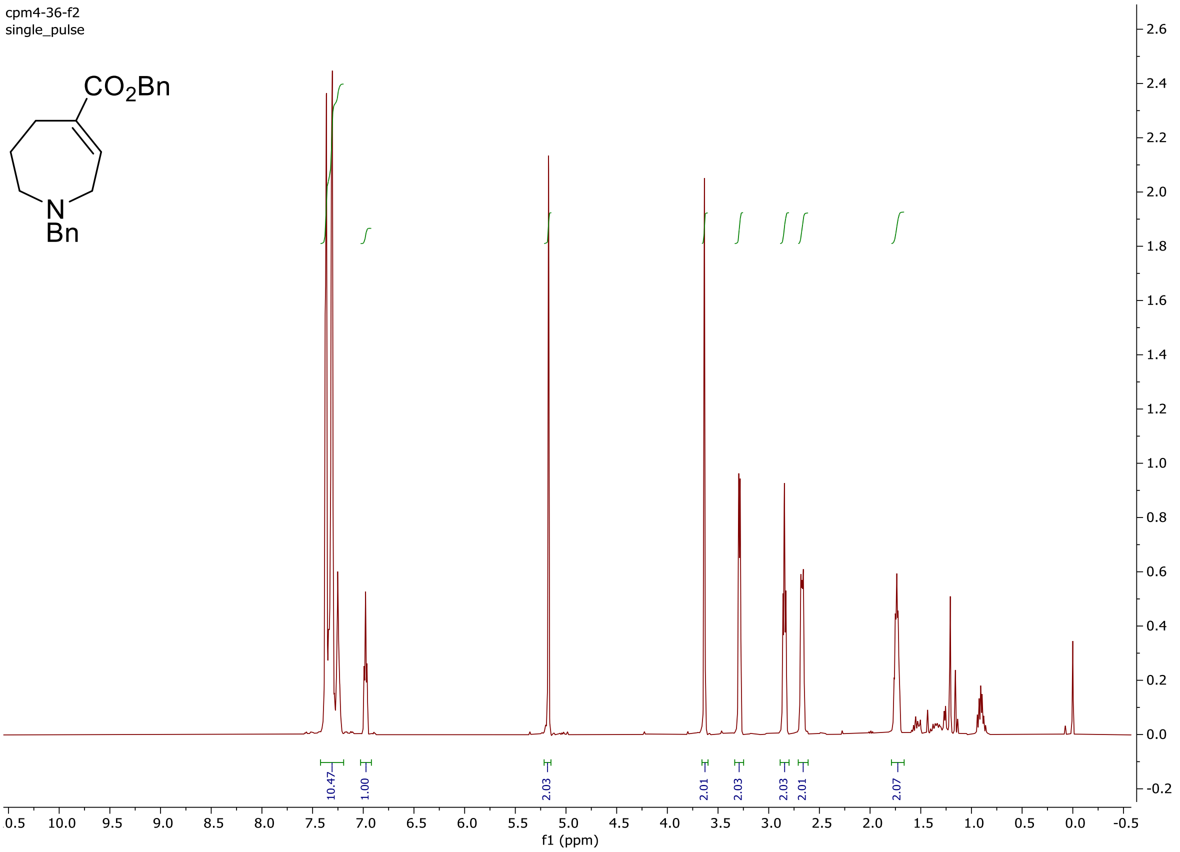

^{13}C NMR ($CDCl_3$ 100 MHz) of compound 8a

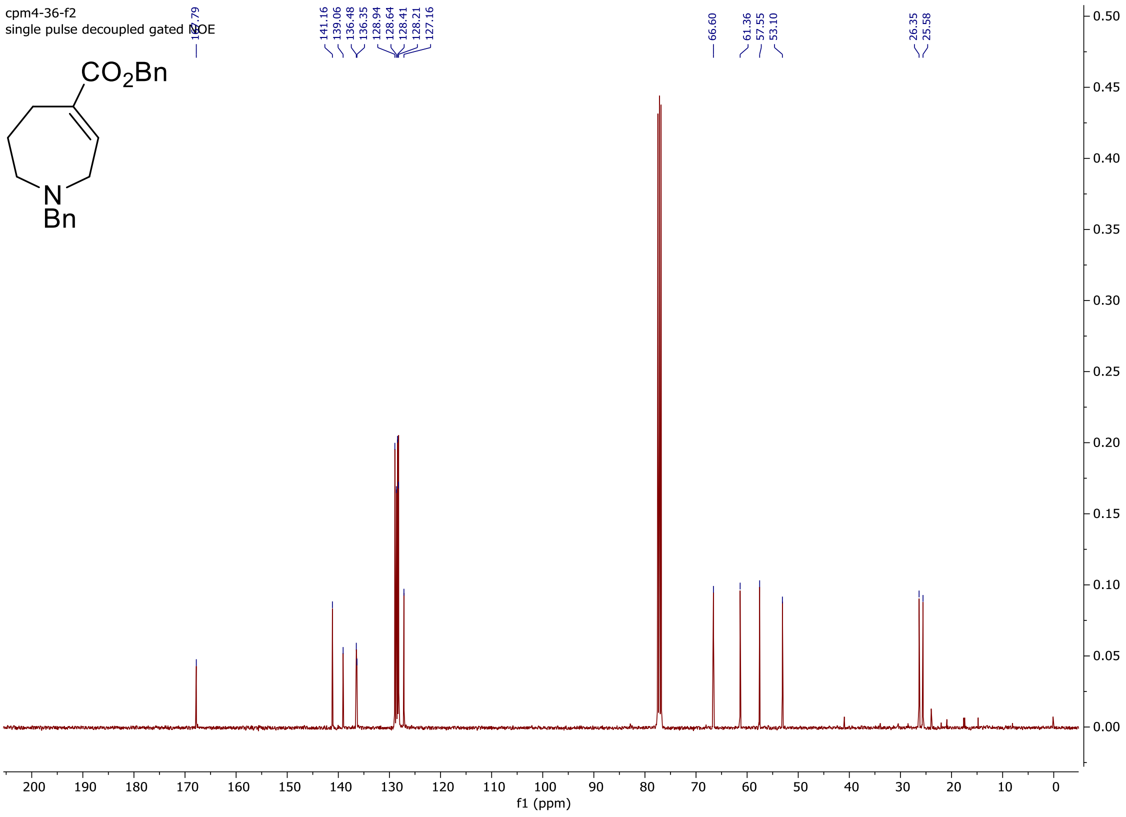

^{1}H NMR ($CDCl_3$ 400 MHz) of compound 8b

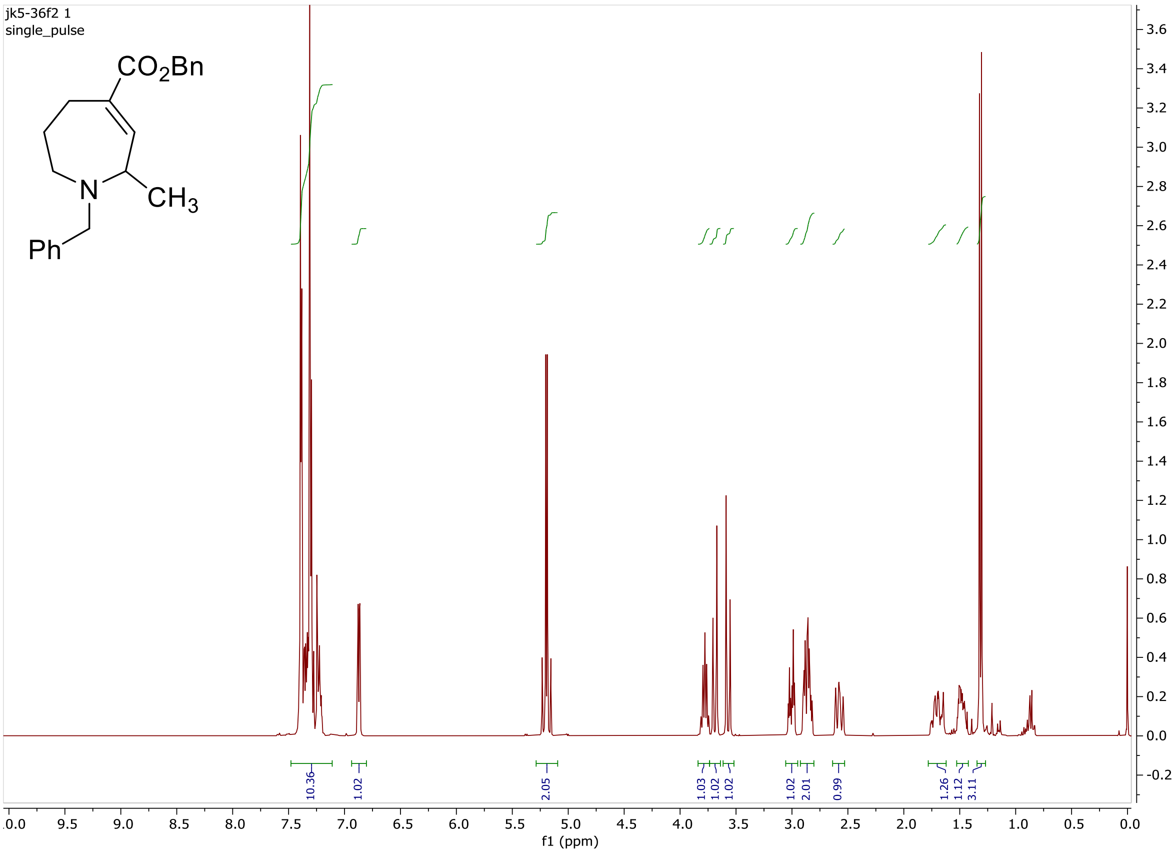

^{13}C NMR ($CDCl_3$ 100 MHz) of compound 8b

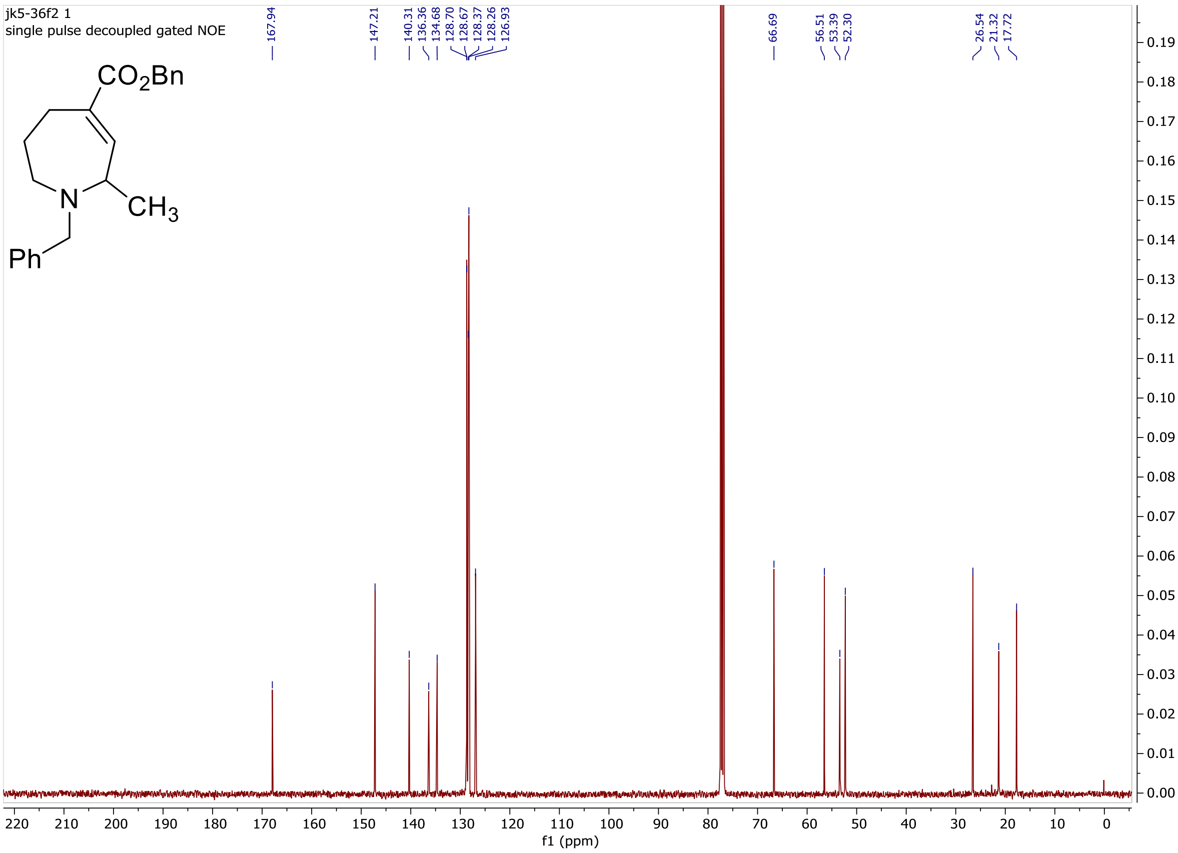

^{1}H NMR ($CDCl_3$ 400 MHz) of compound 8c

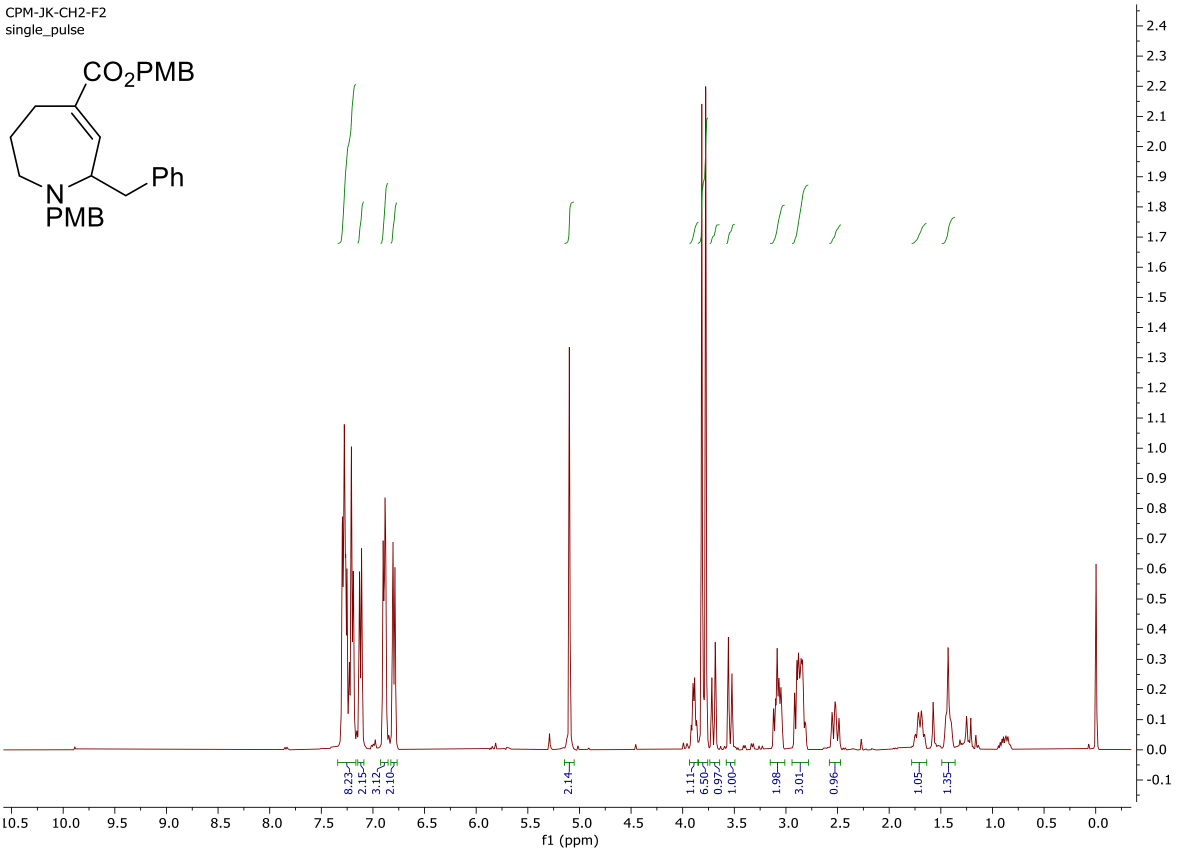

^{13}C NMR ($CDCl_3$ 100 MHz) of compound 8c

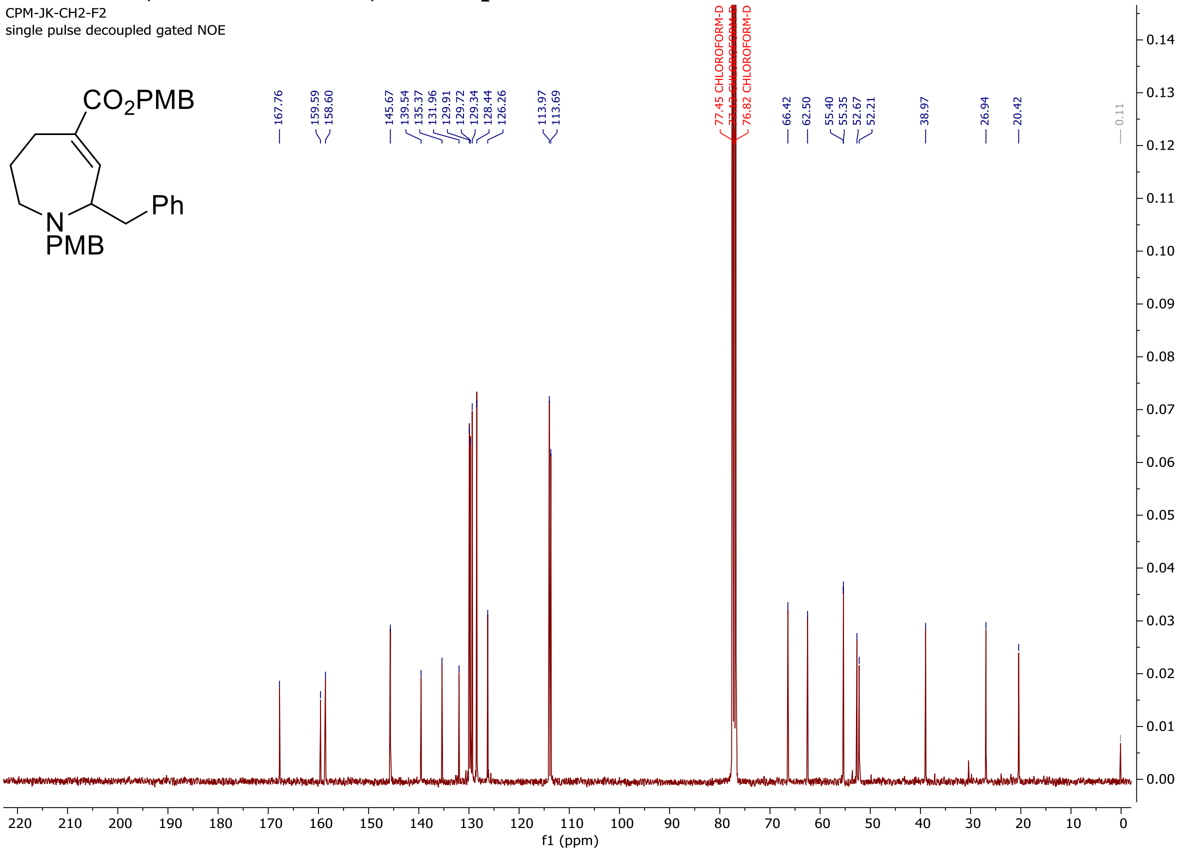

^{1}H NMR ($CDCl_3$ 400 MHz) of compound 8e

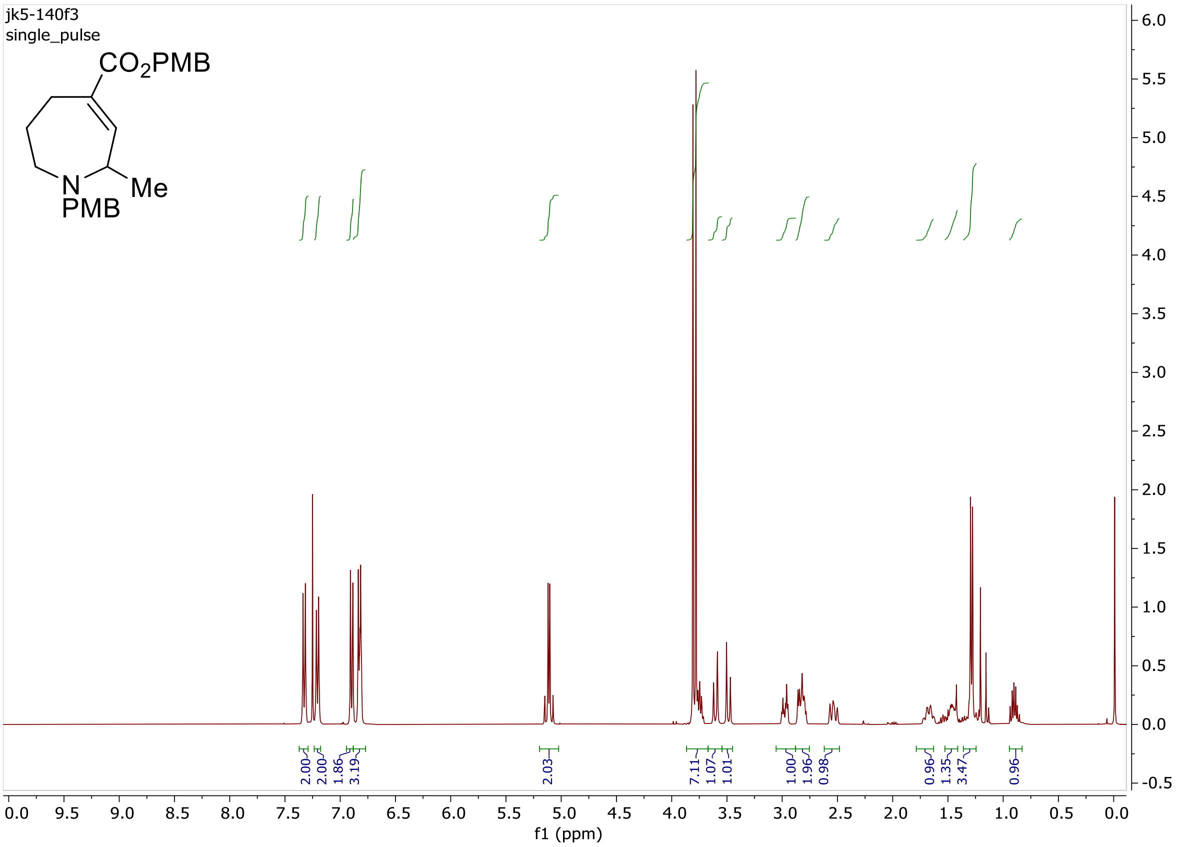

^{13}C NMR ($CDCl_3$ 100 MHz) of compound 8e

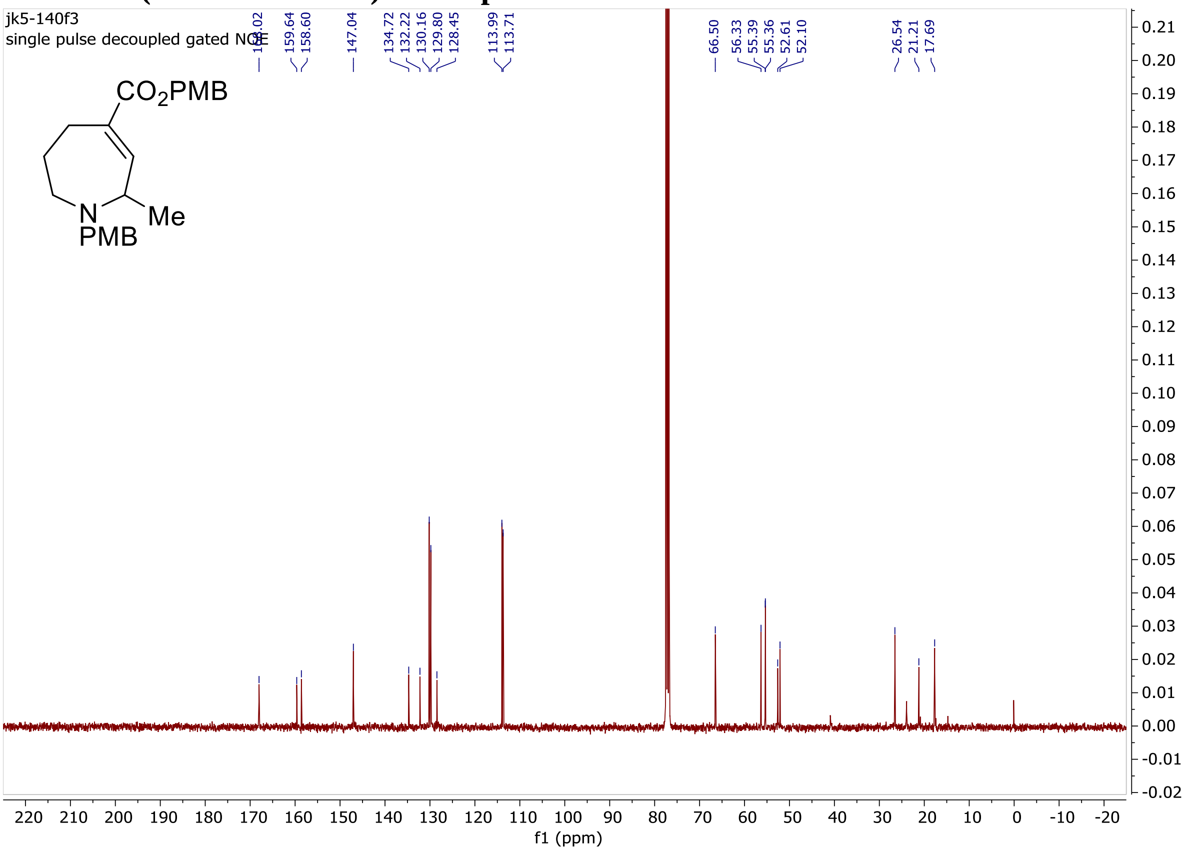

^{1}H NMR (CDCl3 400 MHz) of compound 8f

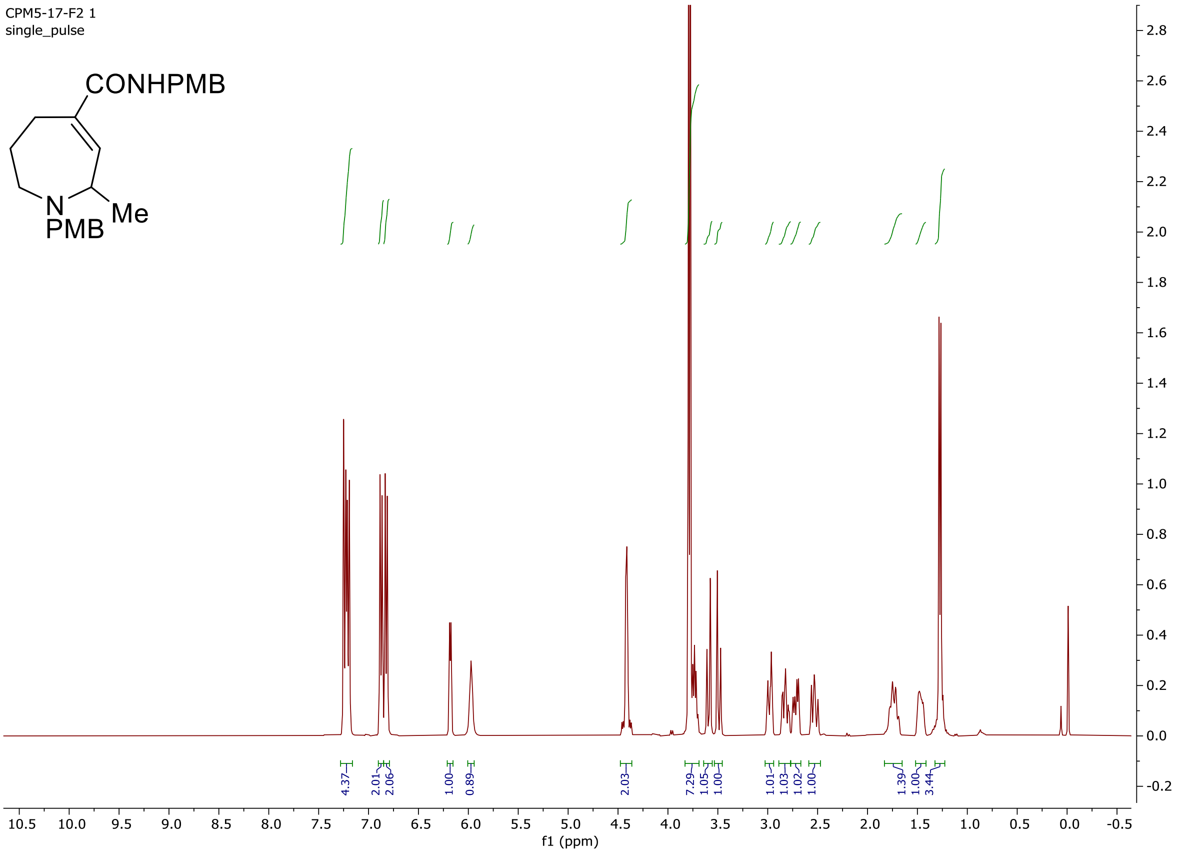

^{13}C NMR (CDCl3 100 MHz) of compound 8f

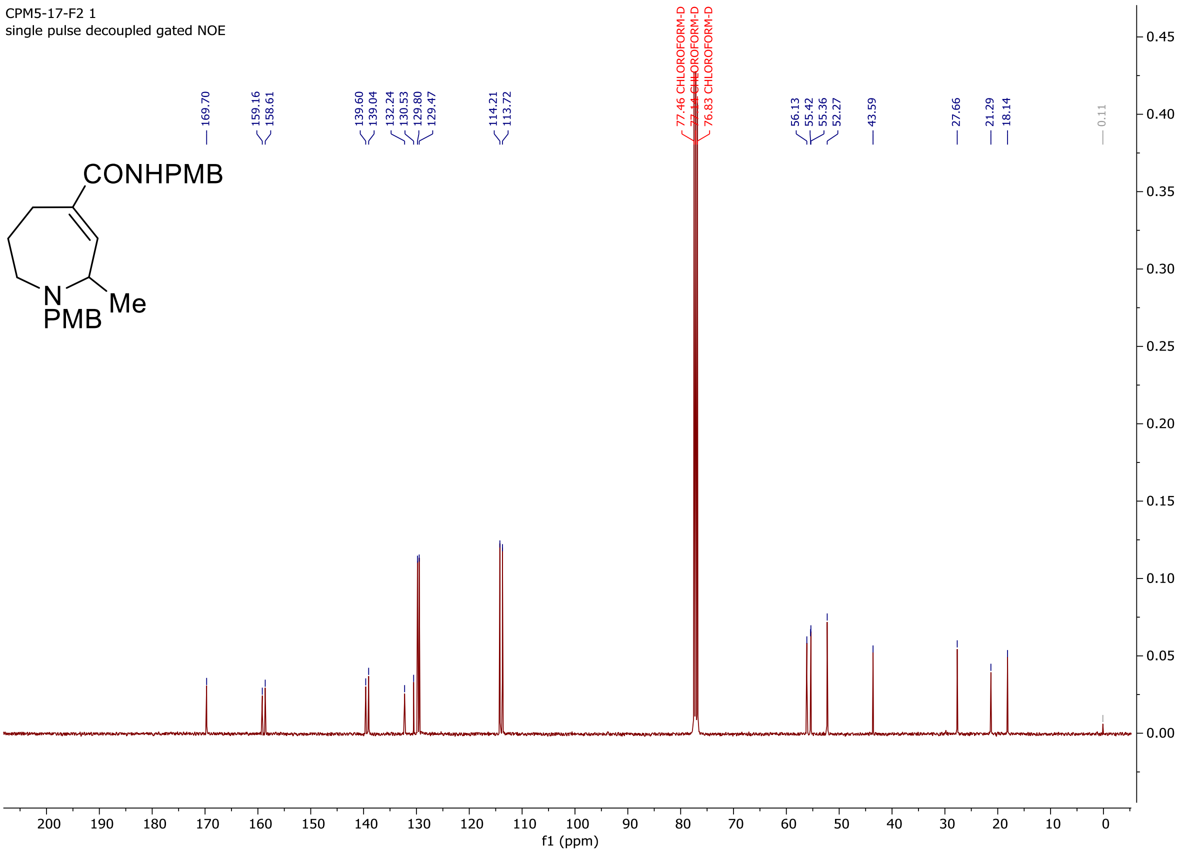

^{1}H NMR ($CDCl_3$ 400 MHz) of compound 8g

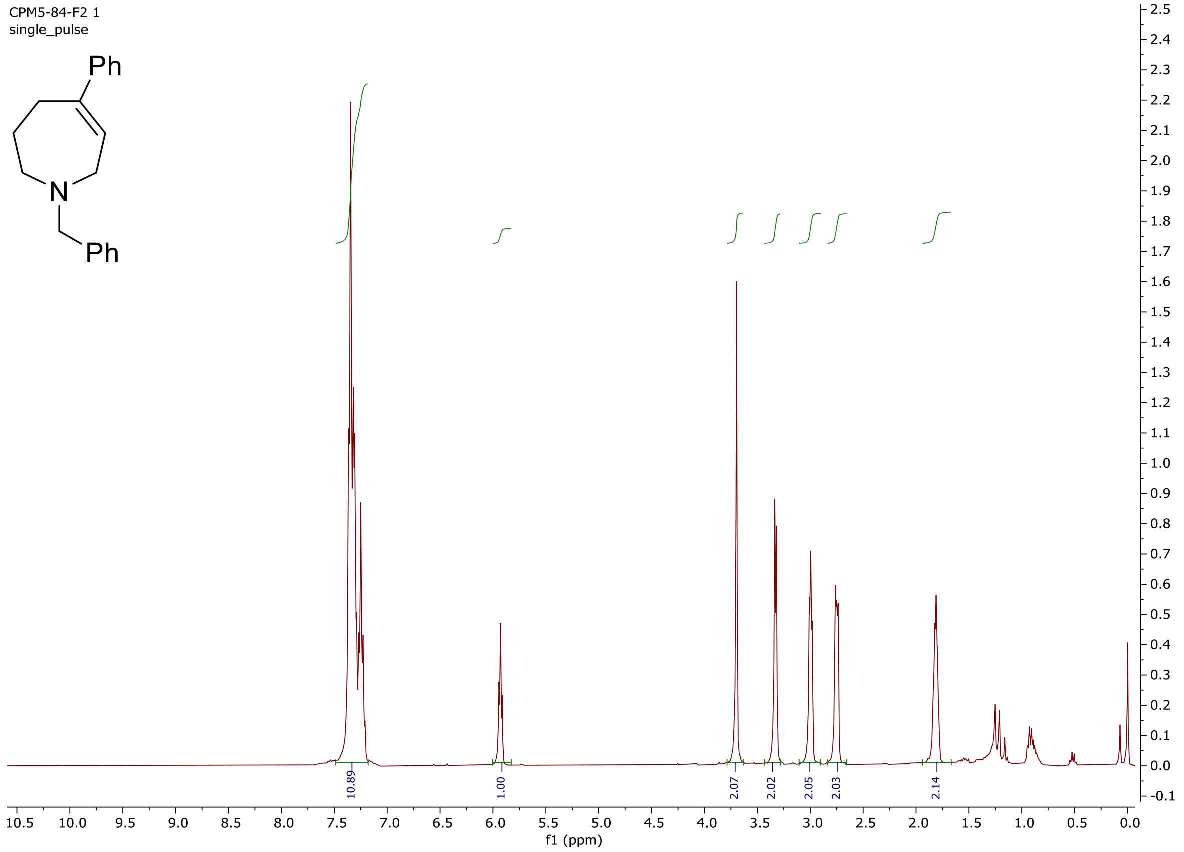

^{13}C NMR ($CDCl_3$ 100 MHz) of compound 8g

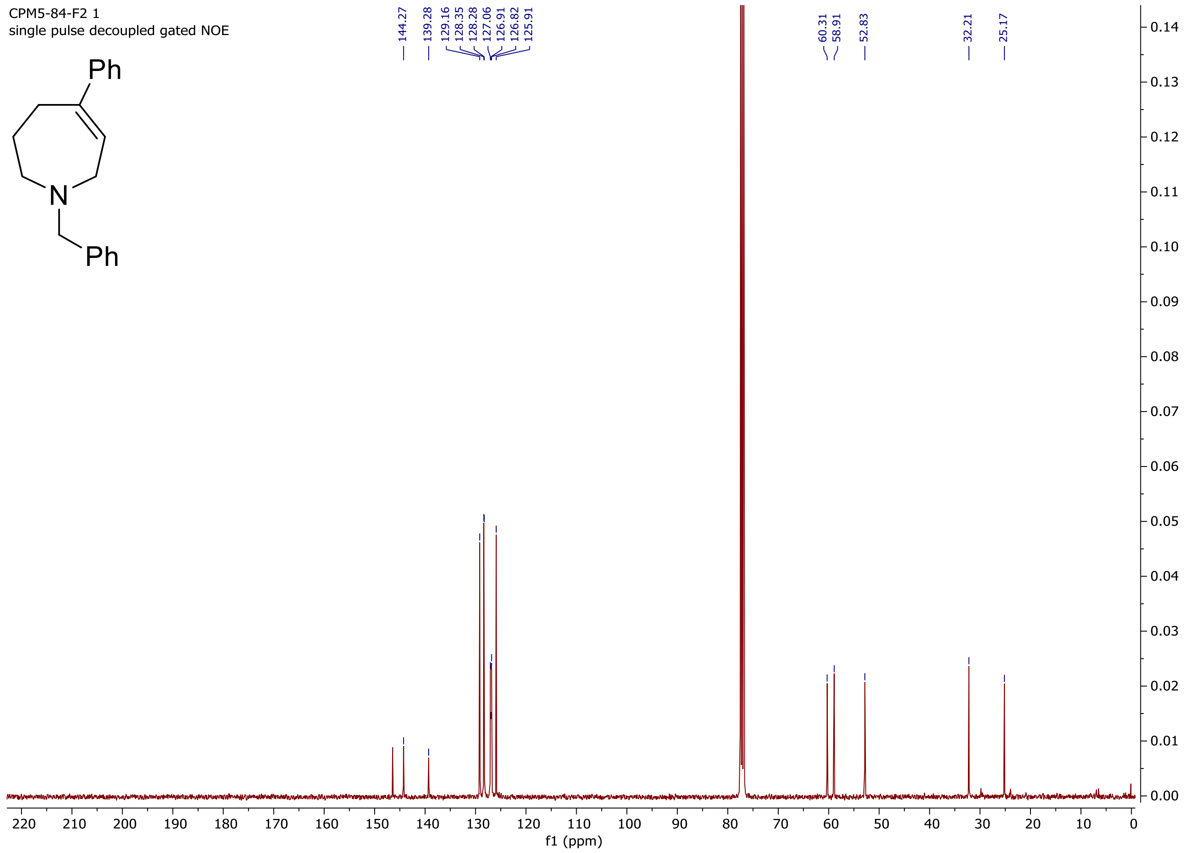

^{1}H NMR (CDCl3 400 MHz) of compound 8h

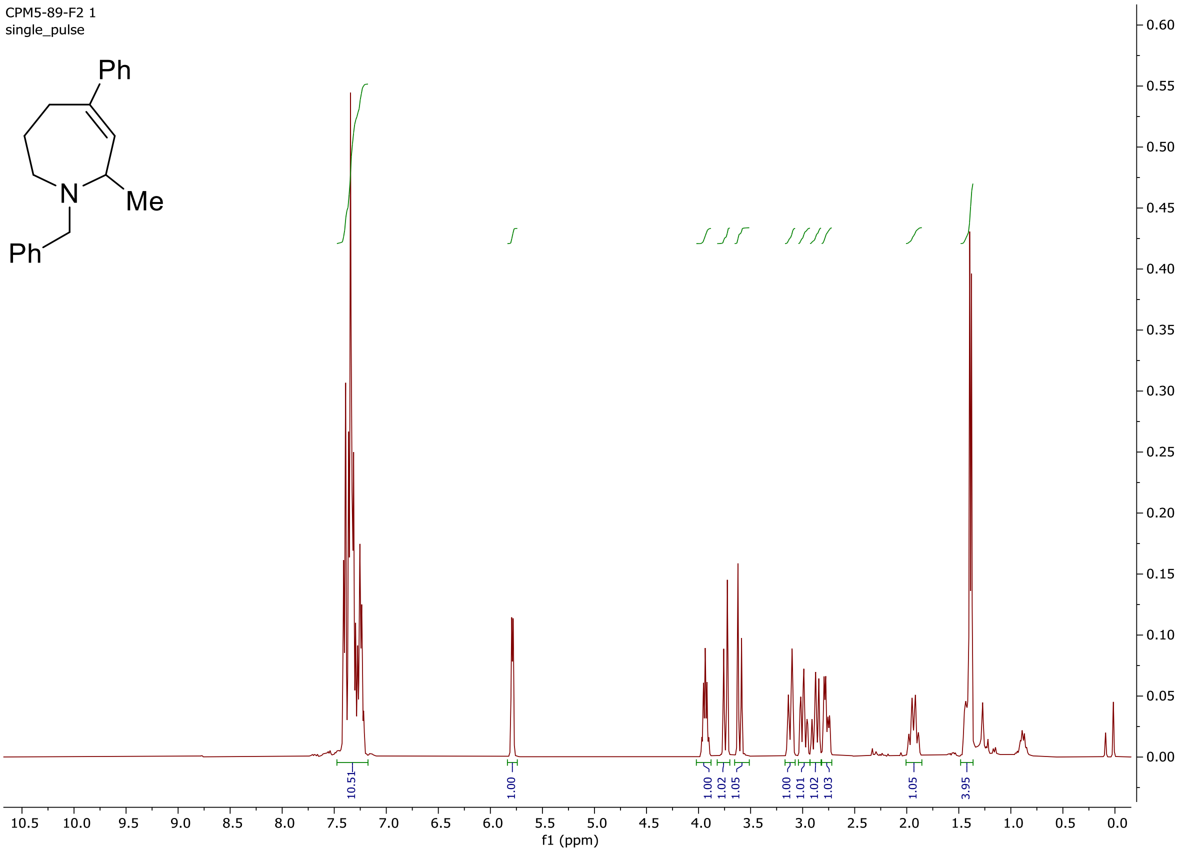

^{13}C NMR (CDCl3 100 MHz) of compound 8h

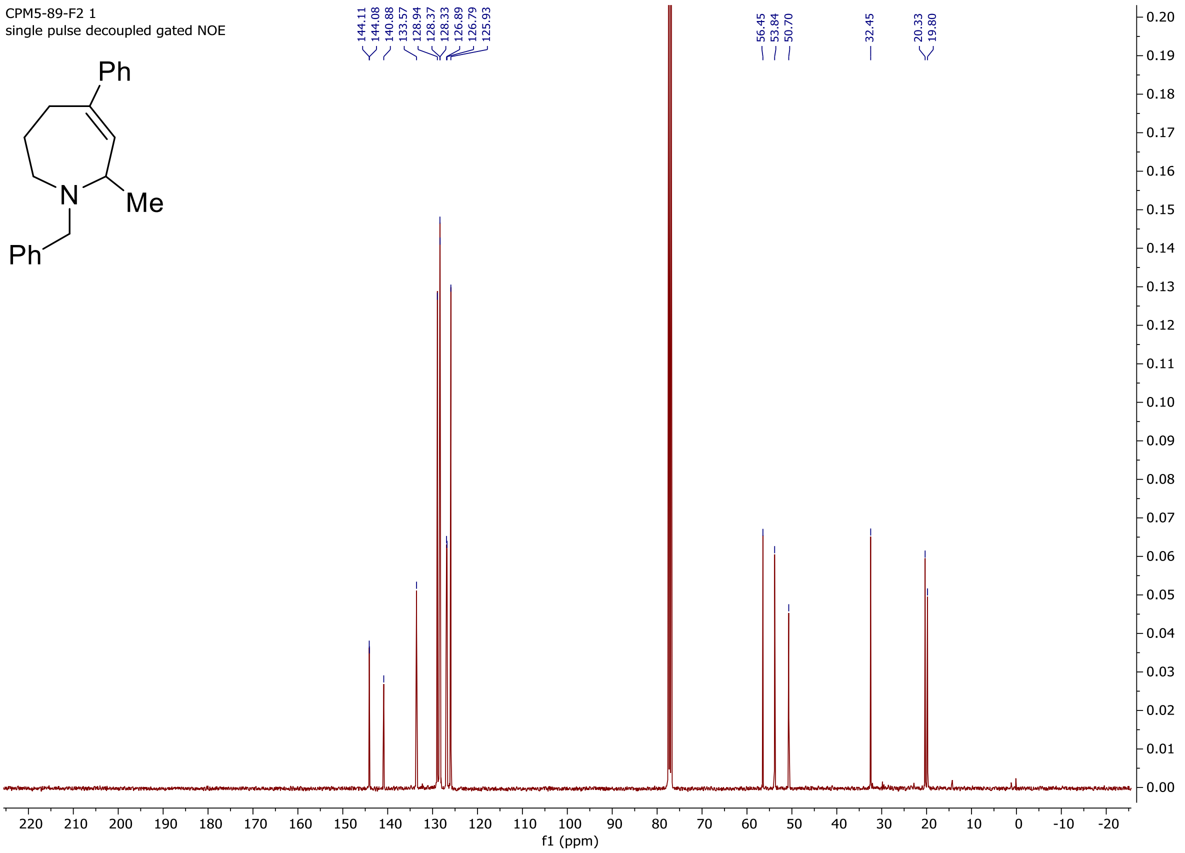

^{1}H NMR (CDCl$_3$ 400 MHz) of compound 8i

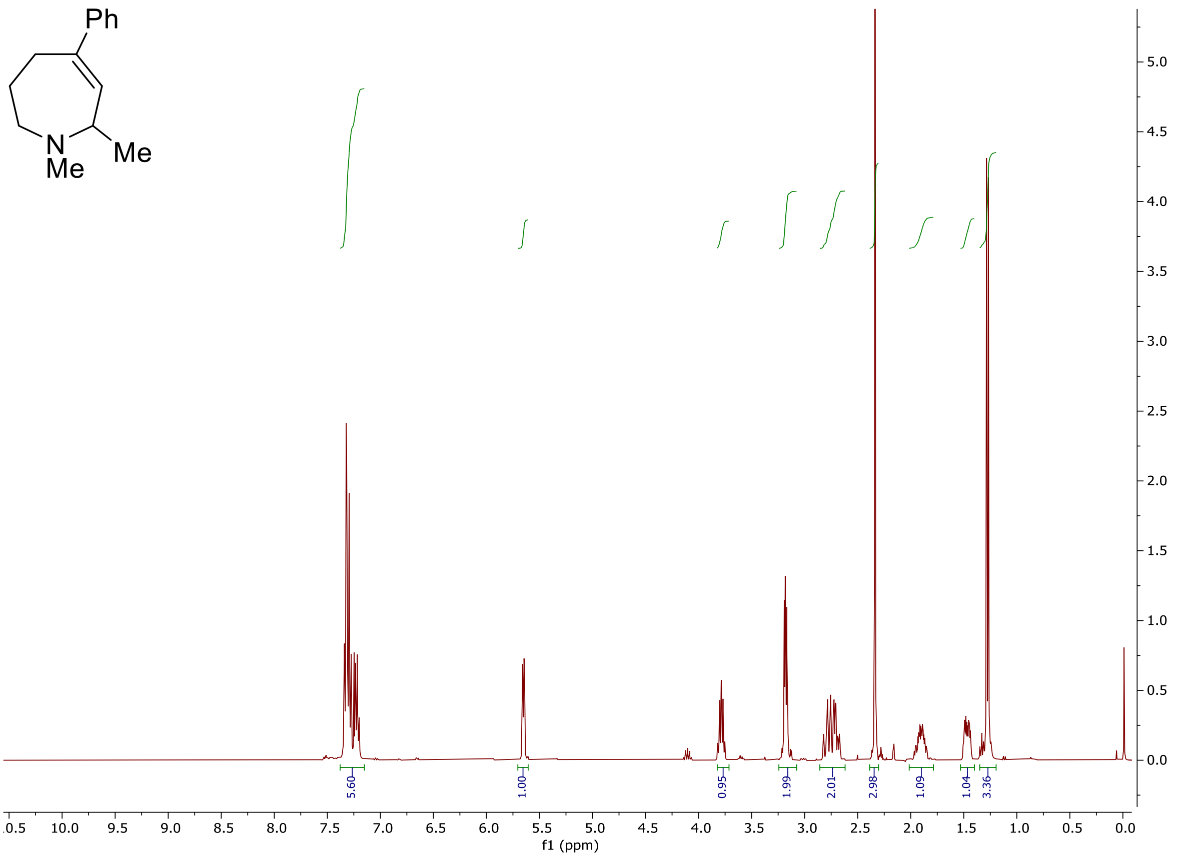

^{13}C NMR (CDCl$_3$ 100 MHz) of compound 8i

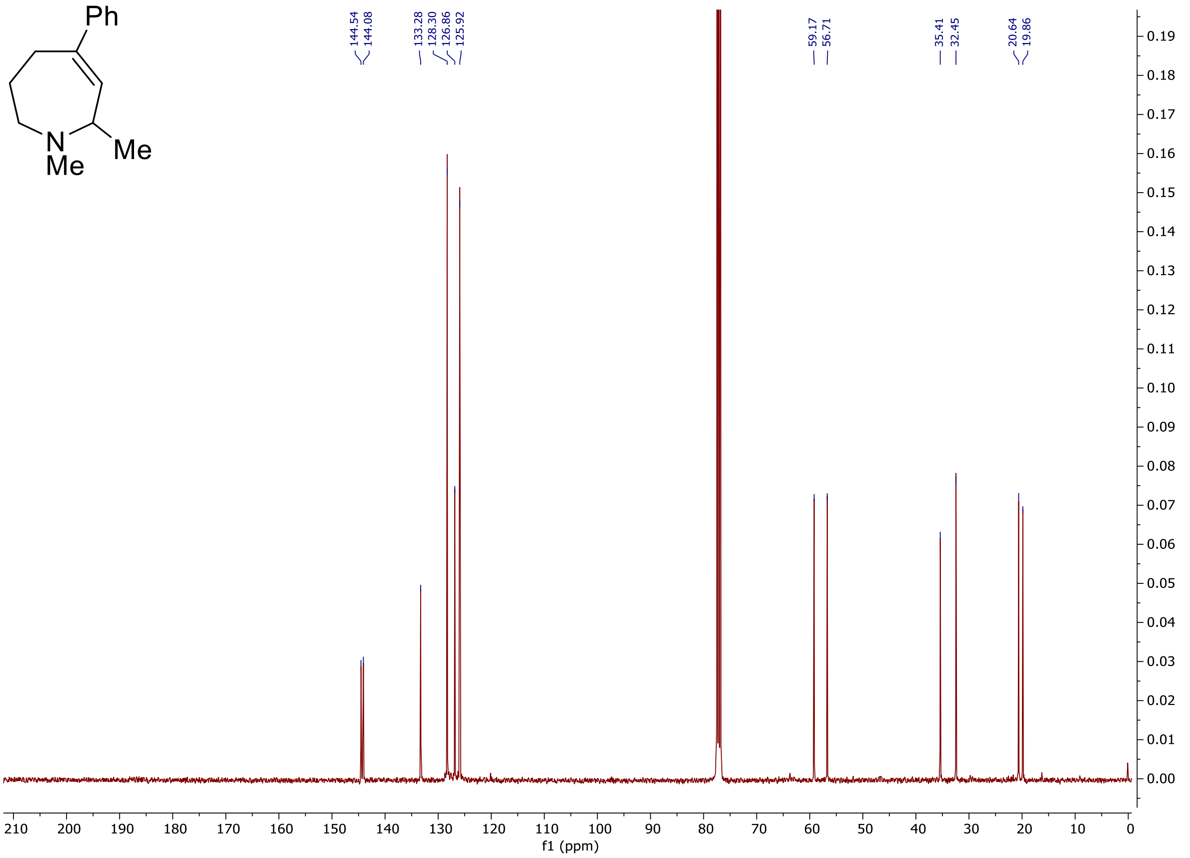

^{1}H NMR ($CDCl_3$ 400 MHz) of compound 8j

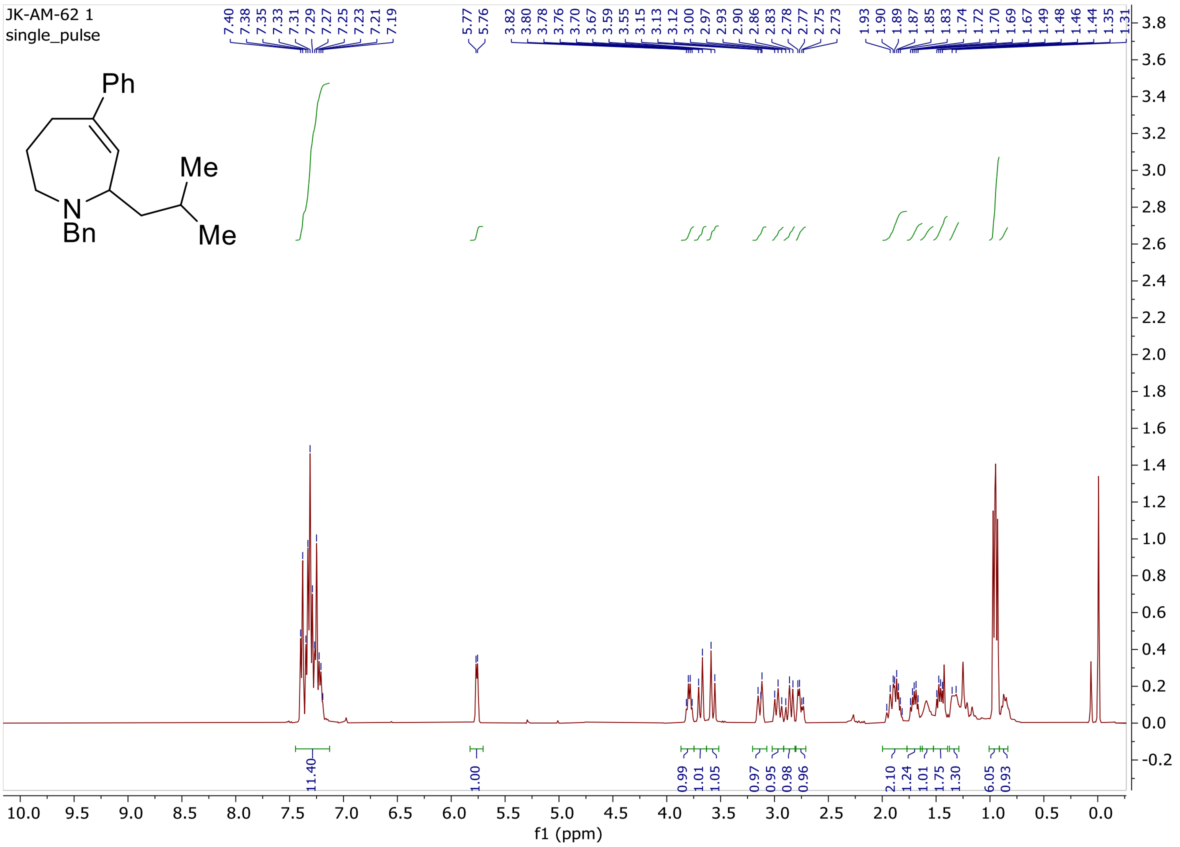

^{13}C NMR ($CDCl_3$ 100 MHz) of compound 8j

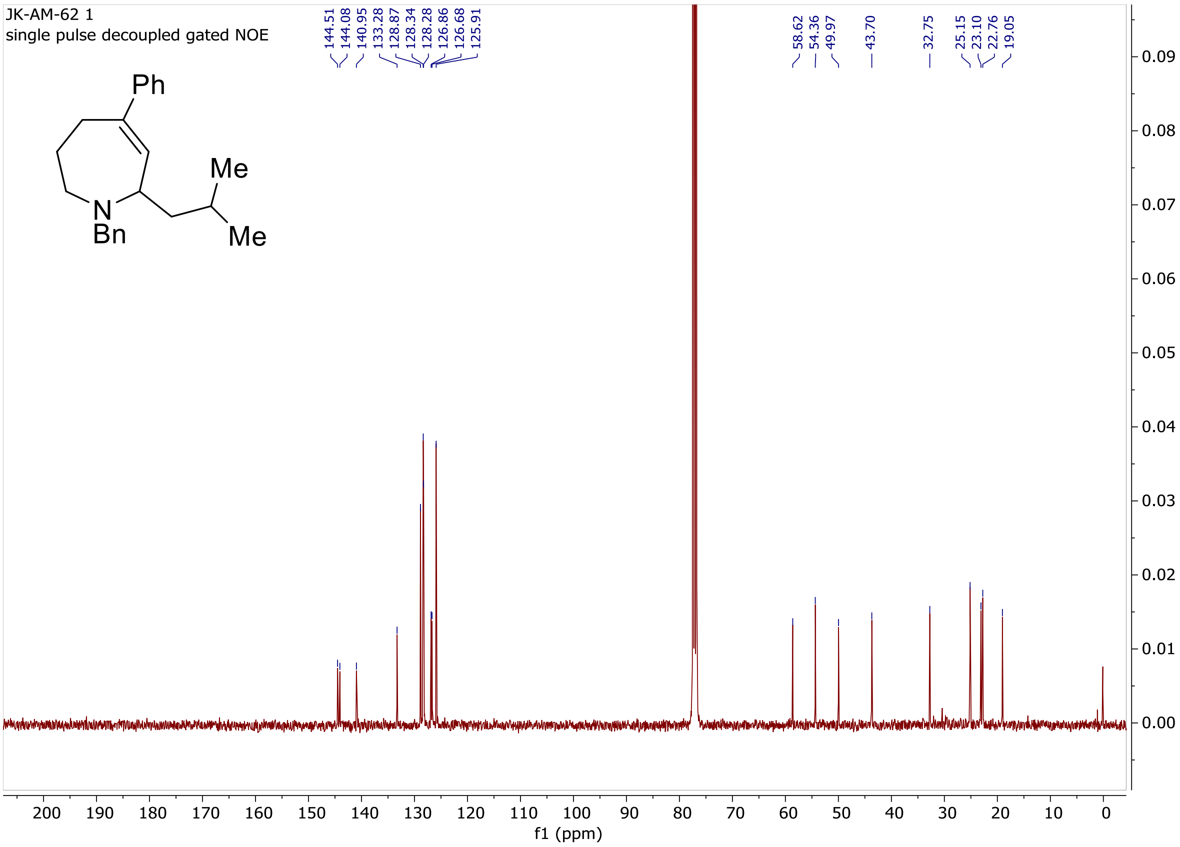

^{1}H NMR ($CDCl_3$ 400 MHz) of compound 8k

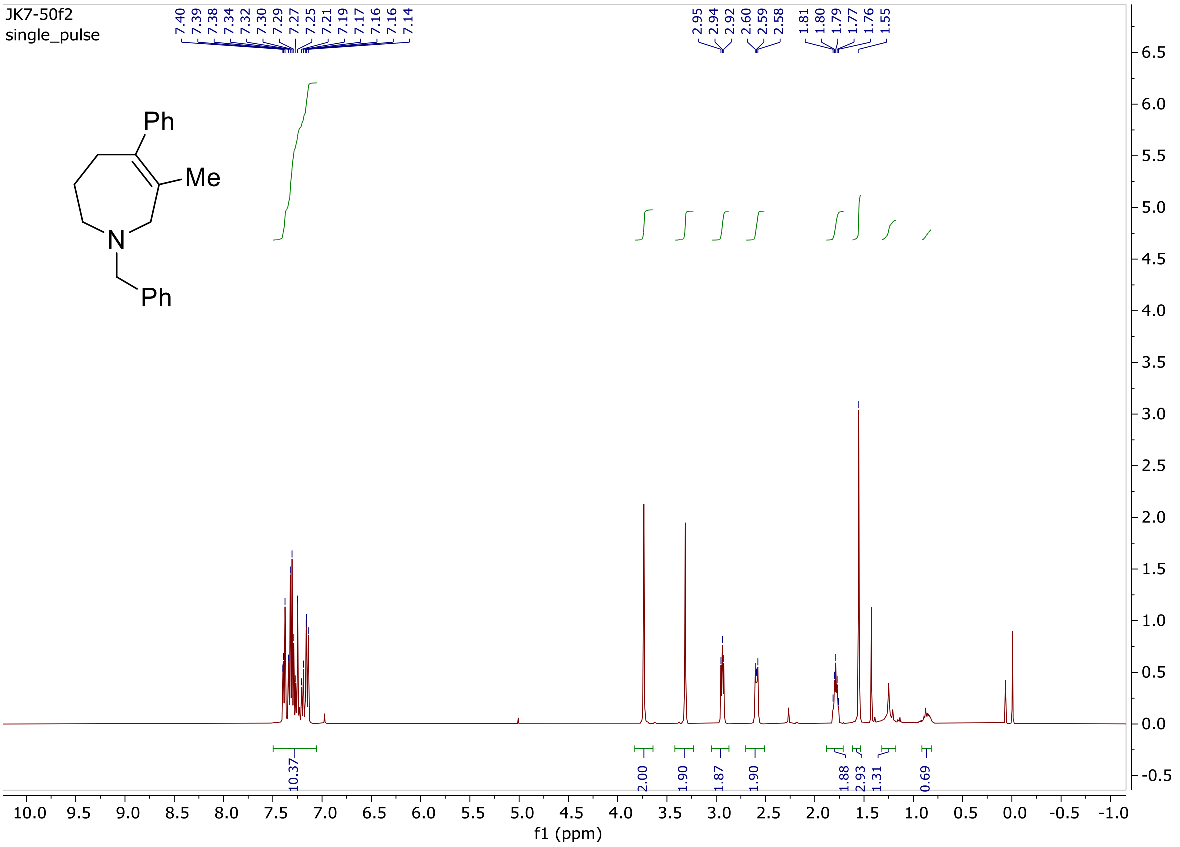

^{13}C NMR ($CDCl_3$ 100 MHz) of compound 8k

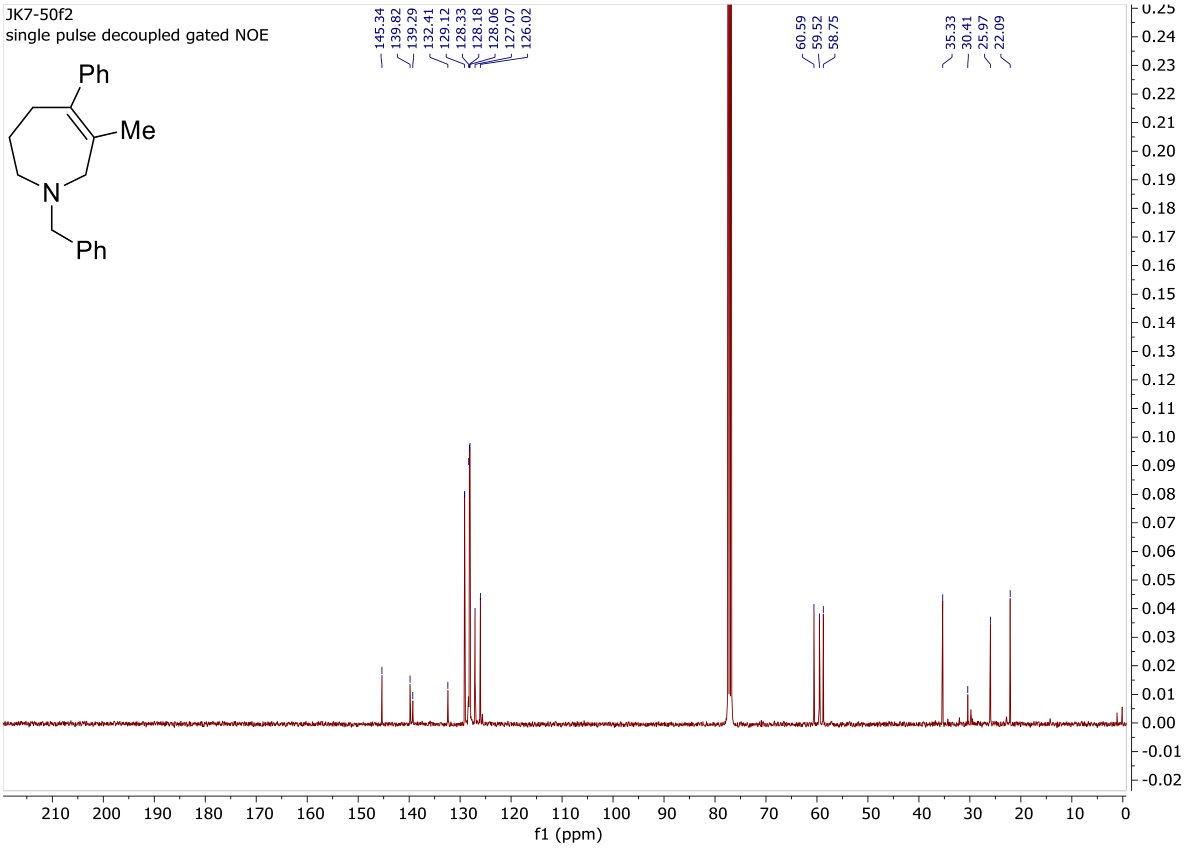

^{1}H NMR ($CDCl_3$ 400 MHz) of compound 8l

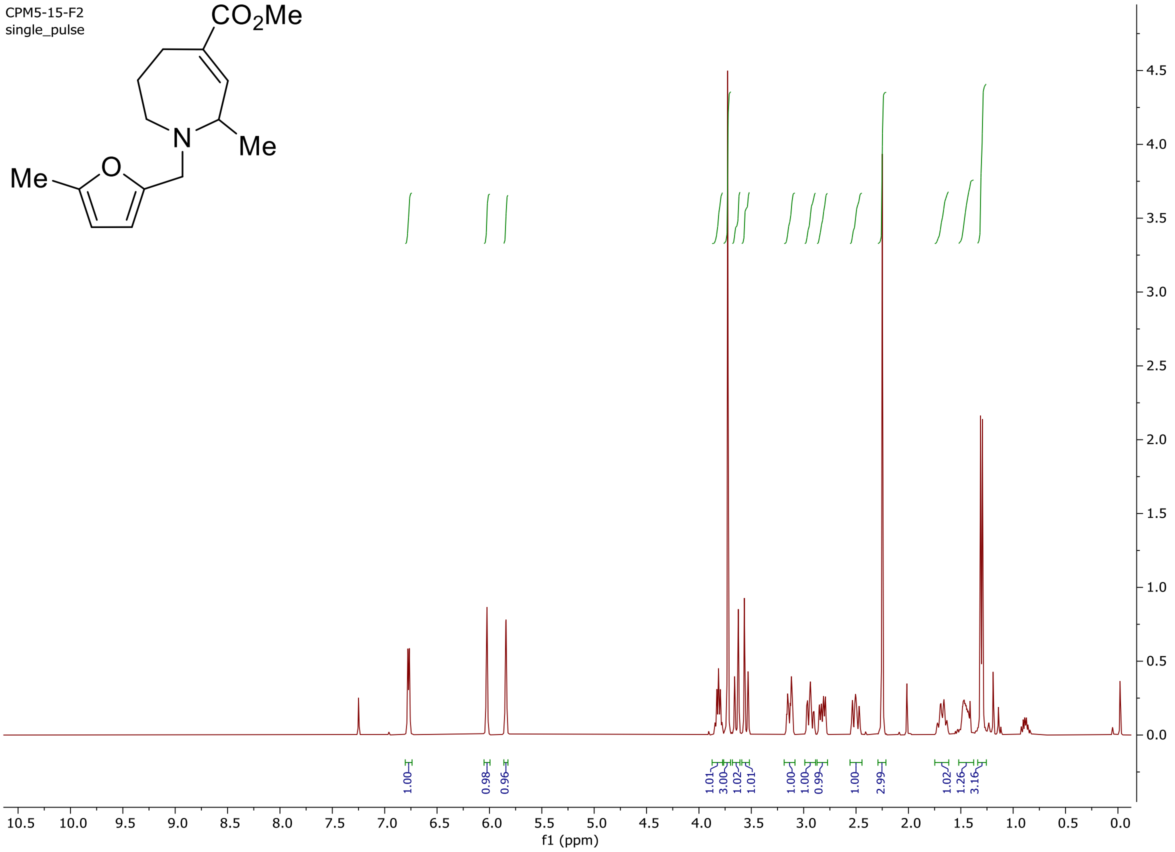

^{13}C NMR ($CDCl_3$ 100 MHz) of compound 8l

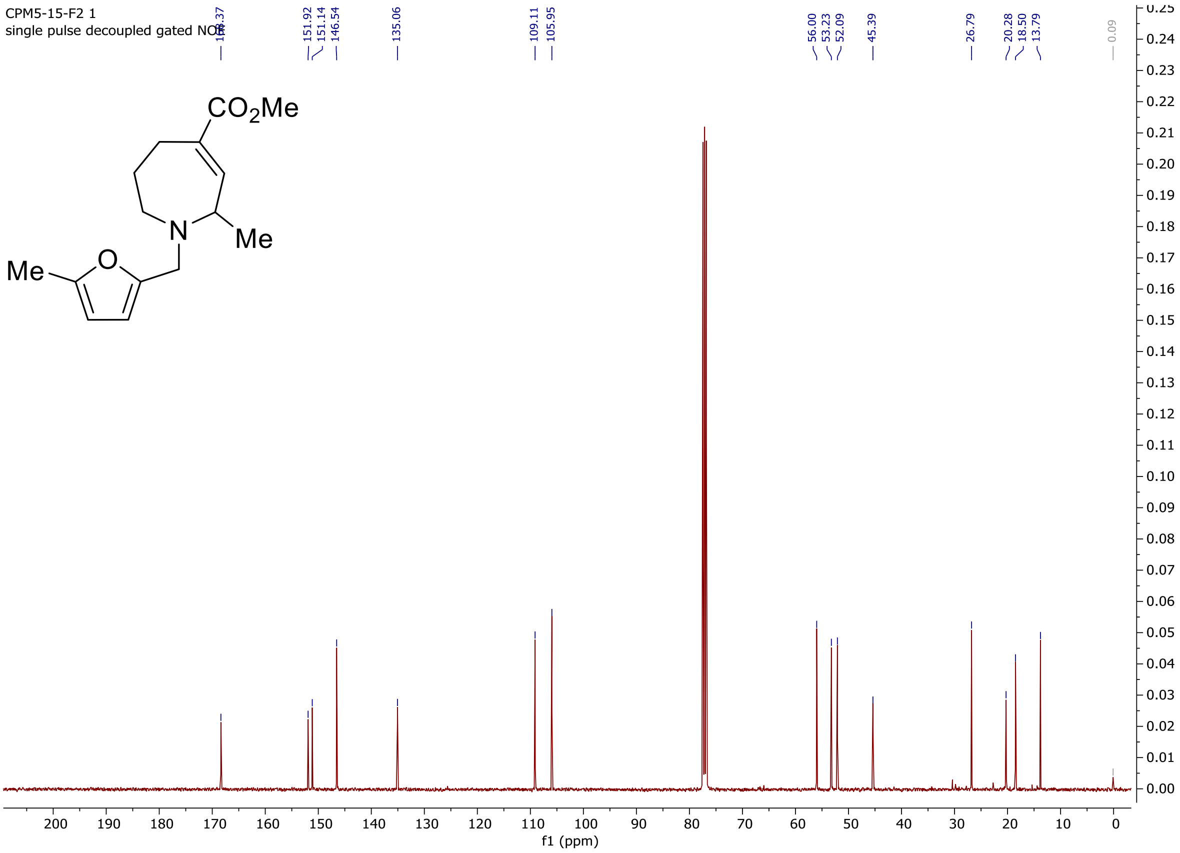

^{1}H NMR (CDCl$_3$ 400 MHz) of compound 8m

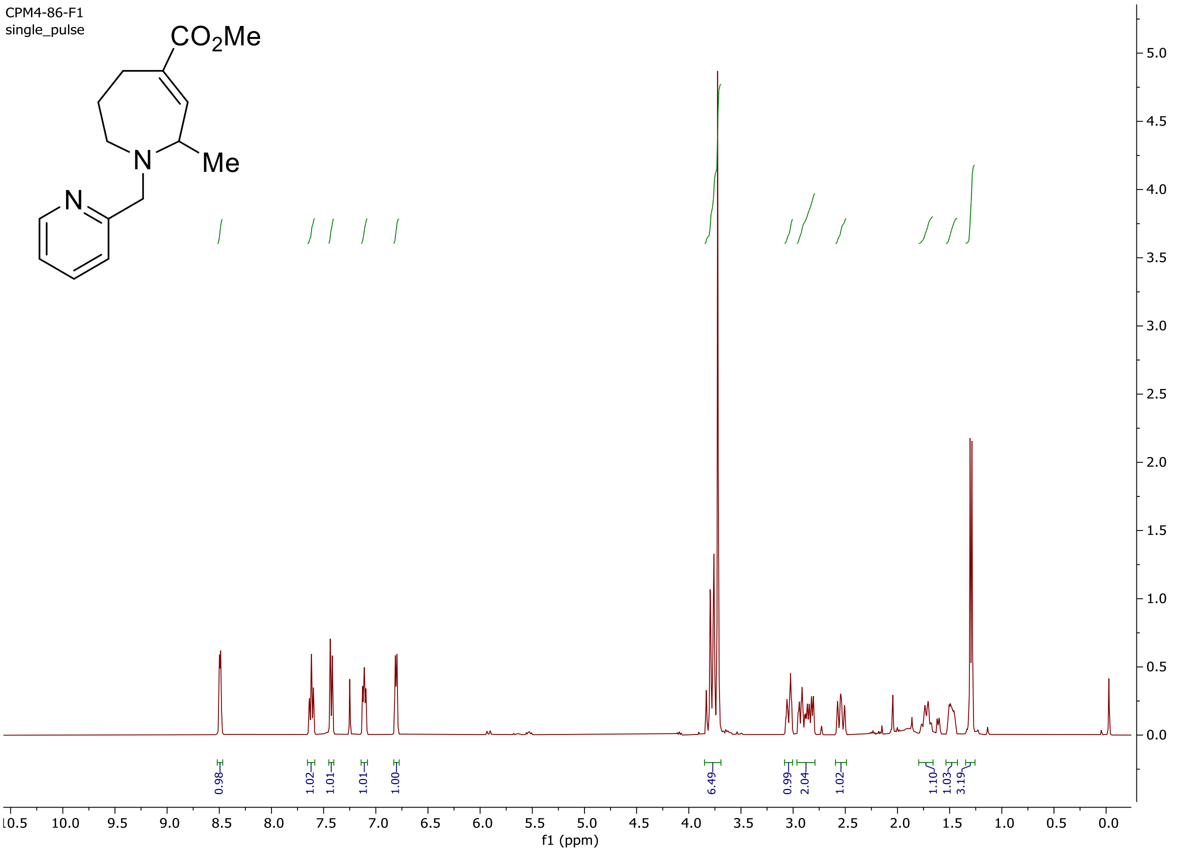

^{13}C NMR (CDCl$_3$ 100 MHz) of compound 8m

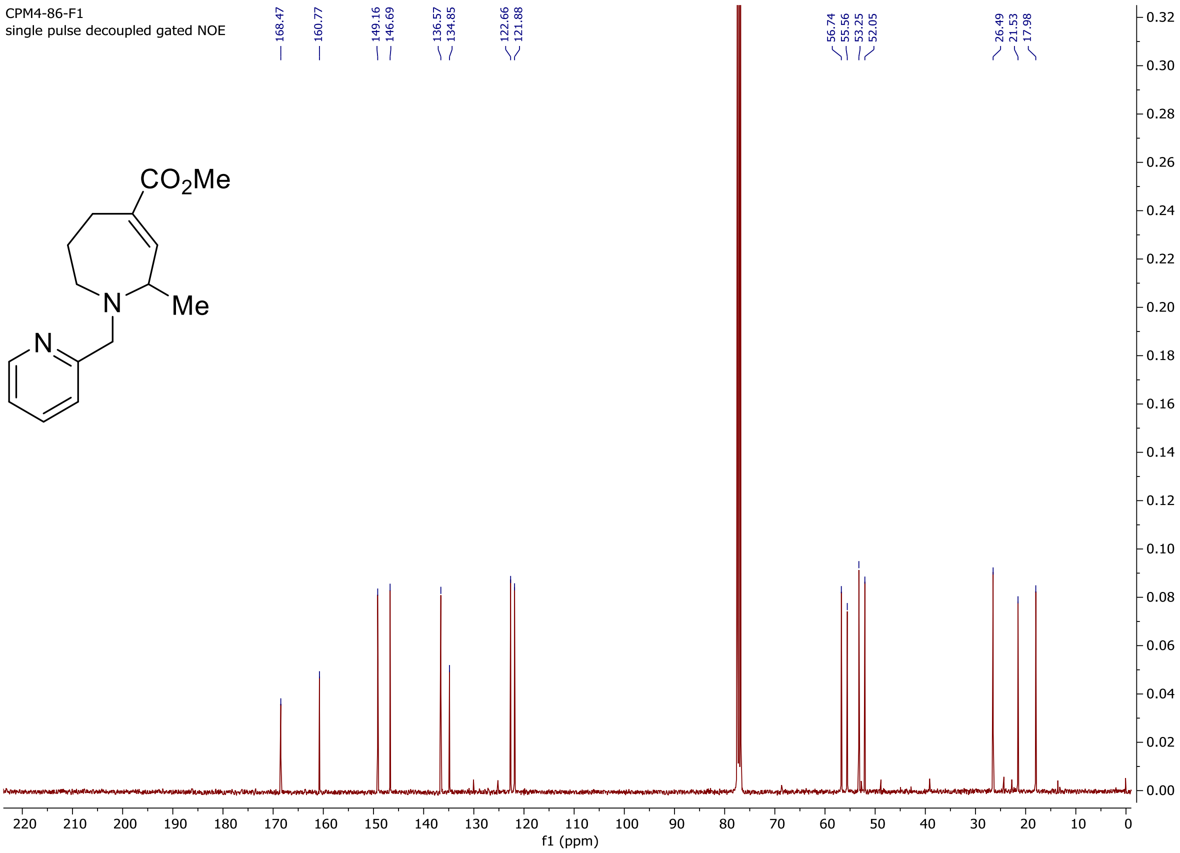

^{1}H NMR ($CDCl_3$ 400 MHz) of compound 8n

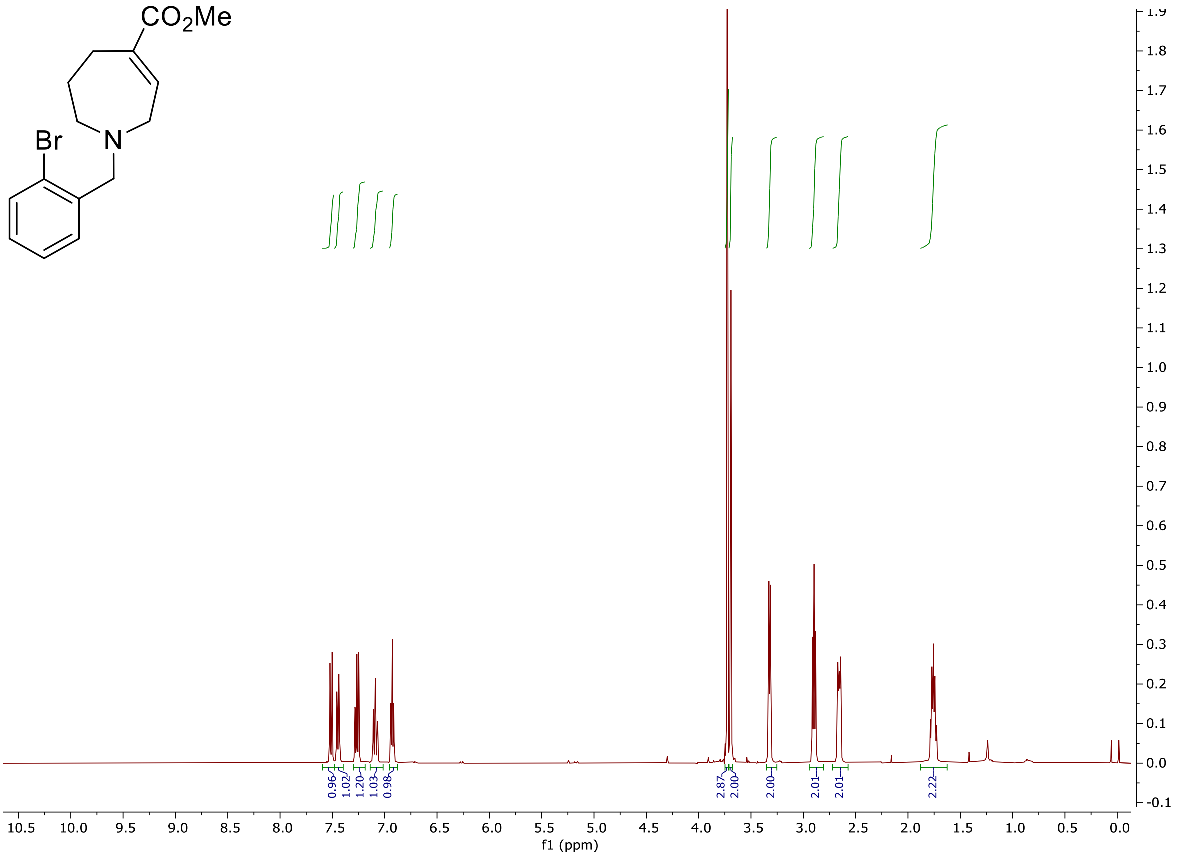

^{13}C NMR ($CDCl_3$ 100 MHz) of compound 8n

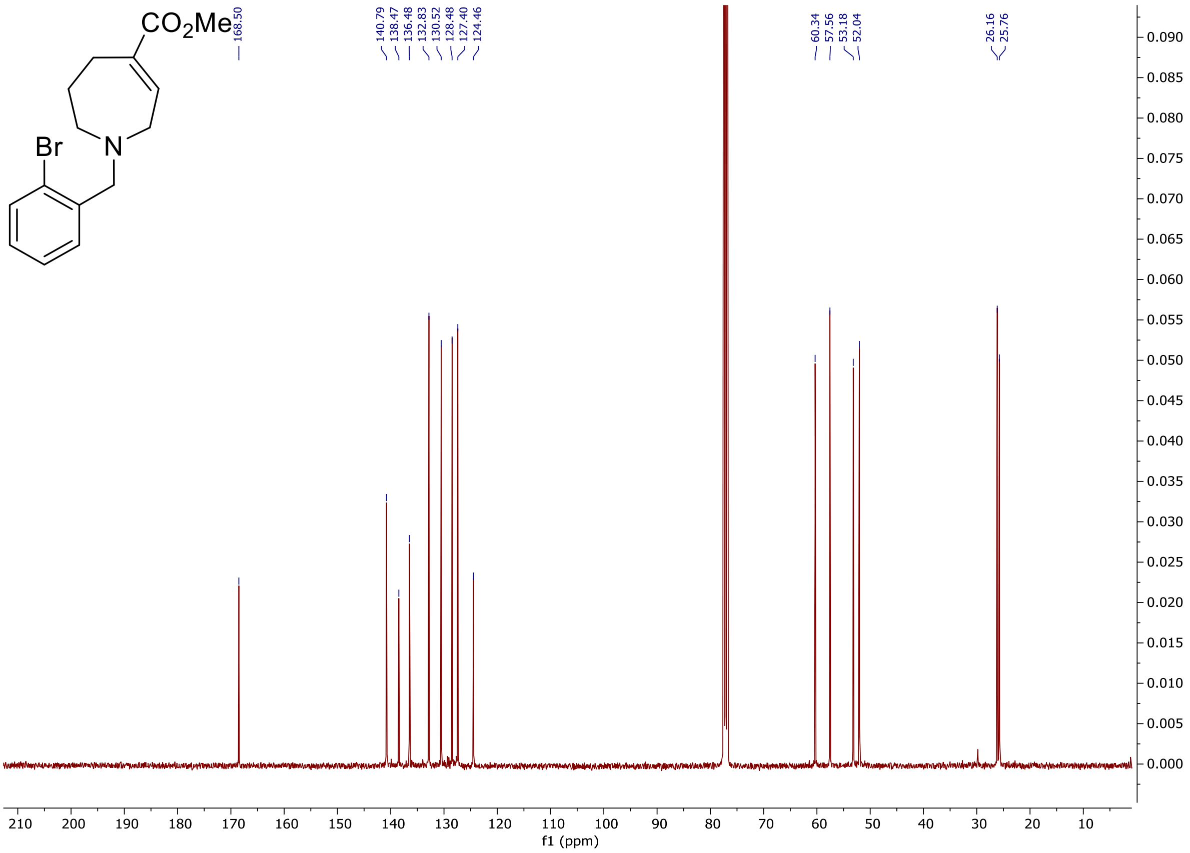

^{1}H NMR ($CDCl_3$ 400 MHz) of compound 8q

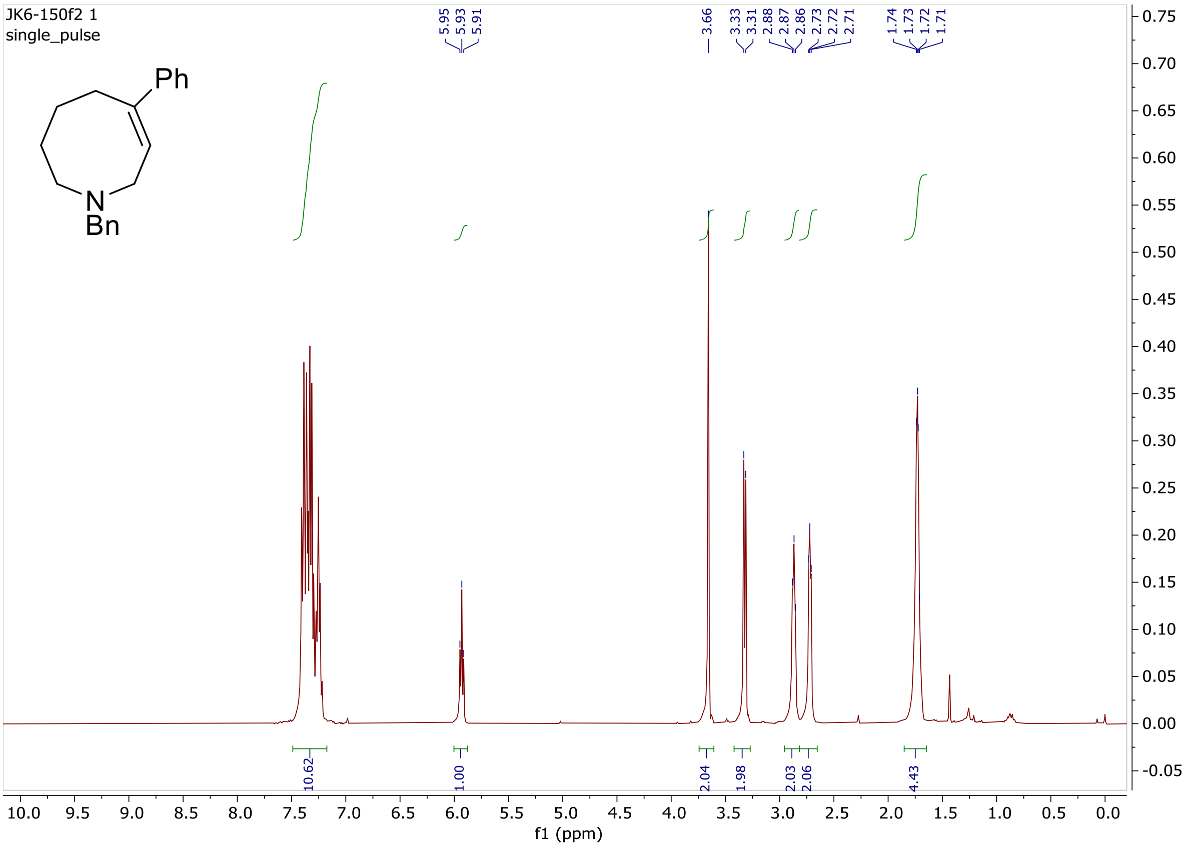

^{13}C NMR ($CDCl_3$ 100 MHz) of compound 8q

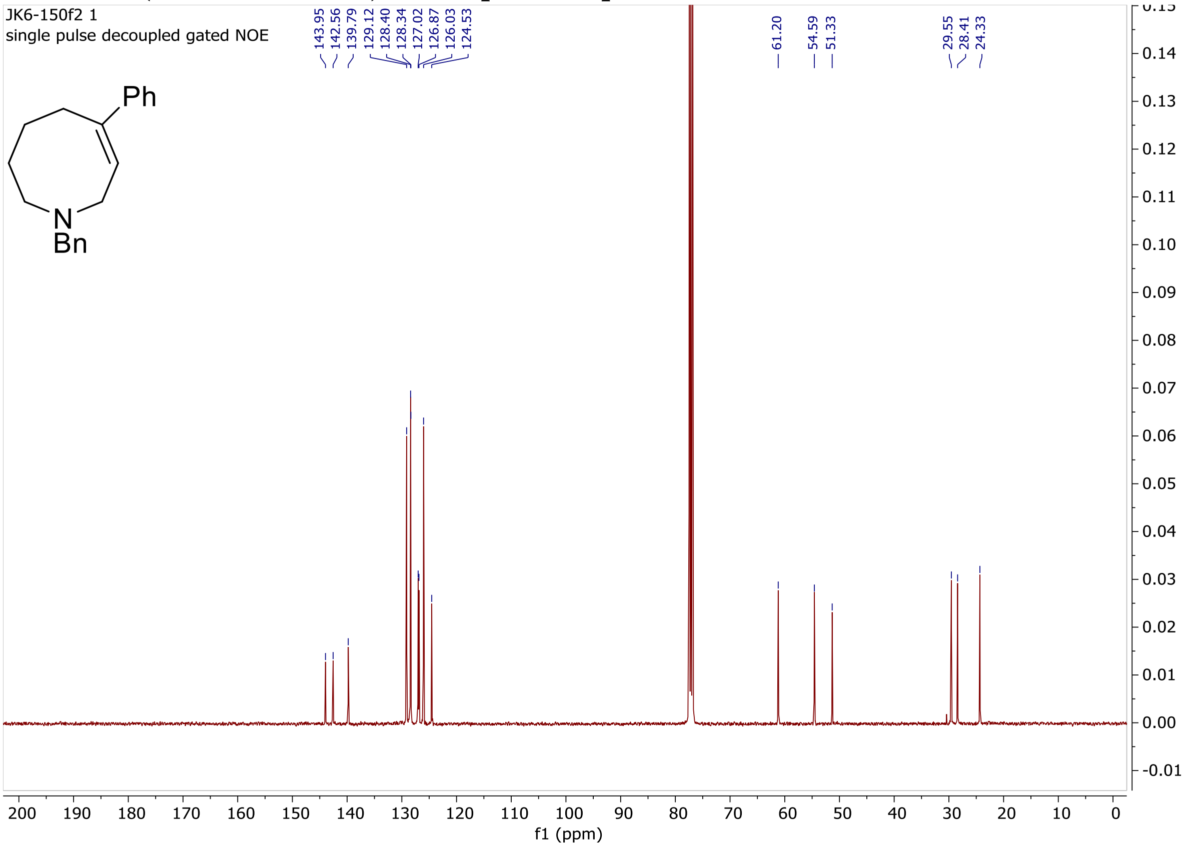

^{1}H NMR (CDCl3 400 MHz) of compound 8r

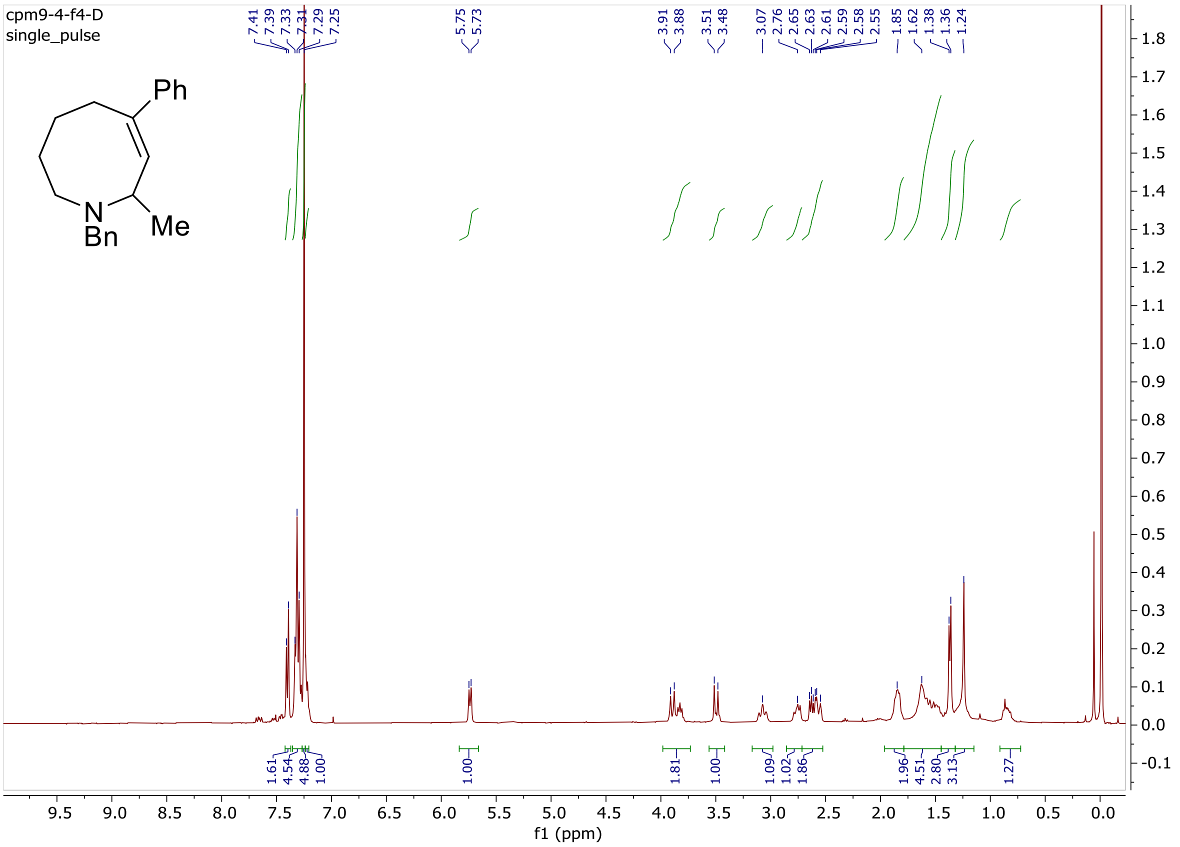

^{13}C NMR (CDCl3 100 MHz) of compound 8r

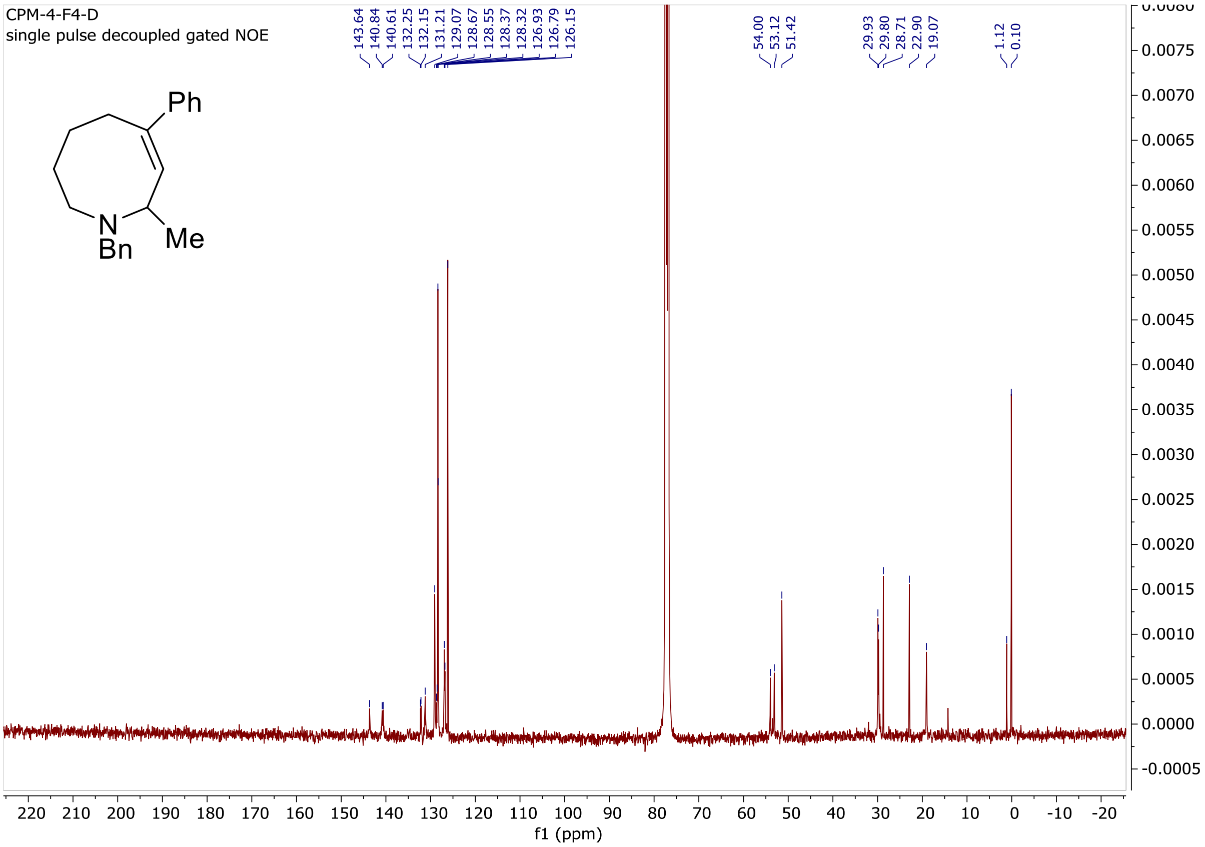

^{1}H NMR ($CDCl_3$ 400 MHz) of compound 8o

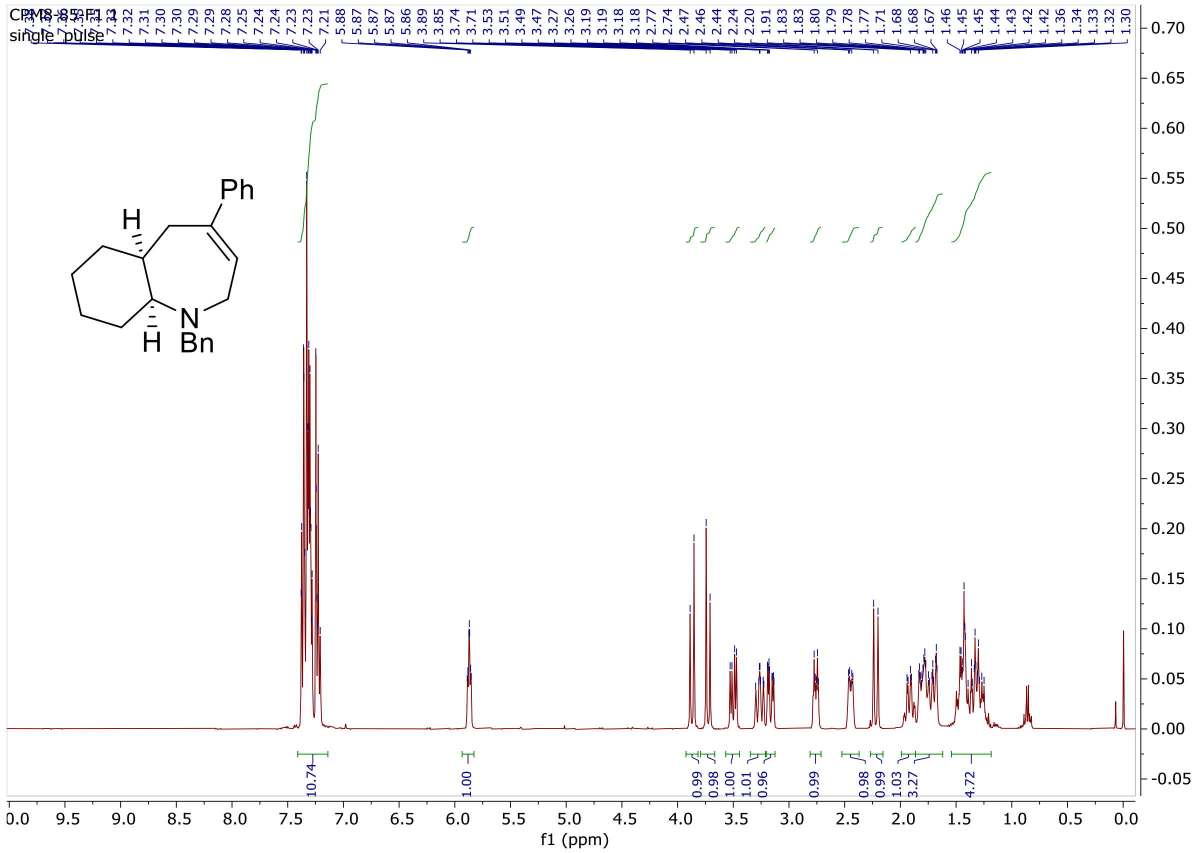

^{13}C NMR ($CDCl_3$ 100 MHz) of compound 8o

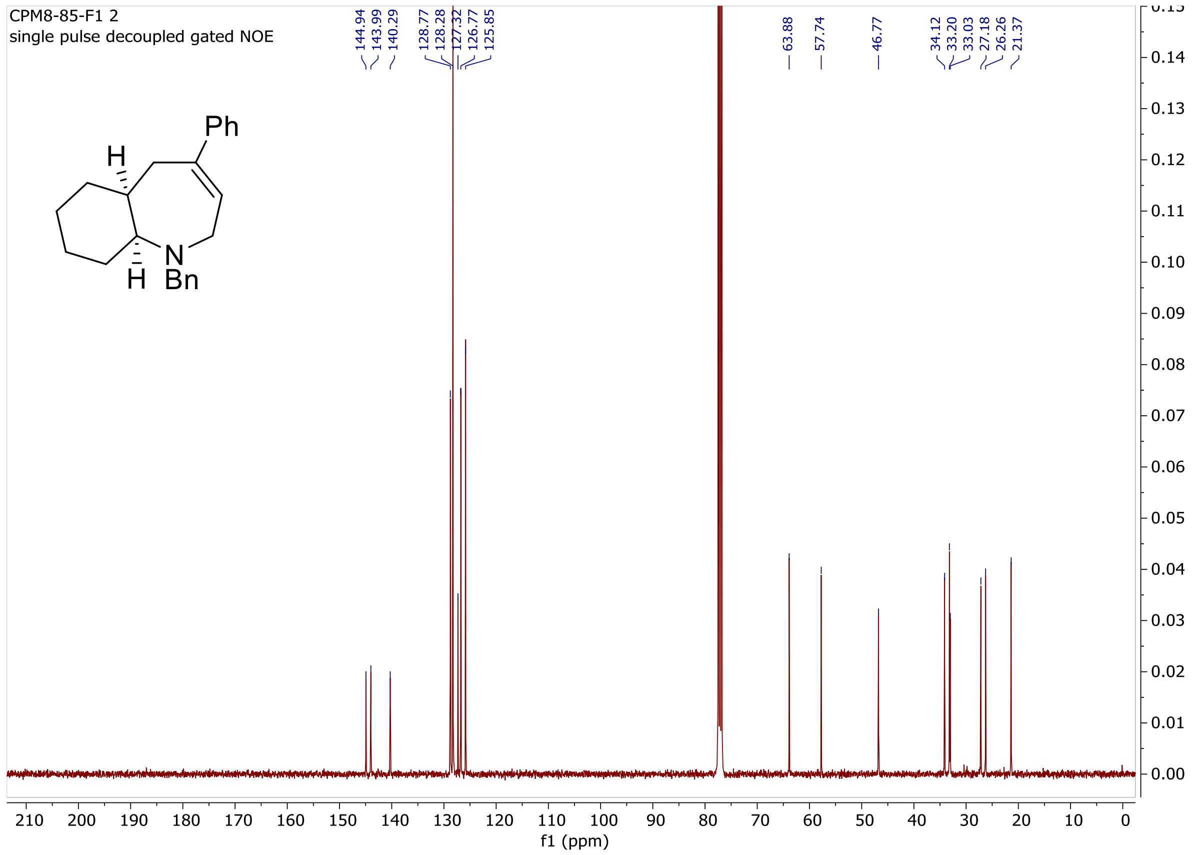

^{1}H NMR ($CDCl_3$ 400 MHz) of compound 8p

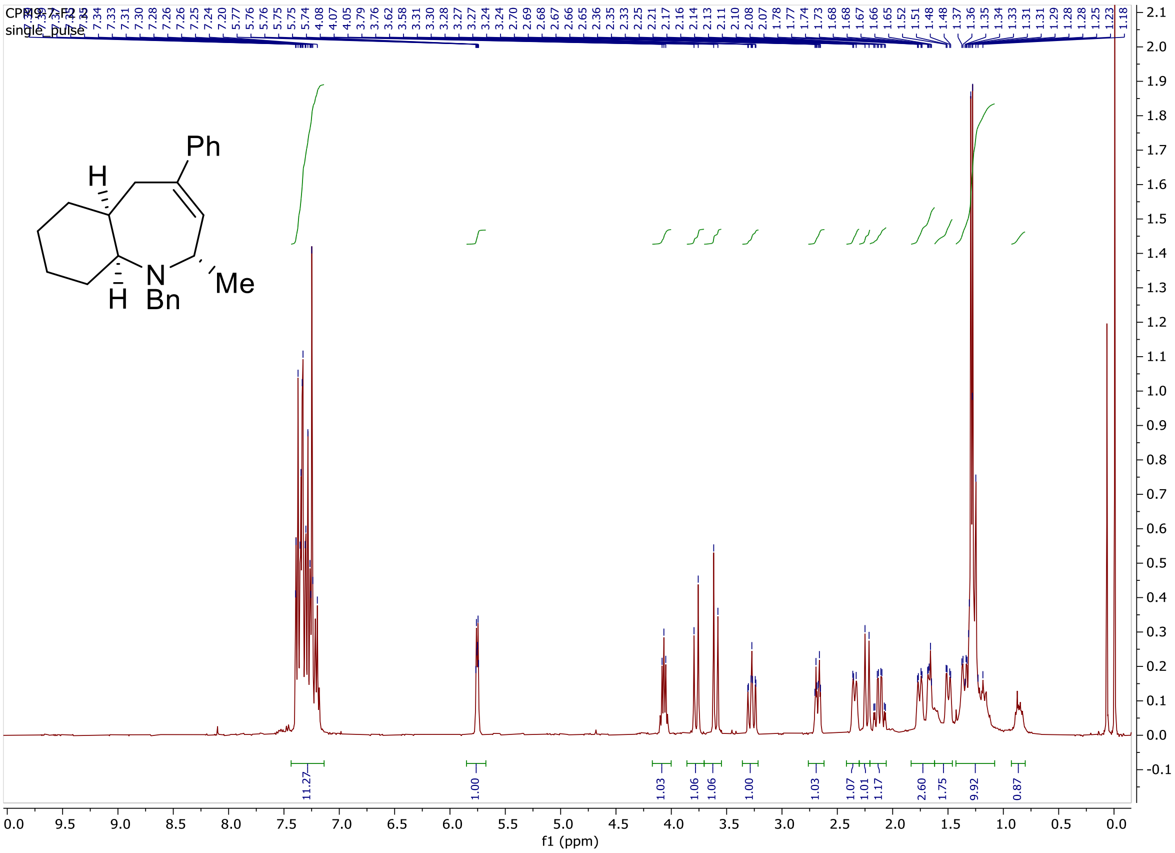

^{13}C NMR ($CDCl_3$ 100 MHz) of compound 8p

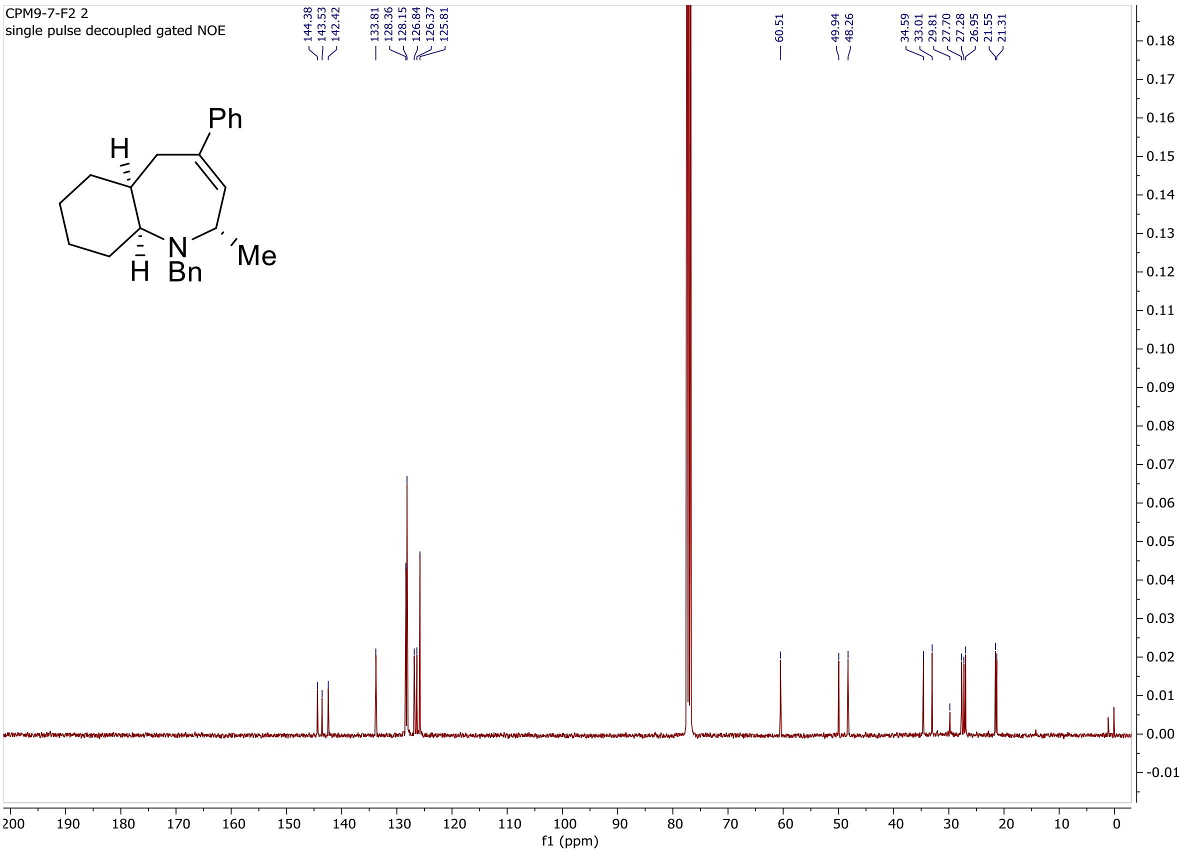

^{1}H COSY interactions of compound 8p

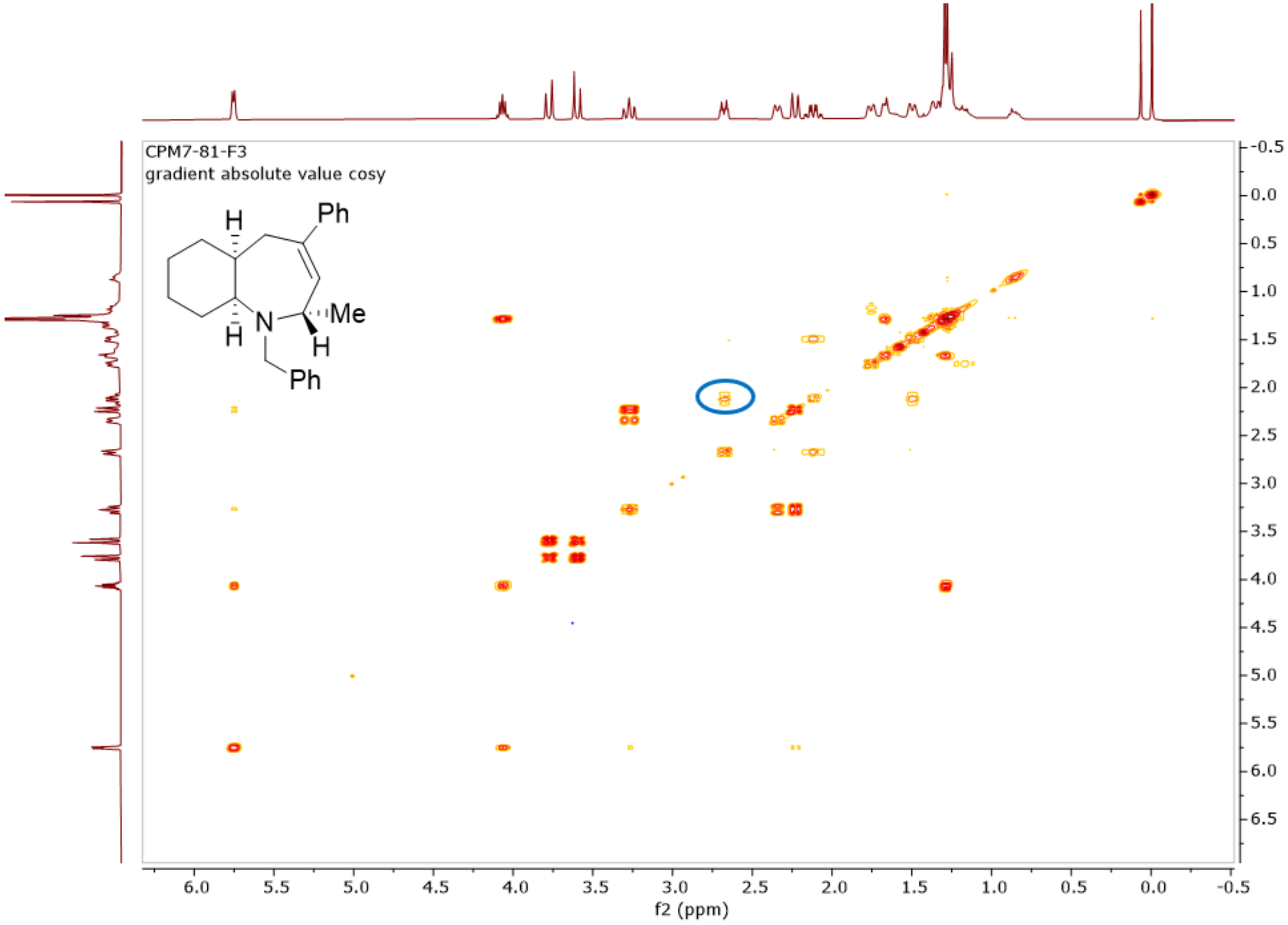

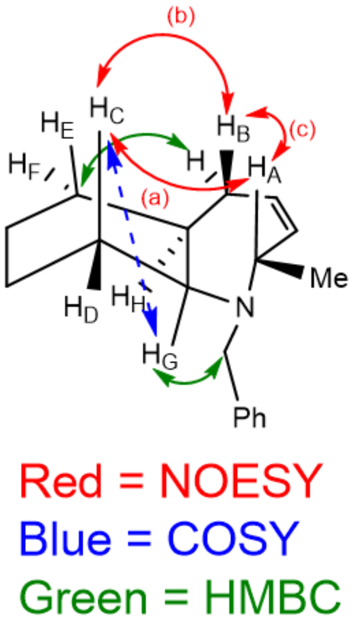

^{1}H-^{13}C HSQC interactions of compound 8p

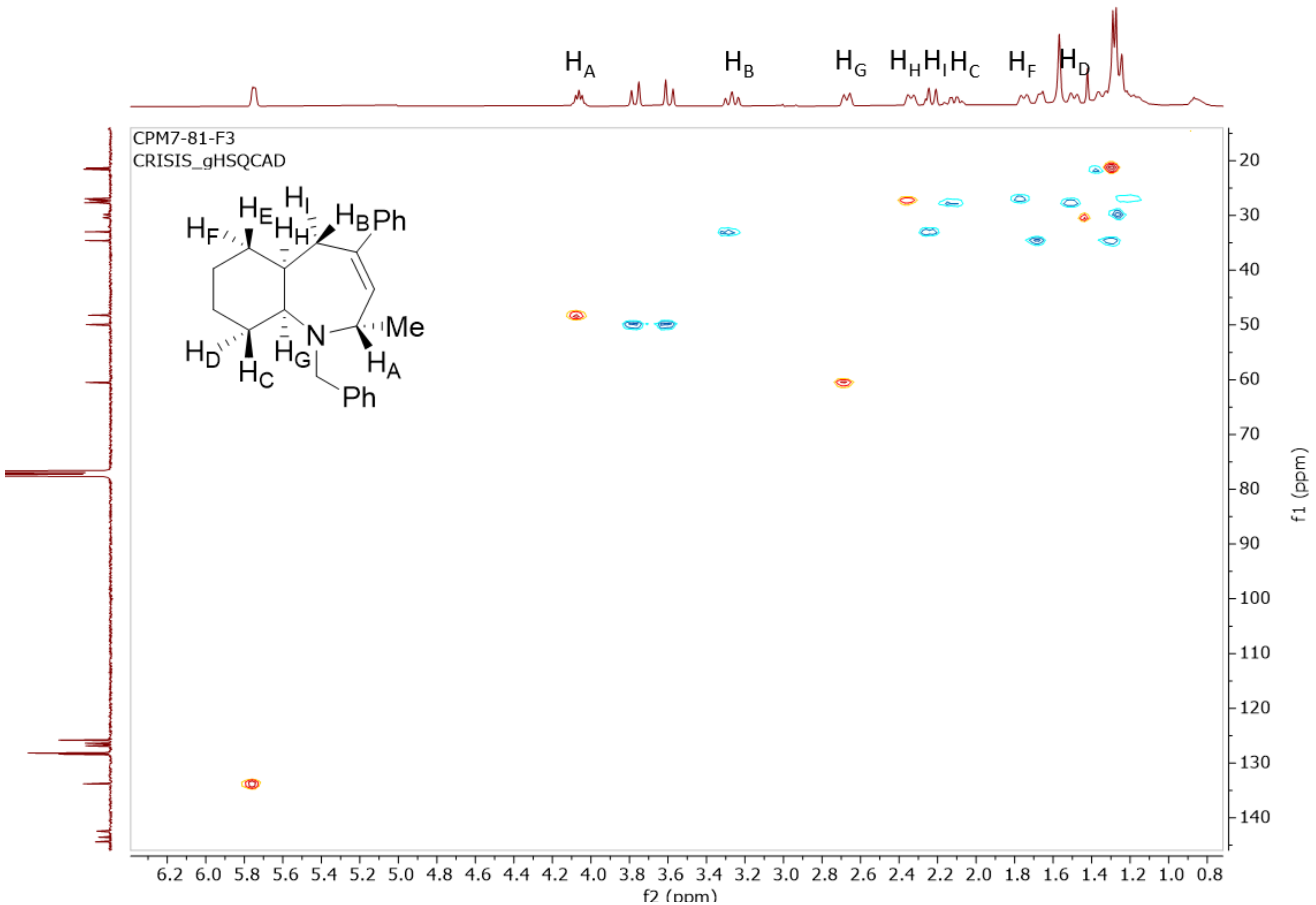

^{1}H-^{13}C HMBC interactions of compound 8p

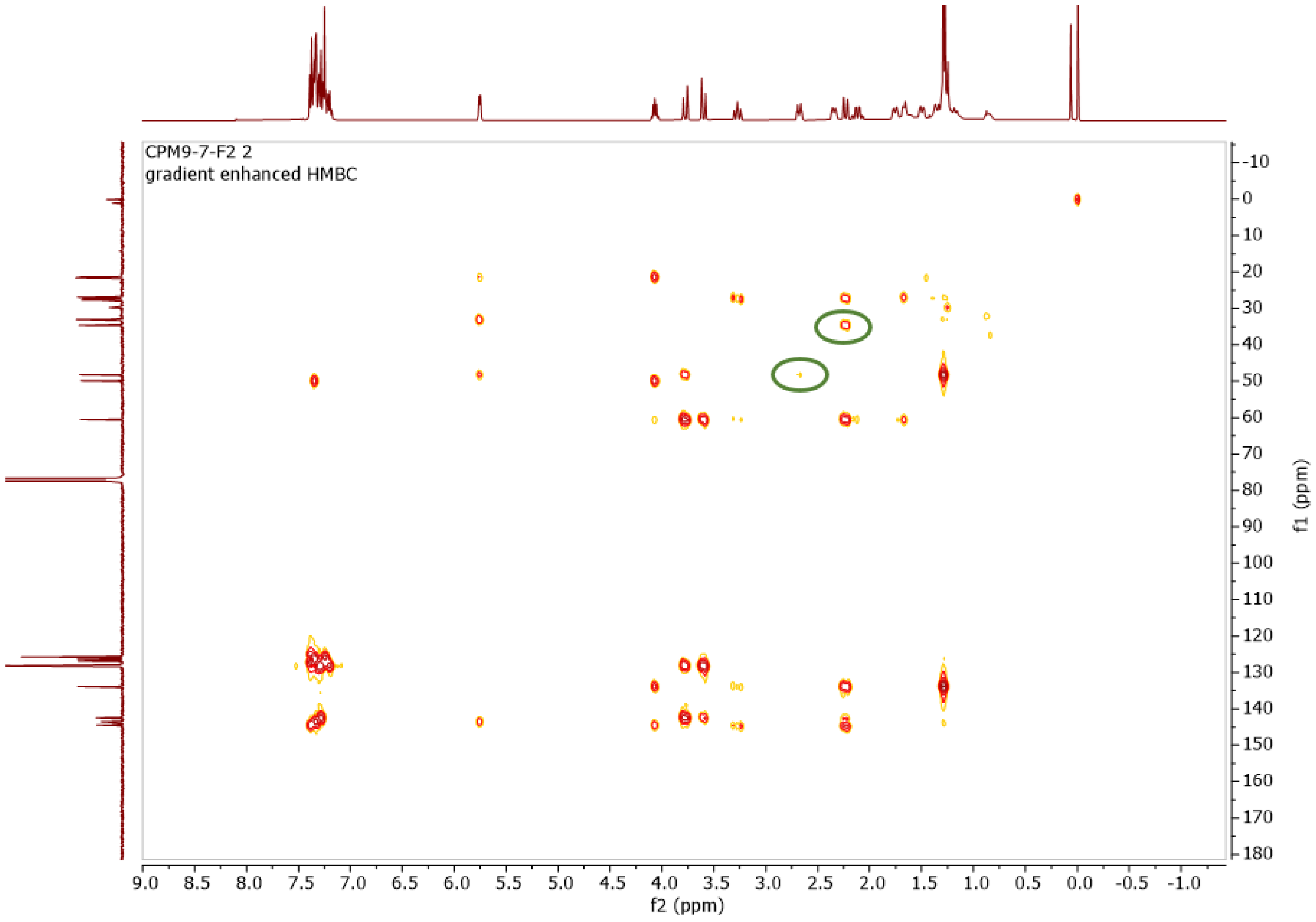

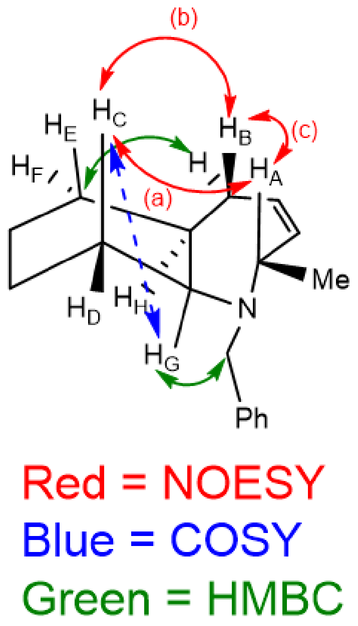

[1]H-NOESY interactions of bicyclic amine 8p

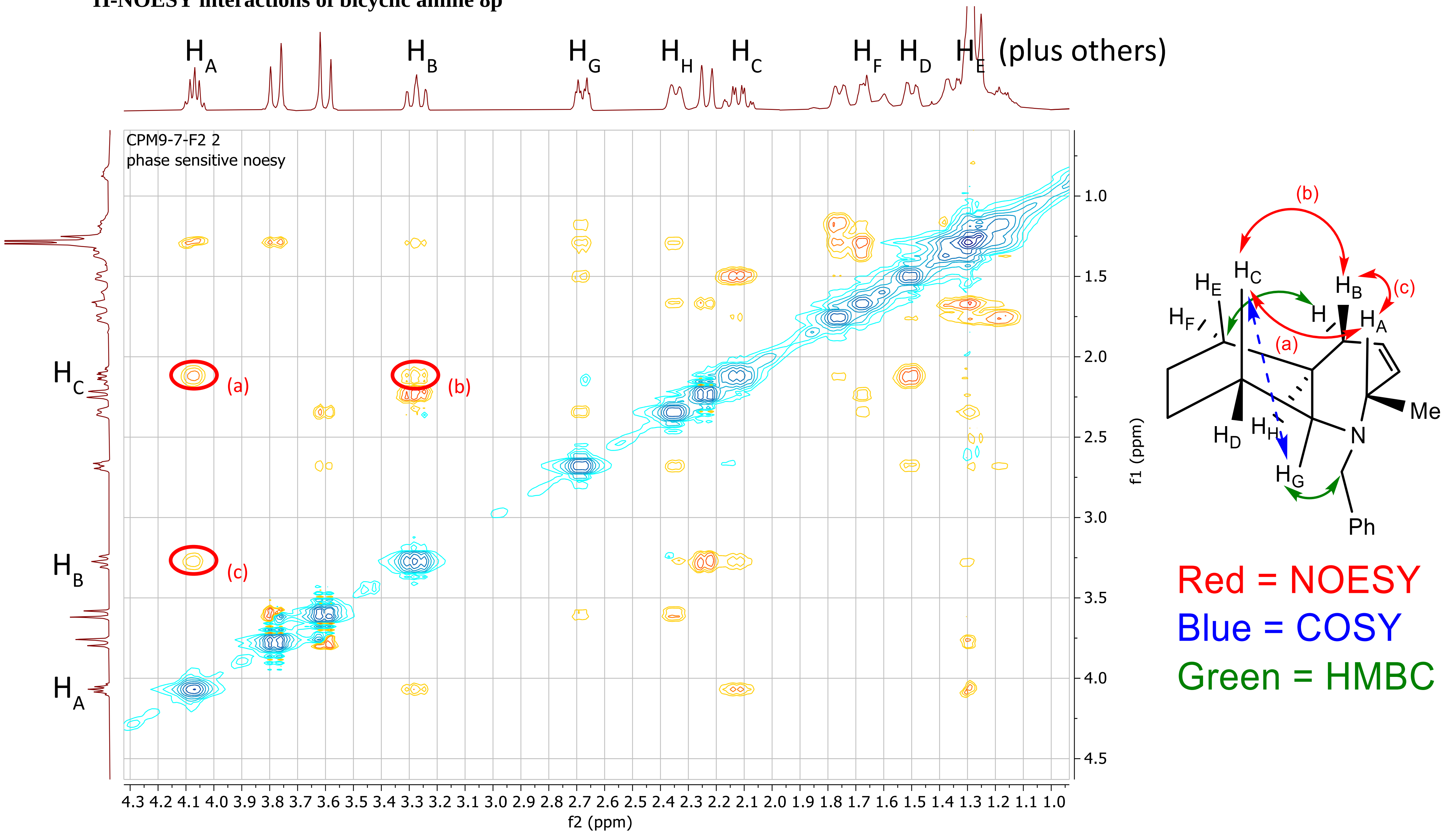

^{1}H NMR (CDCl3 400 MHz) of compound 16a

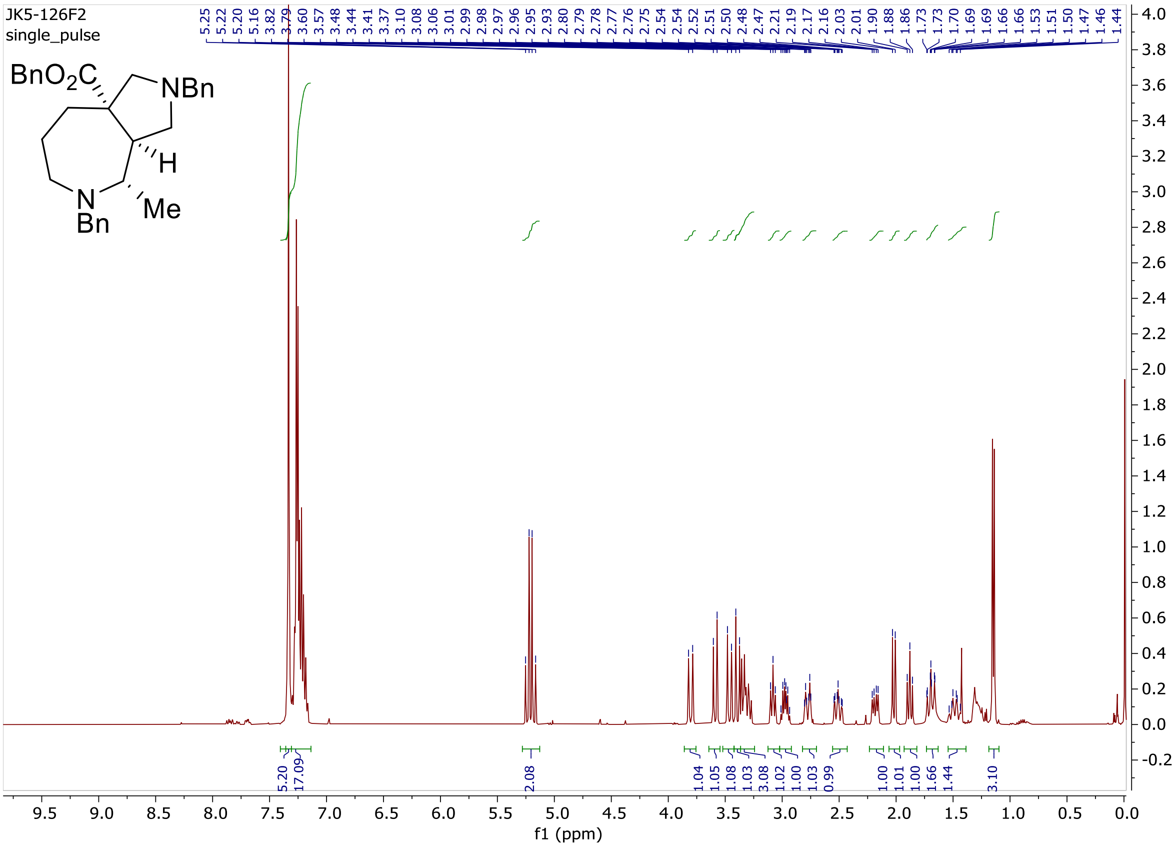

^{13}C NMR (CDCl3 100 MHz) of compound 16a

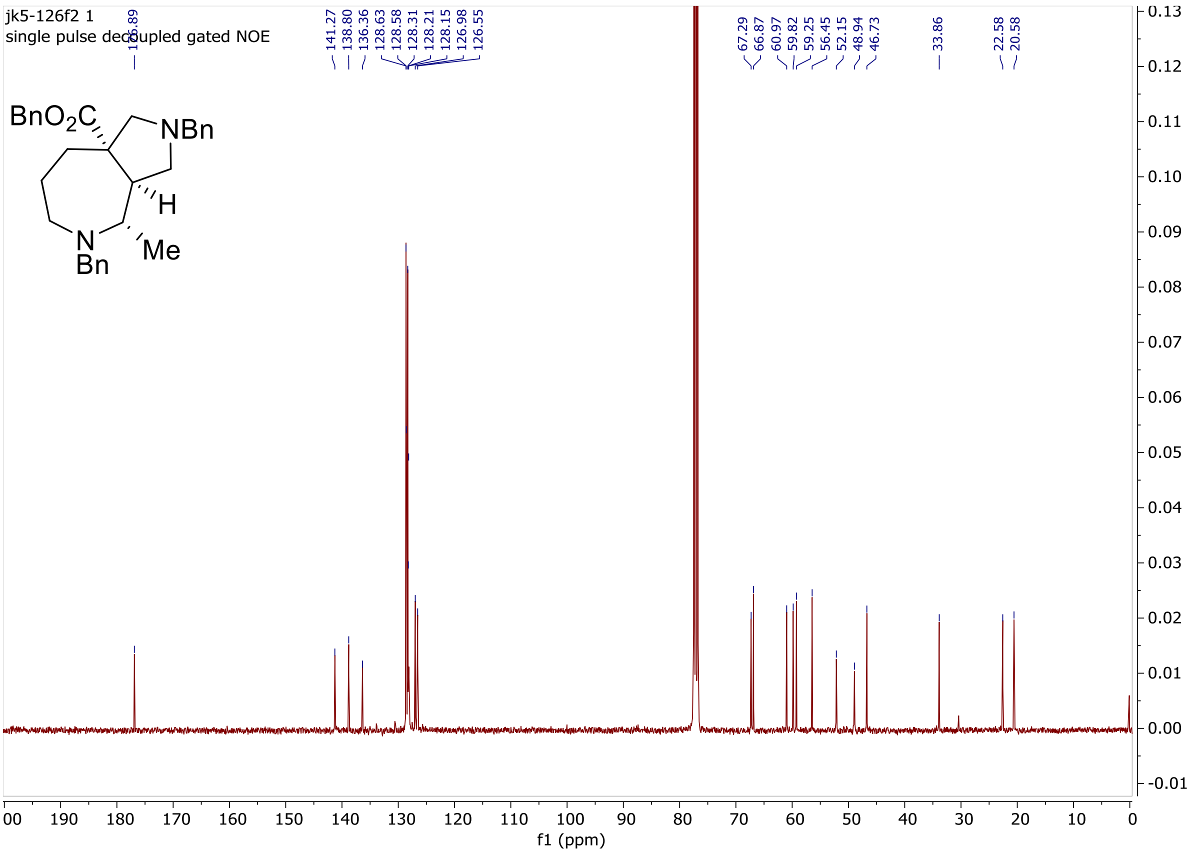

^{1}H COSY interactions of compound 16a

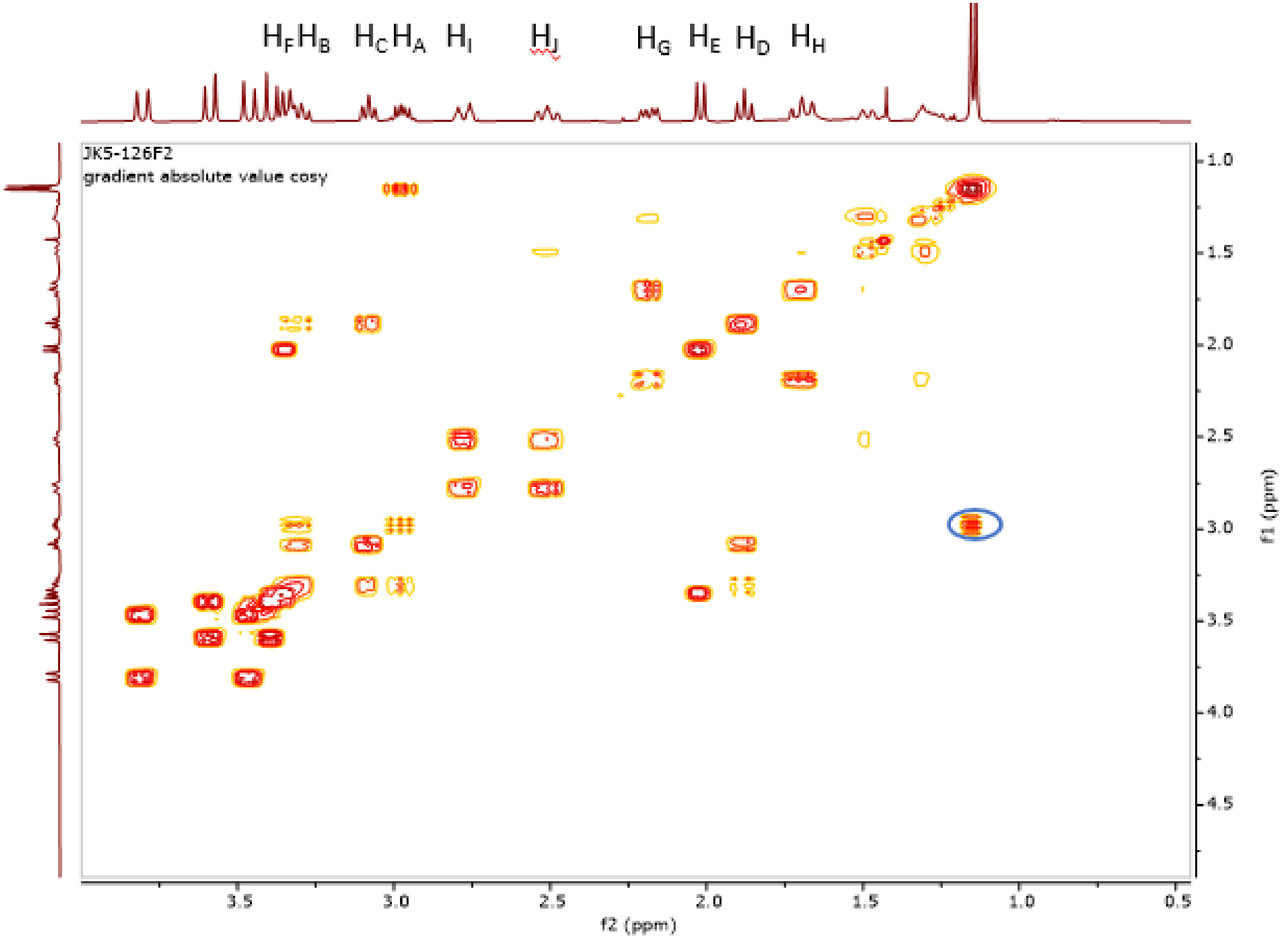

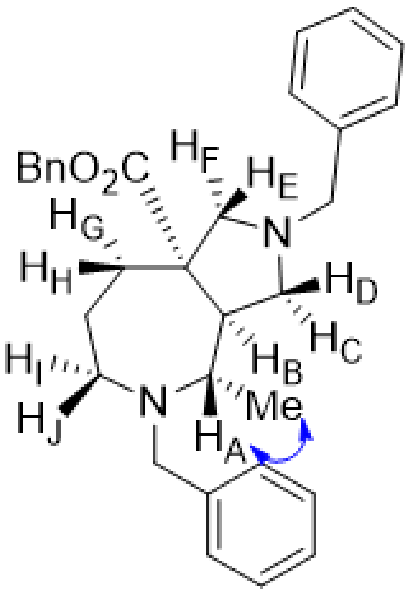

^{1}H-^{13}C HSQC interactions of compound 16a

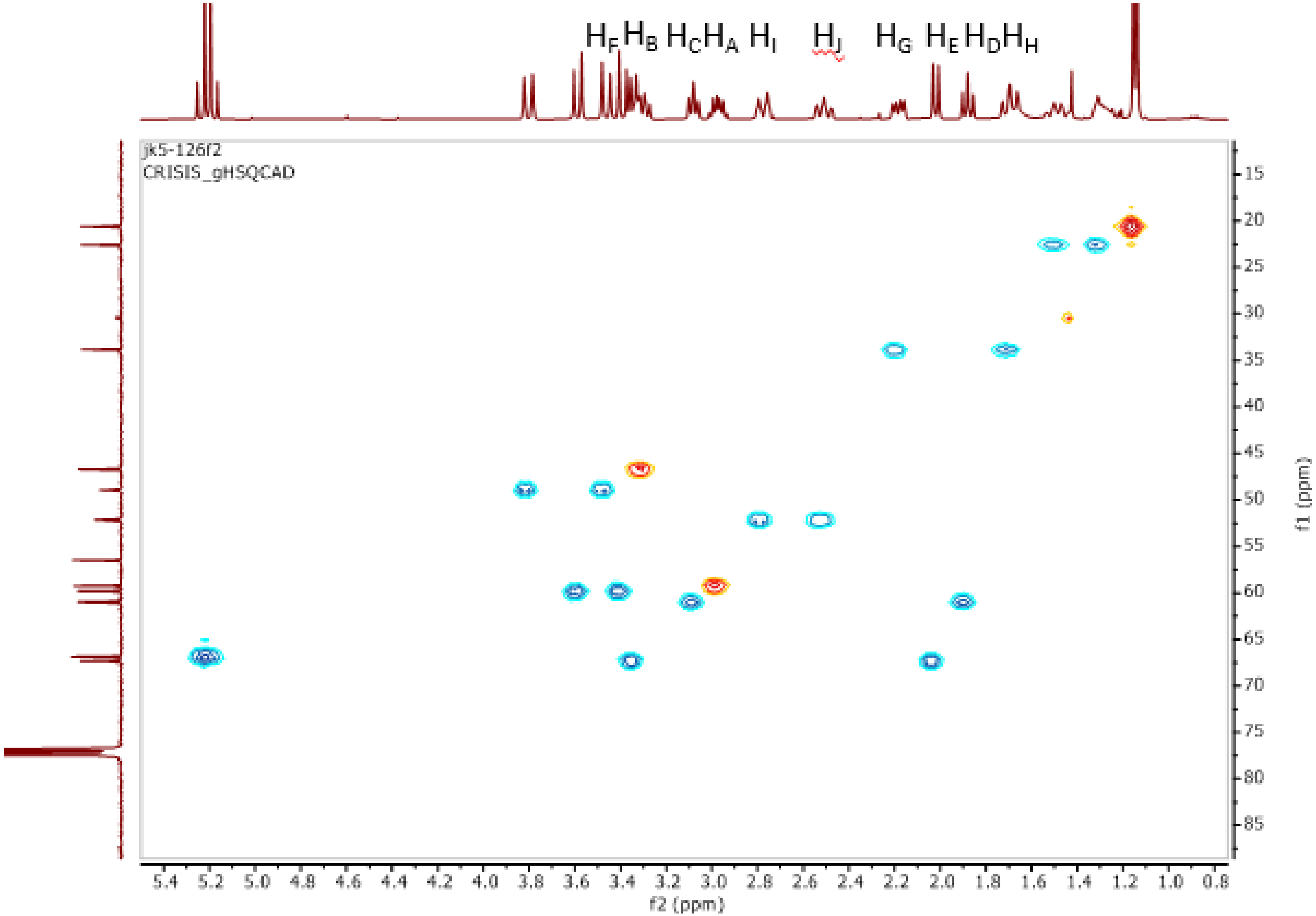

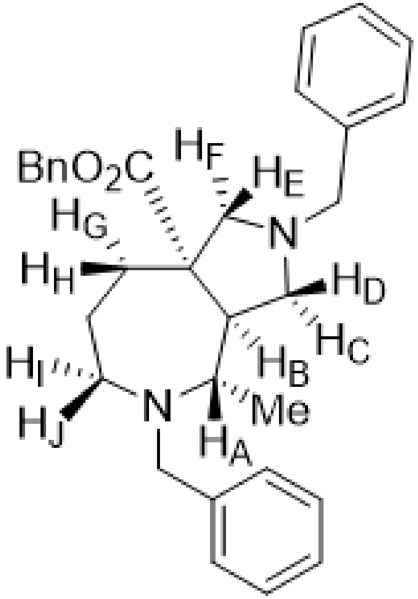

^{1}H-^{13}C HMBC interactions of compound 16a

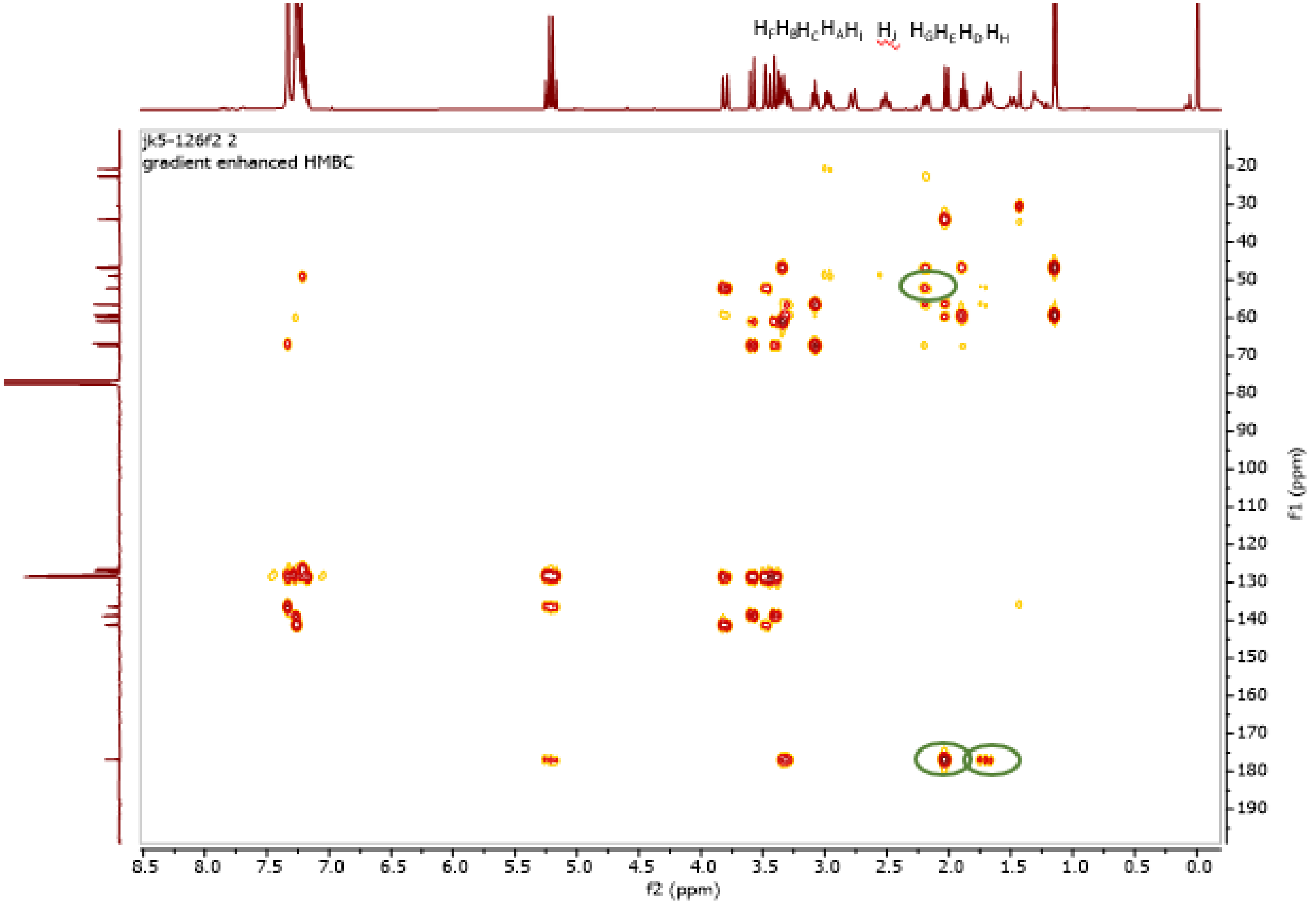

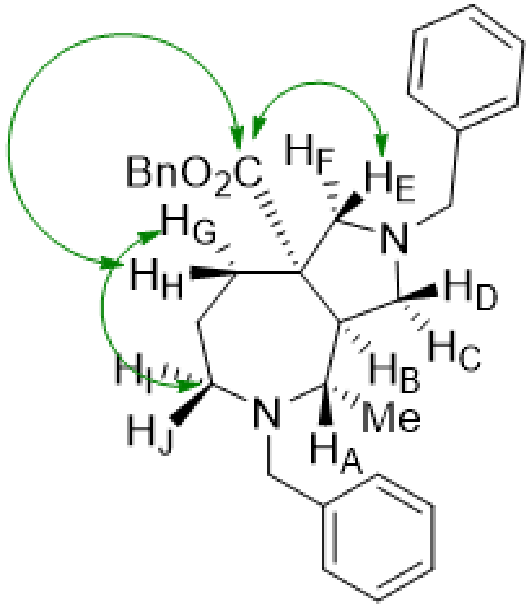

[1]H NOESY interactions of bicyclic amine 16a

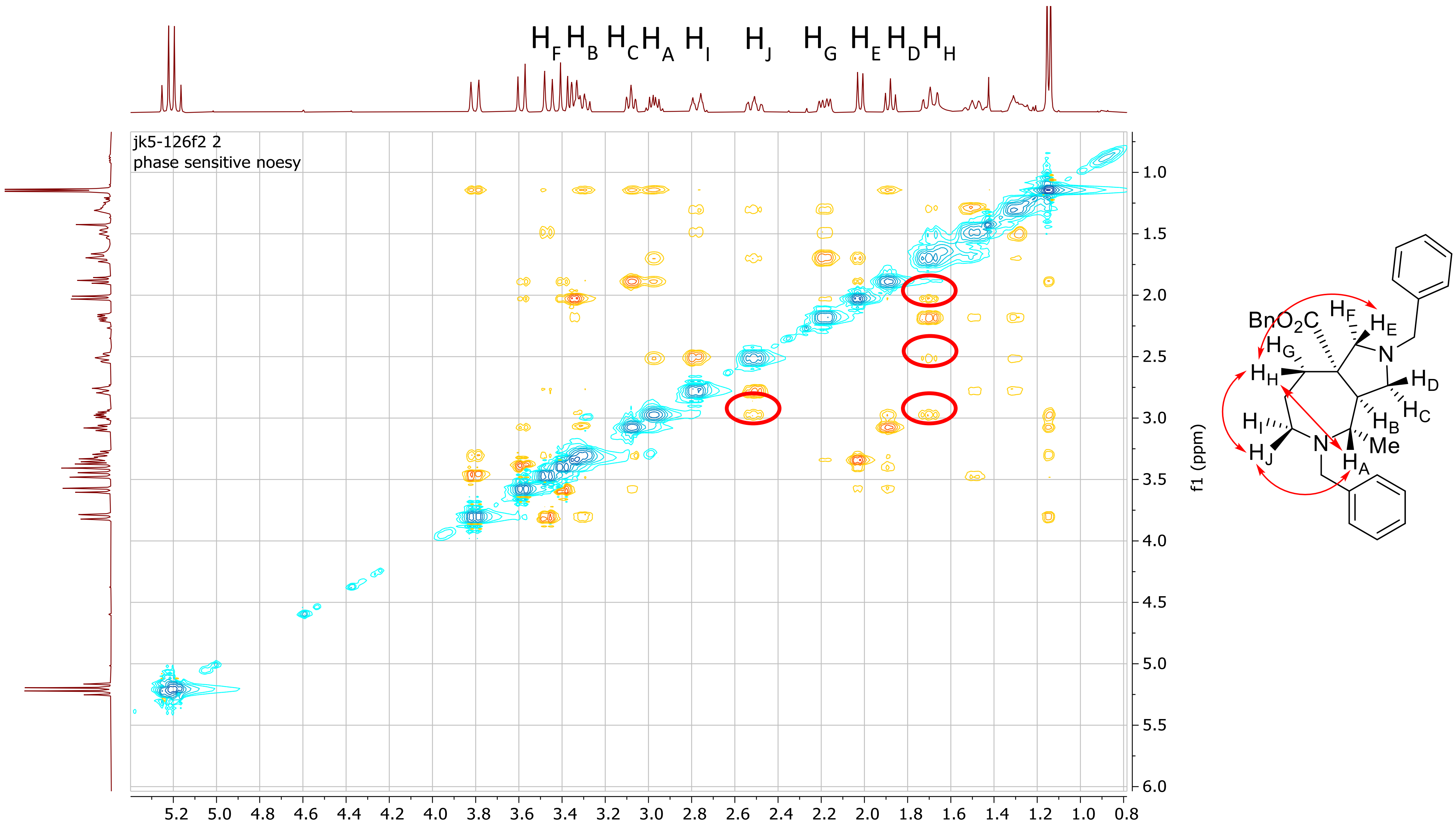

^{1}H NMR ($CDCl_3$ 400 MHz) of compound 16b

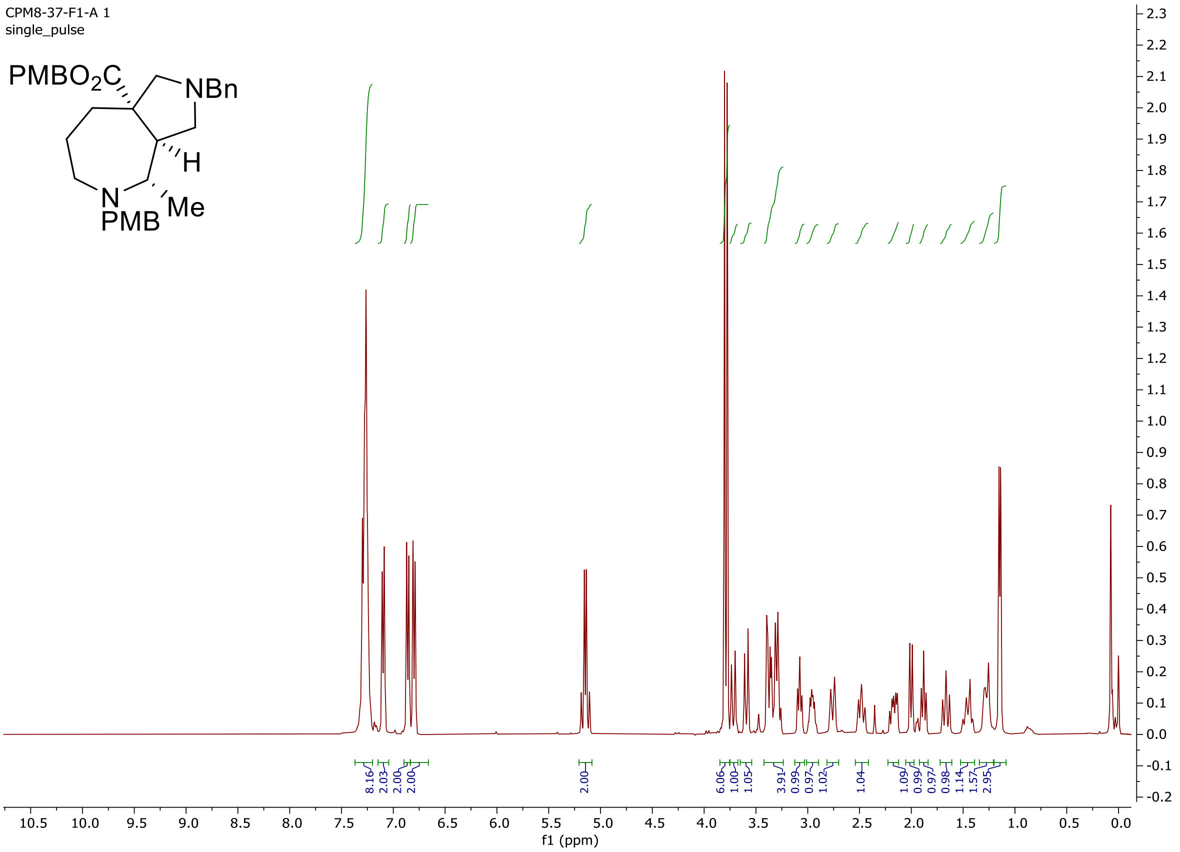

^{13}C NMR ($CDCl_3$ 100 MHz) of compound 16b

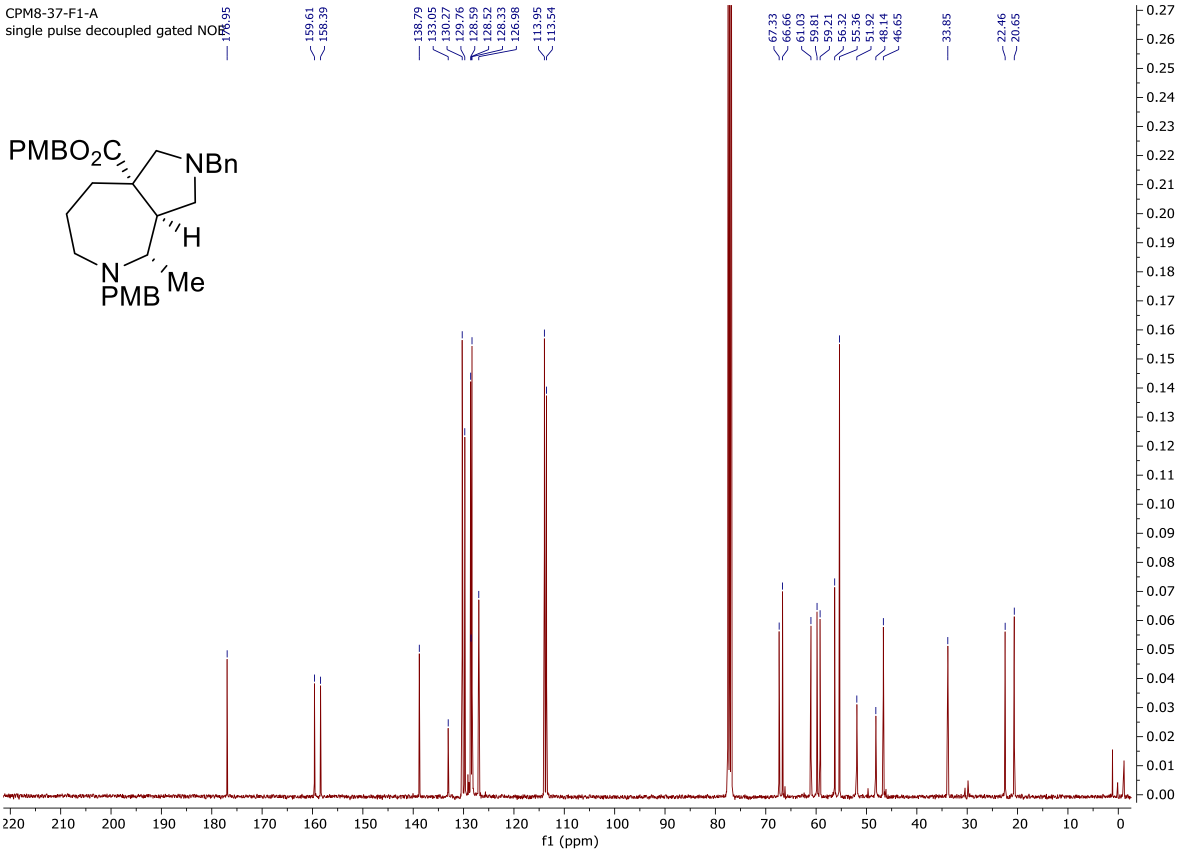

^{1}H COSY interactions of bicyclic amine 16b

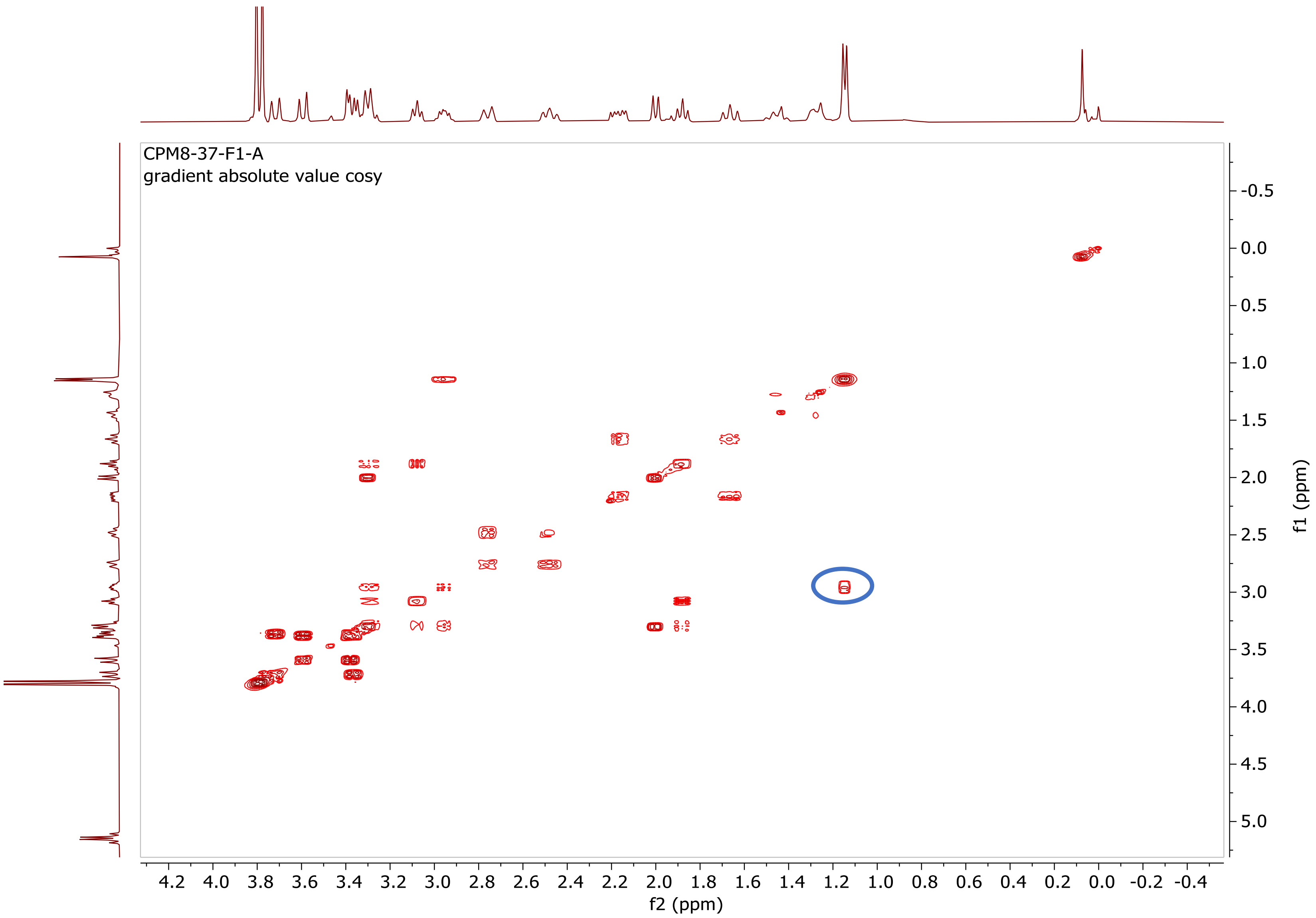

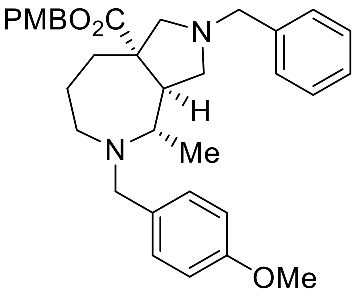

^{1}H-^{13}C HSQC interactions of bicyclic amine 16b

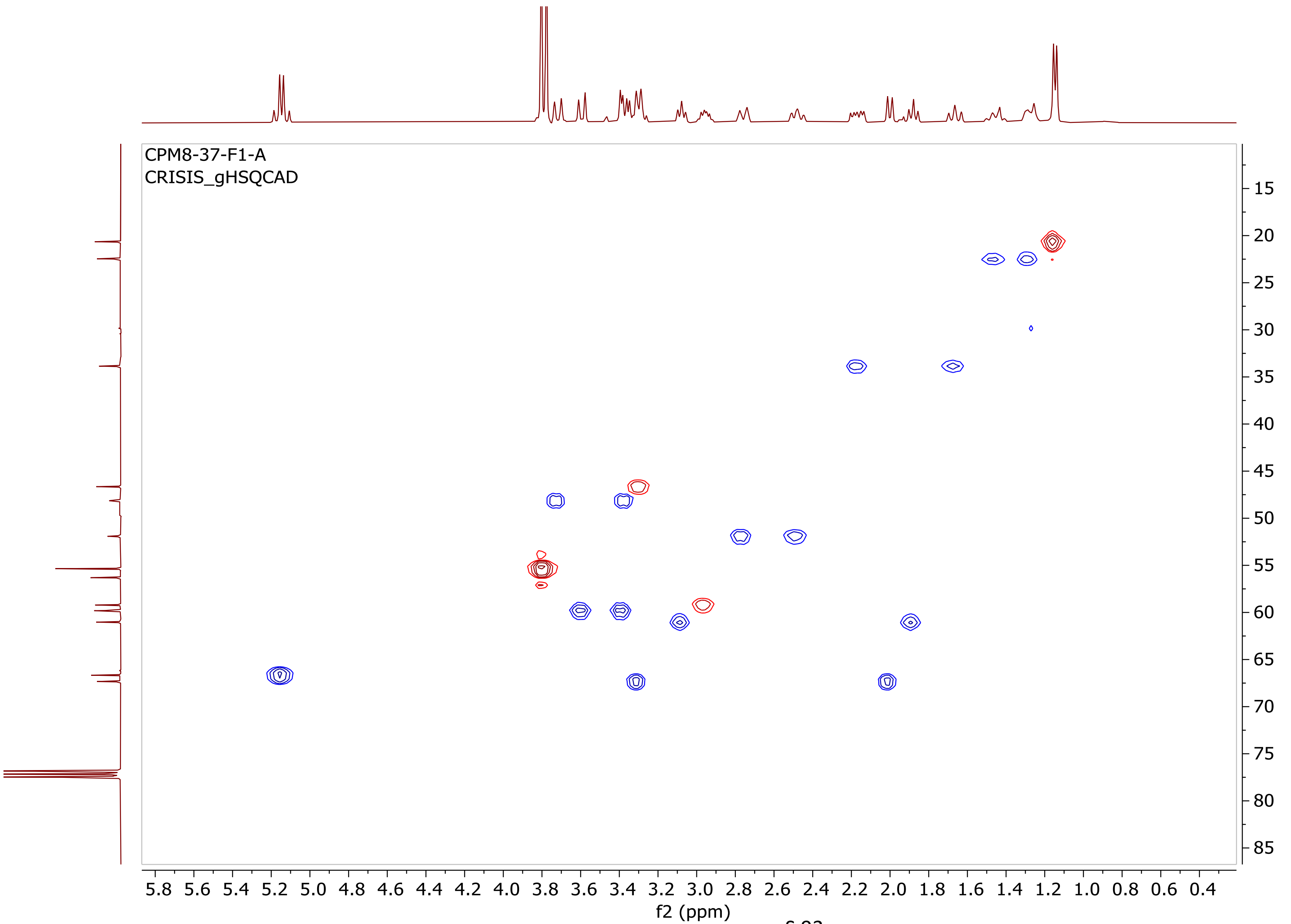

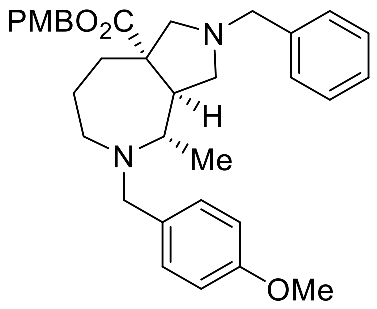

[1]H-NOESY interactions of bicyclic amine 16b

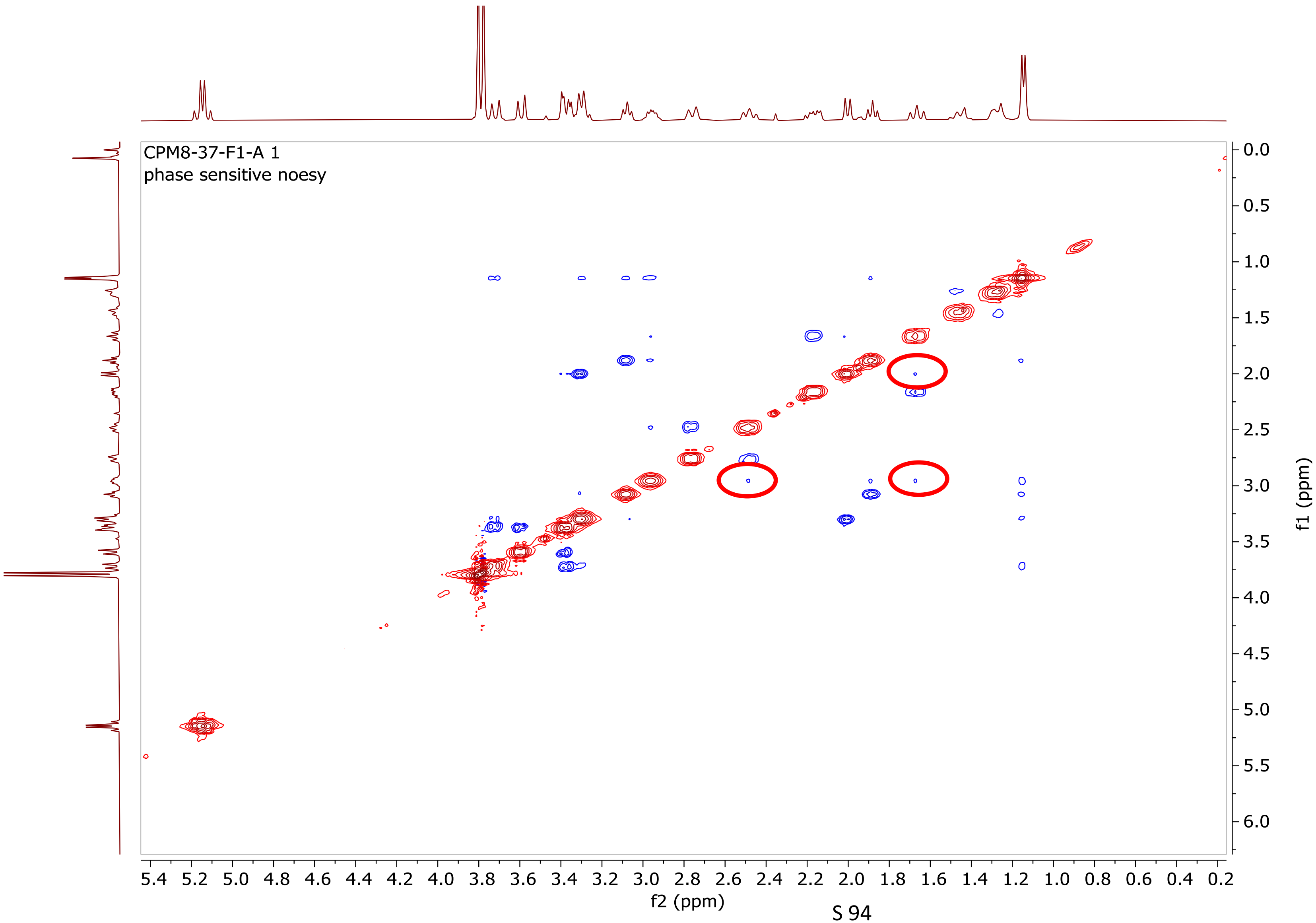

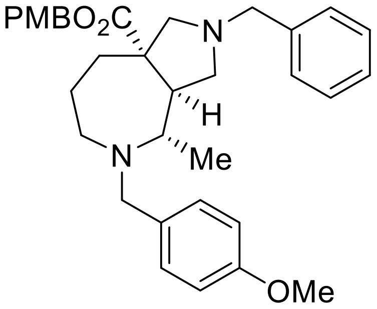

^{1}H NMR (CDCl$_3$ 400 MHz) of compound 17

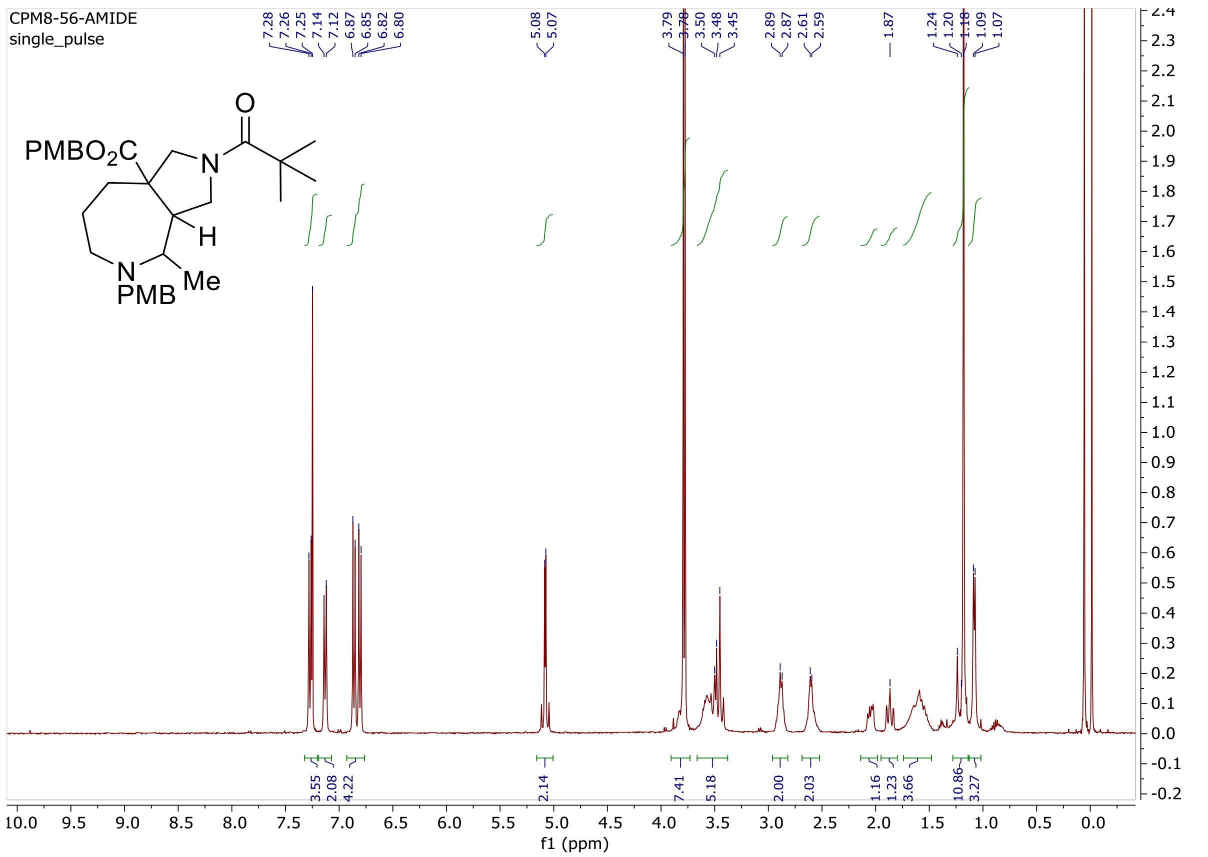

^{13}C NMR (CDCl$_3$ 100 MHz) of compound 17

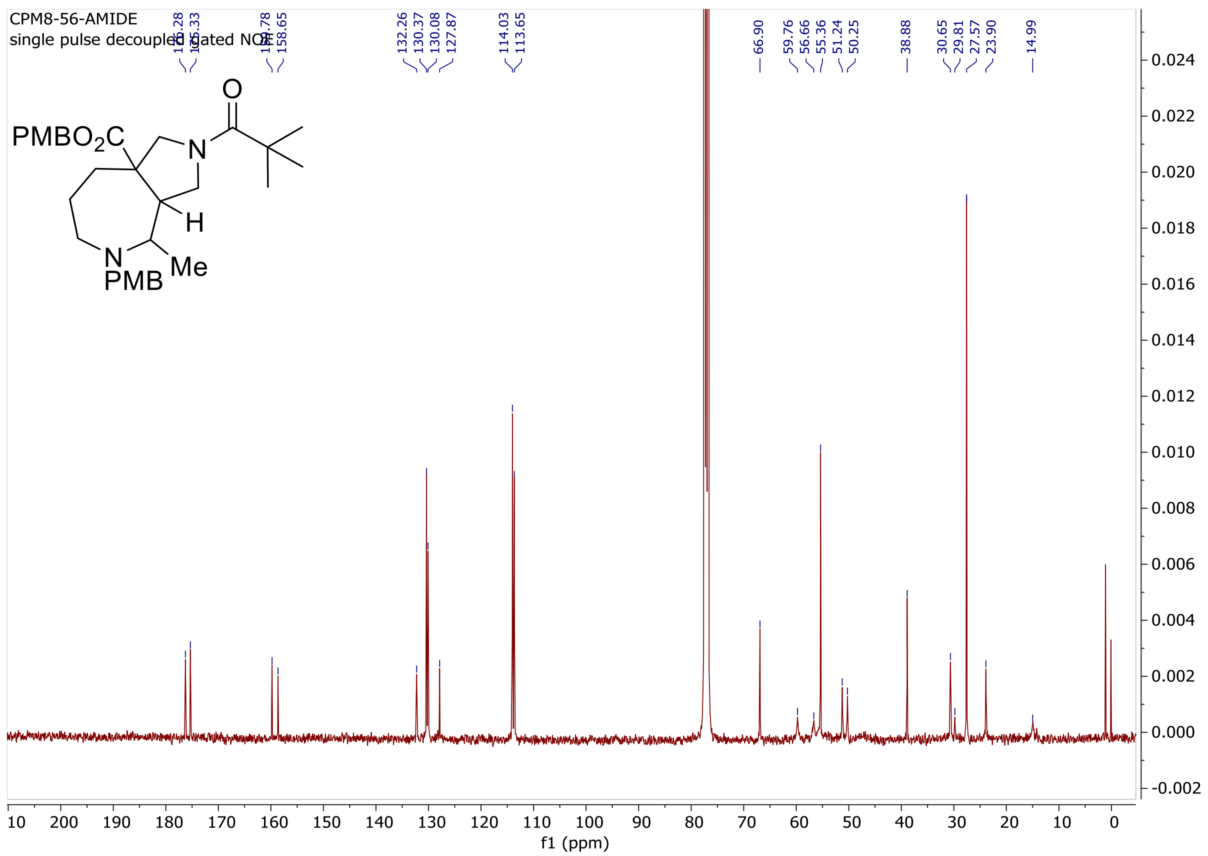

^{1}H NMR (CDCl$_3$ 400 MHz) of compound 18

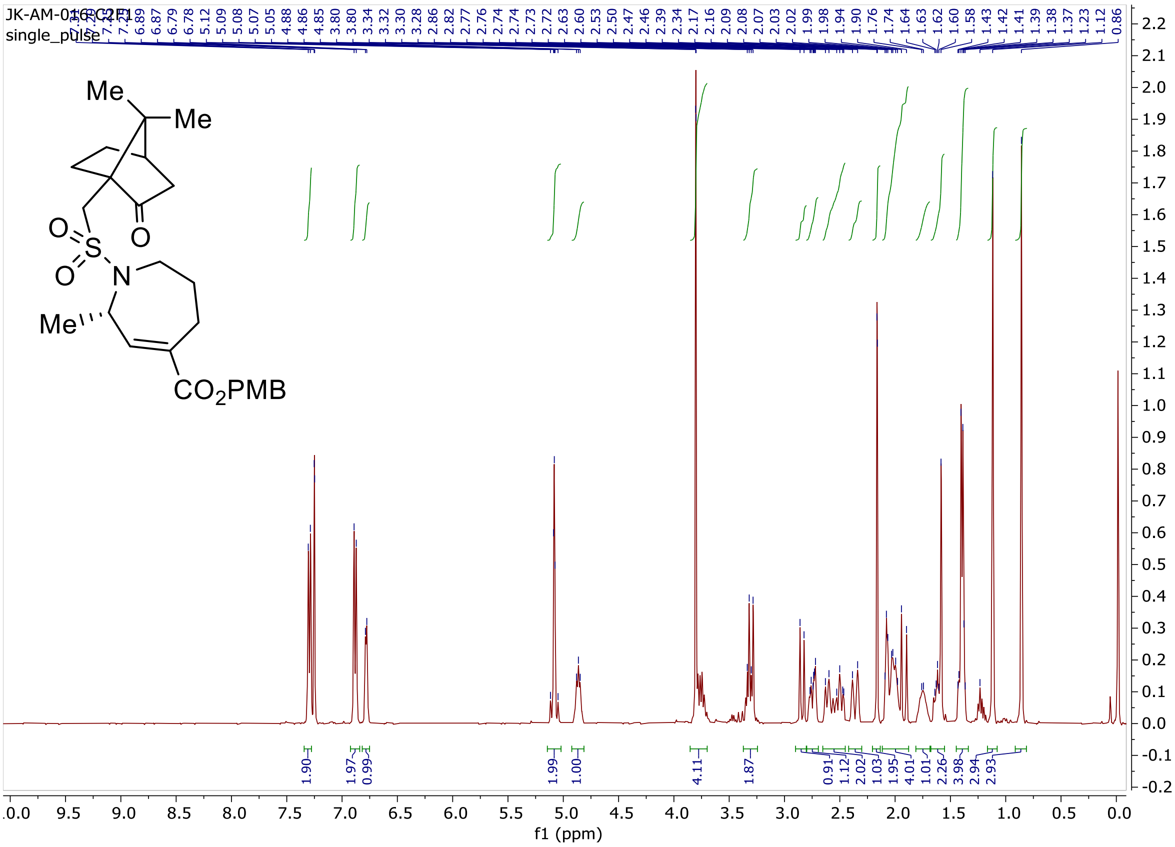

^{13}C NMR (CDCl$_3$ 100 MHz) of compound 18

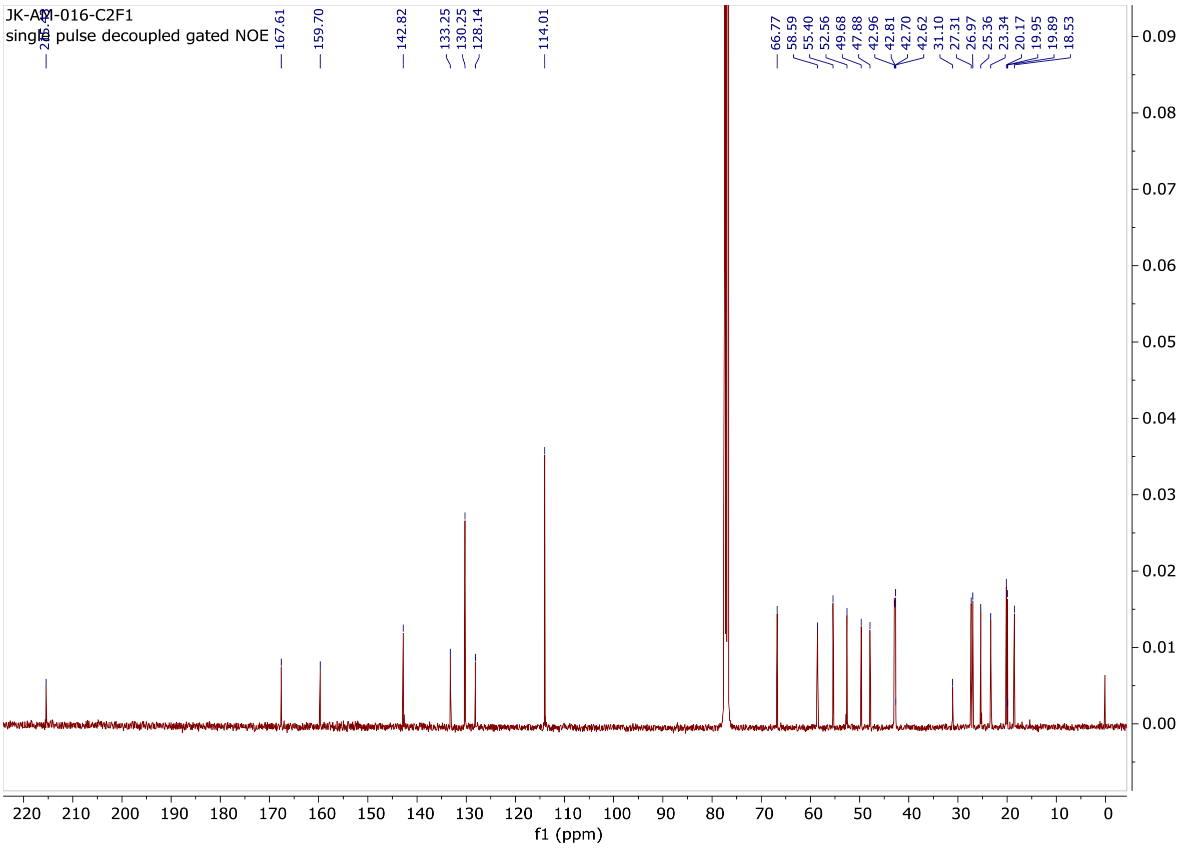

^{1}H NMR (CDCl3 400 MHz) of compound 19 (3:2 mixture of rotamers)

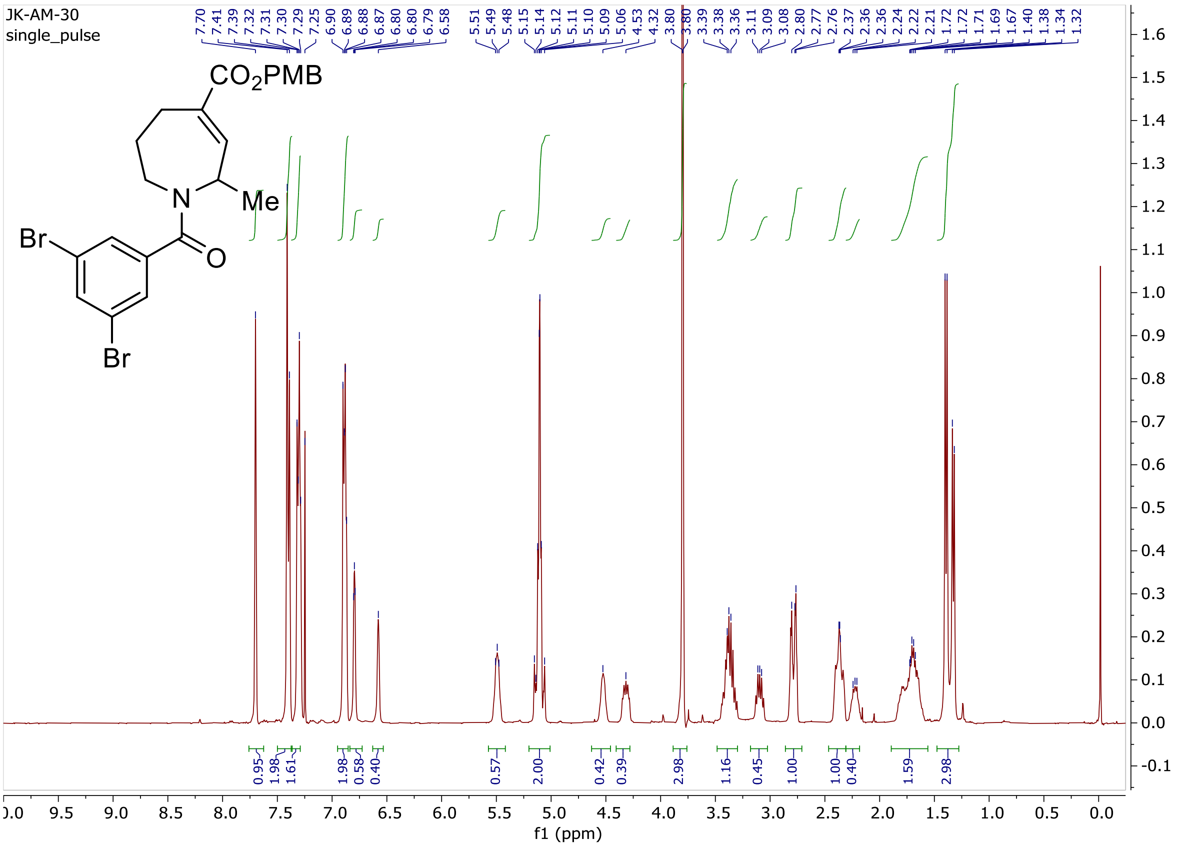

^{13}C NMR (CDCl3 100 MHz) of compound 19 (3:2 mixture of rotamers)

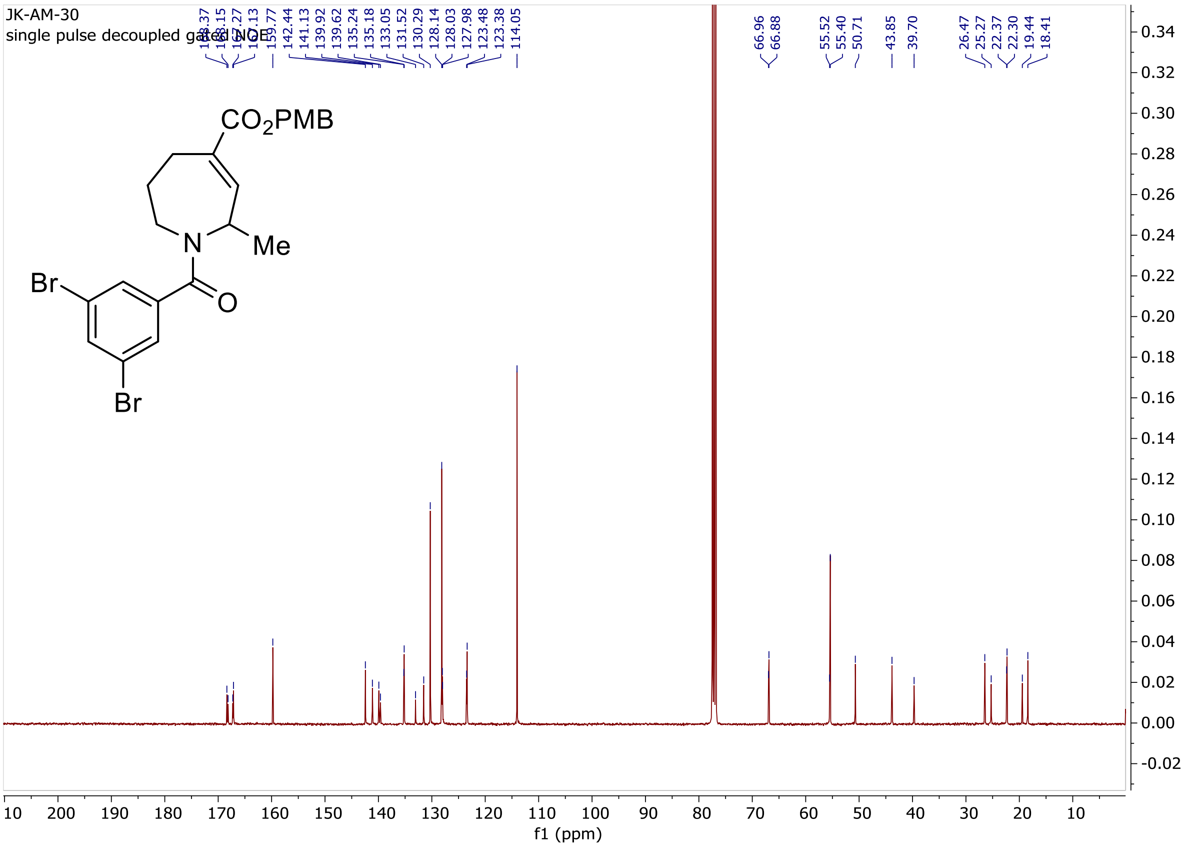

^{1}H NMR ($CDCl_3$ 400 MHz) of compound 20

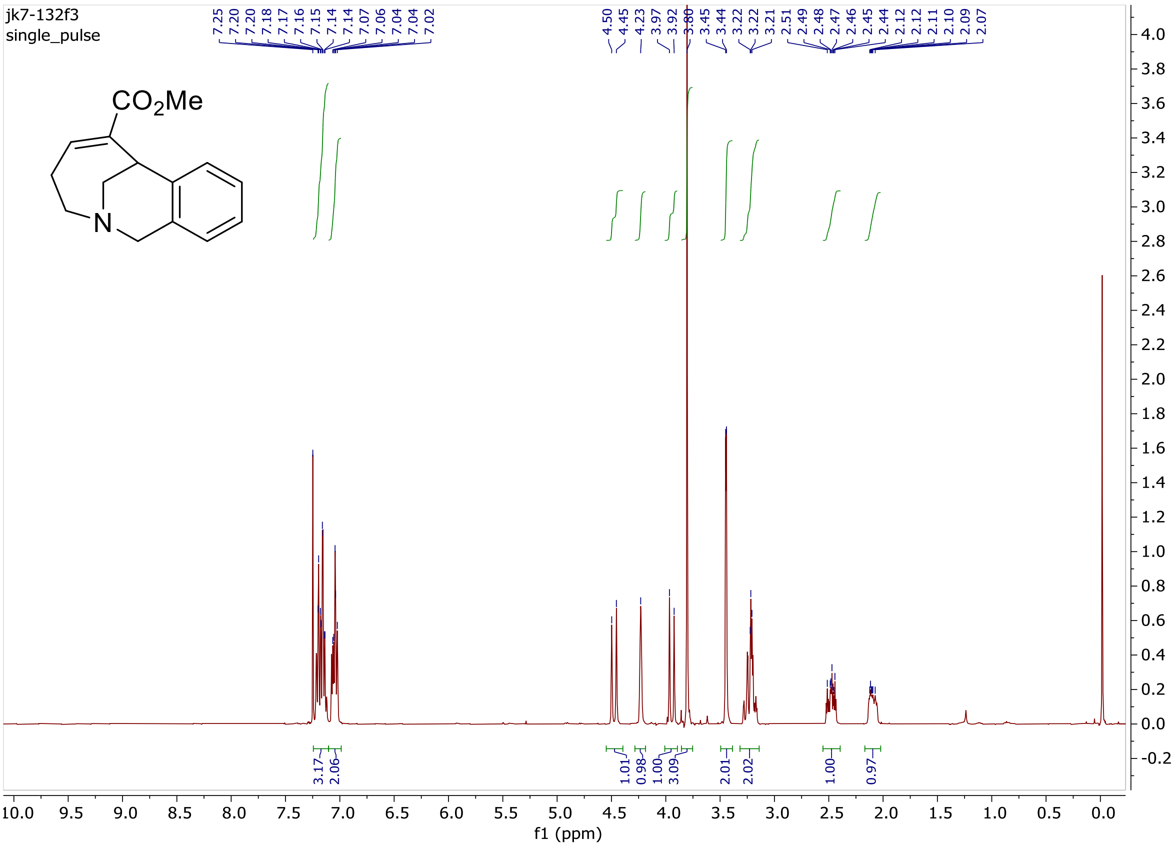

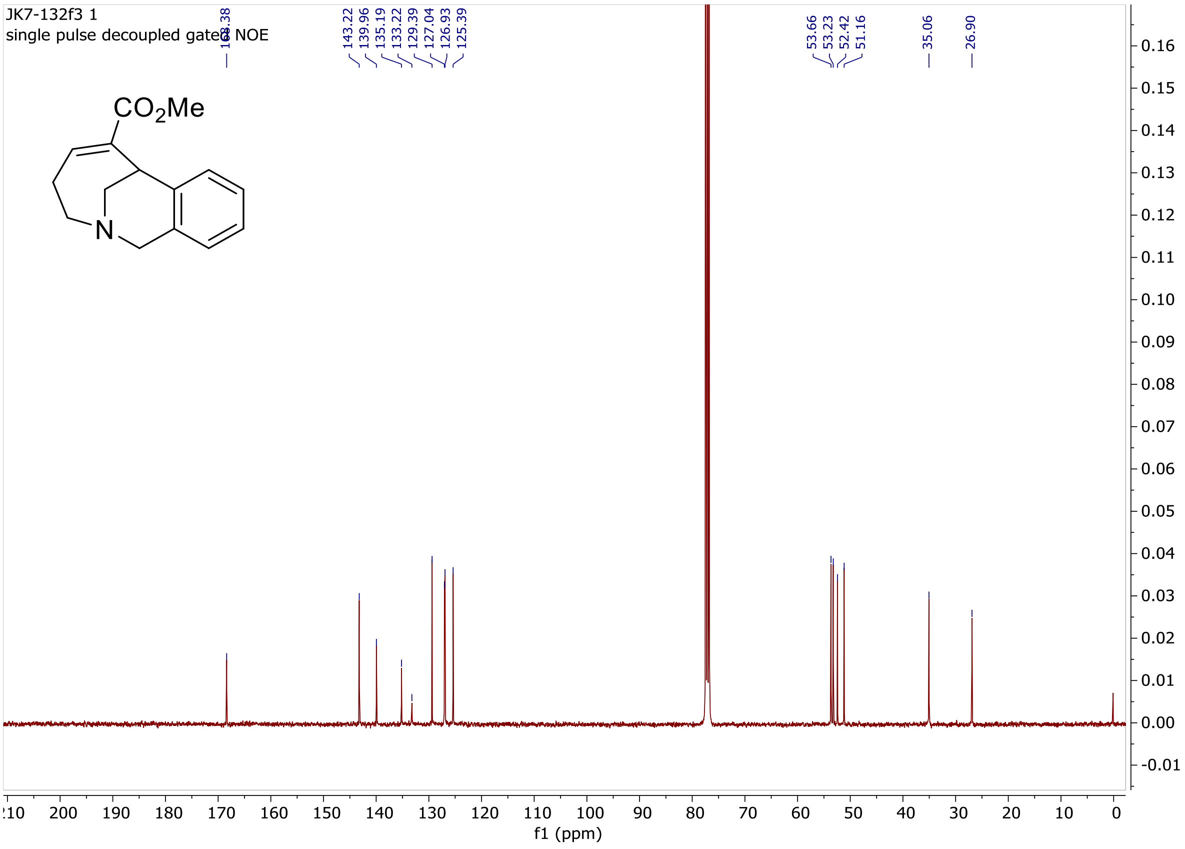

5.0 X-ray Crystallography Data

Experimental

Single crystal diffraction data were collected at 150 K on a XtaLAB Synergy HyPix-Arc 100 diffractometer using copper radiation ($\lambda_{CuK\alpha}$ = 1.54184 Å) equipped with an Oxford Cryosystems CryostreamPlus open-flow N_2 cooling device. Intensities were corrected for absorption using a multifaceted crystal model created by indexing the faces of the crystal for which data were collected.[13] Cell refinement, data collection and data reduction were undertaken via the software CrysAlisPro.[14]

All structures were solved using XT[15] and refined by XL[16] using the Olex2 interface.[17] All non-hydrogen atoms were refined anisotropically and hydrogen atoms were positioned with idealised geometry, with the exception of those bound to heteroatoms, the positions of which were located using peaks in the Fourier difference map. The displacement parameters of the hydrogen atoms were constrained using a riding model with $U_{(H)}$ set to be an appropriate multiple of the U_{eq} value of the parent atom.

Crystal structure determination of **(*S*)-6**: jkpw001_lt_fa ($C_{22}H_{25}NO_2$)

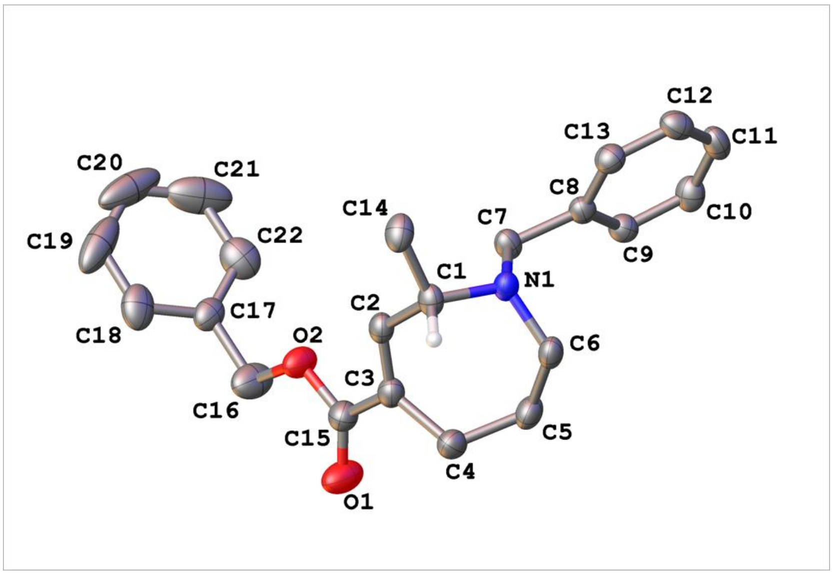

Table 1 : Crystal data and structure refinement for (S)-8b jkpw001_lt_fa.

Empirical formula	$C_{22}H_{25}NO_2$
Formula weight	335.43
Temperature/K	150.0(2)
Crystal system	monoclinic
Space group	$P2_1$
a/Å	6.9158(3)
b/Å	7.5748(3)
c/Å	17.5449(6)
α/°	90
β/°	94.912(3)
γ/°	90
Volume/Å^3	915.73(6)
Z	2
ρ_{calc}g/cm^3	1.217
μ/mm^{-1}	0.606
F(000)	360.0
Crystal size/mm^3	0.25 × 0.18 × 0.05
Radiation	Cu Kα (λ = 1.54184)
2Θ range for data collection/°	10.12 to 153.884
Index ranges	$-8 \leq h \leq 8$, $-9 \leq k \leq 8$, $-21 \leq l \leq 21$
Reflections collected	10179
Independent reflections	3474 [R_{int} = 0.0379, R_{sigma} = 0.0335]
Data/restraints/parameters	3474/187/228
Goodness-of-fit on F^2	1.079
Final R indexes [I>=2σ (I)]	R_1 = 0.0459, wR_2 = 0.1257
Final R indexes [all data]	R_1 = 0.0484, wR_2 = 0.1277
Largest diff. peak/hole / e Å^{-3}	0.19/-0.26
Flack parameter	0.1(2)

Crystal structure determination of: **S28** jkpw003_fa ($C_{35}H_{49}Cl_2NO_9S$)

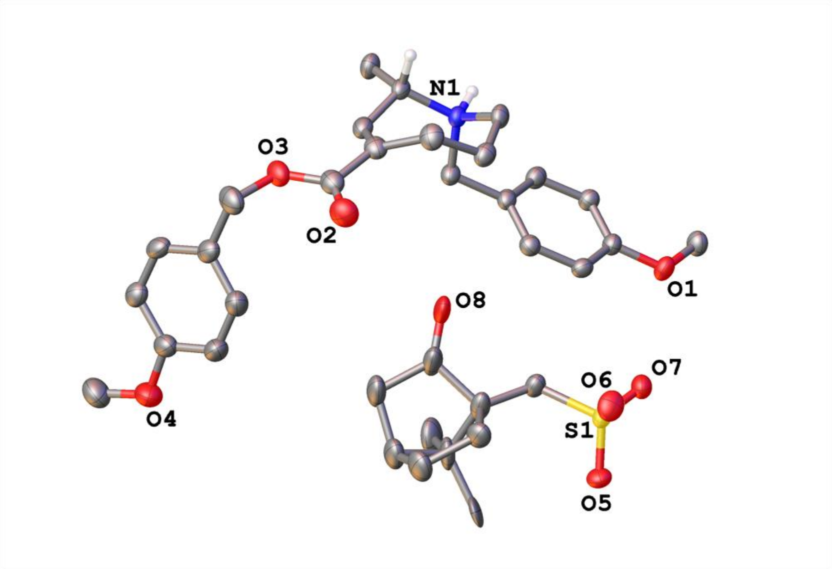

Table 1 : Crystal data and structure refinement for jkpw003_fa.

Empirical formula	$C_{35}H_{49}Cl_2NO_9S$
Formula weight	730.71
Temperature/K	150.0(2)
Crystal system	orthorhombic
Space group	$P2_12_12_1$
a/Å	7.14570(10)
b/Å	14.9447(3)
c/Å	33.7430(7)
α/°	90
β/°	90
γ/°	90
Volume/Å^3	3603.43(12)
Z	4
ρ_{calc}g/cm^3	1.347
μ/mm^{-1}	2.613
F(000)	1552.0
Crystal size/mm^3	0.25 × 0.07 × 0.01
Radiation	Cu Kα (λ = 1.54184)
2Θ range for data collection/°	5.238 to 156.494
Index ranges	$-3 \leq h \leq 8$, $-14 \leq k \leq 18$, $-40 \leq l \leq 36$
Reflections collected	18158
Independent reflections	7049 [R_{int} = 0.0363, R_{sigma} = 0.0422]
Data/restraints/parameters	7049/788/554
Goodness-of-fit on F^2	1.057
Final R indexes [I>=2σ (I)]	R_1 = 0.0530, wR_2 = 0.1233
Final R indexes [all data]	R_1 = 0.0617, wR_2 = 0.1275
Largest diff. peak/hole / e Å^{-3}	0.49/-0.35
Flack parameter	0.032(8)

6.0 Density Functional Theory Data

All DFT calculations were carried out using the Gaussian16 software package.[18] For each compound, conformation searches were performed using the confab method in Open Babel,[19] using RMSD and energy cut-offs of 1.0 Å and 5.0 kcal mol^{-1}, respectively. All identified conformers were then optimised in the gas phase at the B3LYP/6-31g(d) level,[20,21] as used successfully in previous ring expansion studies.[22] Vibrational calculations were used to verify the identification of minimum energy geometries by the absence of any calculated negative frequencies, and to determine the Gibbs energy of each structure at 298.15 K.

7b (G = -1058.21093 Ha)

C	1.014147	1.644109	2.748339
C	0.128722	2.226709	1.622414
C	1.753053	0.454465	2.099772
N	1.489522	0.575990	0.657968
C	0.142723	1.164687	0.487867
C	-0.930758	0.090597	0.797284
C	-0.083248	1.786653	-0.875174
C	0.871230	2.208996	-1.707043
C	1.812016	-0.614490	-0.120284
C	3.307568	-0.865972	-0.235327
C	3.786278	-2.176938	-0.347835
C	5.147939	-2.431387	-0.517264
C	6.056767	-1.373060	-0.565970
C	5.591471	-0.062360	-0.443840
C	4.228225	0.188510	-0.281862
O	-1.339688	-0.557643	-0.315505
O	-1.301400	-0.198971	1.918920
C	-2.288191	-1.650947	-0.134684
C	-3.715750	-1.173596	-0.225075
C	-4.367369	-1.128744	-1.464020
C	-5.684627	-0.679308	-1.558819
C	-6.365500	-0.269972	-0.410670
C	-5.723977	-0.311322	0.829170
C	-4.406371	-0.759995	0.922489
C	0.617134	2.883801	-3.024652
H	0.409920	1.302169	3.591049
H	1.713908	2.398289	3.121498
H	0.565040	3.143837	1.218818
H	-0.888476	2.450676	1.952772
H	1.367907	-0.500248	2.496193
H	2.833673	0.468505	2.274177
H	-1.128289	1.925793	-1.149755
H	1.913524	2.063172	-1.427507
H	1.391451	-0.475805	-1.122838
H	1.334089	-1.521654	0.295965
H	3.084589	-3.007509	-0.301347

H	5.499070	-3.456669	-0.602598
H	7.118521	-1.568293	-0.691662
H	6.292116	0.768510	-0.473249
H	3.865276	1.206277	-0.172871
H	-2.083835	-2.127824	0.825571
H	-2.051643	-2.340050	-0.948170
H	-3.839069	-1.450467	-2.358884
H	-6.179696	-0.652363	-2.525921
H	-7.392953	0.077244	-0.481536
H	-6.251109	0.004344	1.725628
H	-3.899910	-0.783252	1.882741
H	-0.454363	2.990987	-3.225227
H	1.063576	2.318227	-3.853760
H	1.069839	3.884474	-3.051079

8b (G = -1058.213183 Ha)

C	0.141651	-2.104162	-1.304962
C	-0.329333	-0.825886	-0.640668
C	0.726171	-3.114765	-0.306907
C	2.172509	-2.814180	0.112655
N	2.470888	-1.386076	0.226606
C	1.573954	-0.587208	1.078306
C	0.269715	-0.228258	0.404157
C	-1.595285	-0.256098	-1.194235
C	3.888637	-1.150720	0.506204
C	4.355371	0.254753	0.166975
C	5.228930	0.940210	1.018472
C	5.709332	2.207963	0.681925
C	5.312607	2.810285	-0.512064
C	4.433759	2.137468	-1.365457
C	3.960628	0.869870	-1.029344
O	-2.239126	-0.783097	-2.085645
O	-1.956673	0.917960	-0.618957
C	-3.188356	1.526640	-1.089565
C	-4.380704	1.097760	-0.269396
C	-4.776958	1.846344	0.845625
C	-5.872642	1.453036	1.614825
C	-6.585441	0.302081	1.274221
C	-6.197803	-0.451269	0.163758
C	-5.102287	-0.056649	-0.604298
C	1.334845	-1.138039	2.506983
H	0.881151	-1.876130	-2.084978
H	-0.717375	-2.543636	-1.818778
H	0.712274	-4.120281	-0.746216
H	0.069411	-3.158760	0.570257
H	2.848870	-3.204080	-0.659298
H	2.407030	-3.375864	1.037684
H	2.090133	0.373177	1.206322
H	-0.218111	0.644938	0.830108

H	4.451729	-1.864410	-0.110440
H	4.164043	-1.381208	1.553482
H	5.537571	0.476784	1.953493
H	6.387080	2.725513	1.356033
H	5.681262	3.798289	-0.775147
H	4.116945	2.602118	-2.295808
H	3.267631	0.347685	-1.682545
H	-3.317111	1.274700	-2.143715
H	-3.010405	2.599699	-0.986730
H	-4.224405	2.745314	1.110540
H	-6.171156	2.045445	2.475775
H	-7.441246	-0.004973	1.869783
H	-6.751600	-1.346562	-0.106586
H	-4.791467	-0.644040	-1.463044
H	0.783015	-2.082742	2.493994
H	0.752772	-0.421774	3.097552
H	2.286348	-1.300631	3.025569

7d (G = -1478.892052 Ha)

C	-1.124954	-0.559151	2.087227
C	-0.853309	0.913674	1.737380
C	-0.023352	-1.343979	1.339443
N	0.652733	-0.354850	0.482677
C	-0.163466	0.848389	0.335994
C	-1.307561	0.774821	-0.718670
C	0.635238	2.106554	0.052623
C	1.932406	2.272618	0.346831
C	1.330891	-0.885198	-0.691206
C	2.508468	-1.784580	-0.350346
C	2.834080	-2.859282	-1.179398
C	3.944863	-3.672421	-0.933463
C	4.751509	-3.417197	0.180159
C	4.430782	-2.350224	1.031907
C	3.327844	-1.549161	0.764731
O	5.856287	-4.144830	0.527530
C	6.224646	-5.234995	-0.299549
O	-1.667563	-0.495384	-0.995517
O	-1.850545	1.748822	-1.200744
C	-2.801116	-0.674803	-1.902764
C	-4.114205	-0.700047	-1.168900
C	-4.869781	0.464081	-0.998376
C	-6.085419	0.446589	-0.311249
C	-6.561551	-0.757074	0.223084
C	-5.812434	-1.932942	0.061147
C	-4.607397	-1.898017	-0.626601
O	-7.732254	-0.893113	0.908787
C	-8.536633	0.260043	1.103426
C	2.739592	3.484599	0.136403
C	2.216293	4.688362	-0.372891

C	3.030009	5.803038	-0.551223
C	4.388688	5.748709	-0.224766
C	4.923993	4.564666	0.282620
C	4.107490	3.448596	0.460098
H	-2.113266	-0.862405	1.730492
H	-1.098769	-0.729632	3.167910
H	-0.132234	1.357791	2.430027
H	-1.749584	1.540578	1.730129
H	-0.461056	-2.157396	0.741713
H	0.704577	-1.798578	2.021835
H	0.053299	2.905738	-0.396576
H	2.466351	1.432121	0.785988
H	1.688953	-0.026080	-1.273382
H	0.638710	-1.444434	-1.344729
H	2.209962	-3.074289	-2.044641
H	4.160697	-4.496106	-1.604660
H	5.061170	-2.171560	1.897874
H	3.078720	-0.734300	1.438111
H	7.121081	-5.665076	0.151472
H	6.455665	-4.907910	-1.322408
H	5.436775	-5.999546	-0.337603
H	-2.595580	-1.634353	-2.381693
H	-2.769505	0.122725	-2.646811
H	-4.496537	1.401629	-1.399315
H	-6.646450	1.367486	-0.200832
H	-6.200310	-2.856934	0.478328
H	-4.038836	-2.817169	-0.750155
H	-9.408866	-0.075930	1.666970
H	-8.865450	0.688706	0.147620
H	-8.006498	1.029308	1.680120
H	1.163046	4.753462	-0.629860
H	2.602621	6.721266	-0.946132
H	5.020808	6.621479	-0.364807
H	5.978407	4.507855	0.540492
H	4.531873	2.527517	0.853299

8d (G = -1478.886972 Ha)

C	1.056846	-0.739474	2.275598
C	-0.204079	-0.525687	1.461738
C	1.813924	0.567949	2.549798
C	2.637595	1.080757	1.354072
N	2.113904	0.680191	0.045001
C	0.743321	1.112607	-0.288373
C	-0.326623	0.310104	0.415766
C	-1.397648	-1.299445	1.928530
O	-2.502106	-1.107681	1.167222
O	-1.391959	-2.041224	2.895125
C	-3.687869	-1.826768	1.596442

C	-4.818875	-1.464579	0.676886
C	-5.246403	-2.341106	-0.320842
C	-6.294021	-2.011840	-1.185837
C	-6.928772	-0.771924	-1.055835
C	-6.508164	0.122286	-0.058016
C	-5.469993	-0.225417	0.793315
O	-7.959768	-0.340235	-1.838186
C	-8.429999	-1.199550	-2.864471
C	3.087734	0.942181	-1.021761
C	4.301323	0.032295	-0.955165
C	4.152974	-1.352753	-0.836646
C	5.256497	-2.207724	-0.822920
C	6.547259	-1.674251	-0.934033
C	6.712595	-0.287994	-1.052450
C	5.600552	0.547170	-1.059308
O	7.697285	-2.412805	-0.933282
C	7.589238	-3.820975	-0.808359
C	0.484795	2.628992	-0.217262
C	0.818224	3.439075	-1.313313
C	0.639546	4.821778	-1.268816
C	0.113622	5.422542	-0.123578
C	-0.236297	4.628481	0.969002
C	-0.053941	3.245156	0.920114
H	1.717528	-1.454157	1.767674
H	0.764181	-1.204920	3.220288
H	2.501250	0.423227	3.392818
H	1.094019	1.327089	2.876037
H	3.641802	0.648978	1.410848
H	2.762609	2.176389	1.430194
H	0.620752	0.847239	-1.348715
H	-1.309990	0.414987	-0.035464
H	-3.896676	-1.552646	2.635506
H	-3.474305	-2.899585	1.575308
H	-4.756910	-3.306069	-0.431259
H	-6.600947	-2.721131	-1.945834
H	-7.015866	1.077836	0.028552
H	-5.153686	0.475033	1.562621
H	-9.241683	-0.661107	-3.357038
H	-8.816262	-2.142863	-2.456084
H	-7.643558	-1.420184	-3.598354
H	3.426870	1.994638	-1.034365
H	2.573797	0.775230	-1.977908
H	3.153397	-1.768139	-0.741517
H	5.100499	-3.276441	-0.727344
H	7.719417	0.110614	-1.130372
H	5.744727	1.622090	-1.146664
H	8.611713	-4.203033	-0.828157
H	7.023166	-4.259508	-1.641148
H	7.113882	-4.108409	0.139076
H	1.213050	2.980447	-2.216981

H	0.902755	5.428298	-2.131626
H	-0.030569	6.499059	-0.087098
H	-0.658250	5.084270	1.861111
H	-0.351840	2.635681	1.768190

7f (G = -1038.330826 Ha)

C	3.496723	-2.083638	-0.607676
C	2.173584	-2.732154	-1.083529
C	3.052285	-1.013145	0.418868
N	1.651427	-0.664642	0.094367
C	1.050137	-1.970976	-0.311015
C	-0.194365	-1.751642	-1.230571
C	0.631978	-2.761609	0.922722
C	0.527966	-2.295320	2.171324
C	0.110669	-3.112703	3.360830
C	1.585167	0.410199	-0.913241
C	1.959895	1.760027	-0.327936
C	2.827756	2.617874	-1.012317
C	3.130726	3.884535	-0.506427
C	2.570103	4.307803	0.698415
C	1.705797	3.456775	1.393004
C	1.404259	2.194480	0.883981
O	-0.132611	-1.830408	-2.453301
N	-1.334209	-1.416260	-0.567909
C	-2.593226	-1.142810	-1.247396
C	-3.371667	-0.016094	-0.596777
C	-2.757041	1.206450	-0.290819
C	-3.488918	2.245868	0.281890
C	-4.848993	2.080636	0.555988
C	-5.469801	0.868046	0.256128
C	-4.732841	-0.172797	-0.312747
H	4.026826	-1.625874	-1.449800
H	4.181826	-2.808241	-0.156121
H	2.136907	-3.807170	-0.880035
H	2.014411	-2.596808	-2.155134
H	3.668885	-0.110973	0.410759
H	3.084087	-1.426212	1.431703
H	0.389651	-3.807167	0.722660
H	0.778751	-1.250243	2.350247
H	-0.129621	-4.144395	3.081986
H	-0.770161	-2.679049	3.853464
H	0.905740	-3.142390	4.118216
H	0.556854	0.468052	-1.281542
H	2.211200	0.210808	-1.799560
H	3.269417	2.292140	-1.951814
H	3.808326	4.536079	-1.052284
H	2.806213	5.291247	1.096406
H	1.266969	3.778342	2.334377
H	0.742506	1.526136	1.427213

H	-1.320158	-1.452124	0.443078
H	-3.215580	-2.048172	-1.280790
H	-2.332493	-0.903460	-2.283793
H	-1.698818	1.342504	-0.498071
H	-2.996679	3.187196	0.511780
H	-5.418914	2.891300	1.002079
H	-6.526211	0.727568	0.469568
H	-5.220737	-1.118364	-0.539579

8f (G = -1038.333464 Ha)

C	0.070940	-0.194134	0.818453
C	0.334550	-1.233397	-0.258607
C	-0.874990	-0.705070	1.918071
C	-2.360655	-0.674606	1.538477
N	-2.628679	-1.001856	0.138288
C	-2.039161	-2.249849	-0.374184
C	-0.566721	-2.128560	-0.691723
C	1.699294	-1.334999	-0.899266
O	2.067859	-2.323111	-1.531842
N	2.514130	-0.239973	-0.744250
C	3.826174	-0.155384	-1.370902
C	4.856649	0.491830	-0.467071
C	5.069024	0.013804	0.834043
C	6.031349	0.596617	1.656848
C	6.798972	1.667936	1.191202
C	6.595326	2.151277	-0.101025
C	5.627564	1.566917	-0.921672
C	-4.050212	-0.848020	-0.177414
C	-4.503339	0.603191	-0.193702
C	-3.758465	1.570481	-0.882583
C	-4.195008	2.892947	-0.943031
C	-5.386542	3.269822	-0.316689
C	-6.133792	2.315178	0.372939
C	-5.690733	0.991559	0.434852
C	-2.340709	-3.527898	0.447852
H	-0.329575	0.735160	0.387405
H	1.022769	0.065751	1.297068
H	-0.752502	-0.094899	2.821830
H	-0.563658	-1.720345	2.190438
H	-2.744902	0.339864	1.694286
H	-2.924604	-1.326320	2.234483
H	-2.515831	-2.404298	-1.352632
H	-0.198788	-2.860503	-1.409116
H	2.139868	0.603849	-0.335448
H	3.767834	0.396567	-2.320000
H	4.101255	-1.184617	-1.620589
H	4.472457	-0.818688	1.198419
H	6.186016	0.213057	2.662111
H	7.549160	2.122041	1.833110

H	7.184823	2.986027	-0.471230
H	5.469946	1.949707	-1.927843
H	-4.702572	-1.417150	0.512913
H	-4.213719	-1.274311	-1.175769
H	-2.828658	1.272681	-1.359295
H	-3.606937	3.632019	-1.481534
H	-5.726922	4.300902	-0.364541
H	-7.058600	2.599233	0.868572
H	-6.274441	0.251412	0.978526
H	-1.884653	-3.490066	1.441782
H	-1.944517	-4.411807	-0.064113
H	-3.421362	-3.665430	0.568430

7h (G = -830.366829 Ha)

C	0.391557	0.083607	2.888891
C	1.255429	-0.959483	2.135323
C	-0.489847	0.717898	1.791902
N	-0.376751	-0.208859	0.664959
C	1.038739	-0.683647	0.616638
C	1.997743	0.386779	0.047257
C	1.090547	-2.004514	-0.129642
C	2.110674	-2.518696	-0.821931
C	2.115617	-3.889345	-1.437818
C	-1.005490	0.233172	-0.570190
C	-2.522555	0.271557	-0.472167
C	2.765936	1.225151	0.870422
C	3.564564	2.240452	0.338116
C	3.616341	2.447740	-1.038792
C	2.860381	1.626399	-1.876443
C	2.065587	0.614206	-1.339635
C	-3.242605	1.329350	-1.037881
C	-4.638720	1.346762	-1.001054
C	-5.334609	0.304723	-0.387808
C	-4.624729	-0.752572	0.187456
C	-3.231090	-0.768916	0.143871
H	0.997162	0.842933	3.393704
H	-0.217444	-0.403061	3.656692
H	0.887522	-1.967382	2.346655
H	2.314418	-0.945785	2.405509
H	-0.125723	1.728935	1.532299
H	-1.539355	0.816040	2.085896
H	0.192171	-2.605704	0.021511
H	3.014838	-1.927450	-0.962035
H	1.170087	-4.414225	-1.264268
H	2.926588	-4.505469	-1.025836
H	2.283467	-3.838685	-2.522260
H	-0.721276	-0.481916	-1.354179
H	-0.641540	1.222624	-0.902362
H	2.756542	1.092474	1.946566
H	4.148501	2.867294	1.007401

H	4.238887	3.235429	-1.454756
H	2.890647	1.769383	-2.953761
H	1.499906	-0.022011	-2.011834
H	-2.704562	2.148319	-1.510932
H	-5.180448	2.177973	-1.445402
H	-6.420928	0.317289	-0.353705
H	-5.159209	-1.566677	0.670973
H	-2.673808	-1.582060	0.600324

8h (G = -830.370586)

C	1.485629	1.443208	-1.223426
C	1.641039	0.481140	-0.054514
C	0.804806	2.768632	-0.847579
C	-0.725839	2.679463	-0.768172
N	-1.212804	1.402645	-0.248557
C	-0.654334	0.961560	1.043389
C	0.724734	0.346100	0.921050
C	2.886325	-0.339827	0.002070
C	-0.720021	2.004975	2.185715
C	-2.672565	1.318854	-0.303981
C	-3.208228	-0.102102	-0.250246
C	-4.356036	-0.402395	0.491662
C	-4.890158	-1.693041	0.494226
C	-4.274143	-2.704835	-0.242509
C	-3.122289	-2.417208	-0.979864
C	-2.595005	-1.126531	-0.984797
C	2.825697	-1.708359	0.319749
C	3.981855	-2.483829	0.401758
C	5.231429	-1.910371	0.162210
C	5.310843	-0.554472	-0.161156
C	4.153404	0.218212	-0.245213
H	0.926328	0.951567	-2.032002
H	2.475768	1.656814	-1.639011
H	1.052567	3.539500	-1.588734
H	1.222039	3.115744	0.105026
H	-1.135188	2.772652	-1.782908
H	-1.110812	3.549680	-0.201488
H	-1.301511	0.131436	1.355122
H	0.972823	-0.311671	1.754194
H	-0.072199	2.865671	1.992761
H	-0.393555	1.551788	3.128533
H	-1.743880	2.369209	2.330261
H	-2.977397	1.770006	-1.258364
H	-3.168200	1.922362	0.480967
H	-4.837320	0.381967	1.072568
H	-5.782288	-1.907534	1.077153
H	-4.684679	-3.711218	-0.239524
H	-2.634105	-3.201388	-1.553229
H	-1.692564	-0.899841	-1.544825

H	1.854359	-2.166996	0.481440
H	3.904059	-3.541152	0.642421
H	6.133264	-2.513918	0.221074
H	6.278430	-0.094043	-0.344597
H	4.240830	1.275807	-0.479173

7q (G = -830.358078)

C	0.058490	2.451212	-1.442150
C	0.289089	0.940207	-1.429101
N	0.307837	0.426361	-0.056522
C	-0.975199	0.592621	0.687834
C	-1.249845	2.131457	0.692549
C	-1.232291	2.794864	-0.693986
C	-0.715515	0.232397	2.146021
C	-2.160514	-0.227692	0.113364
C	-1.511847	-0.440367	2.977230
C	1.039364	-0.832418	0.059194
C	2.549776	-0.653476	-0.033795
C	3.190788	0.443375	0.555992
C	4.580029	0.562455	0.515602
C	5.352714	-0.415411	-0.115379
C	4.723909	-1.509946	-0.710240
C	3.332836	-1.622952	-0.671382
C	-2.042019	-1.620112	-0.052634
C	-3.085874	-2.389656	-0.565040
C	-4.295948	-1.791619	-0.918609
C	-4.446233	-0.418731	-0.738393
C	-3.394785	0.347368	-0.228137
H	0.016004	2.814336	-2.476889
H	0.914092	2.939465	-0.957500
H	1.260929	0.711321	-1.877693
H	-0.477145	0.432167	-2.044325
H	-0.457283	2.584740	1.299908
H	-2.190161	2.330676	1.217092
H	-1.335078	3.881277	-0.575256
H	-2.089227	2.462499	-1.291550
H	0.218060	0.651292	2.523949
H	-2.454143	-0.875879	2.659242
H	-1.238488	-0.574277	4.020585
H	0.733489	-1.572984	-0.700995
H	0.800457	-1.272259	1.034915
H	2.584573	1.208632	1.031452
H	5.061366	1.421338	0.976985
H	6.435067	-0.321550	-0.147913
H	5.314274	-2.272555	-1.211963
H	2.847690	-2.475322	-1.143344
H	-1.130918	-2.123022	0.245104
H	-2.952954	-3.462593	-0.678973
H	-5.111320	-2.389119	-1.317508

H	-5.385284	0.066745	-0.991956
H	-3.560411	1.409080	-0.091369

8q (G = -830.361959)

C	-0.427309	1.807968	1.179304
N	-1.246015	0.774860	0.543963
C	-0.628302	0.085821	-0.603852
C	0.788168	-0.380921	-0.356823
C	1.938672	0.319785	-0.387275
C	2.124962	1.811531	-0.643883
C	0.916490	2.732442	-0.888195
C	0.020820	3.019803	0.335102
C	3.218558	-0.425425	-0.161013
C	-2.610786	1.194081	0.238386
C	-3.586607	0.032157	0.130521
C	-3.536786	-1.027667	1.046059
C	-4.464272	-2.066817	0.980684
C	-5.460086	-2.061112	0.000216
C	-5.516274	-1.011703	-0.917364
C	-4.581762	0.024336	-0.852616
C	3.464186	-1.633253	-0.837289
C	4.643622	-2.348946	-0.634289
C	5.612259	-1.870537	0.249134
C	5.388551	-0.669582	0.924318
C	4.209831	0.046411	0.716997
H	0.457163	1.317735	1.597563
H	-1.001824	2.182519	2.036101
H	-0.687250	0.698197	-1.518984
H	-1.242225	-0.798558	-0.805716
H	0.874151	-1.434221	-0.096142
H	2.804225	1.903565	-1.504249
H	2.698868	2.226827	0.198154
H	0.321591	2.358484	-1.725754
H	1.320455	3.694698	-1.231262
H	-0.858373	3.587234	0.000190
H	0.567756	3.689806	1.015576
H	-2.674743	1.796737	-0.689460
H	-2.939282	1.852841	1.053894
H	-2.751770	-1.030633	1.796926
H	-4.411269	-2.883334	1.696667
H	-6.183102	-2.871137	-0.050039
H	-6.282117	-1.001389	-1.688855
H	-4.625410	0.836860	-1.575341
H	2.724228	-1.999930	-1.542972
H	4.809001	-3.277269	-1.175182
H	6.533572	-2.424884	0.406747
H	6.132564	-0.288596	1.619306
H	4.052287	0.970197	1.266375

7.0 References

1) D. Halilovic, M. Budanovic, Z. R. Wong, R. D. Webster, J. Huh, and M. C. Stuparu, *J. Org. Chem*. **2018**, *83*, 3529.
2) C. J. Evoniuk, M. Ly and I. V. Alabugin, *Chem. Commun*. **2015**, *51*, 12831.
3) T. Shioiri, Y. Terao, N. Irako and T. Aoyama, *Tetrahedron,* **1998**, *54*, 15701.
4) Y. L. Xiao and P. H. Liu, *Angew. Chem. Int. Ed*. **2008**, *47*, 9722.
5) B. Su, M. Deng and Q. M. Wang, *Eur. J. Org. Chem*. **2013**, 1979.
6) a) H. Bittermann and P. Gmeiner, *J. Org. Chem*. **2006**, *71*, 97; b) C. Reuter, R. Opitz, A. Soicke, S. Dohmen, M. Barone, S. Chiha, M. T. Klein, J. M. Neudorfl, R. Kuhne and H. G. Schmalz, *Chem. Eur. J*. **2015**, *21*, 8464.
7) G. D. Artman, R. J. Rafferty and R. M. William, *Org. Synth*. **2009**, *86*, 262.
8) R. K. Dieter and S. J. Li, *J. Org. Chem*. **1997**, *62*, 7726.
9) N. S. Sheikh, D. Leonori, G. Barker, J. D. Firth, K. R. Campos, A. J. H. M. Meijer, P. O'Brien and I. Coldham, *J. Am. Chem. Soc*. **2012**, *134*, 5300.
10) T. K. Beng, J. S. Woo and R. E. Gawley, *J. Am. Chem. Soc*. **2012**, *134*, 14764.
11) F. J. Sayago, M. I. Calaza, A. I. Jiménez and C. Cativiela, *Tetrahedron*, **2009**, *65*, 5174.
12) J. Maury and J. Clayden, *J. Org. Chem*. **2015**, *80*, 10757.
13) R. C. Clark and J. S. Reid, *Acta Cryst*. **1995**, *A51*, 887.
14) CrysAlisPro. Rigaku Oxford Diffraction.
15) G. M. Sheldrick, *Acta Cryst*. **2015**, *A71*, 3.
16) G. M. Sheldrick, *Acta Cryst*. **2008**, *A64*, 112.
17) O. V. Dolomanov, L. J. Bourhis, R. J. Gildea, J. A. K. Howard and H. Puschmann, *J. Appl. Cryst*. **2009**, *42*, 339.
18) M. J. Frisch, G. W. Trucks, H. B. Schlegel, G. E. Scuseria, M. A. Robb, J. R. Cheeseman, G. Scalmani, V. Barone, B. Mennucci, G. A. Petersson, H. Nakatsuji, M. Caricato, X. Li, H. P. Hratchian, A. F. Izmaylov, J. Bloino, G. Zheng, J. L. Sonnenberg, M. Hada, M. Ehara, K. Toyota, R. Fukuda, J. Hasegawa, M. Ishida, T. Nakajima, Y. Honda, O. Kitao, H. Nakai, T. Vreven, J. J. A. Montgomery, J. E. Peralta, F. Ogliaro, M. Bearpark, J. J. Heyd, E. Brothers, K. N. Kudin, V. N. Staroverov, R. Kobayashi, J. Normand, K. Raghavachari, A. Rendell, J. C. Burant, S. S. Iyengar, J. Tomasi, M. Cossi, N. Rega, J. M. Millam, M. Klene, J. E. Knox, J. B. Cross, V. Bakken, C. Adamo, J. Jaramillo, R. Gomperts, R. E. Stratmann, O. Yazyev, A. J. Austin, R. Cammi, C. Pomelli, J. W. Ochterski, R. L. Martin, K. Morokuma, V. G. Zakrzewski, G. A. Voth, P. Salvador, J. J. Dannenberg, S. Dapprich, A. D. Daniels, Ö. Farkas, J. B. Foresman, J. V. Ortiz, J. Cioslowski and D. J. Fox, Gaussian 16, Revision C.01, 2019.
19) N. M. O'Boyle, M. Banck, C. A. James, C. Morley, T. Vandermeersch and G. R. Hutchison, *J. Cheminform.* **2011**, *3,* 33.
20) A. D. Becke, *J. Chem. Phys*. **1993**, *98,* 5648.
21) C. T. Lee, W. T. Yang and R. G. Parr, *Phys. Rev. B* **1988**, *37*, 785.
22) A. Lawer, R. G. Epton, T. C. Stephens, K. Y. Palate, M. Lodi, E. Marotte, K. J. Lamb, J. K. Sangha, J. M. Lynam and W. P. Unsworth, *Chem. Eur. J*. **2020**, *26*, 12674.
